# District-Wise Heterogeneity in Blood Pressure Measurements, Prehypertension, Raised Blood Pressure, and Their Determinants Among Indians: National Family Health Survey-5

**DOI:** 10.3389/ijph.2024.1606766

**Published:** 2024-03-18

**Authors:** Kavyashree Seenappa, Vaitheeswaran Kulothungan, Rohith Mohan, Prashant Mathur

**Affiliations:** Indian Council of Medical Research-National Centre for Disease Informatics and Research (ICMR-NCDIR), Bengaluru, Karnataka, India

**Keywords:** prehypertension, blood pressure, determinants, prevalence, India

## Abstract

**Objective:** The objective of the study was to determine the prevalence and determinants of ever-measured blood pressure, prehypertension, and raised blood pressure at national, state and district levels in India.

**Methods:** We analysed data from the National Family Health Survey (NFHS-5), on 743,067 adults aged 18–54 years. The sample consisted of 87.6% females and 12.4% males. We estimated prevalence rates and determined adjusted odds ratios for various dependent variables related to blood pressure. Geographical variations were visualized on the map of India, and multivariate logistic regression was employed at state and district levels, with significance set at p < 0.05.

**Results:** The prevalence of ever-measured blood pressure varied widely, from 30.3% to 98.5% across districts, with southern and northern regions showing higher rates. Prehypertension affected 33.7% of the population, with varying prevalence across districts. Raised blood pressure was there in 15.9%, with notably higher rates in southern region (16.8%). Determinants included age, gender, education, wealth, lifestyle, obesity, and blood glucose levels.

**Conclusion:** These findings demonstrate the subnational variations in blood pressure, can guide evidence-based interventions at the state and district level, towards reducing the burden of raised blood pressure and enhancing overall population health.

## Introduction

Noncommunicable diseases (NCDs) are the leading cause of mortality worldwide [1]. It is estimated that 64.9% of all deaths in India are attributed to noncommunicable diseases. Among them, cardiovascular diseases (CVDs) alone contributed to 27.4% of total mortality [2]. Hypertension is a major preventable risk factor for cardiovascular disease (CVD). On a global scale, a substantial number of individuals, i.e., 1.28 billion people, aged between 30 and 79 years were affected by hypertension, with most of them residing in low- and middle-income countries such as India [3]. In 2014, India became the first country to adopt the global NCD action plan and set national NCD targets and indicators. One of the primary targets was to achieve a 25% relative reduction in the prevalence of high blood pressure in individuals aged 18 and above by the year 2025 [4, 5]. As part of this strategy, the country has introduced population-based screening for hypertension, diabetes, cancer of breast (females), cervix and oral cavity [6, 7].

The National Family Health Survey −5 (2019–21) reported that 21% of women and 24% of men aged 15 and over have hypertension and 39% of women and 49% of men have pre-hypertension [8]. According to the National Noncommunicable Disease Monitoring Survey (NNMS, conducted in 2017–18), less than 50% of participants aged 18–49 years, reported having their blood pressure measured at some point, and 28.5% of the respondents were identified as having raised blood pressure. The burden of prehypertension, an intermediate state between normal blood pressure and hypertension, is equally concerning, as it often progresses to full-blown hypertension. The “India State-Level Disease Burden Initiative” highlighted that prehypertension contributed substantially to cardiovascular diseases, warranting urgent attention. Multiple studies have reported the rising prevalence of prehypertension in various regions of the country [9–12].

Several studies have shown considerable heterogeneity in hypertension prevalence across different states and regions of India [13–19]. However, national-level analysis fails to capture disparities within states [20]. India’s district-level administrative structure provides a unique opportunity for comprehensive health assessment and planning. Each district stands as an independent geographical and administrative unit, characterized by its unique amalgamation of health determinants, socio-economic factors, and healthcare provisions. Understanding the prevalence of pre-hypertension and raised blood pressure at the district level, can help in identifying high-risk areas and prioritizing resources accordingly.

## Methods

### Data Sources

We conducted a secondary analysis of data from the fifth wave of the National Family Health Survey (NFHS-5), covering 707 districts in 28 Indian states and 8 union territories. NFHS-5 employed a two-stage cluster sampling method with rural villages and urban census enumeration blocks as primary units. Data collection occurred in two phases: Phase I from June 17, 2019, to January 30, 2020, covering 17 states and 5 union territories, and Phase II from January 2, 2020, to April 30, 2021, covering 11 states and 3 union territories. High response rates were achieved, with data gathered from 636,699 households (98% response rate), 724,115 women (97%), and 101,839 men (92%). Over 89% of eligible women and 82% of eligible men aged 15 and older underwent blood pressure and random blood glucose measurements. Detailed information is available in the NFHS-5 India report and interviewer manual [8, 21].

### Study Participants

The present analysis included adults aged 18–54 years for males and 18–49 years for females, resulting in a final dataset of 743,067 individuals. The sample consisted of 87.6% females and 12.4% males. NFHS-5 sampled more women than men to cover more of maternal and child health indicators. Males were randomly subsampled from 15% of eligible households (state module) but were representative at national, state and district level. The average age (mean ± standard deviation) of male respondents was 34 ± 10 years, while female respondents had an average age of 32 ± 9 years. The overall sample had an average age of 32 ± 9 years.

### Main Outcomes and Variable Definitions

The main objective of this study, conducted as a secondary analysis of NFHS-5 data, was to evaluate district-wise variations in the proportions of individuals with ever-measured blood pressure, as well as to assess the prevalence of prehypertension, raised blood pressure, and their underlying determinants in India.

### Dependant Variables

Participants’ blood pressure was measured using an OMRON TM BP monitor, with three readings taken, each with a 5-min interval and a 5-min break before the first reading. The average of the last two readings was used for analysis, and if only one reading was available, it was considered for analysis (3%). Based on standard recommendations of the World Hypertension League Expert Committee raised blood pressure was defines as, systolic blood pressure (SBP) ≥ 140 mmHg or diastolic blood pressure (DBP) ≥ 90 mmHg or both on the day of survey or who reported currently taking medication for the treatment of high blood pressure, or who report having been diagnosed with hypertension by a health professional [22]. Furthermore, prehypertension was defined as an average systolic blood pressure (SBP) between 120 and 140 mmHg, or an average diastolic blood pressure (DBP) between 80 and 90 mmHg. “Ever measured” indicated individuals whose blood pressure had been assessed by a healthcare provider at least once in their lifetime.

### Independent Variables (Determinants)

**Socio-demographic factors:** Age, sex, marital status, rural or urban residence, religion, household wealth index, education, employment.

**Behavioral risk factors:** Tobacco and alcohol consumption.

**Anthropometric and metabolic factors:** BMI categories for the Asian population: underweight (18.5 kg/m^2^), normal (18.5–22.9 kg/m^2^), overweight (23–24.9 kg/m^2^), and obese (25 kg/m^2^) [23, 24]. Individuals with waist circumference values > 90 cm for men and> 80 cm for women were considered to have central obesity [24] (Supplementary Table S13: Operational definitions).

An individual was classified as having raised blood glucose if random blood glucose level >200 mg/dL on the day of the survey [25]. Biologically implausible biomarker values: SBP below 70 mmHg or above 240 mmHg, DBP below 40 mmHg or above 150 mmHg, or random blood glucose below 40 mg/dL [16, 26] were excluded, If any of the variables needed to define an indicator were not available, we set the respective indicator to missing.

### Statistical Analysis

We conducted an analysis using data from NFHS-5, focusing on participants aged 18 years and older, and incorporated individual sampling weights. Our study explored the relationships between several dependent variables: ever-measured blood pressure, prehypertension prevalence, and raised blood pressure, in relation to various determinants. Our approach involved determining sample sizes N), estimating prevalence rates with 95% confidence intervals (CIs), and calculating adjusted odds ratios (AORs) with their respective 95% CIs.

We went beyond estimating proportions and visualized the data on a color-coded map of India, categorizing it into ranges based on prevalence distributions across all districts. This visualization enabled straightforward geographical comparisons. We employed multivariate logistic regression analysis at both state and district levels and set statistical significance at *p* < 0.05. Our results were presented in tabular form, highlighting factors associated with either “Higher odds” H) or “Lower odds” L) based on the odds ratios. For data analysis, we utilized SPSS software version 27 and employed a data wrapper for visualization purposes.

## Results

### Sample Characteristics

Table 1 outlines sociodemographic characteristics. Females accounted for 87.6%, while males comprised 12.4% of the sample. The majority were in the 18–34 age group (58.5%), and 46.6% had completed secondary education. Employment was reported by 14.3%, primarily among males. Hindus made up 81.3% of the participants. In terms of wealth, the richest quintile constituted 20.7%, and the poorest 17.8%. Marriage was prevalent (78.4%), and 66.7% resided in rural areas. Tobacco use was reported by 9.3%, with 44% being males. Alcohol consumption was reported by 3.8%, primarily among males (24.9%). BMI was normal for the majority (41.2%), while central obesity affected 55.4% of the population. Normal blood glucose levels were observed in 92.2% of the population.

**TABLE 1:** Sample characteristics of analysed individuals (%) (National Family Health Survey-5, India, 2019–2021).

Characteristic	Male n (%)	Female n (%)	Total n (%)
Overall	91,900 (12.4)	651,167 (87.6)	743,067 (100)
Age Group			
18–34	48,134 (52.4)	386,564 (59.4)	434,698 (58.5)
35–49	35,071 (38.2)	264,603 (40.6)	299,674 (40.3)
50–54	8,695 (9.5)	-	8,695 (1.2)
Education			
No Education	11,690 (12.7)	159,904 (24.6)	171,594 (23.1)
Primary	11,707 (12.7)	81,279 (12.5)	92,986 (12.5)
Secondary	48,913 (53.2)	297,118 (45.6)	346,031 (46.6)
Higher	19,589 (21.3)	112,865 (17.3)	132,455 (17.8)
Occupation			
Unemployed	15,428 (16.8)	67,092 (10.3)	82,520 (11.1)
Employed	76,472 (83.2)	30,112 (4.6)	106,584 (14.3)
Religion			
Other	2,444 (2.7)	18,632 (2.9)	21,076 (2.8)
Hindu	73,085 (79.5)	530,978 (81.5)	604,063 (81.3)
Muslim	13,898 (15.1)	86,033 (13.2)	99,931 (13.4)
Christian	2,473 (2.7)	15,524 (2.4)	17,997 (2.4)
Household wealth quintile			
Poorest	14,905 (16.2)	116,995 (18)	131,901 (17.8)
Poorer	17,773 (19.3)	128,097 (19.7)	145,870 (19.6)
Middle	19,631 (21.4)	133,582 (20.5)	153,213 (20.6)
Richer	20,755 (22.6)	137,313 (21.1)	158,068 (21.3)
Richest	18,836 (20.5)	135,180 (20.8)	154,016 (20.7)
Marital status			
Others	28,193 (30.7)	132,651 (20.4)	160,845 (21.6)
Currently married	63,707 (69.3)	518,515 (79.6)	582,222 (78.4)
Regions of India			
Central region	10,456 (11.4)	164,273 (25.2)	174,729 (23.5)
Northern region	7,471 (8.1)	86,446 (13.3)	93,917 (12.6)
Eastern region	23,292 (25.3)	146,359 (22.5)	169,650 (22.8)
Western region	22,356 (24.3)	93,293 (14.3)	115,649 (15.6)
Southern region	23,279 (25.3)	136,597 (21.0)	159,876 (21.5)
North Eastern Region	5,047 (5.5)	24,199 (3.7)	29,246 (3.9)
Place Of Residence			
Rural	59,214 (64.4)	436,328 (67)	495,542 (66.7)
Urban	32,686 (35.6)	214,839 (33)	247,525 (33.3)
Tobacco consumption			
No	51,509 (56)	622,317 (95.6)	673,826 (90.7)
Yes	40,391 (44)	28,850 (4.4)	69,241 (9.3)
Alcohol consumption			
No	69,001 (75.1)	645,884 (99.2)	714,886 (96.2)
Yes	22,899 (24.9)	5,283 (0.8)	28,181 (3.8)
BMI			
Normal	36,693 (39.9)	269,464 (41.4)	306,157 (41.2)
Underweight	10,344 (11.3)	97,802 (15)	108,147 (14.6)
Overweight	16,205 (17.6)	93,953 (14.4)	110,158 (14.8)
Obese	21,767 (23.7)	161,911 (24.9)	183,679 (24.7)
Central obesity			
Present	65,147 (70.9)	346,799 (53.3)	411,946 (55.4)
Absent	19,952 (21.7)	276,112 (42.4)	296,064 (39.8)
Blood glucose level			
Normal	81,575 (88.8)	603,505 (92.7)	685,080 (92.2)
Raised	2,232 (2.4)	9,479 (1.5)	11,711 (1.6)

The population was distributed across various regions of India, with the Central region having the largest representation, encompassing 23.5% (174,729 individuals) of the population. Following closely was the eastern region, comprising 22.8% (169,650 individuals). The southern region constituted 21.5% (159,876 individuals), while the northern, western, and northeastern regions made up 12.6% (93,917 individuals), 15.6% (115,649 individuals), and 3.9% (29,246 individuals) of the population, respectively.

### Prevalence and Determinants of Ever Measured Blood Pressure in India From NFHS 5 Survey

The prevalence of ever-measured blood pressure among individuals in India was 66.7%, revealing significant regional disparities ranging from 30.3% to 98.5% across districts. The southern region led with the highest average prevalence rate of 75.8%, with standout UT/states including Lakshadweep (90.8%), Kerala (88.5%), Tamil Nadu (83.3%), and Puducherry (83.2%). The northern region also showed relatively high average prevalence rate of 69.6%, particularly notable in Chandigarh (82.6%), Punjab (82.5%), Delhi (81.9%), Haryana (78.1%), and Himachal Pradesh (76.5%). In contrast, comparatively lower prevalence rates were noted in certain regions and states, such as Madhya Pradesh (62.4%) and Chhattisgarh (62.3%) in the central region, Rajasthan (58.3%) in the north, Odisha (55.5%) and Jharkhand (59.8%) in the east, Gujarat (58.0%) in the west, and Nagaland (57.5%) in the northeast (Table 2).

**TABLE 2:** Measurement of blood pressure and prevalence of prehypertension and raised blood pressure across the states (%) (National Family Health Survey-5, India, 2019–2021).

Regions	State name	Ever measured blood pressure (%)	Pre - hypertension (%)	Raised blood pressure (%)
Central region	Uttarakhand	74.1 (72.9–75.3)	35.3 (34.0–36.5)	17.4 (16.5–18.4)
	Uttar Pradesh	63.9 (63.6–64.2)	35.2 (34.9–35.5)	17.2 (16.9–17.4)
	Chhattisgarh	62.3 (61.5–63.0)	38.8 (38.0–39.5)	17.6 (17.0–18.2)
	Madhya Pradesh	62.4 (61.9–62.9)	35.5 (35.0–36.0)	14.3 (13.9–14.6)
	Overall Central Region	63.7 (63.5–64.0)	35.6 (35.4–35.8)	16.5 (16.4–16.7)
Northern region	Jammu and Kashmir	71.6 (70.5–72.6)	45.2 (44.1–46.4)	13.3 (12.6–14.1)
	Himachal Pradesh	76.5 (75.2–77.8)	35.3 (33.9–36.8)	16.7 (15.6–17.8)
	Punjab	82.5 (81.9–83.2)	32.2 (31.5–33.0)	25.8 (25.1–26.5)
	Chandigarh	82.6 (79.2–85.8)	28.6 (25.1–32.5)	19.4 (16.3–22.7)
	Haryana	78.1 (77.4–78.9)	36.6 (35.8–37.5)	18.2 (17.6–18.9)
	NCT Of Delhi	81.9 (81.1–82.6)	35.2 (34.3–36.1)	18.6 (17.8–19.3)
	Rajasthan	58.3 (57.8–58.8)	43.5 (43.0–43.9)	12.7 (12.3–13.0)
	Ladakh	72.3 (63.9–79.3)	48.8 (40.1–57.0)	18.1 (12.3–25.4)
	Overall Northern Region	69.6 (69.3–69.9)	39.4 (39.0–39.7)	16.6 (16.4–16.8)
Eastern region	Bihar	62.7 (62.3–63.1)	26.5 (26.2–26.9)	17.6 (17.3–17.9)
	West Bengal	64.8 (64.4–65.2)	32.3 (32.0–32.7)	13.3 (13.1–13.6)
	Jharkhand	59.8 (59.0–60.5)	38.1 (37.4–38.8)	15.2 (14.7–15.7)
	Odisha	55.5 (54.9–56.2)	34.4 (33.8–35.1)	17.2 (16.8–17.7)
	Overall Eastern Region	62.2 (62.0–62.4)	31.1 (30.9–31.3)	15.6 (15.4–15.8)
Western region	Gujarat	58.0 (57.5–58.5)	35.5 (35.0–36.0)	12.9 (12.6–13.2)
	Dadra and Nagar Haveli and Daman and Diu	71.1 (65.9–76.1)	37.0 (31.8–42.5)	10.1 (7.2–13.9)
	Maharashtra	64.5 (64.1–64.8)	34.5 (34.2–34.8)	13.8 (13.6–14.1)
	Goa	85.8 (83.7–87.8)	21.0 (18.8–23.4)	12.8 (11.0–14.8)
	Overall Western Region	62.5 (62.3–62.8)	34.7 (34.4–35.0)	13.5 (13.3–13.7)
Southern region	Andhra Pradesh	74.7 (74.2–75.2)	29.8 (29.3–30.3)	16.6 (16.2–17.0)
	Karnataka	61.5 (61.1–62.0)	30.7 (30.2–31.1)	16.2 (15.9–16.6)
	Lakshadweep	90.8 (80.2–96.9)	40.1 (26.0–53.5)	12.1 (5.6–24.9)
	Kerala	88.5 (88.0–88.9)	32.9 (32.2–33.5)	15.5 (15.0–16.0)
	Tamil Nadu	83.3 (82.9–83.6)	29.7 (29.3–30.2)	17.9 (17.5–18.3)
	Puducherry	83.2 (80.3–86.0)	27.7 (24.5–31.2)	13.1 (10.7–15.8)
	Andaman and Nicobar Islands	85.7 (80.8–89.9)	36.9 (30.8–43.2)	18.8 (14.2–24.3)
	Telangana	78.0 (77.4–78.6)	28.2 (27.6–28.8)	17.3 (16.8–17.8)
	Overall Southern Region	75.8 (75.5–76.0)	30.2 (30.0–30.4)	16.8 (16.6–17.0)
Northeastern Region	Sikkim	75.5 (70.7–79.6)	34.6 (30.0–39.5)	29.1 (24.8–33.9)
	Arunachal Pradesh	65.1 (61.1–69.0)	42.2 (38.1–46.2)	24.6 (21.3–28.3)
	Nagaland	57.5 (54.2–60.8)	40.1 (36.9–43.4)	18.3 (15.8–20.9)
	Manipur	84.6 (82.6–86.4)	37.3 (34.8–39.8)	19.5 (17.4–21.6)
	Mizoram	80.9 (77.7–83.9)	34.5 (30.9–38.2)	17.3 (14.6–20.5)
	Tripura	73.0 (71.2–74.7)	33.8 (31.9–35.6)	17.5 (16.1–19.1)
	Meghalaya	61.0 (58.7–63.1)	35.3 (33.2–37.5)	17.0 (15.4–18.7)
	Assam	67.0 (66.3–67.6)	35.1 (34.4–35.7)	15.3 (14.8–15.8)
	Overall Northeastern Region	68.1 (67.5–68.6)	35.4 (34.8–35.9)	16.3 (15.9–16.7)
Overall, India	Total	66.7 (66.6–66.8)	33.7 (33.6–33.8)	15.9 (15.8–16.0)

**FIGURE 1:**
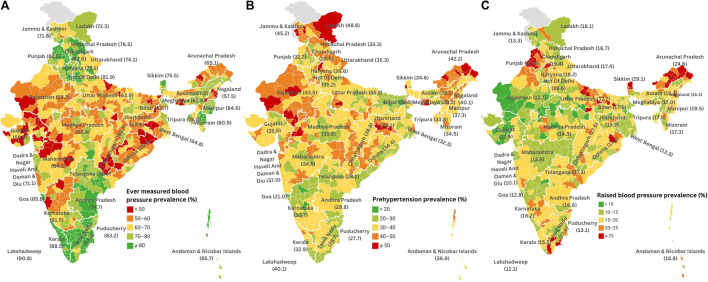
**(A)** District-wise prevalence of ever measured blood pressure among adults in India (heat map) (National Family Health Survey-5, India, 2019–2021). **(B)** District-wise prevalence of prehypertension among adults in India (heat map) (National Family Health Survey-5, India, 2019–2021). **(C)** District-wise prevalence of raised blood pressure among adults in India (heat map) (National Family Health Survey-5, India, 2019–2021).

To enhance data visualization on a color-coded map of India, districts were classified into five groups based on the prevalence of individuals reporting ever-measured blood pressure. The highest category (80.0%–98.5%) included 116 districts (16.4%), while 183 districts (25.9%) fell within the range of 70.1%–80.0%. The majority (27.0%) recorded rates between 60.1% and 70.0%, with 22.2% of districts falling between 50.1% and 60.0%. The lowest range of 30.3%–50.0% was observed in 8.5% of districts. Notably, Mahe in Puducherry had the highest rates of ever-measured blood pressure at 98.5%, while districts like East Garo Hills in Meghalaya (30.3%), Khargone (West Nimar) in Madhya Pradesh (32.2%), North Garo Hills in Meghalaya (33.2%), Alirajpur in Madhya Pradesh (33.8%), and Kodagaon in Chhattisgarh (36.4%) exhibited the lowest rates (Table 3; Figure 1A).

**TABLE 3:** Prevalence (%) of ever measured blood pressure, prehypertension and raised blood pressure among Indian districts (20 Good performers and 20 bad performers) (National Family Health Survey-5, India, 2019–2021).

Prevalence of ever measured blood pressure				Prevalence of prehypertension			Prevalence of raised blood pressure		
**Good performers**									
**Sl. No**	**State name**	**District name**	**Percentage (%)**	**State name**	**District name**	**Percentage (%)**	**State name**	**District name**	**Percentage (%)**
1	Puducherry	Mahe	98.5	Madhya Pradesh	Bhopal	15.6	Rajasthan	Barmer	4.1
2	Puducherry	Yanam	94.2	Bihar	Purnia	16.3	Uttar Pradesh	Kaushambi	5.1
3	Kerala	Kannur	93.6	Bihar	Katihar	17	Madhya Pradesh	Tikamgarh	5.8
4	Tamil Nadu	Tiruppur	93.5	Bihar	Vaishali	17.2	Rajasthan	Jalor	6.0
5	Tamil Nadu	Coimbatore	93.3	Bihar	Begusarai	17.6	Madhya Pradesh	Agar Malwa	6.2
6	Kerala	Kozhikode	92.3	Karnataka	Bagalkot	18.6	Gujarat	Jamnagar	6.8
7	Kerala	Malappuram	92.2	Karnataka	Davanagere	19.5	Gujarat	Botad	6.9
8	Kerala	Wayanad	91.8	Goa	North Goa	19.9	Rajasthan	Baran	7.2
9	Tamil Nadu	Salem	91.6	Bihar	Madhepura	20.1	Haryana	Gurgaon	7.3
10	Goa	South Goa	91.5	Bihar	Kishanganj	20.4	Madhya Pradesh	Khandwa (East Nimar)	7.5
11	Tamil Nadu	Theni	91.4	Madhya Pradesh	Agar Malwa	20.7	Rajasthan	Jaisalmer	7.5
12	Punjab	Gurdaspur	91.1	Bihar	Khagaria	20.7	Madhya Pradesh	Singrauli	7.7
13	Lakshadweep	Lakshadweep	90.8	Karnataka	Yadgir	20.9	Rajasthan	Jhalawar	7.7
14	Punjab	Ludhiana	90.7	Goa	Overall Goa	21	Rajasthan	Karauli	7.9
15	Kerala	Kasaragod	90.5	Tamil Nadu	Thiruvallur	21.2	Gujarat	Amreli	8.2
16	Tamil Nadu	Dindigul	90.3	Telangana	Siddipet	21.2	Karnataka	Yadgir	8.2
17	Tamil Nadu	The Nilgiris	90.3	West Bengal	Bankura	21.3	Madhya Pradesh	Raisen	8.2
18	Kerala	Pathanamthitta	90.2	West Bengal	South Twenty-Four Parganas	21.3	Gujarat	Surendranagar	8.4
19	Mizoram	Aizawl	90.2	Bihar	Araria	21.4	Jammu and Kashmir	Kathua	8.6
20	NCT Of Delhi	Southwest	90.1	Telangana	Medak	21.4	Jammu and Kashmir	Shupiyan	8.7
**Bad performers**									
**Sl. NO**	**State name**	**District name**	**Percentage (%)**	**State name**	**District name**	**Percentage (%)**	**State name**	**District name**	**Percentage (%)**
1	Meghalaya	East Garo Hills	30.3	Jammu and Kashmir	Rajouri	63.4	Sikkim	North District	38.6
2	Madhya Pradesh	Khargone (West Nimar)	32.2	Meghalaya	South Garo Hills	56.8	Punjab	Bathinda	38.5
3	Meghalaya	North Garo Hills	33.2	Jammu and Kashmir	Anantnag	55.8	Punjab	Firozpur	35.2
4	Madhya Pradesh	Alirajpur	33.8	Rajasthan	Barmer	55.3	Sikkim	South District	35.0
5	Chhattisgarh	Kodagaon	36.4	Ladakh	Leh (Ladakh)	54.3	Punjab	Faridkot	33.0
6	Chhattisgarh	Narayanpur	38.2	Arunachal Pradesh	Dibang Valley	53.2	Tamil Nadu	Madurai	31.4
7	Meghalaya	South Garo Hills	38.9	Uttar Pradesh	Hamirpur	52.8	Arunachal Pradesh	Dibang Valley	30.5
8	Chhattisgarh	Bastar	39.1	Jammu and Kashmir	Punch	52.4	Haryana	Kurukshetra	30.3
9	Nagaland	Tuensang	39.4	Madhya Pradesh	Alirajpur	52.3	Uttar Pradesh	Gonda	30.2
10	Gujarat	Kheda	39.4	Sikkim	West District	52.3	Arunachal Pradesh	West Siang	29.9
11	Odisha	Mayurbhanj	40.0	Jharkhand	Gumla	51.8	Arunachal Pradesh	Papum Pare	29.8
12	Gujarat	Botad	40.9	Meghalaya	East Garo Hills	51.7	Punjab	Moga	29.4
13	Uttar Pradesh	Sant Kabir Nagar	41.1	Uttar Pradesh	Shamli	51.6	Arunachal Pradesh	Tawang	29.3
14	Meghalaya	Southwest Garo Hills	41.2	Rajasthan	Banswara	51.5	Arunachal Pradesh	Lower Subansiri	29.3
15	Maharashtra	Nandurbar	41.6	Rajasthan	Udaipur	51.5	Haryana	Yamunanagar	29.2
16	Uttar Pradesh	Shrawasti	41.8	Puducherry	Mahe	51.5	Arunachal Pradesh	East Siang	29.1
17	Maharashtra	Jalgaon	41.9	Rajasthan	Nagaur	51.4	Tamil Nadu	Ramanathapuram	29.0
18	Gujarat	Dohad	42.0	Arunachal Pradesh	Anjaw	51.2	Punjab	Gurdaspur	28.7
19	Odisha	Nuapada	42.1	Uttar Pradesh	Saharanpur	51.1	Arunachal Pradesh	Upper Subansiri	28.7
20	West Bengal	Puruliya	42.3	Jharkhand	Khunti	51.1	Punjab	Mansa	28.4

At the national level, various factors were associated with individuals having their blood pressure measured. Individuals over the age of 30 years (aOR 1.38), females (aOR 1.79), literate individuals (aOR 1.24), those belonging to middle or rich household wealth index (aOR 1.31 and aOR 1.55, respectively), married individuals (aOR 2.13), urban residents (aOR 1.23), alcohol consumers (aOR 1.26), overweight or obese individuals, and those with raised blood glucose levels (aOR 1.52), were more likely to have their blood pressure measured compared to their counterparts (Table 4).

**TABLE 4:** Measurement of blood pressure and prevalence of prehypertension and raised blood pressure and their determinants in Indian population (National Family Health Survey-5, India, 2019–2021).

Subgroups	Ever measured blood pressure			Prehypertension			Raised blood pressure		
	*n*	Prevalence (%)	AOR	n	Prevalence (%)	AOR	n	Prevalence (%)	AOR
		(95% CI)	(95% CI)		(95% CI)	(95% CI)		(95% CI)	(95% CI)
Overall	707,843	66.7 (66.6–66.8)		743,067	33.7 (33.6–33.8)		743,067	15.9 (15.8–16.0)	
Age Group									
<30	304,546	59.6 (59.4–59.7)	1	321,205	27.8 (27.6–27.9)	1	321,205	8.2 (8.1–8.3)	1
≥30	403,297	72.1 (72.0–72.2)	1.38 (1.35–1.41)	421,862	38.3 (38.1–38.4)	1.31 (1.28–1.34)	421,862	21.8 (21.7–21.9)	2.35 (2.27–2.43)
Sex									
Male	85,043	54.3 (54.0–54.7)	1	91,900	42.4 (42.1–42.8)	1	91,900	19.2 (18.9–19.4)	1
Female	622,800	68.4 (68.3–68.5)	1.79 (1.74–1.84)	651,167	32.5 (32.4–32.6)	0.60 (0.58–0.61)	651,167	15.5 (15.4–15.5)	0.72 (0.69–0.75)
Education									
Illiterate	165,288	63.5 (63.3–63.7)	1	171,594	37.1 (36.8–37.3)	1	171,594	20.0 (19.8–20.2)	1
Literate	542,555	67.7 (67.5–67.8)	1.24 (1.21–1.28)	571,472	32.7 (32.6–32.8)	0.91 (0.89–0.94)	571,472	14.7 (14.6–14.8)	0.84 (0.81–0.87)
Occupation									
Unemployed	77,918	64.8 (64.5–65.2)	1	82,520	33.1 (32.8–33.4)	1	82,520	15.0 (14.7–15.2)	1
Employed	100,019	59.7 (59.4–60.0)	1.01 (0.99–1.04)	106,584	40.4 (40.2–40.7)	1.04 (1.01–1.06)	106,584	19.1 (18.8–19.3)	0.92 (0.89–0.95)
Household wealth quintile	177,937	61.9 (61.7–62.2)		189,104	37.2 (37.0–37.5)		189,104	17.3 (17.1–17.5)	
Poorest/Poorer	268,642	59.0 (58.8–59.1)	1	277,770	33.4 (33.2–33.5)	1	277,770	14.8 (14.7–15.0)	1
Middle	147,711	67.2 (67.0–67.5)	1.31 (1.27–1.35)	153,213	33.4 (33.2–33.7)	1.00 (0.97–1.02)	153,213	16.3 (16.2–16.5)	1.11 (1.07–1.15)
Richer/Richest	291,490	73.6 (73.4–73.7)	1.55 (1.51–1.60)	312,083	34.2 (34.0–34.4)	1.08 (1.05–1.11)	312,083	16.7 (16.5–16.8)	1.06 (1.02–1.10)
Marital status									
Others	150,108	47.0 (46.8–47.3)	1	160,845	30.0 (29.8–30.3)	1	160,845	10.1 (9.9–10.2)	1
Currently married	557,735	72.0 (71.9–72.1)	2.13 (2.07–2.18)	582,222	34.8 (34.6–34.9)	0.97 (0.94–0.99)	582,222	17.5 (17.4–17.6)	1.19 (1.14–1.23)
Place Of Residence									
Rural	478,833	63.9 (63.8–64.0)	1	495,542	33.8 (33.7–33.9)	1	495,542	15.7 (15.6–15.8)	1
Urban	229,009	72.6 (72.4–72.7)	1.23 (1.20–1.26)	247,525	33.6 (33.4–33.8)	1.01 (0.99–1.04)	247,525	16.3 (16.2–16.5)	1.01 (0.98–1.04)
Tobacco consumption									
No	641,882	67.7 (67.6–67.8)	1	673,826	33.1 (33.0–33.2)	1	673,826	15.5 (15.4–15.6)	1
Yes	65,961	56.7 (56.3–57.0)	0.79 (0.77–0.82)	69,241	39.5 (39.2–39.9)	1.00 (0.97–1.02)	69,241	20.1 (19.8–20.4)	1.01 (0.97–1.04)
Alcohol consumption									
No	681,364	67.0 (66.9–67.1)	1	714,886	33.5 (33.3–33.6)	1	714,886	15.6 (15.5–15.7)	1
Yes	26,479	59.3 (58.7–59.9)	1.26 (1.22–1.30)	28,181	40.7 (40.1–41.3)	0.93 (0.90–0.96)	28,181	24.1 (23.6–24.6)	1.38 (1.32–1.43)
BMI									
Normal/Underweight	412,944	60.9 (60.7–61.0)	1	414,304	32.5 (32.3–32.6)	1	414,304	11.8 (11.7–11.9)	1
Overweight/Obese	292,870	74.9 (74.7–75.0)	1.22 (1.19–1.25)	293,837	39.3 (39.1–39.5)	1.21 (1.18–1.24)	293,837	23.5 (23.3–23.6)	1.49 (1.44–1.54)
Central obesity									
Absent	410,769	60.3 (60.2–60.5)	1	411,946	33.2 (33.1–33.4)	1	411,946	12.3 (12.2–12.4)	1
Present	295,169	75.5 (75.4–75.7)	1.33 (1.30–1.37)	296,064	38.3 (38.1–38.4)	1.04 (1.01–1.07)	296,064	22.7 (22.6–22.9)	1.63 (1.58–1.68)
Blood glucose level									
Normal	683,918	66.4 (66.2–66.5)	1	685,080	35.6 (35.5–35.7)	1	685,080	15.7 (15.6–15.8)	1
Raised	11,691	81.4 (80.7–82.1)	1.52 (1.40–1.65)	11,711	34.6 (33.7–35.4)	0.65 (0.60–0.69)	11,711	44.8 (43.9–45.7)	2.47 (2.31–2.64)

*p* value <0.05 is considered statistically significant.

At the state level, age was a significant factor, with individuals aged over 30 years having higher odds of ever measuring blood pressure in 17 states (47.2%). Gender also played a role, with females associated with higher odds in 21 states (58.3%). Education was linked to higher odds in 8 states (22.2%). Household wealth, specifically middle and rich wealth indices, showed positive associations in 11 states (30.6%) and 15 states (41.7%), respectively. Marriage and urban residence were positive factors, with 23 states (63.9%) and 10 states (27.8%) showing higher odds. Alcohol consumption, obesity/overweight, central obesity, and raised blood glucose were associated with higher odds in a few states, ranging from 2 states (5.6%) for alcohol consumption to 15 states (41.7%) for obesity/overweight (detailed in Supplementary Table S7).

Similar patterns were observed at the district level, with age, gender, education, household wealth, marriage, and urban residence serving as significant determinants linked to higher odds of having ever measured blood pressure in various districts. Furthermore, alcohol consumption, obesity/overweight, central obesity, and raised blood glucose showed associations with higher odds, with marriage being the most prevalent factor in28.5% of districts (Detailed in Supplementary Table S10).

### Prevalence and Determinants of Prehypertension in India From NFHS-5 Survey

Prehypertension prevalence varied widely across Indian districts, with an overall rate of 33.7% (95% CI: 33.6–33.8), ranging from 15.6% to 63.4%. The Southern region had a lower average prevalence at 30.2%, including Puducherry (27.7%), Telengana (28.2%), Tamil Nadu (29.7%), and Andhra Pradesh (29.8%), with relatively lower rates. The northern region also performed well, with an average rate of 39.4%, with Himachal Pradesh (35.3%) and Chandigarh (28.6%) showing lower rates. Conversely, Jammu and Kashmir (45.2%), Ladakh (48.8%), and Rajasthan (43.5%) in the north, and Chhattisgarh (38.8%) in the central region had higher prehypertension rates (Table 2).

To enhance data visualization on a color-coded map of India, districts were categorized into five groups based on prehypertension prevalence percentages. The highest range (50.1%–63.4%) included 25 districts (3.5%), while 165 districts (23.3%) fell in the 40.1%–50.0% range. The majority, 347 districts (49.1%), had prevalence rates between 30.1% and 40.0%. Prevalence rates between 20.1% and 30.0% were observed in 162 districts (22.9%), with only 8 districts (1.1%) having the lowest range of 15.6%–20.0%. Notably, Bhopal in Madhya Pradesh had the lowest rate at 15.6%, while Rajouri (63.4%) and Anantnag (55.8%) in Jammu and Kashmir had the highest rates. Bihar and Karnataka had the lowest rates in the top 20 districts, while Rajasthan and Jammu and Kashmir had the highest rates in the bottom 20 districts (Table 3; Figure 1B). Detailed district-wise data is available in Supplementary Tables S1–S6.

Various factors were associated with prevalence of prehypertension at the national level. Individuals aged over 30 years had higher odds of being prehypertensive (aOR 1.31), with notably high rates (27.8%) among younger individuals. Higher odds of prehypertension were observed in individuals from wealthier households (aOR 1.08) and those overweight or obese (aOR 1.21).

Conversely, females (aOR 0.60), literate individuals (aOR 0.91), alcohol consumers (aOR 0.93), and individuals with elevated blood glucose levels (aOR 0.65) had lower odds of being prehypertensive compared to their counterparts. There was no statistically significant link between tobacco consumption and the prevalence of prehypertension (Table 4).

Age over 30 was associated with higher odds in several states and districts, while being female was linked to lower odds in many areas. Literacy generally lowered the odds of prehypertension. Employment had mixed effects, with both higher and lower odds observed. Household wealth showed diverse impacts in a few regions. Marriage and urban residence were associated with lower odds in several places. Tobacco and alcohol consumption had varying effects, and obesity, particularly obesity/overweight, was consistently linked to higher odds. Central obesity also showed higher odds in a few districts. Raised blood glucose was associated with lower odds in some areas (detailed in Supplementary Tables S8–S11).

### Prevalence and Determinants (Sociodemographic and Behavioural) of Raised Blood Pressure in India From NFHS5 Survey

The prevalence of raised blood pressure in India was found to be 15.9% (95% CI: 15.8–16.0), exhibiting considerable variation across districts, ranging from 4.1% to 51.8%.

The southern region performed relatively better with a lower average raised blood pressure prevalence rate of 16.8%, showcasing states such as Lakshadweep (12.1%), Kerala (15.5%), and Tamil Nadu (17.9%) had lower rates. The northern region also demonstrated lower average prevalence, with an average rate of 16.6%. This region included states like Himachal Pradesh (16.7%), Chandigarh (19.4%), and Delhi (18.6%) which displayed higher rates. Conversely, some regions and states exhibited higher prevalence rates of raised blood pressure. The Northeastern region, with an average prevalence rate of 16.3%, encompassed states like Sikkim (29.1%) and Arunachal Pradesh (24.6%) with higher prevalence rates. States in the Central region showed varying rates, with Madhya Pradesh having a relatively lower prevalence rate (14.3%) (Table 2).

To enhance data visualization on a color-coded map of India, the districts were classified into five groups based on raised blood pressure prevalence percentages. The highest range (25.1%–51.8%) encompassed 129 districts (18.2%), while 85 districts (12.0%) fell in the 20.1%–25.0% range. Most districts, 258 (36.5%), had prevalence rates between 15.1% and 20.0%. Rates of 10.1%–15.0% were observed in 192 districts (27.2%), and 43 districts (6.0%) had the lowest range of 4.1%–10.0%. Among low-prevalence districts, Barmer in Rajasthan had the lowest at 4.1%. Conversely, high-prevalence districts included North and South Districts in Sikkim, Bathinda, Firozpur, and Faridkot in Punjab. Arunachal Pradesh had the most districts [7] among the bottom 20 with high rates, while Rajasthan and Madhya Pradesh had the most (7 and 5, respectively) low-prevalence districts among the top 20 (Table 3; Figure 1C). Detailed district-wise data is available in Supplementary Tables S1–S6.

The prevalence of raised blood pressure at the national level was associated with several determinants. Individuals over the age of 30 years (aOR 2.35) had higher odds of having raised blood pressure; however, the prevalence rate was also high even among younger age groups (8.2%). Belonging to wealthier households (aOR 1.11), being married (aOR 1.19), and consuming alcohol (aOR 1.38) were associated with higher odds of having raised blood pressure. Being overweight or obese (aOR 1.49), having central obesity (aOR 1.63), and having raised blood glucose levels (aOR 2.47) were also associated with higher odds of raised blood pressure. On the other hand, females (aOR 0.72), literate individuals (aOR 0.84), and employed individuals (aOR 0.92) had lower odds of having raised blood pressure. However, no statistically significant link was found between place of residence, tobacco consumption, and the prevalence of raised blood pressure (Table 4).

In the state-level analysis, most states (75.0%) exhibited higher odds of raised blood pressure among individuals over 30 years old. Female gender was associated with lower odds in half of the states (50.0%), while education was linked to lower odds in 22.2%. Employment status predominantly indicated lower odds in 13.9% of states, while the household wealth index showed higher odds in 13.4% (middle) and 11.1% (rich) of states. Marriage correlated with higher odds in 22.2% of states, and urban residence had varying odds in 11.1%. Tobacco consumption had mixed effects, while alcohol consumption was associated with higher odds in 27.8%. Both obesity/overweight and central obesity were associated with higher odds in 58.3% and 63.9% of states, respectively, with raised blood glucose associated with higher odds in 55.6% of states (detailed in Supplementary Table S9).

At the district level, Individuals aged over-30 had higher raised blood pressure odds in 33.0%% of districts. Female gender had lower odds in 14.2%%, education in 6.2%%, and employment had lower odds in 4.5% of districts. Household wealth index had raised odds in 4.5% (middle) and 5.9% (rich) of districts. Married individuals had higher odds in 6.2%, while urban residence varied in 8.0% districts. Alcohol consumption was associated with higher odds in 8.3% of districts, and obesity/overweight and central obesity was associated with higher odds in 17.5% and 17.1%, respectively. Raised blood glucose linked to higher odds in 10.4% of districts (Supplementary Table S12).

## Discussion

This study offers crucial insights into the prevalence and determinants of blood pressure measurement, prehypertension and raised blood pressure at national, state, and district levels in India.

Ever measured blood pressure rates, prevalence of prehypertension and raised blood pressure exhibited wide variations across the states and districts. The states of southern region were better performing when compared to others. The regional disparities highlighted in our study are consistent with numerous other studies conducted in India, illustrating similar inter-state and intra-state disparities [18, 19, 27–30]. For example, a multilevel analysis conducted in the state of Maharashtra revealed variations in raised blood pressure prevalence across the districts, with rates ranging from 15% in Hingoli to 36% in Mumbai. Districts such as Satara, Dhule, Gadchiroli, and Mumbai have a high blood pressure prevalence of over 30%, while Hingoli, Nagpur, Osmanabad, Wardha, and Akola have a prevalence rate below 20% [27].

These disparities can be attributed to various factors, including differences in healthcare infrastructure, socio-economic conditions, lifestyle choices, and urban-rural divides. Addressing these multifaceted factors is crucial for reducing healthcare disparities and enhancing raised BP-related health outcomes in India, both at the state and district levels.

The study investigated various sociodemographic and behaviour factors linked to blood pressure measurement and the prevalence of prehypertension and raised blood pressure. Age was a significant factor, with older individuals having higher odds of these conditions [19, 28, 31–33]. These findings were consistent with prior research, including a repeated cross-sectional analysis conducted using NFHS data [34]. However, there is a growing concern about the rising rates of prehypertension and elevated blood pressure in younger individuals [12, 35, 36]. The health system in India mainly focuses on screening the older adult population [7] and most health promotion efforts target middle-aged and elderly populations. Therefore, there is a need to develop or adopt successful strategies, such as the life course approach, which has been effective in preventing NCDs and emphasizes early screening and diagnosis. Implementing interventions in schools, colleges, and workplaces is crucial for reaching adolescents and younger adults.

Women are more likely to have their blood pressure checked, possibly due to ante-natal care services, and they also have a lower probability of experiencing prehypertension and raised blood pressure [37]. These findings align with previous studies highlighting women’s health-conscious and proactive healthcare-seeking behaviour [37, 38]. In contrast, men tend to exhibit suboptimal health-seeking behaviour, regardless of the specific medical condition [39, 40]. They often seek medical attention only during emergencies or when chronic illnesses have already advanced [41]. Hence encouraging men to seek healthcare proactively is crucial, particularly through health education and opportunistic screening.

Education and wealth played important roles, with higher educational attainment associated with a higher likelihood of blood pressure measurement and a lower likelihood of prehypertension. Wealthier individuals had increased odds of blood pressure measurement, prehypertension and raised blood pressure, which is consistent with findings from previous studies [14, 15, 42]. This reflects the influence of economic status on healthcare access and lifestyle factors.

Urban residents had higher odds of having their blood pressure measured, likely benefiting from improved healthcare access. However the prevalence of raised blood pressure did not vary significantly, which is consistent with some previous study [11]. Conversely, several studies in India have highlighted rural-urban discrepancies in raised blood pressure prevalence [14, 20, 32, 38]. This may indicate a potential narrowing of the urban-rural divide, even concerning other non-communicable diseases and their associated risk factors [43].

Alcohol consumption was associated with higher odds of ever measured blood pressure and raised blood pressure prevalence. General and central obesity, along with raised blood glucose levels, were consistently associated with higher odds of raised blood pressure aligning with numerous studies conducted in India that have examined the impact of alcohol consumption, tobacco use, obesity, and elevated blood glucose levels on ever-measured blood pressure, prevalence of prehypertension, and raised blood pressure [19, 27, 28, 44–48].

Despite India’s pioneering role in adopting the global NCD action plan and setting national targets, achieving a 25% relative reduction in high blood pressure prevalence among adults aged 18 and above by 2025 proved challenging. This difficulty was highlighted by the study’s findings of a 10.1% decrease in age-standardized premature mortality rates (ASPMR) and a 9.3% decrease in the underlying potential years of life lost (UPoD) for cardiovascular diseases (CVD) between 2010 and 2025, indicating some progress. However, the lack of significant decline from 2001 to 2019 revealed a failure to meet the WHO’s reduction targets for CVD, resulting in a shortfall of over 15% and 25% respectively, by 2025 [49].

Our study’s identification of district-level variations and specific determinants is vital for policymakers and healthcare providers. It informs targeted interventions for prehypertension and raised blood pressure in India, enabling cost-effective approaches and tailored health policies at state and district levels. Learning from successful districts can uplift care in underperforming areas, enhancing raised blood pressure care nationwide.

The study has several strengths, including its large sample size, standardized data collection, comprehensive assessment, district-level analysis, and inclusion of various determinants. However, it also has some limitations, such as single day measurement of blood pressure, which might have overestimated prevalence of prehypertension and raised blood pressure, alcohol consumption was a significant determinant We have considered data on current alcohol consumption, i.e., who respondent yes to the question do you consume alcohol, from NFHS 5 survey. However, we did not use detailed data on the amount of alcohol, drinking patterns (such as occasional use, abuse, binge drinking), or the type of alcoholic beverage consumed for our analysis, the cross-sectional design hinders establishing causal relationships. Additionally, sampling bias may have excluded certain population groups. Despite these limitations, the study offers a valuable foundation for monitoring raised blood pressure care in India and identifying areas for enhancement.

### Conclusion

Our study sheds light on the varying landscape of blood pressure measurement, prehypertension, and raised blood pressure prevalence in India. These variations underscore the urgent need for targeted interventions to address healthcare disparities, especially among vulnerable populations. Strategies should encompass health education, healthcare access, and awareness campaigns, promoting proactive healthcare-seeking behaviour, particularly among men. Factors like age, gender, education, wealth, and urban residence influence these conditions, while factors like alcohol consumption, obesity, and elevated blood glucose levels need attention, highlighting the need for targeted interventions.

Leveraging existing national programs like National Programme for Prevention and Control of Non-Communicable Diseases and Ayushman Arogya Mandir can provide a solid foundation for evidence-based interventions to enhance raised blood pressure care across diverse regions. Aligning efforts with national programs is a crucial approach, and insights from successful districts can guide strategies to uplift underperforming areas, ultimately reducing the burden of raised blood pressure across India’s varied regions.

## Data Availability

Publicly available datasets were analysed in this study. The comprehensive dataset employed in this study can be accessed at: https://www.dhsprogram.com.
