# Supplementary material for: District-Wise Heterogeneity in Blood Pressure Measurements, Prehypertension, Raised Blood Pressure, and Their Determinants Among Indians: National Family Health Survey-5
Source: Int J Public Health. 2024 Mar 18;69:1606766. doi: 10.3389/ijph.2024.1606766 (PMC10982880; doi:10.3389/ijph.2024.1606766)
Supplement: Supplementary file 1 [file DataSheet1.docx]

## **Journal Name: International Journal of Public Health**

**Article Title:**

**District-Wise Heterogeneity in Blood Pressure Measurements, Prehypertension, Raised Blood Pressure, and their Determinants among Indians: National Family Health Survey-5**

## **Supplementary materials table of content**

Figure S1: Number of adults included for analysis (Flow chart) (National Family Health Survey-5, India, 2019-2021)

Table S1: District wise prevalence of Ever measured blood pressure (BP), Prehypertension, and Raised blood pressure in the states of northern region of India (%) (National Family Health Survey-5, India, 2019-2021)

Table S2: District wise prevalence of Ever measured blood pressure (BP), Prehypertension and Raised blood pressure in the states of central region of India (%) (National Family Health Survey-5, India, 2019-2021)

Table S3: District wise prevalence of Ever measured blood pressure (BP), Prehypertension, and Raised blood pressure in the states of Eastern region of India (%) (National Family Health Survey-5, India, 2019-2021)

Table S4: District wise prevalence of Ever measured blood pressure (BP), Prehypertension, and Raised blood pressure in the states of western region of India (%) (National Family Health Survey-5, India, 2019-2021)

Table S5: District wise prevalence of Ever measured blood pressure (BP), Prehypertension and Raised blood pressure in the states of southern region of India (%) (National Family Health Survey-5, India, 2019-2021)

Table S6: District wise prevalence of Ever measured blood pressure (BP), Prehypertension and Raised blood pressure in the states of northeastern region of India (%) (National Family Health Survey-5, India, 2019-2021)

Table S7: State wise determinants of Ever measured blood pressure among adults in India (AOR) (National Family Health Survey-5, India, 2019-2021)

Table S8: State wise determinants Prehypertension among adults in India (AOR) (National Family Health Survey-5, India, 2019-2021)

Table S9: State wise determinants of Raised blood pressure among adults in India (AOR) (National Family Health Survey-5, India, 2019-2021)

Table S10: District wise determinants of Ever measured blood pressure among adults in India (AOR) (National Family Health Survey-5, India, 2019-2021)

Table S11: District wise determinants of Prehypertension among adults in India (AOR) (National Family Health Survey-5, India, 2019-2021)

Table S12: District wise determinants of Raised blood pressure among adults in India (AOR) (National Family Health Survey-5, India, 2019-2021)

Table S13: Operational definitions (National Family Health Survey-5, India, 2019-2021)

## **Figure S1: Number of adults included for analysis (Flow chart) (National Family Health Survey-5, India, 2019-2021)**

**Total Number of individual interviews conducted in NFHS 5 survey N= 825,954**

**(Women aged 15-49 years + Men aged15-54 years)**

**Population included for final analysis of prevalence of prehypertension and raised blood pressure =743,067 (90.0%))**

**(Women 18-49 age + Men 18-54 age)**

**Excluded individuals who are<18 years of age (82,887/825,954=10.0%)**

**Excluded individuals with no response to question on ever measured blood pressure 35,224(4.2%)**

**Population included for analysis of prevalence of ever measured blood pressure 707,843 (85.7%)**

## **Table S1: District wise prevalence of Ever measured blood pressure (BP), Prehypertension, and Raised blood pressure in the states of northern region of India (%) (National Family Health Survey-5, India, 2019-2021)**

| Sl. NO | State name | District name | A. | B | C | D |
| --- | --- | --- | --- | --- | --- | --- |
|  |  |  | **Total population analysed** | **Ever measured blood pressure among the population (B/A)** | **Prevalence of prehypertension in the population(C/A)** | **Prevalence of raised BP in the population(D/A)** |
|  |  |  | **n(%)** | **n(%)** | **n(%)** | **n(%)** |
| Overall Indian population | | | **743067 (100.0)** | **472130 (66.7)** | **250624 (33.7)** | **118231 (15.9)** |
| Overall North Region | | | **93917 (12.6)** | **62201 (69.6)** | **36965 (39.4)** | **15589 (16.6)** |
| 1 | **1.Jammu & Kashmir** | Kupwara | 405 (5.5) | 247 (63.3) | 193 (47.8) | 53 (13.0) |
| 2 |  | Badgam | 365 (5.0) | 269 (75.7) | 166 (45.5) | 53 (14.4) |
| 3 |  | Punch | 316 (4.3) | 215 (69.1) | 166 (52.4) | 43 (13.5) |
| 4 |  | Rajouri | 440 (6.0) | 317 (72.6) | 279 (63.4) | 56 (12.8) |
| 5 |  | Kathua | 410 (5.6) | 330 (82.0) | 103 (25.1) | 35 (8.6) |
| 6 |  | Baramula | 649 (8.9) | 426 (67.8) | 261 (40.2) | 106 (16.4) |
| 7 |  | Bandipore | 229 (3.1) | 133 (60.8) | 75 (32.9) | 43 (18.7) |
| 8 |  | Srinagar | 723 (9.9) | 514 (74.7) | 333 (46.0) | 105 (14.6) |
| 9 |  | Ganderbal | 165 (2.3) | 107 (66.6) | 58 (35.2) | 26 (15.8) |
| 10 |  | Pulwama | 281 (3.8) | 198 (75.5) | 132 (47.0) | 39 (13.7) |
| 11 |  | Shupiyan | 150 (2.0) | 104 (74.6) | 65 (43.8) | 13 (8.7) |
| 12 |  | Anantnag | 562 (7.7) | 380 (71.0) | 314 (55.8) | 52 (9.2) |
| 13 |  | Kulgam | 263 (3.6) | 176 (72.3) | 117 (44.6) | 30 (11.5) |
| 14 |  | Doda | 241 (3.3) | 140 (59.7) | 110 (45.6) | 29 (12.1) |
| 15 |  | Ramban | 200 (2.7) | 149 (74.7) | 98 (49.0) | 35 (17.3) |
| 16 |  | Kishtwar | 165 (2.3) | 106 (65.5) | 56 (34.0) | 18 (11.2) |
| 17 |  | Udhampur | 333 (4.6) | 247 (75.4) | 157 (47.0) | 49 (14.6) |
| 18 |  | Reasi | 188 (2.6) | 125 (67.4) | 94 (49.9) | 20 (10.9) |
| 19 |  | Jammu | 1023 (14.0) | 740 (74.2) | 437 (42.7) | 139 (13.6) |
| 20 |  | Samba | 205 (2.8) | 146 (71.8) | 93 (45.1) | 31 (15.2) |
|  |  | Overall Jammu & Kashmir | 7313 (100.0) | 5068 (71.6) | 3307 (45.2) | 976 (13.3) |
| 1 | **2.Himachal Pradesh** | Chamba | 283 (6.6) | 207 (74.4) | 121 (42.9) | 45 (15.9) |
| 2 |  | Kangra | 989 (23.1) | 635 (67.6) | 287 (29.0) | 176 (17.8) |
| 3 |  | Lahul & Spiti | 14 (0.3) | 12 (82.6) | 6 (40.7) | 2 (11.6) |
| 4 |  | Kullu | 270 (6.3) | 202 (76.3) | 95 (35.2) | 47 (17.2) |
| 5 |  | Mandi | 627 (14.6) | 506 (81.6) | 221 (35.3) | 79 (12.6) |
| 6 |  | Hamirpur | 263 (6.1) | 208 (80.0) | 72 (27.5) | 52 (19.9) |
| 7 |  | Una | 318 (7.4) | 224 (73.2) | 84 (26.4) | 75 (23.6) |
| 8 |  | Bilaspur | 221 (5.2) | 163 (74.9) | 68 (30.7) | 36 (16.3) |
| 9 |  | Solan | 402 (9.4) | 307 (78.8) | 164 (40.8) | 73 (18.1) |
| 10 |  | Sirmaur | 319 (7.4) | 253 (80.4) | 125 (39.2) | 52 (16.3) |
| 11 |  | Shimla | 536 (12.5) | 441 (83.5) | 256 (47.7) | 74 (13.7) |
| 12 |  | Kinnaur | 46 (1.1) | 39 (84.9) | 17 (36.7) | 5 (11.7) |
|  |  | Overall Himachal Pradesh | 4289 (100.0) | 3197 (76.5) | 1516 (35.3) | 716 (16.7) |
| 1 | **3.Punjab** | Kapurthala | 453 (2.9) | 374 (87.3) | 168 (37.2) | 113 (24.9) |
| 2 |  | Jalandhar | 1197 (7.7) | 868 (83.9) | 353 (29.5) | 279 (23.3) |
| 3 |  | Hoshiarpur | 937 (6.1) | 737 (80.9) | 401 (42.8) | 241 (25.7) |
| 4 |  | Shahid Bhagat Singh Nagar | 353 (2.3) | 297 (85.6) | 153 (43.4) | 96 (27.2) |
| 5 |  | Fatehgarh Sahib | 321 (2.1) | 246 (84.8) | 89 (27.8) | 74 (23.0) |
| 6 |  | Ludhiana | 1762 (11.4) | 1361 (90.7) | 517 (29.4) | 377 (21.4) |
| 7 |  | Moga | 545 (3.5) | 424 (80.1) | 191 (35.1) | 160 (29.4) |
| 8 |  | Muktsar | 510 (3.3) | 362 (74.3) | 160 (31.4) | 138 (27.0) |
| 9 |  | Faridkot | 335 (2.2) | 262 (81.5) | 88 (26.4) | 111 (33.0) |
| 10 |  | Bathinda | 844 (5.5) | 606 (78.2) | 194 (23.0) | 325 (38.5) |
| 11 |  | Mansa | 442 (2.9) | 277 (65.5) | 156 (35.3) | 125 (28.4) |
| 12 |  | Patiala | 1160 (7.5) | 861 (89.9) | 305 (26.3) | 226 (19.5) |
| 13 |  | Amritsar | 1362 (8.8) | 1036 (84.5) | 509 (37.4) | 373 (27.4) |
| 14 |  | Tarn Taran | 597 (3.9) | 490 (90.1) | 191 (32.0) | 167 (28.1) |
| 15 |  | Rupnagar | 391 (2.5) | 326 (89.2) | 133 (33.9) | 68 (17.5) |
| 16 |  | Sahibzada Ajit Singh Nagar | 592 (3.8) | 482 (88.9) | 156 (26.3) | 132 (22.4) |
| 17 |  | Sangrur | 971 (6.3) | 598 (68.2) | 315 (32.4) | 242 (24.9) |
| 18 |  | Barnala | 337 (2.2) | 220 (69.5) | 132 (39.3) | 78 (23.1) |
| 19 |  | Fazilka | 691 (4.5) | 468 (70.7) | 211 (30.6) | 176 (25.5) |
| 20 |  | Firozpur | 435 (2.8) | 330 (76.7) | 132 (30.4) | 153 (35.2) |
| 21 |  | Gurdaspur | 889 (5.7) | 701 (91.1) | 302 (34.0) | 255 (28.7) |
| 22 |  | Pathankot | 345 (2.2) | 276 (86.5) | 130 (37.5) | 79 (23.0) |
|  |  | Overall Punjab | 15468 (100.0) | 11602 (82.5) | 4986 (32.2) | 3989 (25.8) |
| 1 | **4.Chandigarh** | Chandigarh | 579 (100.0) | 421 (82.6) | 166 (28.6) | 112 (19.4) |
| 2 |  | Overall Chandigarh | 579 (0.1) | 421 (82.6) | 166 (28.6) | 112 (19.4) |
| 1 | **5.Haryana** | Panchkula | 294 (2.1) | 224 (83.7) | 97 (32.9) | 46 (15.5) |
| 2 |  | Ambala | 573 (4.2) | 417 (74.5) | 194 (33.9) | 157 (27.4) |
| 3 |  | Yamunanagar | 611 (4.5) | 464 (76.6) | 177 (29.1) | 178 (29.2) |
| 4 |  | Kurukshetra | 535 (3.9) | 382 (73.8) | 212 (39.7) | 162 (30.3) |
| 5 |  | Kaithal | 562 (4.1) | 439 (87.9) | 170 (30.3) | 59 (10.5) |
| 6 |  | Karnal | 918 (6.7) | 742 (82.1) | 389 (42.4) | 189 (20.6) |
| 7 |  | Panipat | 671 (4.9) | 472 (75.6) | 193 (28.8) | 134 (20.0) |
| 8 |  | Sonipat | 790 (5.8) | 618 (79.8) | 267 (33.7) | 154 (19.6) |
| 9 |  | Jind | 727 (5.3) | 542 (80.3) | 247 (34.0) | 108 (14.9) |
| 10 |  | Fatehabad | 545 (4.0) | 400 (78.8) | 180 (33.0) | 68 (12.4) |
| 11 |  | Sirsa | 660 (4.8) | 479 (77.0) | 203 (30.7) | 156 (23.6) |
| 12 |  | Hisar | 1002 (7.3) | 713 (76.0) | 312 (31.1) | 198 (19.7) |
| 13 |  | Rohtak | 589 (4.3) | 433 (81.0) | 155 (26.4) | 129 (22.0) |
| 14 |  | Jhajjar | 533 (3.9) | 429 (84.2) | 217 (40.7) | 92 (17.3) |
| 15 |  | Mahendragarh | 473 (3.5) | 337 (75.6) | 192 (40.7) | 62 (13.1) |
| 16 |  | Rewari | 508 (3.7) | 369 (75.8) | 249 (48.9) | 71 (14.0) |
| 17 |  | Gurgaon | 823 (6.0) | 573 (83.4) | 307 (37.3) | 60 (7.3) |
| 18 |  | Mewat | 470 (3.4) | 307 (68.3) | 206 (43.9) | 50 (10.6) |
| 19 |  | Faridabad | 918 (6.7) | 735 (81.3) | 433 (47.2) | 176 (19.1) |
| 20 |  | Palwal | 554 (4.0) | 370 (68.4) | 251 (45.3) | 109 (19.7) |
| 21 |  | Bhiwani | 661 (4.8) | 462 (74.0) | 262 (39.7) | 100 (15.2) |
| 22 |  | Charkhi Dadri | 285 (2.1) | 210 (78.8) | 106 (37.3) | 41 (14.2) |
|  |  | Overall Haryana | 13700 (100.0) | 10116 (78.1) | 5021 (36.6) | 2499 (18.2) |
| 1 | **6.NCT Of Delhi** | Central | 910 (8.4) | 659 (80.0) | 296 (32.5) | 208 (22.9) |
| 2 |  | East | 928 (8.6) | 724 (81.6) | 417 (44.9) | 173 (18.6) |
| 3 |  | New Delhi | 627 (5.8) | 441 (82.2) | 190 (30.3) | 128 (20.4) |
| 4 |  | North | 945 (8.8) | 637 (75.5) | 248 (26.3) | 212 (22.4) |
| 5 |  | North East | 909 (8.4) | 682 (84.2) | 349 (38.4) | 183 (20.1) |
| 6 |  | North West | 1363 (12.6) | 1019 (84.0) | 420 (30.8) | 248 (18.2) |
| 7 |  | Shahdara | 786 (7.3) | 536 (78.1) | 276 (35.1) | 137 (17.5) |
| 8 |  | South | 710 (6.6) | 552 (85.5) | 306 (43.1) | 107 (15.1) |
| 9 |  | South East | 973 (9.0) | 801 (88.1) | 458 (47.0) | 151 (15.5) |
| 10 |  | South West | 869 (8.1) | 704 (90.1) | 282 (32.5) | 117 (13.5) |
| 11 |  | West | 1751 (16.3) | 1207 (75.9) | 554 (31.6) | 335 (19.1) |
|  |  | Overall NCT Of Delhi | 10771 (100.0) | 7962 (81.9) | 3796 (35.2) | 2000 (18.6) |
| 1 | **7.Rajasthan** | Ganganagar | 1340 (3.2) | 861 (65.0) | 544 (40.6) | 296 (22.1) |
| 2 |  | Hanumangarh | 1190 (2.9) | 727 (62.0) | 478 (40.2) | 273 (23.0) |
| 3 |  | Bikaner | 1466 (3.5) | 918 (63.5) | 644 (43.9) | 225 (15.3) |
| 4 |  | Churu | 1387 (3.3) | 769 (57.3) | 559 (40.3) | 325 (23.4) |
| 5 |  | Jhunjhunun | 1359 (3.3) | 762 (57.0) | 535 (39.4) | 327 (24.0) |
| 6 |  | Alwar | 2027 (4.9) | 1109 (58.1) | 721 (35.6) | 314 (15.5) |
| 7 |  | Bharatpur | 1261 (3.0) | 680 (56.4) | 410 (32.5) | 171 (13.6) |
| 8 |  | Dhaulpur | 635 (1.5) | 367 (58.9) | 213 (33.5) | 71 (11.2) |
| 9 |  | Karauli | 756 (1.8) | 426 (57.6) | 227 (30.1) | 60 (7.9) |
| 10 |  | Sawai Madhopur | 781 (1.9) | 427 (57.9) | 320 (41.0) | 81 (10.4) |
| 11 |  | Dausa | 921 (2.2) | 579 (64.0) | 304 (33.0) | 92 (10.0) |
| 12 |  | Jaipur | 3449 (8.3) | 2295 (68.4) | 1224 (35.5) | 385 (11.1) |
| 13 |  | Sikar | 1737 (4.2) | 893 (52.8) | 817 (47.1) | 309 (17.8) |
| 14 |  | Nagaur | 2399 (5.8) | 1192 (50.2) | 1234 (51.4) | 284 (11.8) |
| 15 |  | Jodhpur | 2480 (6.0) | 1252 (50.9) | 1229 (49.5) | 252 (10.1) |
| 16 |  | Jaisalmer | 435 (1.0) | 231 (53.1) | 213 (49.0) | 33 (7.5) |
| 17 |  | Barmer | 1675 (4.0) | 813 (48.7) | 926 (55.3) | 69 (4.1) |
| 18 |  | Jalor | 1303 (3.1) | 755 (58.4) | 646 (49.6) | 78 (6.0) |
| 19 |  | Sirohi | 671 (1.6) | 310 (47.0) | 319 (47.5) | 64 (9.5) |
| 20 |  | Pali | 1316 (3.2) | 859 (67.5) | 506 (38.4) | 123 (9.3) |
| 21 |  | Ajmer | 1538 (3.7) | 916 (61.2) | 595 (38.7) | 244 (15.9) |
| 22 |  | Tonk | 812 (1.9) | 432 (55.5) | 359 (44.2) | 95 (11.6) |
| 23 |  | Bundi | 689 (1.7) | 389 (57.3) | 281 (40.8) | 89 (13.0) |
| 24 |  | Bhilwara | 898 (2.2) | 504 (57.5) | 387 (43.1) | 79 (8.8) |
| 25 |  | Rajsamand | 750 (1.8) | 408 (55.3) | 353 (47.0) | 110 (14.7) |
| 26 |  | Dungarpur | 968 (2.3) | 461 (48.0) | 448 (46.3) | 96 (9.9) |
| 27 |  | Banswara | 1061 (2.5) | 596 (58.0) | 547 (51.5) | 111 (10.5) |
| 28 |  | Chittaurgarh | 904 (2.2) | 580 (66.6) | 404 (44.7) | 96 (10.6) |
| 29 |  | Kota | 1287 (3.1) | 833 (65.9) | 638 (49.6) | 121 (9.4) |
| 30 |  | Baran | 795 (1.9) | 404 (51.6) | 347 (43.6) | 57 (7.2) |
| 31 |  | Jhalawar | 880 (2.1) | 453 (52.6) | 403 (45.8) | 68 (7.7) |
| 32 |  | Udaipur | 1961 (4.7) | 1268 (65.7) | 1009 (51.5) | 223 (11.4) |
| 33 |  | Pratapgarh | 534 (1.3) | 274 (53.1) | 270 (50.5) | 54 (10.2) |
|  |  | Overall Rajasthan | 41665 (100.0) | 23743 (58.3) | 18110 (43.5) | 5274 (12.7) |
| 1 | **8.Ladakh** | Leh(Ladakh) | 66 (50.4) | 49 (75.5) | 36 (54.3) | 8 (12.4) |
| 2 |  | Kargil | 65 (49.6) | 44 (69.0) | 28 (43.3) | 16 (23.9) |
|  |  | Overall Ladakh | 132 (100.0) | 93 (72.3) | 64 (48.8) | 24 (18.1) |

## **Table S2: District wise prevalence of Ever measured blood pressure (BP), Prehypertension and Raised blood pressure in the states of central region of India (%) (National Family Health Survey-5, India, 2019-2021)**

| Sl. NO | State name |  | A. | B | C | D |
| --- | --- | --- | --- | --- | --- | --- |
|  |  | **District name** | **Total population analysed*** | **Ever measured blood pressure among the population (B/A)** | **Prevalence of prehypertension in the population(C/A)** | **Prevalence of raised BP in the population(D/A)** |
|  |  |  | **n(%)** | **n(%)** | **n(%)** | **n(%)** |
| Overall Indian population | | | **743067 (100.0)** | **472130 (66.7)** | **250624 (33.7)** | **118231 (15.9)** |
| Overall central region | | | **174729 (23.5)** | **105038 (63.7)** | **62200 (35.6)** | **28912 (16.5)** |
| 1 | **1.Uttarakhand** | Uttarkashi | 161 (2.8) | 99 (63.9) | 68 (42.4) | 20 (12.4) |
| 2 |  | Chamoli | 220 (3.8) | 140 (66.7) | 89 (40.3) | 40 (18.2) |
| 3 |  | Rudraprayag | 132 (2.3) | 83 (64.2) | 53 (40.1) | 15 (11.2) |
| 4 |  | Tehri Garhwal | 268 (4.6) | 173 (66.3) | 99 (36.8) | 34 (12.7) |
| 5 |  | Dehradun | 1022 (17.7) | 708 (81.5) | 330 (32.2) | 155 (15.2) |
| 6 |  | Garhwal | 298 (5.2) | 185 (64.0) | 118 (39.5) | 45 (15.2) |
| 7 |  | Pithoragarh | 301 (5.2) | 193 (65.5) | 135 (44.9) | 59 (19.5) |
| 8 |  | Bageshwar | 140 (2.4) | 88 (64.1) | 56 (39.9) | 19 (13.9) |
| 9 |  | Almora | 280 (4.9) | 204 (73.5) | 107 (38.3) | 48 (17.1) |
| 10 |  | Champawat | 143 (2.5) | 91 (65.2) | 56 (38.9) | 23 (15.9) |
| 11 |  | Nainital | 549 (9.5) | 425 (80.0) | 217 (39.6) | 97 (17.7) |
| 12 |  | Udham Singh Nagar | 1133 (19.6) | 817 (78.1) | 361 (31.9) | 213 (18.8) |
| 13 |  | Hardwar | 1124 (19.5) | 747 (75.0) | 347 (30.8) | 237 (21.1) |
|  |  | **Overall Uttarakhand** | 5771 (100.0) | 3954 (74.1) | 2035 (35.3) | 1006 (17.4) |
| 1 | **2.Uttar Pradesh** | Saharanpur | 2072 (1.8) | 1442 (70.2) | 1059 (51.1) | 346 (16.7) |
| 2 |  | Bijnor | 2347 (2.1) | 1788 (79.1) | 906 (38.6) | 397 (16.9) |
| 3 |  | Rampur | 1195 (1.1) | 720 (63.6) | 381 (31.8) | 158 (13.2) |
| 4 |  | Jyotiba Phule Nagar | 1065 (0.9) | 834 (78.6) | 501 (47.0) | 212 (19.9) |
| 5 |  | Meerut | 2158 (1.9) | 1570 (81.2) | 659 (30.5) | 327 (15.1) |
| 6 |  | Baghpat | 717 (0.6) | 515 (77.1) | 223 (31.1) | 142 (19.8) |
| 7 |  | Gautam Buddha Nagar | 993 (0.9) | 622 (82.0) | 250 (25.2) | 111 (11.2) |
| 8 |  | Bulandshahr | 1774 (1.6) | 1399 (82.9) | 578 (32.6) | 296 (16.7) |
| 9 |  | Aligarh | 1928 (1.7) | 1333 (75.0) | 563 (29.2) | 303 (15.7) |
| 10 |  | Mahamaya Nagar | 839 (0.7) | 558 (68.0) | 255 (30.5) | 192 (22.9) |
| 11 |  | Mathura | 1376 (1.2) | 909 (69.8) | 469 (34.1) | 260 (18.9) |
| 12 |  | Agra | 2891 (2.6) | 1878 (66.0) | 1088 (37.6) | 529 (18.3) |
| 13 |  | Firozabad | 1337 (1.2) | 915 (70.3) | 515 (38.5) | 166 (12.4) |
| 14 |  | Mainpuri | 946 (0.8) | 617 (65.6) | 333 (35.3) | 109 (11.5) |
| 15 |  | Bareilly | 2393 (2.1) | 1488 (73.9) | 677 (28.3) | 353 (14.7) |
| 16 |  | Pilibhit | 1033 (0.9) | 720 (75.5) | 343 (33.2) | 172 (16.7) |
| 17 |  | Shahjahanpur | 1754 (1.6) | 1057 (60.5) | 647 (36.9) | 294 (16.8) |
| 18 |  | Kheri | 2512 (2.2) | 1560 (65.1) | 1028 (40.9) | 436 (17.4) |
| 19 |  | Sitapur | 2545 (2.3) | 1506 (62.7) | 917 (36.0) | 352 (13.8) |
| 20 |  | Hardoi | 2202 (2.0) | 1142 (53.0) | 844 (38.4) | 328 (14.9) |
| 21 |  | Unnao | 1646 (1.5) | 910 (57.5) | 621 (37.7) | 328 (19.9) |
| 22 |  | Lucknow | 2700 (2.4) | 1739 (75.8) | 816 (30.2) | 436 (16.1) |
| 23 |  | Farrukhabad | 1046 (0.9) | 641 (61.7) | 386 (36.9) | 161 (15.4) |
| 24 |  | Kannauj | 872 (0.8) | 440 (51.7) | 293 (33.6) | 98 (11.3) |
| 25 |  | Etawah | 901 (0.8) | 604 (67.4) | 331 (36.8) | 145 (16.1) |
| 26 |  | Auraiya | 751 (0.7) | 463 (64.8) | 350 (46.6) | 126 (16.7) |
| 27 |  | Kanpur Dehat | 1010 (0.9) | 610 (64.2) | 366 (36.2) | 94 (9.3) |
| 28 |  | Kanpur Nagar | 2361 (2.1) | 1528 (69.0) | 921 (39.0) | 261 (11.1) |
| 29 |  | Jalaun | 991 (0.9) | 597 (61.0) | 423 (42.7) | 184 (18.5) |
| 30 |  | Jhansi | 1297 (1.2) | 767 (59.7) | 641 (49.4) | 169 (13.0) |
| 31 |  | Lalitpur | 818 (0.7) | 438 (54.4) | 340 (41.6) | 158 (19.3) |
| 32 |  | Hamirpur | 689 (0.6) | 360 (52.9) | 364 (52.8) | 79 (11.5) |
| 33 |  | Mahoba | 513 (0.5) | 266 (53.0) | 241 (47.0) | 64 (12.4) |
| 34 |  | Banda | 943 (0.8) | 477 (51.5) | 376 (39.9) | 154 (16.3) |
| 35 |  | Chitrakoot | 486 (0.4) | 228 (47.9) | 171 (35.3) | 71 (14.7) |
| 36 |  | Fatehpur | 1374 (1.2) | 710 (53.2) | 484 (35.3) | 189 (13.8) |
| 37 |  | Pratapgarh | 2015 (1.8) | 1106 (58.9) | 508 (25.2) | 371 (18.4) |
| 38 |  | Kaushambi | 790 (0.7) | 434 (62.3) | 191 (24.2) | 40 (5.1) |
| 39 |  | Allahabad | 3296 (2.9) | 1803 (61.2) | 1030 (31.3) | 326 (9.9) |
| 40 |  | Bara Banki | 1669 (1.5) | 820 (52.9) | 558 (33.4) | 275 (16.5) |
| 41 |  | Faizabad | 1516 (1.4) | 862 (62.3) | 412 (27.2) | 200 (13.2) |
| 42 |  | Ambedkar Nagar | 1254 (1.1) | 716 (62.2) | 351 (28.0) | 157 (12.5) |
| 43 |  | Bahraich | 1757 (1.6) | 691 (43.4) | 541 (30.8) | 411 (23.4) |
| 44 |  | Shrawasti | 632 (0.6) | 247 (41.8) | 208 (32.9) | 146 (23.1) |
| 45 |  | Balrampur | 1021 (0.9) | 560 (56.0) | 357 (35.0) | 258 (25.3) |
| 46 |  | Gonda | 1706 (1.5) | 811 (50.0) | 460 (26.9) | 515 (30.2) |
| 47 |  | Siddharthnagar | 1310 (1.2) | 782 (60.2) | 480 (36.7) | 276 (21.1) |
| 48 |  | Basti | 1336 (1.2) | 721 (54.5) | 383 (28.7) | 365 (27.3) |
| 49 |  | Sant Kabir Nagar | 840 (0.7) | 330 (41.1) | 293 (34.9) | 198 (23.6) |
| 50 |  | Mahrajganj | 1459 (1.3) | 772 (55.7) | 496 (34.0) | 297 (20.3) |
| 51 |  | Gorakhpur | 2614 (2.3) | 1643 (66.1) | 908 (34.7) | 526 (20.1) |
| 52 |  | Kushinagar | 1935 (1.7) | 1165 (64.1) | 518 (26.8) | 427 (22.1) |
| 53 |  | Deoria | 1678 (1.5) | 943 (60.4) | 523 (31.2) | 322 (19.2) |
| 54 |  | Azamgarh | 2717 (2.4) | 1569 (64.9) | 740 (27.2) | 407 (15.0) |
| 55 |  | Mau | 1159 (1.0) | 595 (51.8) | 486 (41.9) | 263 (22.7) |
| 56 |  | Ballia | 1563 (1.4) | 826 (53.3) | 539 (34.5) | 436 (27.9) |
| 57 |  | Jaunpur | 2810 (2.5) | 1493 (53.8) | 908 (32.3) | 628 (22.3) |
| 58 |  | Ghazipur | 1926 (1.7) | 1197 (62.5) | 818 (42.5) | 387 (20.1) |
| 59 |  | Chandauli | 1182 (1.1) | 733 (66.7) | 387 (32.8) | 212 (18.0) |
| 60 |  | Varanasi | 2250 (2.0) | 1255 (56.6) | 831 (36.9) | 506 (22.5) |
| 61 |  | Sant Ravidas Nagar (Bhadohi) | 1024 (0.9) | 583 (61.1) | 305 (29.8) | 154 (15.0) |
| 62 |  | Mirzapur | 1302 (1.2) | 693 (59.4) | 374 (28.7) | 339 (26.0) |
| 63 |  | Sonbhadra | 1040 (0.9) | 501 (50.5) | 416 (40.0) | 199 (19.1) |
| 64 |  | Etah | 965 (0.9) | 630 (65.6) | 377 (39.0) | 131 (13.5) |
| 65 |  | Kanshiram Nagar | 689 (0.6) | 383 (56.8) | 228 (33.1) | 100 (14.5) |
| 66 |  | Amethi | 1106 (1.0) | 706 (68.2) | 380 (34.4) | 156 (14.1) |
| 67 |  | Budaun | 1570 (1.4) | 1054 (68.3) | 607 (38.7) | 163 (10.4) |
| 68 |  | Ghaziabad | 2645 (2.4) | 1796 (84.6) | 698 (26.4) | 358 (13.5) |
| 69 |  | Hapur | 808 (0.7) | 677 (84.3) | 385 (47.6) | 164 (20.3) |
| 70 |  | Moradabad | 1756 (1.6) | 1134 (65.5) | 858 (48.9) | 299 (17.0) |
| 71 |  | Muzaffarnagar | 1568 (1.4) | 1116 (76.6) | 642 (40.9) | 268 (17.1) |
| 72 |  | Rae Bareli | 1536 (1.4) | 857 (57.8) | 576 (37.5) | 247 (16.1) |
| 73 |  | Sambhal | 1217 (1.1) | 944 (78.6) | 591 (48.6) | 187 (15.4) |
| 74 |  | Shamli | 740 (0.7) | 513 (71.1) | 382 (51.6) | 116 (15.6) |
| 75 |  | Sultanpur | 1307 (1.2) | 743 (62.6) | 362 (27.7) | 220 (16.8) |
|  |  | **Overall Uttar Pradesh** | 112176 (100.0) | 67755 (63.9) | 39468 (35.2) | 19247 (17.2) |
| 1 | **3.Chhattisgarh** | Koriya | 390 (2.4) | 202 (53.6) | 135 (34.5) | 59 (15.1) |
| 2 |  | Jashpur | 497 (3.0) | 318 (65.2) | 193 (38.9) | 97 (19.4) |
| 3 |  | Raigarh | 1718 (10.4) | 1225 (72.1) | 686 (39.9) | 338 (19.7) |
| 4 |  | Korba | 738 (4.5) | 395 (58.1) | 246 (33.3) | 163 (22.0) |
| 5 |  | Janjgir - Champa | 998 (6.0) | 548 (57.5) | 349 (35.0) | 174 (17.4) |
| 6 |  | Kabeerdham | 564 (3.4) | 363 (65.0) | 249 (44.1) | 86 (15.2) |
| 7 |  | Rajnandgaon | 914 (5.5) | 623 (69.2) | 358 (39.2) | 171 (18.7) |
| 8 |  | Mahasamund | 645 (3.9) | 361 (57.5) | 261 (40.4) | 102 (15.9) |
| 9 |  | Dhamtari | 513 (3.1) | 306 (61.0) | 187 (36.3) | 86 (16.8) |
| 10 |  | Uttar Bastar Kanker | 420 (2.5) | 210 (50.4) | 194 (46.2) | 77 (18.4) |
| 11 |  | Narayanpur | 83 (0.5) | 31 (38.2) | 36 (43.7) | 12 (14.6) |
| 12 |  | Bijapur | 153 (0.9) | 65 (43.6) | 59 (38.7) | 17 (11.3) |
| 13 |  | Balod | 481 (2.9) | 253 (53.0) | 224 (46.6) | 86 (17.9) |
| 14 |  | Baloda Bazar | 804 (4.9) | 502 (64.8) | 309 (38.4) | 134 (16.7) |
| 15 |  | Balrampur | 462 (2.8) | 234 (52.5) | 169 (36.5) | 77 (16.7) |
| 16 |  | Bastar | 479 (2.9) | 176 (39.1) | 108 (22.6) | 100 (20.8) |
| 17 |  | Bemetara | 508 (3.1) | 363 (71.8) | 213 (41.9) | 92 (18.2) |
| 18 |  | Bilaspur | 1231 (7.5) | 724 (63.1) | 429 (34.8) | 224 (18.2) |
| 19 |  | Dantewada | 182 (1.1) | 90 (50.5) | 79 (43.5) | 30 (16.8) |
| 20 |  | Durg | 1103 (6.7) | 858 (78.7) | 494 (44.7) | 182 (16.5) |
| 21 |  | Gariyaband | 383 (2.3) | 194 (51.9) | 157 (41.0) | 65 (16.9) |
| 22 |  | Kodagaon | 343 (2.1) | 123 (36.4) | 103 (30.2) | 72 (21.1) |
| 23 |  | Mungeli | 398 (2.4) | 255 (67.5) | 162 (40.7) | 66 (16.7) |
| 24 |  | Raipur | 1495 (9.1) | 998 (67.8) | 599 (40.0) | 228 (15.2) |
| 25 |  | Sukma | 145 (0.9) | 61 (42.8) | 68 (46.7) | 19 (13.4) |
| 26 |  | Surajpur | 339 (2.1) | 206 (62.4) | 134 (39.6) | 59 (17.4) |
| 27 |  | Surguja | 511 (3.1) | 292 (59.7) | 194 (37.9) | 89 (17.4) |
|  |  | **Overall Chhattisgarh** | 16496 (100.0) | 9977 (62.3) | 6393 (38.8) | 2906 (17.6) |
| 1 | **4.Madhya Pradesh** | Sheopur | 423 (1.0) | 254 (61.9) | 150 (35.4) | 57 (13.4) |
| 2 |  | Morena | 927 (2.3) | 591 (68.3) | 296 (32.0) | 118 (12.7) |
| 3 |  | Bhind | 778 (1.9) | 482 (66.2) | 255 (32.8) | 99 (12.8) |
| 4 |  | Gwalior | 1235 (3.1) | 749 (70.2) | 397 (32.2) | 179 (14.5) |
| 5 |  | Datia | 436 (1.1) | 247 (59.7) | 130 (29.9) | 40 (9.3) |
| 6 |  | Shivpuri | 843 (2.1) | 482 (61.3) | 261 (30.9) | 79 (9.4) |
| 7 |  | Tikamgarh | 747 (1.9) | 445 (72.8) | 212 (28.3) | 43 (5.8) |
| 8 |  | Chhatarpur | 942 (2.3) | 520 (58.5) | 294 (31.2) | 107 (11.3) |
| 9 |  | Panna | 575 (1.4) | 329 (59.3) | 206 (35.9) | 72 (12.6) |
| 10 |  | Sagar | 1487 (3.7) | 854 (61.0) | 553 (37.2) | 256 (17.2) |
| 11 |  | Damoh | 760 (1.9) | 492 (68.8) | 253 (33.3) | 149 (19.6) |
| 12 |  | Satna | 1272 (3.2) | 901 (73.8) | 498 (39.2) | 125 (9.8) |
| 13 |  | Rewa | 1356 (3.4) | 792 (60.0) | 545 (40.2) | 181 (13.3) |
| 14 |  | Umaria | 436 (1.1) | 289 (68.2) | 203 (46.5) | 68 (15.7) |
| 15 |  | Neemuch | 538 (1.3) | 370 (70.3) | 243 (45.2) | 91 (16.9) |
| 16 |  | Mandsaur | 838 (2.1) | 555 (70.9) | 349 (41.6) | 157 (18.8) |
| 17 |  | Ratlam | 931 (2.3) | 526 (60.7) | 367 (39.4) | 133 (14.3) |
| 18 |  | Ujjain | 1201 (3.0) | 712 (61.1) | 497 (41.4) | 221 (18.4) |
| 19 |  | Dewas | 942 (2.3) | 566 (62.8) | 318 (33.7) | 216 (23.0) |
| 20 |  | Dhar | 1324 (3.3) | 744 (58.0) | 663 (50.0) | 171 (12.9) |
| 21 |  | Indore | 1952 (4.8) | 1300 (67.3) | 740 (37.9) | 273 (14.0) |
| 22 |  | Khargone (West Nimar) | 1019 (2.5) | 305 (32.2) | 449 (44.1) | 130 (12.7) |
| 23 |  | Barwani | 753 (1.9) | 410 (56.0) | 299 (39.8) | 122 (16.2) |
| 24 |  | Rajgarh | 902 (2.2) | 472 (53.7) | 308 (34.2) | 200 (22.2) |
| 25 |  | Vidisha | 810 (2.0) | 572 (74.1) | 232 (28.7) | 101 (12.5) |
| 26 |  | Bhopal | 1234 (3.1) | 629 (77.6) | 193 (15.6) | 145 (11.8) |
| 27 |  | Sehore | 760 (1.9) | 427 (58.5) | 231 (30.4) | 159 (21.0) |
| 28 |  | Raisen | 461 (1.1) | 153 (52.8) | 118 (25.7) | 38 (8.2) |
| 29 |  | Betul | 889 (2.2) | 572 (68.8) | 239 (26.9) | 135 (15.2) |
| 30 |  | Harda | 348 (0.9) | 184 (53.6) | 140 (40.3) | 52 (14.9) |
| 31 |  | Hoshangabad | 745 (1.8) | 370 (52.9) | 217 (29.2) | 148 (19.9) |
| 32 |  | Katni | 678 (1.7) | 371 (55.3) | 269 (39.6) | 83 (12.2) |
| 33 |  | Jabalpur | 654 (1.6) | 373 (66.0) | 161 (24.6) | 111 (16.9) |
| 34 |  | Narsimhapur | 729 (1.8) | 502 (71.0) | 245 (33.6) | 106 (14.5) |
| 35 |  | Dindori | 460 (1.1) | 307 (68.5) | 202 (43.8) | 74 (16.2) |
| 36 |  | Mandla | 609 (1.5) | 373 (64.2) | 210 (34.6) | 118 (19.3) |
| 37 |  | Chhindwara | 1126 (2.8) | 755 (72.1) | 316 (28.1) | 136 (12.1) |
| 38 |  | Seoni | 862 (2.1) | 530 (64.0) | 248 (28.8) | 160 (18.6) |
| 39 |  | Balaghat | 1022 (2.5) | 555 (57.9) | 408 (39.9) | 109 (10.7) |
| 40 |  | Guna | 754 (1.9) | 410 (54.9) | 339 (44.9) | 104 (13.8) |
| 41 |  | Ashoknagar | 461 (1.1) | 251 (59.1) | 143 (31.0) | 41 (8.8) |
| 42 |  | Shahdol | 644 (1.6) | 348 (55.8) | 223 (34.5) | 80 (12.5) |
| 43 |  | Anuppur | 468 (1.2) | 283 (62.7) | 170 (36.3) | 79 (16.8) |
| 44 |  | Sidhi | 660 (1.6) | 417 (64.7) | 278 (42.1) | 86 (13.0) |
| 45 |  | Singrauli | 583 (1.4) | 231 (47.4) | 207 (35.5) | 45 (7.7) |
| 46 |  | Jhabua | 504 (1.3) | 335 (66.9) | 213 (42.2) | 76 (15.0) |
| 47 |  | Alirajpur | 318 (0.8) | 107 (33.8) | 166 (52.3) | 45 (14.3) |
| 48 |  | Khandwa (East Nimar) | 649 (1.6) | 247 (55.7) | 185 (28.6) | 49 (7.5) |
| 49 |  | Burhanpur | 426 (1.1) | 268 (63.3) | 196 (45.9) | 64 (15.1) |
| 50 |  | Agar Malwa | 269 (0.7) | 87 (59.9) | 56 (20.7) | 17 (6.2) |
| 51 |  | Shajapur | 542 (1.3) | 310 (57.6) | 212 (39.2) | 104 (19.1) |
|  |  | Overall Madhya Pradesh | 40286 (100.0) | 23353 (62.4) | 14304 (35.5) | 5753 (14.3) |

## **Table S3: District wise prevalence of Ever measured blood pressure (BP), Prehypertension, and Raised blood pressure in the states of Eastern region of India (%) (National Family Health Survey-5, India, 2019-2021)**

| Sl. NO | State name | District name | A.  Total population analysed*  n(%) | B  Ever measured blood pressure among the population (B/A)  n(%) | | C  Prevalence of prehypertension in the population(C/A)  n(%) | D  Prevalence of raised BP in the population(D/A)  n(%) |
| --- | --- | --- | --- | --- | --- | --- | --- |
| Overall Indian population | | | 743067 (100.0) | | 472130 (66.7) | 250624 (33.7) | 118231 (15.9) |
| Overalll eastern region | | | 169650 (22.8) | | 102050 (62.2) | 52741 (31.1) | 26495 (15.6) |
| 1 | **1.Bihar** | Pashchim Champaran | 2334 (3.7) | 1369 (62.6) | | 752 (32.2) | 399 (17.1) |
| 2 |  | Purba Champaran | 3227 (5.2) | 2075 (67.1) | | 827 (25.6) | 670 (20.8) |
| 3 |  | Sheohar | 401 (0.6) | 227 (58.9) | | 182 (45.3) | 50 (12.3) |
| 4 |  | Sitamarhi | 2169 (3.5) | 1178 (57.7) | | 599 (27.6) | 383 (17.7) |
| 5 |  | Madhubani | 2403 (3.8) | 1256 (55.7) | | 618 (25.7) | 381 (15.9) |
| 6 |  | Supaul | 1425 (2.3) | 796 (57.2) | | 348 (24.4) | 235 (16.5) |
| 7 |  | Araria | 1841 (2.9) | 1183 (66.2) | | 393 (21.4) | 258 (14.0) |
| 8 |  | Kishanganj | 1077 (1.7) | 469 (47.1) | | 219 (20.4) | 194 (18.0) |
| 9 |  | Purnia | 2069 (3.3) | 997 (51.0) | | 338 (16.3) | 394 (19.0) |
| 10 |  | Katihar | 1735 (2.8) | 885 (54.7) | | 295 (17.0) | 298 (17.1) |
| 11 |  | Madhepura | 1141 (1.8) | 549 (48.9) | | 230 (20.1) | 151 (13.2) |
| 12 |  | Saharsa | 1114 (1.8) | 487 (45.3) | | 240 (21.5) | 200 (17.9) |
| 13 |  | Darbhanga | 2470 (4.0) | 1422 (59.6) | | 608 (24.6) | 487 (19.7) |
| 14 |  | Muzaffarpur | 3167 (5.1) | 1859 (63.2) | | 841 (26.5) | 580 (18.3) |
| 15 |  | Gopalganj | 1621 (2.6) | 957 (61.2) | | 537 (33.1) | 356 (22.0) |
| 16 |  | Siwan | 1897 (3.0) | 1290 (72.4) | | 616 (32.5) | 339 (17.8) |
| 17 |  | Saran | 2319 (3.7) | 1348 (62.3) | | 554 (23.9) | 530 (22.9) |
| 18 |  | Vaishali | 1901 (3.0) | 1151 (63.5) | | 327 (17.2) | 530 (27.9) |
| 19 |  | Samastipur | 2543 (4.1) | 1316 (53.2) | | 551 (21.7) | 436 (17.1) |
| 20 |  | Begusarai | 2241 (3.6) | 1558 (71.6) | | 394 (17.6) | 594 (26.5) |
| 21 |  | Khagaria | 1054 (1.7) | 594 (59.1) | | 218 (20.7) | 128 (12.2) |
| 22 |  | Bhagalpur | 2210 (3.5) | 1176 (57.4) | | 584 (26.4) | 301 (13.6) |
| 23 |  | Banka | 1089 (1.7) | 663 (61.6) | | 356 (32.7) | 203 (18.7) |
| 24 |  | Munger | 898 (1.4) | 631 (73.0) | | 246 (27.3) | 137 (15.2) |
| 25 |  | Lakhisarai | 596 (1.0) | 381 (65.8) | | 142 (23.8) | 105 (17.5) |
| 26 |  | Sheikhpura | 351 (0.6) | 247 (71.3) | | 118 (33.5) | 78 (22.1) |
| 27 |  | Nalanda | 1651 (2.6) | 1154 (70.5) | | 600 (36.4) | 274 (16.6) |
| 28 |  | Patna | 3232 (5.2) | 1903 (61.2) | | 779 (24.1) | 485 (15.0) |
| 29 |  | Bhojpur | 1799 (2.9) | 1311 (73.9) | | 467 (25.9) | 306 (17.0) |
| 30 |  | Buxar | 976 (1.6) | 637 (66.5) | | 277 (28.4) | 119 (12.2) |
| 31 |  | Kaimur (Bhabua) | 844 (1.4) | 590 (71.2) | | 366 (43.4) | 88 (10.4) |
| 32 |  | Rohtas | 1921 (3.1) | 1516 (79.6) | | 736 (38.3) | 254 (13.2) |
| 33 |  | Aurangabad | 1320 (2.1) | 951 (72.4) | | 496 (37.6) | 179 (13.6) |
| 34 |  | Gaya | 2227 (3.6) | 1370 (63.8) | | 780 (35.0) | 341 (15.3) |
| 35 |  | Nawada | 1241 (2.0) | 774 (63.9) | | 374 (30.1) | 234 (18.9) |
| 36 |  | Jamui | 949 (1.5) | 564 (60.9) | | 266 (28.1) | 155 (16.3) |
| 37 |  | Jehanabad | 583 (0.9) | 424 (73.9) | | 158 (27.1) | 67 (11.4) |
| 38 |  | Arwal | 396 (0.6) | 291 (74.6) | | 125 (31.6) | 50 (12.6) |
|  |  | **Overall Bihar** | 62431 (100.0) | 37546 (62.7) | | 16555 (26.5) | 10967 (17.6) |
| 1 | **2.West Bengal** | Darjiling | 1262 (1.9) | 933 (76.5) | | 466 (37.0) | 267 (21.1) |
| 2 |  | Jalpaiguri | 2887 (4.4) | 1765 (61.5) | | 1018 (35.3) | 423 (14.7) |
| 3 |  | Koch Bihar | 2220 (3.4) | 1439 (65.5) | | 686 (30.9) | 342 (15.4) |
| 4 |  | Uttar Dinajpur | 1937 (2.9) | 1401 (72.9) | | 815 (42.1) | 218 (11.2) |
| 5 |  | Dakshin Dinajpur | 1339 (2.0) | 927 (70.2) | | 447 (33.4) | 194 (14.5) |
| 6 |  | Maldah | 2833 (4.3) | 1515 (54.3) | | 701 (24.7) | 331 (11.7) |
| 7 |  | Murshidabad | 5389 (8.2) | 4010 (75.3) | | 1353 (25.1) | 671 (12.4) |
| 8 |  | Birbhum | 2776 (4.2) | 1889 (68.5) | | 929 (33.5) | 350 (12.6) |
| 9 |  | Nadia | 3760 (5.7) | 2609 (70.8) | | 1386 (36.9) | 583 (15.5) |
| 10 |  | North Twenty Four Parganas | 7264 (11.0) | 4984 (69.1) | | 3435 (47.3) | 931 (12.8) |
| 11 |  | Hugli | 4094 (6.2) | 2523 (64.5) | | 1092 (26.7) | 549 (13.4) |
| 12 |  | Bankura | 2547 (3.9) | 1058 (45.6) | | 543 (21.3) | 294 (11.5) |
| 13 |  | Puruliya | 1860 (2.8) | 744 (42.3) | | 630 (33.9) | 260 (14.0) |
| 14 |  | Haora | 3626 (5.5) | 2648 (73.9) | | 1352 (37.3) | 642 (17.7) |
| 15 |  | Kolkata | 3141 (4.8) | 2191 (72.4) | | 1255 (39.9) | 387 (12.3) |
| 16 |  | South Twenty Four Parganas | 5993 (9.1) | 3931 (67.4) | | 1276 (21.3) | 724 (12.1) |
| 17 |  | Paschim Medinipur | 4284 (6.5) | 2248 (56.0) | | 1369 (31.9) | 528 (12.3) |
| 18 |  | Purba Medinipur | 3275 (5.0) | 1602 (54.0) | | 753 (23.0) | 407 (12.4) |
| 19 |  | Paschim Barddhaman | 2095 (3.2) | 1250 (61.6) | | 673 (32.1) | 261 (12.5) |
| 20 |  | Purba Barddhaman | 3499 (5.3) | 1879 (56.0) | | 1193 (34.1) | 436 (12.5) |
|  |  | **Overall West Bengal** | 66082 (100.0) | 41549 (64.8) | | 21371 (32.3) | 8797 (13.3) |
| 1 | **3.Jharkhand** | Garhwa | 679 (3.9) | 448 (67.2) | | 233 (34.3) | 88 (12.9) |
| 2 |  | Chatra | 551 (3.1) | 329 (61.8) | | 194 (35.3) | 65 (11.7) |
| 3 |  | Kodarma | 373 (2.1) | 245 (71.0) | | 105 (28.2) | 36 (9.6) |
| 4 |  | Giridih | 1139 (6.5) | 647 (58.2) | | 344 (30.2) | 184 (16.2) |
| 5 |  | Deoghar | 774 (4.4) | 382 (51.0) | | 257 (33.3) | 86 (11.1) |
| 6 |  | Godda | 653 (3.7) | 300 (49.0) | | 244 (37.4) | 76 (11.6) |
| 7 |  | Sahibganj | 616 (3.5) | 341 (59.9) | | 241 (39.1) | 62 (10.0) |
| 8 |  | Pakur | 494 (2.8) | 271 (57.5) | | 163 (33.0) | 49 (9.9) |
| 9 |  | Dhanbad | 1583 (9.0) | 936 (62.3) | | 495 (31.2) | 289 (18.2) |
| 10 |  | Bokaro | 1149 (6.5) | 650 (57.9) | | 409 (35.6) | 248 (21.6) |
| 11 |  | Lohardaga | 225 (1.3) | 149 (66.5) | | 92 (40.8) | 36 (16.1) |
| 12 |  | Purbi Singhbhum | 1256 (7.2) | 872 (69.5) | | 610 (48.6) | 175 (13.9) |
| 13 |  | Palamu | 1083 (6.2) | 740 (69.4) | | 384 (35.4) | 167 (15.4) |
| 14 |  | Latehar | 385 (2.2) | 200 (52.8) | | 174 (45.2) | 53 (13.8) |
| 15 |  | Hazaribagh | 989 (5.6) | 509 (56.8) | | 315 (31.9) | 136 (13.7) |
| 16 |  | Ramgarh | 502 (2.9) | 287 (60.7) | | 151 (30.1) | 108 (21.6) |
| 17 |  | Dumka | 686 (3.9) | 392 (58.8) | | 224 (32.6) | 115 (16.7) |
| 18 |  | Jamtara | 428 (2.4) | 200 (50.6) | | 150 (35.0) | 65 (15.2) |
| 19 |  | Ranchi | 1501 (8.6) | 887 (59.4) | | 692 (46.1) | 232 (15.4) |
| 20 |  | Khunti | 282 (1.6) | 155 (55.5) | | 144 (51.1) | 35 (12.4) |
| 21 |  | Gumla | 532 (3.0) | 307 (58.2) | | 276 (51.8) | 71 (13.3) |
| 22 |  | Simdega | 310 (1.8) | 170 (56.2) | | 150 (48.4) | 57 (18.3) |
| 23 |  | Pashchimi Singhbhum | 752 (4.3) | 357 (47.9) | | 336 (44.7) | 137 (18.2) |
| 24 |  | Saraikela-Kharsawan | 611 (3.5) | 379 (62.6) | | 307 (50.2) | 96 (15.7) |
|  |  | **Overall Jharkhand** | 17553 (100.0) | 10154 (59.8) | | 6691 (38.1) | 2664 (15.2) |
| 1 | **4.Odisha** | Bargarh | 847 (3.6) | 357 (42.9) | | 312 (36.8) | 145 (17.1) |
| 2 |  | Jharsuguda | 348 (1.5) | 241 (71.2) | | 134 (38.5) | 47 (13.4) |
| 3 |  | Sambalpur | 559 (2.4) | 350 (63.5) | | 271 (48.5) | 88 (15.7) |
| 4 |  | Debagarh | 162 (0.7) | 87 (54.9) | | 56 (34.6) | 28 (17.1) |
| 5 |  | Sundargarh | 1204 (5.1) | 633 (53.8) | | 459 (38.1) | 199 (16.6) |
| 6 |  | Kendujhar | 1119 (4.7) | 466 (42.5) | | 385 (34.4) | 288 (25.7) |
| 7 |  | Mayurbhanj | 1421 (6.0) | 560 (40.0) | | 524 (36.9) | 321 (22.6) |
| 8 |  | Baleshwar | 1243 (5.3) | 575 (47.7) | | 392 (31.5) | 240 (19.3) |
| 9 |  | Bhadrak | 804 (3.4) | 430 (56.3) | | 230 (28.6) | 126 (15.7) |
| 10 |  | Kendrapara | 789 (3.3) | 424 (55.4) | | 219 (27.8) | 130 (16.4) |
| 11 |  | Jagatsinghapur | 586 (2.5) | 330 (57.5) | | 167 (28.5) | 141 (24.1) |
| 12 |  | Cuttack | 1425 (6.0) | 843 (61.1) | | 423 (29.7) | 279 (19.6) |
| 13 |  | Jajapur | 1043 (4.4) | 530 (52.1) | | 317 (30.4) | 197 (18.9) |
| 14 |  | Dhenkanal | 641 (2.7) | 341 (54.7) | | 200 (31.2) | 113 (17.6) |
| 15 |  | Anugul | 713 (3.0) | 399 (56.6) | | 213 (29.8) | 117 (16.4) |
| 16 |  | Nayagarh | 547 (2.3) | 366 (67.7) | | 161 (29.4) | 65 (11.8) |
| 17 |  | Khordha | 1332 (5.6) | 806 (65.0) | | 379 (28.5) | 178 (13.4) |
| 18 |  | Puri | 954 (4.0) | 555 (59.7) | | 286 (30.0) | 137 (14.4) |
| 19 |  | Ganjam | 1949 (8.3) | 1078 (57.1) | | 575 (29.5) | 308 (15.8) |
| 20 |  | Gajapati | 308 (1.3) | 189 (62.6) | | 126 (40.9) | 44 (14.2) |
| 21 |  | Kandhamal | 449 (1.9) | 251 (55.9) | | 177 (39.4) | 84 (18.7) |
| 22 |  | Baudh | 270 (1.1) | 128 (48.2) | | 97 (35.8) | 39 (14.6) |
| 23 |  | Subarnapur | 356 (1.5) | 239 (67.7) | | 162 (45.4) | 56 (15.8) |
| 24 |  | Balangir | 1035 (4.4) | 711 (69.2) | | 470 (45.4) | 150 (14.5) |
| 25 |  | Nuapada | 348 (1.5) | 146 (42.1) | | 133 (38.3) | 52 (14.9) |
| 26 |  | Kalahandi | 710 (3.0) | 413 (58.3) | | 287 (40.5) | 136 (19.1) |
| 27 |  | Rayagada | 549 (2.3) | 307 (57.0) | | 190 (34.6) | 67 (12.3) |
| 28 |  | Nabarangapur | 712 (3.0) | 504 (71.2) | | 342 (48.0) | 119 (16.7) |
| 29 |  | Koraput | 797 (3.4) | 343 (43.2) | | 321 (40.3) | 121 (15.2) |
| 30 |  | Malkangiri | 365 (1.5) | 198 (54.3) | | 115 (31.6) | 52 (14.2) |
|  |  | **Overall Odisha** | 23584 (100.0) | 12799 (55.5) | | 8124 (34.4) | 4067 (17.2) |

## **Table S4: District wise prevalence of Ever measured blood pressure (BP), Prehypertension, and Raised blood pressure in the states of western region of India (%) (National Family Health Survey-5, India, 2019-2021)**

| SL.NO | State name | District name | A. | B | C | D |
| --- | --- | --- | --- | --- | --- | --- |
|  |  |  | **Total population analysed*** | **Ever measured blood pressure among the population (B/A)** | **Prevalence of prehypertension in the population(C/A)** | **Prevalence of raised BP in the population(D/A)** |
|  |  |  | **n(%)** | **n(%)** | **n(%)** | **n(%)** |
| **Overall Indian population** | | | 743067 (100.0) | 472130 (66.7) | 250624 (33.7) | 118231 (15.9) |
| **Overall western region** | | | 115649 (15.6) | 68148 (62.5) | 40108 (34.7) | 15628 (13.5) |
| 1 | **1.Gujarat** | Kachchh | 1288 (3.4) | 858 (67.6) | 512 (39.8) | 141 (11.0) |
| 2 |  | Banas Kantha | 1763 (4.7) | 780 (46.4) | 496 (28.1) | 171 (9.7) |
| 3 |  | Patan | 813 (2.2) | 405 (50.1) | 272 (33.5) | 78 (9.6) |
| 4 |  | Mahesana | 1364 (3.6) | 787 (59.4) | 378 (27.7) | 219 (16.0) |
| 5 |  | Gandhinagar | 1004 (2.7) | 477 (50.5) | 319 (31.8) | 157 (15.6) |
| 6 |  | Porbandar | 383 (1.0) | 234 (62.1) | 157 (41.0) | 49 (12.9) |
| 7 |  | Amreli | 866 (2.3) | 377 (45.5) | 330 (38.1) | 71 (8.2) |
| 8 |  | Anand | 1274 (3.4) | 761 (60.5) | 464 (36.4) | 222 (17.4) |
| 9 |  | Dohad | 1272 (3.4) | 526 (42.0) | 436 (34.3) | 183 (14.4) |
| 10 |  | Narmada | 383 (1.0) | 174 (45.5) | 155 (40.4) | 51 (13.3) |
| 11 |  | Bharuch | 956 (2.5) | 395 (43.8) | 271 (28.4) | 169 (17.7) |
| 12 |  | The Dangs | 130 (0.3) | 72 (55.2) | 55 (42.5) | 18 (14.0) |
| 13 |  | Navsari | 897 (2.4) | 657 (73.9) | 392 (43.7) | 128 (14.3) |
| 14 |  | Valsad | 1253 (3.3) | 722 (57.8) | 502 (40.0) | 231 (18.5) |
| 15 |  | Surat | 4128 (11.0) | 2571 (62.7) | 1676 (40.6) | 593 (14.4) |
| 16 |  | Tapi | 523 (1.4) | 337 (65.2) | 170 (32.5) | 80 (15.2) |
| 17 |  | Ahmadabad | 4190 (11.2) | 2713 (66.2) | 1460 (34.8) | 410 (9.8) |
| 18 |  | Aravali | 715 (1.9) | 354 (49.7) | 184 (25.7) | 92 (12.8) |
| 19 |  | Bhavnagar | 1340 (3.6) | 920 (72.0) | 410 (30.6) | 182 (13.5) |
| 20 |  | Botad | 419 (1.1) | 164 (40.9) | 131 (31.3) | 29 (6.9) |
| 21 |  | Chhota Udaipur | 593 (1.6) | 353 (60.0) | 251 (42.4) | 61 (10.4) |
| 22 |  | Devbhumi Dwarka | 485 (1.3) | 206 (43.8) | 165 (34.0) | 74 (15.2) |
| 23 |  | Gir Somnath | 731 (1.9) | 465 (65.1) | 257 (35.2) | 71 (9.8) |
| 24 |  | Jamnagar | 849 (2.3) | 530 (62.6) | 370 (43.5) | 57 (6.8) |
| 25 |  | Junagadh | 942 (2.5) | 400 (43.4) | 340 (36.0) | 121 (12.9) |
| 26 |  | Kheda | 1367 (3.6) | 515 (39.4) | 489 (35.8) | 214 (15.6) |
| 27 |  | Mahisagar | 615 (1.6) | 297 (48.9) | 224 (36.4) | 63 (10.2) |
| 28 |  | Morbi | 616 (1.6) | 357 (58.7) | 222 (36.0) | 63 (10.2) |
| 29 |  | Panch Mahals | 1172 (3.1) | 758 (64.9) | 410 (35.0) | 170 (14.5) |
| 30 |  | Rajkot | 1758 (4.7) | 1152 (68.1) | 623 (35.4) | 180 (10.3) |
| 31 |  | Sabar Kantha | 861 (2.3) | 408 (48.1) | 336 (39.0) | 139 (16.2) |
| 32 |  | Surendranagar | 888 (2.4) | 397 (45.5) | 285 (32.1) | 75 (8.4) |
| 33 |  | Vadodara | 1694 (4.5) | 1176 (70.5) | 576 (34.0) | 278 (16.4) |
|  |  | **Overall Gujarat** | 37534 (100.0) | 21295 (58.0) | 13320 (35.5) | 4841 (12.9) |
| 1 | **2.Dadra & Nagar Haveli And Daman & Diu** | Diu | 21 (6.6) | 15 (72.1) | 9 (41.9) | 2 (9.0) |
| 2 |  | Daman | 76 (24.3) | 43 (60.2) | 27 (36.0) | 11 (14.2) |
| 3 |  | Dadra & Nagar Haveli | 217 (69.2) | 157 (74.7) | 80 (36.9) | 19 (8.8) |
| 4 |  | **Overall Dadra & Nagar Haveli And Daman & Diu** | 313 (100.0) | 215 (71.1) | 116 (37.0) | 32 (10.1) |
| 1 | **3.Maharashtra** | Nandurbar | 1095 (1.4) | 440 (41.6) | 460 (42.0) | 160 (14.6) |
| 2 |  | Dhule | 1238 (1.6) | 501 (42.5) | 369 (29.8) | 165 (13.3) |
| 3 |  | Jalgaon | 2496 (3.3) | 990 (41.9) | 818 (32.8) | 334 (13.4) |
| 4 |  | Buldana | 1760 (2.3) | 1183 (68.3) | 737 (41.9) | 248 (14.1) |
| 5 |  | Akola | 1364 (1.8) | 878 (64.9) | 573 (42.0) | 151 (11.1) |
| 6 |  | Washim | 781 (1.0) | 456 (59.4) | 291 (37.2) | 114 (14.7) |
| 7 |  | Amravati | 2018 (2.6) | 1103 (55.1) | 775 (38.4) | 233 (11.6) |
| 8 |  | Wardha | 854 (1.1) | 545 (64.0) | 322 (37.7) | 85 (10.0) |
| 9 |  | Nagpur | 2937 (3.8) | 2152 (74.0) | 1211 (41.2) | 424 (14.4) |
| 10 |  | Bhandara | 782 (1.0) | 552 (70.7) | 315 (40.3) | 97 (12.4) |
| 11 |  | Gondiya | 819 (1.1) | 535 (65.5) | 345 (42.2) | 98 (12.0) |
| 12 |  | Gadchiroli | 659 (0.9) | 436 (66.3) | 275 (41.7) | 68 (10.4) |
| 13 |  | Chandrapur | 1505 (2.0) | 1212 (80.8) | 660 (43.8) | 185 (12.3) |
| 14 |  | Yavatmal | 1857 (2.4) | 1179 (64.4) | 661 (35.6) | 212 (11.4) |
| 15 |  | Nanded | 1985 (2.6) | 1206 (63.5) | 627 (31.6) | 238 (12.0) |
| 16 |  | Hingoli | 770 (1.0) | 370 (49.3) | 244 (31.7) | 81 (10.5) |
| 17 |  | Parbhani | 1134 (1.5) | 480 (49.4) | 274 (24.2) | 127 (11.2) |
| 18 |  | Jalna | 1330 (1.7) | 622 (53.7) | 341 (25.6) | 178 (13.4) |
| 19 |  | Aurangabad | 2405 (3.1) | 1372 (63.2) | 601 (25.0) | 293 (12.2) |
| 20 |  | Nashik | 4003 (5.2) | 1766 (46.0) | 1321 (33.0) | 609 (15.2) |
| 21 |  | Mumbai Suburban | 6425 (8.4) | 3199 (76.5) | 1682 (26.2) | 631 (9.8) |
| 22 |  | Mumbai | 2250 (2.9) | 1670 (78.6) | 956 (42.5) | 196 (8.7) |
| 23 |  | Raigarh | 1922 (2.5) | 1328 (70.1) | 654 (34.0) | 324 (16.8) |
| 24 |  | Pune | 8571 (11.2) | 5278 (66.3) | 2502 (29.2) | 1401 (16.3) |
| 25 |  | Ahmadnagar | 2979 (3.9) | 1747 (60.2) | 1061 (35.6) | 459 (15.4) |
| 26 |  | Bid | 1373 (1.8) | 768 (59.3) | 476 (34.7) | 170 (12.4) |
| 27 |  | Latur | 1346 (1.8) | 978 (73.7) | 519 (38.6) | 154 (11.5) |
| 28 |  | Osmanabad | 892 (1.2) | 620 (69.8) | 350 (39.2) | 128 (14.3) |
| 29 |  | Solapur | 2800 (3.7) | 1840 (66.3) | 1171 (41.8) | 459 (16.4) |
| 30 |  | Satara | 1963 (2.6) | 1402 (71.8) | 741 (37.7) | 343 (17.5) |
| 31 |  | Ratnagiri | 1045 (1.4) | 559 (57.4) | 368 (35.2) | 181 (17.3) |
| 32 |  | Sindhudurg | 487 (0.6) | 287 (61.0) | 183 (37.6) | 94 (19.3) |
| 33 |  | Kolhapur | 2733 (3.6) | 1806 (68.6) | 1043 (38.2) | 578 (21.1) |
| 34 |  | Sangli | 1883 (2.5) | 1152 (62.7) | 796 (42.3) | 305 (16.2) |
| 35 |  | Palghar | 2004 (2.6) | 1418 (72.2) | 829 (41.4) | 267 (13.3) |
| 36 |  | Thane | 6133 (8.0) | 3616 (72.1) | 1869 (30.5) | 811 (13.2) |
|  |  | **Overall Maharashtra** | 76602 (100.0) | 45644 (64.5) | 26420 (34.5) | 10602 (13.8) |
| 1 | **4.Goa** | North Goa | 682 (56.8) | 528 (81.4) | 136 (19.9) | 92 (13.5) |
| 2 |  | South Goa | 518 (43.2) | 467 (91.5) | 116 (22.5) | 62 (11.9) |
|  |  | **Overall Goa** | 1199 (100.0) | 994 (85.8) | 252 (21.0) | 154 (12.8) |

## **Table S5: District wise prevalence of Ever measured blood pressure (BP), Prehypertension, and Raised blood pressure in the states of southern region of India (%) (National Family Health Survey-5, India, 2019-2021)**

| Sl. NO | State name |  | A. | B | C | D |
| --- | --- | --- | --- | --- | --- | --- |
|  |  | **District name** | **Total population analysed*** | **Ever measured blood pressure among the population (B/A)** | **Prevalence of prehypertension in the population(C/A)** | **Prevalence of raised BP in the population(D/A)** |
|  |  |  | **n(%)** | **n(%)** | **n(%)** | **n(%)** |
| Overall Indian population | | | 743067 (100.0) | 472130 (66.7) | 250624 (33.7) | 118231 (15.9) |
| Overall southern region | | | 159876 (21.5) | 115260 (75.8) | 48268 (30.2) | 26843 (16.8) |
| 1 | **1.Andhra Pradesh** | Srikakulam | 1546 (4.8) | 1075 (73.0) | 424 (27.4) | 248 (16.0) |
| 2 |  | Vizianagaram | 1433 (4.4) | 889 (63.7) | 373 (26.1) | 231 (16.1) |
| 3 |  | Visakhapatnam | 2934 (9.0) | 1407 (51.3) | 729 (24.9) | 509 (17.4) |
| 4 |  | East Godavari | 3452 (10.6) | 2113 (66.4) | 857 (24.8) | 623 (18.0) |
| 5 |  | West Godavari | 2698 (8.3) | 1983 (77.5) | 841 (31.2) | 541 (20.0) |
| 6 |  | Krishna | 2998 (9.2) | 2396 (84.5) | 1038 (34.6) | 369 (12.3) |
| 7 |  | Guntur | 3210 (9.9) | 2249 (72.4) | 1007 (31.4) | 641 (20.0) |
| 8 |  | Prakasam | 1963 (6.1) | 1542 (82.5) | 567 (28.9) | 336 (17.1) |
| 9 |  | Sri Potti Sriramulu Nellore | 2228 (6.9) | 1815 (84.5) | 756 (33.9) | 358 (16.1) |
| 10 |  | Y.S.R. | 2110 (6.5) | 1780 (85.1) | 760 (36.0) | 327 (15.5) |
| 11 |  | Kurnool | 2603 (8.0) | 1872 (73.6) | 813 (31.2) | 458 (17.6) |
| 12 |  | Anantapur | 2740 (8.4) | 2124 (79.2) | 890 (32.5) | 412 (15.0) |
| 13 |  | Chittoor | 2515 (7.8) | 1929 (80.7) | 619 (24.6) | 339 (13.5) |
|  |  | **Overall Andhra Pradesh** | 32430 (100.0) | 23174 (74.7) | 9675 (29.8) | 5389 (16.6) |
| 1 | **2.Karnataka** | Belgaum | 3486 (8.2) | 1970 (59.1) | 965 (27.7) | 406 (11.7) |
| 2 |  | Bagalkot | 1241 (2.9) | 728 (60.6) | 231 (18.6) | 146 (11.8) |
| 3 |  | Bijapur | 1331 (3.1) | 590 (47.9) | 332 (24.9) | 177 (13.3) |
| 4 |  | Bidar | 1210 (2.8) | 724 (61.9) | 364 (30.1) | 225 (18.6) |
| 5 |  | Raichur | 1440 (3.4) | 786 (56.2) | 323 (22.4) | 170 (11.8) |
| 6 |  | Koppal | 907 (2.1) | 440 (52.4) | 252 (27.8) | 113 (12.5) |
| 7 |  | Gadag | 834 (2.0) | 406 (51.0) | 220 (26.3) | 114 (13.7) |
| 8 |  | Dharwad | 1406 (3.3) | 1016 (74.4) | 348 (24.8) | 222 (15.8) |
| 9 |  | Uttara Kannada | 1189 (2.8) | 485 (43.0) | 352 (29.6) | 170 (14.3) |
| 10 |  | Haveri | 1153 (2.7) | 593 (57.2) | 282 (24.5) | 153 (13.3) |
| 11 |  | Bellary | 1684 (4.0) | 834 (50.6) | 607 (36.0) | 236 (14.0) |
| 12 |  | Chitradurga | 986 (2.3) | 587 (60.5) | 369 (37.5) | 201 (20.4) |
| 13 |  | Davanagere | 1346 (3.2) | 636 (54.4) | 262 (19.5) | 226 (16.8) |
| 14 |  | Shimoga | 1220 (2.9) | 695 (57.8) | 410 (33.6) | 275 (22.5) |
| 15 |  | Udupi | 841 (2.0) | 612 (75.5) | 234 (27.8) | 135 (16.1) |
| 16 |  | Chikmagalur | 764 (1.8) | 502 (67.2) | 222 (29.1) | 148 (19.3) |
| 17 |  | Tumkur | 1893 (4.4) | 1406 (75.2) | 718 (37.9) | 325 (17.2) |
| 18 |  | Bangalore | 6460 (15.2) | 3246 (57.0) | 2300 (35.6) | 1257 (19.5) |
| 19 |  | Mandya | 1143 (2.7) | 759 (70.7) | 362 (31.7) | 201 (17.6) |
| 20 |  | Hassan | 1283 (3.0) | 834 (65.9) | 443 (34.5) | 277 (21.6) |
| 21 |  | Dakshina Kannada | 1485 (3.5) | 859 (62.5) | 486 (32.7) | 227 (15.3) |
| 22 |  | Kodagu | 341 (0.8) | 229 (69.5) | 106 (31.1) | 61 (17.8) |
| 23 |  | Mysore | 2500 (5.9) | 1853 (76.5) | 890 (35.6) | 385 (15.4) |
| 24 |  | Chamarajanagar | 768 (1.8) | 423 (57.0) | 239 (31.1) | 162 (21.1) |
| 25 |  | Gulbarga | 1695 (4.0) | 904 (56.1) | 447 (26.4) | 311 (18.3) |
| 26 |  | Yadgir | 880 (2.1) | 465 (54.2) | 184 (20.9) | 72 (8.2) |
| 27 |  | Kolar | 1018 (2.4) | 751 (76.2) | 394 (38.7) | 164 (16.1) |
| 28 |  | Chikkaballapura | 757 (1.8) | 521 (71.0) | 230 (30.4) | 133 (17.6) |
| 29 |  | Bangalore Rural | 662 (1.6) | 503 (77.8) | 256 (38.7) | 119 (18.0) |
| 30 |  | Ramanagara | 654 (1.5) | 427 (66.9) | 232 (35.4) | 102 (15.7) |
|  |  | **Overall Karnataka** | 42577 (100.0) | 24784 (61.5) | 13060 (30.7) | 6914 (16.2) |
| 1 | **3.Lakshadweep** | Lakshadweep | 46 (100.0) | 41 (90.8) | 18 (40.1) | 6 (12.1) |
|  |  | **Overall Lakshadweep** | 46 (100.0) | 41 (90.8) | 18 (40.1) | 6 (12.1) |
| 1 | **4.Kerala** | Kasaragod | 864 (4.3) | 771 (90.5) | 329 (38.1) | 102 (11.8) |
| 2 |  | Kannur | 1573 (7.9) | 1416 (93.6) | 566 (36.0) | 226 (14.4) |
| 3 |  | Wayanad | 512 (2.6) | 470 (91.8) | 194 (37.8) | 78 (15.2) |
| 4 |  | Kozhikode | 1873 (9.4) | 1706 (92.3) | 821 (43.8) | 237 (12.6) |
| 5 |  | Malappuram | 2675 (13.4) | 2413 (92.2) | 1035 (38.7) | 354 (13.2) |
| 6 |  | Palakkad | 1738 (8.7) | 1467 (86.8) | 522 (30.0) | 310 (17.8) |
| 7 |  | Thrissur | 1707 (8.5) | 1456 (87.7) | 420 (24.6) | 291 (17.0) |
| 8 |  | Ernakulam | 2029 (10.1) | 1676 (86.4) | 782 (38.5) | 313 (15.4) |
| 9 |  | Idukki | 667 (3.3) | 561 (85.6) | 242 (36.3) | 125 (18.7) |
| 10 |  | Kottayam | 1071 (5.4) | 819 (79.9) | 266 (24.8) | 195 (18.2) |
| 11 |  | Alappuzha | 1227 (6.1) | 1050 (88.7) | 356 (29.0) | 224 (18.2) |
| 12 |  | Pathanamthitta | 604 (3.0) | 518 (90.2) | 158 (26.2) | 118 (19.5) |
| 13 |  | Kollam | 1532 (7.7) | 1289 (86.2) | 410 (26.8) | 262 (17.1) |
| 14 |  | Thiruvananthapuram | 1938 (9.7) | 1483 (84.7) | 478 (24.7) | 259 (13.3) |
|  |  | **Overall Kerala** | 20010 (100.0) | 17095 (88.5) | 6579 (32.9) | 3093 (15.5) |
| 1 | **5.Tamil Nadu** | Thiruvallur | 2272 (5.4) | 1661 (77.5) | 482 (21.2) | 484 (21.3) |
| 2 |  | Chennai | 2648 (6.3) | 1820 (84.3) | 713 (26.9) | 455 (17.2) |
| 3 |  | Kancheepuram | 2573 (6.1) | 1892 (79.5) | 584 (22.7) | 510 (19.8) |
| 4 |  | Vellore | 2308 (5.5) | 1866 (85.4) | 688 (29.8) | 370 (16.0) |
| 5 |  | Tiruvannamalai | 1280 (3.0) | 1086 (85.5) | 417 (32.6) | 165 (12.9) |
| 6 |  | Viluppuram | 1800 (4.3) | 1391 (78.0) | 449 (24.9) | 316 (17.6) |
| 7 |  | Salem | 1908 (4.5) | 1729 (91.6) | 618 (32.4) | 333 (17.5) |
| 8 |  | Namakkal | 864 (2.0) | 725 (86.2) | 305 (35.3) | 111 (12.8) |
| 9 |  | Erode | 1700 (4.0) | 1409 (85.4) | 591 (34.8) | 275 (16.2) |
| 10 |  | The Nilgiris | 506 (1.2) | 454 (90.3) | 204 (40.3) | 114 (22.5) |
| 11 |  | Dindigul | 1500 (3.5) | 1347 (90.3) | 513 (34.2) | 250 (16.7) |
| 12 |  | Karur | 546 (1.3) | 436 (85.6) | 140 (25.7) | 68 (12.4) |
| 13 |  | Tiruchirappalli | 1524 (3.6) | 1292 (86.7) | 574 (37.7) | 147 (9.6) |
| 14 |  | Perambalur | 304 (0.7) | 250 (84.0) | 88 (28.8) | 43 (14.0) |
| 15 |  | Ariyalur | 405 (1.0) | 312 (77.6) | 112 (27.7) | 61 (15.2) |
| 16 |  | Cuddalore | 1559 (3.7) | 1246 (80.9) | 445 (28.6) | 197 (12.6) |
| 17 |  | Nagapattinam | 1040 (2.5) | 841 (81.1) | 253 (24.3) | 207 (19.9) |
| 18 |  | Thiruvarur | 655 (1.5) | 544 (84.3) | 203 (31.0) | 147 (22.4) |
| 19 |  | Thanjavur | 1089 (2.6) | 866 (82.7) | 326 (29.9) | 177 (16.2) |
| 20 |  | Pudukkottai | 887 (2.1) | 748 (86.9) | 266 (30.0) | 118 (13.3) |
| 21 |  | Sivaganga | 794 (1.9) | 649 (81.7) | 260 (32.8) | 114 (14.4) |
| 22 |  | Madurai | 1936 (4.6) | 1409 (73.8) | 541 (28.0) | 608 (31.4) |
| 23 |  | Theni | 820 (1.9) | 745 (91.4) | 295 (35.9) | 163 (19.9) |
| 24 |  | Virudhunagar | 1254 (3.0) | 843 (68.6) | 437 (34.8) | 188 (15.0) |
| 25 |  | Ramanathapuram | 880 (2.1) | 727 (82.9) | 220 (25.0) | 255 (29.0) |
| 26 |  | Thoothukkudi | 1116 (2.6) | 820 (74.7) | 332 (29.8) | 183 (16.4) |
| 27 |  | Tirunelveli | 1849 (4.4) | 1483 (81.4) | 445 (24.1) | 516 (27.9) |
| 28 |  | Kanniyakumari | 905 (2.1) | 757 (86.9) | 228 (25.2) | 199 (21.9) |
| 29 |  | Dharmapuri | 932 (2.2) | 721 (78.6) | 328 (35.1) | 158 (16.9) |
| 30 |  | Krishnagiri | 1047 (2.5) | 824 (79.3) | 369 (35.2) | 158 (15.1) |
| 31 |  | Coimbatore | 2013 (4.8) | 1801 (93.3) | 598 (29.7) | 313 (15.6) |
| 32 |  | Tiruppur | 1398 (3.3) | 1273 (93.5) | 554 (39.7) | 176 (12.6) |
|  |  | **Overall Tamil Nadu** | 42312 (100.0) | 33969 (83.3) | 12578 (29.7) | 7578 (17.9) |
| 1 | **6.Puducherry** | Yanam | 31 (4.5) | 29 (94.2) | 13 (40.8) | 6 (21.1) |
| 2 |  | Puducherry | 492 (72.6) | 385 (81.2) | 131 (26.7) | 64 (13.1) |
| 3 |  | Mahe | 20 (2.9) | 19 (98.5) | 10 (51.5) | 3 (13.3) |
| 4 |  | Karaikal | 136 (20.0) | 115 (85.8) | 34 (25.0) | 15 (11.2) |
|  |  | **Overall Puducherry** | 678 (100.0) | 548 (83.2) | 188 (27.7) | 89 (13.1) |
| 1 | **7.Andaman & Nicobar Islands** | Nicobars | 17 (7.6) | 13 (76.7) | 6 (37.0) | 4 (24.2) |
| 2 |  | North & Middle Andaman | 66 (28.9) | 57 (87.0) | 31 (47.9) | 12 (18.3) |
| 3 |  | South Andaman | 145 (63.5) | 123 (86.2) | 46 (31.8) | 27 (18.4) |
|  |  | **Overall Andaman & Nicobar Islands** | 228 (100.0) | 193 (85.7) | 84 (36.9) | 43 (18.8) |
| 1 | 8.Telangana | Adilabad | 479 (2.2) | 365 (79.3) | 161 (33.6) | 77 (16.0) |
| 2 |  | Bhadradri Kothagudem | 769 (3.6) | 625 (83.0) | 260 (33.9) | 160 (20.8) |
| 3 |  | Hyderabad | 2679 (12.4) | 1588 (81.7) | 633 (23.6) | 451 (16.8) |
| 4 |  | Jagitial | 708 (3.3) | 562 (83.0) | 224 (31.7) | 119 (16.7) |
| 5 |  | Jangoan | 378 (1.8) | 252 (69.4) | 111 (29.3) | 62 (16.5) |
| 6 |  | Jayashankar Bhupalapally | 483 (2.2) | 359 (75.5) | 159 (32.8) | 108 (22.2) |
| 7 |  | Jogulamba Gadwal | 387 (1.8) | 303 (79.7) | 114 (29.5) | 59 (15.2) |
| 8 |  | Kamareddy | 563 (2.6) | 397 (74.8) | 129 (23.0) | 80 (14.2) |
| 9 |  | Karimnagar | 665 (3.1) | 455 (72.5) | 161 (24.3) | 117 (17.6) |
| 10 |  | Khammam | 1083 (5.0) | 922 (87.2) | 347 (32.0) | 193 (17.9) |
| 11 |  | Komaram Bheem Asifabad | 337 (1.6) | 237 (73.2) | 115 (34.0) | 53 (15.8) |
| 12 |  | Mahabubabad | 560 (2.6) | 409 (75.8) | 166 (29.6) | 81 (14.4) |
| 13 |  | Mahabubnagar | 870 (4.0) | 720 (85.0) | 280 (32.2) | 153 (17.6) |
| 14 |  | Mancherial | 510 (2.4) | 372 (77.4) | 122 (23.8) | 92 (18.1) |
| 15 |  | Medak | 455 (2.1) | 298 (70.5) | 97 (21.4) | 81 (17.8) |
| 16 |  | Medchal-Malkajgiri | 354 (1.6) | 226 (78.2) | 87 (24.7) | 48 (13.6) |
| 17 |  | Nagarkurnool | 544 (2.5) | 443 (83.6) | 167 (30.7) | 93 (17.0) |
| 18 |  | Nalgonda | 1056 (4.9) | 760 (75.7) | 272 (25.8) | 180 (17.0) |
| 19 |  | Nirmal | 419 (1.9) | 298 (77.2) | 128 (30.5) | 68 (16.3) |
| 20 |  | Nizamabad | 1003 (4.6) | 664 (73.0) | 260 (25.9) | 182 (18.1) |
| 21 |  | Peddapalli | 563 (2.6) | 396 (72.6) | 174 (30.8) | 106 (18.7) |
| 22 |  | Rajanna Sircilla | 375 (1.7) | 312 (87.9) | 117 (31.3) | 80 (21.5) |
| 23 |  | Ranga Reddy | 1606 (7.4) | 1081 (75.2) | 474 (29.5) | 283 (17.6) |
| 24 |  | Sangareddy | 759 (3.5) | 504 (71.5) | 175 (23.0) | 131 (17.3) |
| 25 |  | Siddipet | 676 (3.1) | 461 (79.1) | 143 (21.2) | 110 (16.3) |
| 26 |  | Suryapet | 695 (3.2) | 567 (83.3) | 220 (31.6) | 121 (17.4) |
| 27 |  | Vikarabad | 569 (2.6) | 288 (55.9) | 151 (26.5) | 108 (19.0) |
| 28 |  | Wanaparthy | 334 (1.5) | 254 (77.9) | 105 (31.4) | 49 (14.8) |
| 29 |  | Warangal Rural | 494 (2.3) | 343 (72.6) | 146 (29.6) | 88 (17.9) |
| 30 |  | Warangal Urban | 738 (3.4) | 642 (89.0) | 235 (31.9) | 125 (16.9) |
| 31 |  | Yadadri Bhuvanagiri | 481 (2.2) | 352 (75.5) | 152 (31.5) | 72 (15.0) |
|  |  | **Overall Telangana** | 21596 (100.0) | 15455 (78.0) | 6086 (28.2) | 3731 (17.3) |

## **Table S6: District wise prevalence of Ever measured blood pressure (BP), Prehypertension, and Raised blood pressure in northeastern region of India (%) (National Family Health Survey-5, India, 2019-2021)**

| Sl. NO | State name |  | A. | B | C | D |
| --- | --- | --- | --- | --- | --- | --- |
|  |  | **District name** | **Total population analysed*** | **Ever measured blood pressure among the population (B/A)** | **Prevalence of prehypertension in the population(C/A)** | **Prevalence of raised BP in the population(D/A)** |
|  |  |  | **n(%)** | **n(%)** | **n(%)** | **n(%)** |
| **Overall Indian population** | | | 743067 (100.0) | 472130 (66.7) | 250624 (33.7) | 118231 (15.9) |
| **Overalll north -eastern region** | | | 29246 (3.9) | 19433 (68.1) | 10341 (35.4) | 4763 (16.3) |
| 1 | **1.Sikkim** | North District | 17 (4.4) | 14 (83.5) | 6 (34.3) | 7 (38.6) |
| 2 |  | West District | 79 (20.4) | 51 (65.4) | 41 (52.3) | 20 (24.9) |
| 3 |  | South District | 82 (21.2) | 71 (87.8) | 30 (35.9) | 29 (35.0) |
| 4 |  | East District | 209 (53.9) | 136 (73.7) | 57 (27.4) | 58 (27.7) |
|  |  | **Overall Sikkim** | 387 (100) | 272 (75.5) | 134 (34.6) | 113 (29.1) |
| 1 | **2.Arunachal Pradesh** | Tawang | 21 (3.7) | 12 (60.8) | 10 (47.0) | 6 (29.3) |
| 2 |  | West Kameng | 34 (5.9) | 23 (70.1) | 17 (49.9) | 8 (23.6) |
| 3 |  | East Kameng | 27 (4.7) | 16 (58.5) | 11 (41.9) | 6 (22.3) |
| 4 |  | Papum Pare | 48 (8.4) | 35 (74.5) | 17 (34.3) | 14 (29.8) |
| 5 |  | Upper Subansiri | 36 (6.3) | 19 (53.3) | 16 (43.5) | 10 (28.7) |
| 6 |  | Upper Siang | 12 (2.1) | 7 (54.3) | 5 (40.2) | 3 (21.9) |
| 7 |  | Changlang | 61 (10.6) | 44 (73.7) | 24 (39.9) | 11 (18.4) |
| 8 |  | Lower Subansiri | 34 (6.0) | 24 (70.7) | 14 (41.5) | 10 (29.3) |
| 9 |  | Dibang Valley | 5 (0.8) | 3 (59.6) | 3 (53.2) | 1 (30.5) |
| 10 |  | Lower Dibang Valley | 33 (5.8) | 20 (59.4) | 17 (50.4) | 9 (26.6) |
| 11 |  | Anjaw | 10 (1.7) | 6 (58.2) | 5 (51.2) | 3 (27.9) |
| 12 |  | East Siang | 39 (6.9) | 23 (60.5) | 14 (35.4) | 11 (29.1) |
| 13 |  | Kra Daadi | 14 (2.5) | 9 (63.1) | 7 (46.0) | 3 (19.3) |
| 14 |  | Kurung Kumey | 23 (3.9) | 10 (42.7) | 11 (50.5) | 4 (15.8) |
| 15 |  | Lohit | 23 (4.0) | 15 (66.1) | 9 (38.9) | 5 (20.1) |
| 16 |  | Longding | 17 (2.9) | 11 (64.7) | 8 (48.3) | 2 (13.7) |
| 17 |  | Namsai | 51 (9.0) | 39 (76.0) | 19 (37.3) | 12 (23.1) |
| 18 |  | Siang | 12 (2.0) | 6 (54.0) | 6 (50.7) | 3 (26.0) |
| 19 |  | Tirap | 26 (4.5) | 17 (67.8) | 10 (41.1) | 6 (22.2) |
| 20 |  | West Siang | 48 (8.3) | 30 (65.1) | 19 (40.9) | 14 (29.9) |
| 21 |  | **Overall Arunachal Pradesh** | 572 (100) | 368 (65.1) | 241 (42.2) | 141 (24.6) |
| 1 | **3.Nagaland** | Mon | 89 (10.3) | 54 (61.5) | 41 (46.5) | 14 (15.5) |
| 2 |  | Mokokchung | 82 (9.5) | 40 (49.4) | 36 (43.5) | 17 (20.8) |
| 3 |  | Zunheboto | 45 (5.2) | 25 (56.3) | 18 (40.3) | 11 (25.0) |
| 4 |  | Wokha | 49 (5.7) | 35 (70.4) | 21 (42.9) | 9 (18.7) |
| 5 |  | Dimapur | 215 (24.9) | 142 (66.4) | 76 (35.2) | 38 (17.9) |
| 6 |  | Phek | 75 (8.7) | 43 (56.8) | 34 (44.8) | 14 (19.1) |
| 7 |  | Tuensang | 102 (11.8) | 40 (39.4) | 39 (38.0) | 16 (15.8) |
| 8 |  | Longleng | 27 (3.1) | 20 (74.1) | 10 (36.1) | 6 (21.0) |
| 9 |  | Kiphire | 29 (3.4) | 14 (46.9) | 10 (34.5) | 4 (13.9) |
| 10 |  | Kohima | 115 (13.2) | 65 (57.1) | 47 (41.1) | 21 (18.0) |
| 11 |  | Peren | 36 (4.2) | 17 (47.9) | 15 (41.4) | 7 (19.9) |
| 12 |  | **Overall Nagaland** | 865 (100) | 495 (57.5) | 347 (40.1) | 158 (18.3) |
| 1 | **4.Manipur** | Senapati | 63 (4.5) | 48 (76.2) | 25 (38.9) | 10 (15.5) |
| 2 |  | Tamenglong | 49 (3.5) | 38 (78.4) | 18 (36.0) | 9 (18.3) |
| 3 |  | Churachandpur | 100 (7.1) | 78 (78.3) | 36 (35.4) | 14 (14.0) |
| 4 |  | Bishnupur | 150 (10.7) | 121 (82.4) | 46 (30.6) | 29 (19.2) |
| 5 |  | Thoubal | 296 (21.0) | 248 (84.2) | 125 (42.2) | 58 (19.5) |
| 6 |  | Imphal West | 310 (22.0) | 275 (89.3) | 134 (43.3) | 65 (21.0) |
| 7 |  | Imphal East | 323 (22.9) | 276 (86.6) | 101 (31.2) | 68 (21.1) |
| 8 |  | Ukhrul | 67 (4.7) | 52 (78.2) | 22 (33.4) | 12 (17.8) |
| 9 |  | Chandel | 50 (3.6) | 45 (88.8) | 19 (38.4) | 10 (19.5) |
| 10 |  | **Overall Manipur** | 1409 (0.2) | 1181 (84.6) | 525 (37.3) | 274 (19.5) |
| 1 | **5.Mizoram** | Mamit | 50 (7.6) | 37 (74.8) | 20 (39.8) | 8 (16.2) |
| 2 |  | Kolasib | 52 (8.1) | 45 (85.7) | 20 (37.7) | 8 (14.9) |
| 3 |  | Aizawl | 258 (39.8) | 203 (90.2) | 78 (30.2) | 57 (21.9) |
| 4 |  | Champhai | 66 (10.1) | 54 (82.1) | 25 (38.3) | 10 (15.3) |
| 5 |  | Serchhip | 35 (5.3) | 29 (82.5) | 12 (33.9) | 4 (12.1) |
| 6 |  | Lunglei | 90 (13.9) | 66 (73.9) | 37 (41.4) | 11 (12.4) |
| 7 |  | Lawngtlai | 69 (10.6) | 43 (62.2) | 23 (32.9) | 11 (15.2) |
| 8 |  | Saiha | 30 (4.6) | 21 (72.1) | 10 (32.5) | 4 (13.8) |
| 9 |  | **Overall Mizoram** | 649 (100) | 497 (80.9) | 224 (34.5) | 113 (17.3) |
| 1 | **6.Tripura** | Dhalai | 264 (10.7) | 173 (67.0) | 95 (35.9) | 32 (12.0) |
| 2 |  | Gomati | 262 (10.6) | 135 (52.8) | 89 (34.2) | 46 (17.5) |
| 3 |  | Khowai | 227 (9.2) | 167 (74.5) | 78 (34.4) | 45 (19.6) |
| 4 |  | North Tripura | 271 (11.0) | 172 (64.3) | 96 (35.6) | 40 (14.6) |
| 5 |  | Sepahijala | 310 (12.5) | 216 (71.5) | 103 (33.3) | 51 (16.6) |
| 6 |  | South Tripura | 284 (11.5) | 209 (74.3) | 94 (33.1) | 55 (19.4) |
| 7 |  | Unakoti | 167 (6.8) | 127 (76.6) | 44 (26.4) | 31 (18.4) |
| 8 |  | West Tripura | 686 (27.8) | 581 (85.0) | 235 (34.2) | 134 (19.6) |
| 9 |  | **Overall Tripura** | 2470 (100) | 1780 (73.0) | 834 (33.8) | 433 (17.5) |
| 1 | **7.Meghalaya** | South Garo Hills | 77 (4.0) | 30 (38.9) | 44 (56.8) | 13 (16.5) |
| 2 |  | Ribhoi | 169 (8.8) | 120 (71.1) | 55 (32.3) | 21 (12.4) |
| 3 |  | East Khasi Hills | 567 (29.4) | 356 (72.9) | 137 (24.2) | 108 (19.0) |
| 4 |  | East Garo Hills | 117 (6.1) | 35 (30.3) | 60 (51.7) | 20 (17.2) |
| 5 |  | East Jantia Hills | 84 (4.4) | 65 (77.0) | 24 (28.9) | 14 (16.7) |
| 6 |  | North Garo Hills | 105 (5.4) | 34 (33.2) | 45 (42.8) | 22 (20.7) |
| 7 |  | South West Garo Hills | 102 (5.3) | 42 (41.2) | 51 (49.8) | 12 (11.8) |
| 8 |  | South West Khasi Hills | 58 (3.0) | 40 (69.1) | 18 (31.2) | 10 (17.5) |
| 9 |  | West Garo Hills | 319 (16.6) | 168 (52.9) | 151 (47.3) | 58 (18.2) |
| 10 |  | West Jaintia Hills | 163 (8.4) | 113 (70.9) | 44 (26.9) | 27 (16.5) |
| 11 |  | West Khasi Hills | 167 (8.7) | 120 (71.6) | 52 (30.9) | 23 (14.0) |
| 12 |  | **Overall Meghalaya** | 1928 (100) | 1123 (61.0) | 681 (35.3) | 328 (17.0) |
| 1 | **8.Assam** | Kokrajhar | 590 (2.8) | 364 (63.8) | 233 (39.5) | 92 (15.6) |
| 2 |  | Goalpara | 636 (3.0) | 379 (60.1) | 209 (32.8) | 101 (15.9) |
| 3 |  | Barpeta | 1126 (5.4) | 670 (60.3) | 350 (31.1) | 197 (17.5) |
| 4 |  | Morigaon | 617 (2.9) | 492 (80.9) | 246 (39.8) | 98 (15.9) |
| 5 |  | Lakhimpur | 599 (2.9) | 473 (79.9) | 261 (43.5) | 103 (17.1) |
| 6 |  | Dhemaji | 418 (2.0) | 302 (73.7) | 186 (44.5) | 55 (13.2) |
| 7 |  | Tinsukia | 868 (4.1) | 607 (71.7) | 294 (33.8) | 118 (13.6) |
| 8 |  | Dibrugarh | 998 (4.8) | 744 (75.9) | 338 (33.8) | 129 (12.9) |
| 9 |  | Golaghat | 708 (3.4) | 502 (72.9) | 260 (36.7) | 133 (18.8) |
| 10 |  | Dima Hasao | 136 (0.6) | 99 (76.6) | 57 (42.2) | 18 (13.2) |
| 11 |  | Cachar | 1267 (6.0) | 743 (58.9) | 441 (34.8) | 142 (11.2) |
| 12 |  | Karimganj | 856 (4.1) | 521 (61.2) | 326 (38.1) | 106 (12.3) |
| 13 |  | Hailakandi | 494 (2.4) | 314 (65.3) | 172 (34.8) | 74 (14.9) |
| 14 |  | Bongaigaon | 467 (2.2) | 256 (56.2) | 165 (35.3) | 58 (12.4) |
| 15 |  | Chirang | 302 (1.4) | 180 (60.4) | 111 (36.8) | 52 (17.2) |
| 16 |  | Kamrup | 1088 (5.2) | 634 (62.1) | 304 (27.9) | 184 (16.9) |
| 17 |  | Kamrup Metropolitan | 894 (4.3) | 644 (76.9) | 275 (30.8) | 115 (12.8) |
| 18 |  | Nalbari | 530 (2.5) | 313 (64.1) | 148 (28.0) | 83 (15.6) |
| 19 |  | Baksa | 682 (3.3) | 354 (52.8) | 202 (29.7) | 123 (18.0) |
| 20 |  | Darrang | 601 (2.9) | 339 (58.1) | 155 (25.8) | 71 (11.8) |
| 21 |  | Udalguri | 557 (2.7) | 282 (51.4) | 183 (32.9) | 88 (15.9) |
| 22 |  | Biswanath | 298 (1.4) | 228 (77.7) | 129 (43.3) | 56 (18.7) |
| 23 |  | Charaideo | 317 (1.5) | 221 (71.7) | 110 (34.7) | 36 (11.4) |
| 24 |  | Dhubri | 879 (4.2) | 492 (56.3) | 346 (39.4) | 142 (16.1) |
| 25 |  | Hojai | 156 (0.7) | 102 (67.1) | 65 (41.6) | 25 (15.8) |
| 26 |  | Jorhat | 662 (3.2) | 463 (74.3) | 196 (29.7) | 114 (17.3) |
| 27 |  | Karbi Anglong | 429 (2.0) | 296 (70.7) | 188 (43.7) | 74 (17.2) |
| 28 |  | Majuli | 102 (0.5) | 75 (75.8) | 45 (43.9) | 16 (15.4) |
| 29 |  | Nagaon | 1771 (8.4) | 1310 (75.1) | 581 (32.8) | 239 (13.5) |
| 30 |  | Sivasagar | 468 (2.2) | 363 (78.6) | 160 (34.1) | 86 (18.4) |
| 31 |  | Sonitpur | 886 (4.2) | 599 (68.2) | 368 (41.5) | 193 (21.8) |
| 32 |  | South Salmara Mancachar | 395 (1.9) | 235 (60.1) | 172 (43.6) | 62 (15.6) |
| 33 |  | West Karbi Anglong | 171 (0.8) | 118 (70.2) | 81 (47.0) | 23 (13.2) |
| 34 |  | **Overall Assam** | 20965 (100) | 13717 (67.0) | 7355 (35.1) | 3204 (15.3) |

## **Table S7: State wise determinants of Ever measured blood pressure among adults in India (AOR) (National Family Health Survey-5, India, 2019-2021)**

| **State Name** | **Age Group (>=30)** | **Sex (Female)** | **Education (Literate)** | **Occupation (Employed)** | **Household wealth quintile (middle)** | **Household wealth quintile (richer/richest)** | **Marital status (Currently married)** | **Place Of Residence (Urban)** | **Tobacco consumption (Yes)** | **Alcohol consumption (Yes)** | **BMI (Overweight/Obese)** | **Central obesity (Present)** | **Blood glucose level (Raised)** |
| --- | --- | --- | --- | --- | --- | --- | --- | --- | --- | --- | --- | --- | --- |
| JAMMU & KASHMIR | H | H | NS | NS | NS | H | H | H | NS | NS | H | NS | NS |
| HIMACHAL PRADESH | H | H | NS | NS | NS | NS | H | NS | NS | NS | H | NS | NS |
| PUNJAB | H | H | NS | NS | NS | NS | H | NS | NS | NS | H | NS | NS |
| CHANDIGARH | NS | NS | NS | NS | NS | NS | NS | NS | NS | NS | NS | NS | NS |
| UTTARAKHAND | NS | NS | NS | NS | NS | H | H | NS | NS | NS | NS | NS | NS |
| HARYANA | H | H | NS | NS | NS | NS | H | NS | NS | NS | H | NS | NS |
| NCT OF DELHI | H | H | NS | NS | NS | H | H | NS | NS | NS | H | NS | NS |
| RAJASTHAN | H | H | NS | NS | NS | NS | H | H | NS | NS | H | NS | H |
| UTTAR PRADESH | H | H | NS | NS | H | H | H | H | L | NS | H | H | H |
| BIHAR | H | H | H | NS | H | H | H | H | NS | NS | H | H | H |
| SIKKIM | NS | NS | NS | NS | NS | NS | NS | NS | NS | NS | NS | NS | NS |
| ARUNACHAL PRADESH | NS | NS | NS | NS | NS | NS | NS | NS | NS | NS | NS | NS | NS |
| NAGALAND | NS | NS | NS | NS | NS | NS | NS | NS | NS | NS | NS | NS | NS |
| MANIPUR | NS | NS | NS | NS | NS | NS | NS | H | NS | NS | NS | NS | NS |
| MIZORAM | NS | NS | NS | NS | NS | NS | NS | NS | NS | NS | NS | NS | NS |
| TRIPURA | NS | H | NS | NS | NS | NS | NS | NS | NS | NS | NS | NS | NS |
| MEGHALAYA | NS | NS | NS | NS | NS | NS | NS | NS | NS | NS | NS | NS | NS |
| ASSAM | H | H | NS | NS | H | H | H | NS | NS | NS | H | H | NS |
| WEST BENGAL | H | H | H | H | H | H | H | H | H | NS | H | H | NS |
| JHARKHAND | H | NS | H | NS | NS | H | H | NS | NS | NS | H | NS | NS |
| ODISHA | NS | H | NS | NS | H | H | H | NS | NS | NS | NS | H | NS |
| CHHATTISGARH | NS | H | NS | NS | H | H | H | NS | NS | NS | NS | H | NS |
| MADHYA PRADESH | NS | H | H | NS | H | H | H | H | NS | NS | NS | H | NS |
| GUJARAT | H | H | H | NS | H | H | H | H | H | H | H | NS | NS |
| DADRA & NAGAR HAVELI AND DAMAN & DIU | NS | NS | NS | NS | NS | NS | NS | NS | NS | NS | NS | NS | NS |
| MAHARASHTRA | NS | H | H | H | H | H | H | H | H | H | H | H | H |
| ANDHRA PRADESH | H | H | NS | NS | NS | NS | H | NS | NS | NS | NS | H | NS |
| KARNATAKA | H | H | H | NS | H | H | H | NS | NS | NS | L | H | NS |
| GOA | NS | NS | NS | NS | NS | NS | H | NS | NS | NS | NS | NS | NS |
| LAKSHADWEEP | NS | NS | NS | NS | NS | NS | NS | NS | NS | NS | NS | NS | NS |
| KERALA | H | H | NS | H | NS | NS | H | H | NS | NS | H | H | NS |
| TAMIL NADU | H | H | NS | NS | NS | NS | H | NS | NS | NS | H | NS | NS |
| PUDUCHERRY | NS | NS | NS | NS | NS | NS | NS | NS | NS | NS | NS | NS | NS |
| ANDAMAN & NICOBAR ISLANDS | NS | NS | NS | NS | NS | NS | NS | NS | NS | NS | NS | NS | NS |
| TELANGANA | H | H | H | NS | H | H | H | NS | NS | NS | NS | NS | NS |
| LADAKH | NS | NS | NS | NS | NS | NS | NS | NS | NS | NS | NS | NS | NS |
| **H%** | **47.2** | **58.3** | **22.2** | **8.3** | **30.6** | **41.7** | **63.9** | **27.8** | **8.3** | **5.6** | **41.7** | **30.6** | **11.1** |
| **L%** | **0.0** | **0.0** | **0.0** | **0.0** | **0.0** | **0.0** | **0.0** | **0.0** | **2.8** | **0.0** | **2.8** | **0.0** | **0.0** |

*H: Higher odds [Adjusted odds ratio(AOR) >1 and P<0.05], L: Lower odds (AOR<1 and P<0.05), NS: Not Significant (p>0.05), NA:* *Data Not Available.*

## **Table S8: State wise determinants Prehypertension among adults in India (AOR) (National Family Health Survey-5, India, 2019-2021)**

| **State Name** | **Age Group (>=30)** | **Sex (Female)** | **Education (Literate)** | **Occupation (Employed)** | **Household wealth quintile (middle)** | **Household wealth quintile (richer/richest)** | **Marital status (Currently married)** | **Place Of Residence (Urban)** | **Tobacco consumption (Yes)** | **Alcohol consumption (Yes)** | **BMI (Overweight/Obese)** | **Central obesity (Present)** | **Blood glucose level (Raised)** |
| --- | --- | --- | --- | --- | --- | --- | --- | --- | --- | --- | --- | --- | --- |
| JAMMU & KASHMIR | H | L | NS | NS | NS | NS | H | NS | NS | NS | NS | NS | NS |
| HIMACHAL PRADESH | NS | L | NS | NS | NS | NS | NS | NS | NS | NS | NS | NS | NS |
| PUNJAB | NS | L | NS | NS | NS | NS | NS | L | NS | NS | NS | NS | L |
| CHANDIGARH | NS | L | NS | NS | NS | NS | NS | NA | NS | NS | NS | NS | NS |
| UTTARAKHAND | NS | NS | NS | NS | NS | NS | NS | NS | NS | NS | H | NS | NS |
| HARYANA | H | L | L | NS | NS | NS | NS | NS | NS | NS | H | NS | L |
| NCT OF DELHI | NS | L | NS | NS | NS | NS | NS | NS | NS | NS | H | NS | L |
| RAJASTHAN | H | L | L | NS | NS | NS | NS | NS | L | NS | NS | H | L |
| UTTAR PRADESH | H | L | L | NS | NS | NS | L | NS | L | NS | H | H | L |
| BIHAR | H | L | L | NS | NS | H | NS | NS | L | NS | H | NS | L |
| SIKKIM | NS | NS | NS | NS | NS | NS | NS | NS | NS | NS | NS | NS | NS |
| ARUNACHAL PRADESH | NS | NS | NS | NS | NS | NS | NS | NS | NS | NS | NS | NS | NS |
| NAGALAND | NS | NS | NS | NS | NS | NS | NS | NS | NS | NS | NS | NS | NS |
| MANIPUR | NS | L | NS | NS | NS | NS | NS | NS | NS | NS | NS | NS | NS |
| MIZORAM | NS | NS | NS | NS | NS | NS | NS | NS | NS | NS | NS | NS | NS |
| TRIPURA | NS | L | NS | NS | NS | NS | NS | NS | NS | NS | NS | L | NS |
| MEGHALAYA | NS | L | NS | NS | NS | NS | NS | NS | NS | NS | NS | NS | NS |
| ASSAM | H | L | NS | NS | NS | NS | NS | NS | NS | NS | H | NS | L |
| WEST BENGAL | H | L | NS | H | L | H | NS | H | H | NS | NS | NS | NS |
| JHARKHAND | H | L | NS | NS | NS | NS | NS | NS | NS | NS | H | L | NS |
| ODISHA | H | L | L | NS | NS | L | NS | NS | NS | NS | H | NS | L |
| CHHATTISGARH | NS | L | NS | H | NS | NS | NS | NS | NS | NS | H | NS | L |
| MADHYA PRADESH | H | L | L | NS | NS | NS | NS | L | NS | NS | H | NS | NS |
| GUJARAT | H | L | NS | NS | L | NS | NS | NS | L | NS | H | NS | L |
| DADRA & NAGAR HAVELI AND DAMAN & DIU | NS | NS | NS | NS | NS | NS | NS | NS | NS | NS | NS | NS | NS |
| MAHARASHTRA | NS | L | L | NS | NS | H | H | L | NS | NS | H | NS | L |
| ANDHRA PRADESH | H | L | L | NS | H | NS | NS | NS | NS | L | H | NS | NS |
| KARNATAKA | H | L | L | H | H | NS | L | H | L | NS | H | NS | L |
| GOA | NS | NS | L | NS | NS | NS | NS | NS | NS | NS | NS | NS | NS |
| LAKSHADWEEP | NS | NS | NS | NS | NS | NS | NS | NS | NS | NS | NS | NS | NS |
| KERALA | H | L | L | H | NS | NS | NS | L | L | L | NS | NS | L |
| TAMIL NADU | H | L | NS | NS | NS | NS | L | NS | NS | L | H | H | L |
| PUDUCHERRY | NS | NS | NS | NS | NS | NS | NS | NS | NS | NS | NS | NS | NS |
| ANDAMAN & NICOBAR ISLANDS | NS | NS | NS | NS | NS | NS | NS | NS | NS | NS | NS | NS | NS |
| TELANGANA | H | L | NS | NS | NS | NS | L | L | L | NS | H | NS | NS |
| LADAKH | NS | NS | NS | NS | NS | NS | NS | NS | NS | NS | NS | NS | NA |
| **H%** | 44.4 | 0.0 | 0.0 | 11.1 | 5.6 | 8.3 | 5.6 | 5.6 | 2.8 | 0.0 | 44.4 | 8.3 | 0.0 |
| **L%** | 0.0 | 69.4 | 30.6 | 0.0 | 5.6 | 2.8 | 11.1 | 13.9 | 19.4 | 8.3 | 0.0 | 5.6 | 38.9 |

*H: Higher odds [Adjusted odds ratio(AOR) >1 and P<0.05], L: Lower odds (AOR<1 and P<0.05), NS: Not Significant (p>0.05), NA:* *Data Not Available.*

**Table S9: State wise determinants of raised blood pressure among adults in India(AOR) (National Family Health Survey-5, India, 2019-2021)**

|  | Age Group (>=30) | Sex (Female) | Education (Literate) | Occupation (Employed) | Household wealth quintile (middle) | Household wealth quintile (richer/richest) | Marital status (Currently married) | Place Of Residence (Urban) | Tobacco consumption (Yes) | Alcohol consumption (Yes) | BMI (Overweight/Obese) | Central obesity (Present) | Blood glucose level (Raised) |
| --- | --- | --- | --- | --- | --- | --- | --- | --- | --- | --- | --- | --- | --- |
| JAMMU & KASHMIR | H | NS | NS | NS | NS | NS | H | NS | NS | NS | H | H | H |
| HIMACHAL PRADESH | H | L | NS | NS | NS | NS | NS | NS | NS | NS | NS | H | NS |
| PUNJAB | H | L | L | NS | NS | NS | NS | L | NS | NS | H | H | H |
| CHANDIGARH | NS | NS | NS | NS | NS | NS | NS | NS | NS | NS | NS | NS | NS |
| UTTARAKHAND | H | L | NS | NS | NS | NS | NS | NS | NS | NS | H | NS | NS |
| HARYANA | H | L | NS | NS | NS | NS | NS | NS | NS | NS | H | H | H |
| NCT OF DELHI | H | L | NS | NS | NS | NS | NS | NS | NS | NS | NS | H | H |
| RAJASTHAN | H | NS | NS | NS | H | H | H | H | NS | NS | H | H | H |
| UTTAR PRADESH | H | L | L | L | H | H | H | NS | NS | NS | H | H | H |
| BIHAR | H | NS | NS | NS | H | H | H | H | NS | NS | H | H | H |
| SIKKIM | NS | NS | NS | NS | NS | NS | NS | NS | NS | NS | NS | NS | NS |
| ARUNACHAL PRADESH | NS | NS | NS | NS | NS | NS | NS | NS | NS | NS | NS | NS | NS |
| NAGALAND | H | NS | NS | NS | NS | NS | NS | NS | NS | NS | NS | NS | NS |
| MANIPUR | H | L | NS | NS | NS | NS | NS | NS | NS | NS | NS | H | NS |
| MIZORAM | NS | NS | NS | NS | NS | NS | NS | NS | NS | NS | NS | NS | NS |
| TRIPURA | H | NS | NS | NS | NS | NS | NS | NS | NS | NS | H | H | NS |
| MEGHALAYA | H | NS | NS | NS | NS | NS | NS | NS | NS | H | NS | NS | NS |
| ASSAM | H | L | NS | NS | NS | NS | H | NS | NS | NS | H | H | H |
| WEST BENGAL | H | NS | L | NS | H | NS | NS | NS | NS | H | H | H | H |
| JHARGHAND | H | L | NS | NS | NS | NS | NS | NS | NS | NS | H | H | NS |
| ODISHA | H | NS | L | NS | NS | NS | NS | NS | NS | H | H | H | H |
| CHATTISGARH | H | L | NS | NS | NS | NS | NS | NS | NS | NS | H | H | H |
| MADHYA PRADESH | H | L | L | NS | NS | H | NS | NS | NS | NS | H | H | H |
| GUJARAT | H | L | NS | H | NS | NS | H | L | NS | H | H | H | H |
| DADRA & NAGAR HAVELI AND DAMAN & DIU | NS | NS | NS | NS | NS | NS | NS | NS | NS | NS | NS | NS | NS |
| MAHARASHTRA | H | L | L | NS | H | NS | H | H | H | H | H | H | H |
| ANDRA PRADESH | H | L | NS | L | L | L | NS | L | NS | H | H | H | H |
| KARNATAKA | H | L | L | L | NS | NS | H | NS | NS | H | H | H | H |
| GOA | H | NS | NS | NS | NS | NS | NS | NS | NS | NS | NS | NS | H |
| LAKSHADWEEP | NS | NS | NS | NS | NS | NS | NS | NS | NS | NS | NS | NS | NS |
| KERALA | H | L | NS | L | L | L | NS | NS | NS | H | H | H | H |
| TAMILNADU | H | L | L | L | NS | NS | NS | NS | L | H | H | H | H |
| PUDUCHERRY | NS | NS | NS | NS | NS | NS | NS | NS | NS | NS | NS | NS | NS |
| ANDAMAN & NICOBAR ISLANDS | NS | NS | NS | NS | NS | NS | NS | NS | NS | NS | NS | NS | NS |
| TELANGANA | H | L | NS | NS | NS | NS | NS | H | NS | H | H | H | H |
| LADAKH | NS | NS | NS | NS | NS | NS | NS | NS | NS | NS | NS | NS | NA |
| **H%** | **75.0** | **0.0** | **0.0** | **2.8** | **13.9** | **11.1** | **22.2** | **11.1** | **2.8** | **27.8** | **58.3** | **63.9** | **55.6** |
| **L%** | **0.0** | **50.0** | **22.2** | **13.9** | **5.6** | **5.6** | **0.0** | **8.3** | **2.8** | **0.0** | **0.0** | **0.0** | **0.0** |

*H: Higher odds [Adjusted odds ratio(AOR) >1 and P<0.05], L: Lower odds (AOR<1 and P<0.05), NS: Not Significant (p>0.05), NA:* *Data Not Available.*

## **Table S10: District wise determinants of Ever measured blood pressure among adults in India (AOR) (National Family Health Survey-5, India, 2019-2021)**

| **State Name** | **District Name** | Age Group (>=30) | Sex (Female) | Education (Literate) | Occupation (Employed) | Household wealth quintile (middle) | Household wealth quintile (richer/richest) | Marital status (Currently married) | Place Of Residence (Urban) | Tobacco consumption (Yes) | Alcohol consumption (Yes) | BMI (Overweight/Obese) | Central obesity (Present) | Blood glucose level (Raised) |
| --- | --- | --- | --- | --- | --- | --- | --- | --- | --- | --- | --- | --- | --- | --- |
| ANDHRA PRADESH | Srikakulam | NS | NS | NS | NS | NS | NS | NS | NS | NS | NS | NS | NS | NS |
| ANDHRA PRADESH | Vizianagaram | NS | NS | NS | NS | NS | NS | NS | NS | NS | NS | NS | NS | NS |
| ANDHRA PRADESH | Visakhapatnam | NS | NS | H | H | NS | NS | NS | L | NS | NS | NS | NS | NS |
| ANDHRA PRADESH | East Godavari | H | NS | H | NS | H | NS | NS | NS | NS | L | NS | H | L |
| ANDHRA PRADESH | West Godavari | H | NS | NS | NS | L | NS | H | NS | NS | NS | NS | NS | NS |
| ANDHRA PRADESH | Krishna | H | H | NS | NS | NS | NS | NS | NS | NS | NS | NS | NS | NS |
| ANDHRA PRADESH | Guntur | H | NS | NS | L | NS | NS | NS | H | NS | NS | NS | NS | NS |
| ANDHRA PRADESH | Prakasam | NS | NS | NS | NS | NS | NS | H | NS | L | NS | NS | H | NS |
| ANDHRA PRADESH | Sri Potti Sriramulu Nellore | NS | NS | NS | NS | NS | NS | NS | NS | NS | NS | NS | NS | NS |
| ANDHRA PRADESH | Y.S.R. | H | NS | NS | NS | NS | NS | NS | NS | NS | NS | NS | H | NS |
| ANDHRA PRADESH | Kurnool | NS | H | NS | NS | NS | H | H | NS | NS | L | L | H | NS |
| ANDHRA PRADESH | Anantapur | H | H | NS | NS | NS | NS | H | NS | NS | NS | NS | NS | NS |
| ANDHRA PRADESH | Chittoor | NS | NS | NS | NS | NS | H | H | H | NS | NS | NS | NS | NS |
| ASSAM | Kokrajhar | NS | NS | NS | NS | NS | NS | NS | NS | NS | NS | NS | NS | NS |
| ASSAM | Goalpara | NS | NS | NS | NS | NS | NS | NS | NS | NS | NS | NS | NS | NS |
| ASSAM | Barpeta | NS | NS | NS | NS | NS | NS | NS | NS | NS | NS | H | NS | NS |
| ASSAM | Morigaon | NS | NS | NS | NS | NS | NS | NS | NS | NS | NS | NS | NS | NS |
| ASSAM | Lakhimpur | NS | NS | NS | NS | NS | NS | NS | NS | NS | NS | NS | NS | NS |
| ASSAM | Dhemaji | NS | NS | NS | NS | NS | NS | NS | NS | NS | NS | NS | NS | NS |
| ASSAM | Tinsukia | NS | NS | NS | NS | NS | NS | NS | NS | NS | NS | NS | NS | NS |
| ASSAM | Dibrugarh | H | NS | NS | NS | H | NS | NS | H | NS | NS | H | NS | NS |
| ASSAM | Golaghat | H | NS | NS | NS | NS | NS | NS | NS | NS | NS | NS | NS | NS |
| ASSAM | Dima Hasao | NS | NS | NS | NS | NS | NS | NS | NS | NS | NS | NS | NS | NS |
| ASSAM | Cachar | NS | NS | NS | NS | NS | NS | NS | NS | NS | NS | H | NS | NS |
| ASSAM | Karimganj | H | NS | NS | NS | NS | NS | NS | NS | NS | NS | NS | NS | NS |
| ASSAM | Hailakandi | NS | NS | NS | NS | NS | NS | H | NS | NS | NS | NS | NS | NS |
| ASSAM | Bongaigaon | NS | NS | NS | NS | NS | NS | NS | NS | NS | NS | NS | NS | NS |
| ASSAM | Chirang | NS | NS | NS | NS | NS | NS | NS | NS | NS | NS | NS | NS | NS |
| ASSAM | Kamrup | H | NS | NS | NS | NS | NS | NS | NS | NS | NS | NS | NS | NS |
| ASSAM | Kamrup Metropolitan | NS | NS | NS | H | NS | NS | NS | NS | NS | NS | NS | NS | NS |
| ASSAM | Nalbari | NS | NS | NS | NS | NS | NS | H | NS | NS | NS | NS | NS | NS |
| ASSAM | Baksa | NS | NS | NS | NS | NS | NS | NS | NS | NS | NS | NS | NS | NS |
| ASSAM | Darrang | NS | NS | NS | NS | NS | NS | NS | NS | NS | NS | NS | NS | NS |
| ASSAM | Udalguri | NS | NS | NS | NS | NS | NS | H | NS | NS | NS | NS | NS | NS |
| ASSAM | Biswanath | NS | NS | NS | NS | NS | NS | NS | NS | NS | NS | NS | NS | NS |
| ASSAM | Charaideo | NS | NS | NS | NS | NS | NS | NS | NS | NS | NS | NS | NS | NS |
| ASSAM | Dhubri | NS | NS | NS | NS | NS | NS | H | NS | NS | NS | NS | NS | NS |
| ASSAM | Hojai | NS | NS | NS | NS | NS | NS | NS | NS | NS | NS | NS | NS | NS |
| ASSAM | Jorhat | H | NS | NS | NS | NS | NS | NS | NS | NS | NS | NS | NS | NS |
| ASSAM | Karbi Anglong | NS | NS | NS | NS | NS | NS | NS | NS | NS | NS | NS | NS | NS |
| ASSAM | Majuli | NS | NS | NS | NS | NS | NS | NS | NS | NS | NS | NS | NS | NS |
| ASSAM | Nagaon | NS | NS | NS | NS | NS | H | NS | NS | H | NS | NS | NS | NS |
| ASSAM | Sivasagar | NS | NS | NS | NS | NS | NS | NS | NS | NS | NS | NS | NS | NS |
| ASSAM | Sonitpur | NS | H | NS | NS | NS | NS | NS | NS | NS | NS | NS | NS | NS |
| ASSAM | South Salmara Mancachar | NS | NS | NS | NS | NS | NS | NS | NS | NS | NS | NS | NS | NS |
| ASSAM | West Karbi Anglong | NS | NS | NS | NS | NS | NS | NS | NS | NS | NS | NS | NS | NS |
| BIHAR | Pashchim Champaran | NS | NS | NS | H | NS | NS | NS | NS | NS | NS | NS | NS | NS |
| BIHAR | Purba Champaran | NS | H | NS | NS | NS | NS | H | NS | NS | NS | NS | NS | NS |
| BIHAR | Sheohar | NS | NS | NS | NS | NS | NS | H | NS | NS | NS | NS | NS | NS |
| BIHAR | Sitamarhi | NS | NS | NS | NS | NS | NS | NS | H | H | NS | NS | NS | NS |
| BIHAR | Madhubani | NS | H | NS | NS | NS | NS | L | H | NS | NS | NS | NS | NS |
| BIHAR | Supaul | NS | NS | NS | NS | NS | NS | H | NS | NS | NS | NS | NS | NS |
| BIHAR | Araria | L | NS | H | NS | NS | NS | NS | H | NS | NS | NS | NS | NS |
| BIHAR | Kishanganj | NS | NS | NS | NS | NS | NS | NS | NS | NS | NS | NS | NS | NS |
| BIHAR | Purnia | NS | H | NS | NS | NS | NS | NS | H | L | NS | H | NS | NS |
| BIHAR | Katihar | H | H | NS | NS | NS | NS | NS | NS | NS | NS | NS | NS | NS |
| BIHAR | Madhepura | NS | NS | NS | NS | NS | NS | NS | NS | NS | NS | NS | NS | NS |
| BIHAR | Saharsa | NS | NS | NS | NS | NS | NS | NS | NS | NS | NS | NS | NS | NS |
| BIHAR | Darbhanga | NS | H | H | NS | H | NS | NS | H | H | NS | H | NS | NS |
| BIHAR | Muzaffarpur | NS | NS | H | NS | NS | NS | NS | H | H | NS | NS | NS | NS |
| BIHAR | Gopalganj | NS | NS | NS | NS | NS | NS | NS | H | H | NS | NS | NS | NS |
| BIHAR | Siwan | H | NS | NS | NS | NS | NS | NS | H | NS | NS | NS | NS | NS |
| BIHAR | Saran | NS | NS | NS | NS | H | NS | NS | H | NS | NS | NS | NS | NS |
| BIHAR | Vaishali | H | H | NS | NS | H | NS | NS | H | NS | NS | NS | NS | NS |
| BIHAR | Samastipur | NS | NS | NS | NS | NS | NS | NS | NS | H | NS | NS | NS | NS |
| BIHAR | Begusarai | H | H | NS | NS | NS | NS | NS | NS | L | NS | NS | NS | NS |
| BIHAR | Khagaria | NS | NS | NS | NS | NS | NS | NS | H | NS | NS | NS | NS | NS |
| BIHAR | Bhagalpur | NS | NS | NS | NS | L | NS | NS | H | NS | NS | NS | NS | NS |
| BIHAR | Banka | H | H | NS | NS | NS | NS | NS | NS | NS | NS | NS | NS | NS |
| BIHAR | Munger | NS | NS | NS | NS | NS | NS | NS | H | H | NS | NS | NS | NS |
| BIHAR | Lakhisarai | NS | NS | NS | NS | NS | NS | NS | NS | NS | NS | NS | NS | NS |
| BIHAR | Sheikhpura | NS | NS | NS | NS | NS | NS | NS | NS | NS | NS | NS | NS | NS |
| BIHAR | Nalanda | NS | NS | NS | NS | NS | NS | NS | H | L | NS | NS | NS | NS |
| BIHAR | Patna | NS | NS | L | H | H | NS | NS | H | H | H | NS | NS | NS |
| BIHAR | Bhojpur | NS | NS | NS | NS | NS | NS | NS | H | H | NS | NS | NS | NS |
| BIHAR | Buxar | H | NS | NS | NS | NS | NS | NS | NS | NS | NS | NS | NS | NS |
| BIHAR | Kaimur (Bhabua) | NS | H | NS | NS | NS | NS | NS | H | NS | NS | NS | NS | NS |
| BIHAR | Rohtas | H | NS | NS | NS | NS | NS | NS | H | H | NS | NS | NS | NS |
| BIHAR | Aurangabad | NS | NS | NS | NS | NS | NS | NS | NS | NS | NS | NS | NS | NS |
| BIHAR | Gaya | NS | NS | NS | NS | NS | NS | NS | H | NS | L | NS | H | NS |
| BIHAR | Nawada | NS | H | NS | NS | NS | NS | NS | NS | H | NS | NS | NS | NS |
| BIHAR | Jamui | NS | NS | NS | NS | NS | NS | NS | NS | NS | NS | NS | NS | NS |
| BIHAR | Jehanabad | H | NS | NS | NS | NS | NS | NS | H | NS | NS | NS | NS | NS |
| BIHAR | Arwal | NS | NS | NS | NS | NS | NS | NS | NS | NS | NS | NS | NS | NS |
| CHHATTISGARH | Koriya | NS | NS | NS | NS | NS | NS | NS | NS | NS | NS | NS | NS | NS |
| CHHATTISGARH | Jashpur | NS | NS | NS | NS | NS | NS | NS | NS | NS | NS | NS | NS | NS |
| CHHATTISGARH | Raigarh | NS | H | NS | NS | NS | NS | H | NS | NS | H | NS | NS | NS |
| CHHATTISGARH | Korba | NS | NS | NS | NS | NS | NS | NS | NS | NS | NS | NS | NS | NS |
| CHHATTISGARH | Janjgir - Champa | NS | NS | NS | NS | NS | NS | H | NS | NS | NS | NS | NS | NS |
| CHHATTISGARH | Kabeerdham | NS | NS | NS | NS | NS | NS | NS | NS | NS | NS | NS | NS | NS |
| CHHATTISGARH | Rajnandgaon | NS | NS | NS | NS | NS | NS | H | NS | NS | NS | NS | NS | NS |
| CHHATTISGARH | Mahasamund | NS | NS | NS | NS | NS | NS | H | NS | NS | NS | NS | NS | NS |
| CHHATTISGARH | Dhamtari | NS | NS | NS | NS | NS | NS | NS | NS | NS | NS | NS | NS | NS |
| CHHATTISGARH | Uttar Bastar Kanker | NS | NS | NS | NS | NS | NS | NS | NS | NS | NS | NS | NS | NS |
| CHHATTISGARH | Narayanpur | NS | NS | NS | NS | NS | NS | NS | NS | NS | NS | NS | NS | NS |
| CHHATTISGARH | Bijapur | NS | NS | NS | NS | NS | NS | NS | NS | NS | NS | NS | NS | NS |
| CHHATTISGARH | Balod | NS | NS | NS | NS | NS | NS | NS | NS | NS | NS | NS | NS | NS |
| CHHATTISGARH | Baloda Bazar | NS | NS | NS | NS | NS | NS | NS | NS | NS | NS | NS | NS | NS |
| CHHATTISGARH | Balrampur | NS | NS | NS | NS | NS | NS | NS | NS | NS | NS | NS | NS | NS |
| CHHATTISGARH | Bastar | NS | NS | NS | NS | NS | NS | NS | NS | NS | NS | NS | NS | NS |
| CHHATTISGARH | Bemetara | NS | NS | NS | NS | NS | NS | H | NS | NS | NS | NS | NS | NS |
| CHHATTISGARH | Bilaspur | NS | H | NS | NS | NS | NS | NS | NS | NS | NS | NS | NS | NS |
| CHHATTISGARH | Dantewada | NS | NS | NS | NS | NS | NS | NS | NS | NS | NS | NS | NS | NS |
| CHHATTISGARH | Durg | NS | NS | NS | NS | NS | NS | NS | NS | NS | NS | NS | NS | NS |
| CHHATTISGARH | Gariyaband | NS | NS | NS | NS | NS | NS | NS | NS | NS | NS | NS | NS | NS |
| CHHATTISGARH | Kodagaon | NS | NS | NS | NS | NS | NS | NS | NS | NS | NS | NS | NS | NS |
| CHHATTISGARH | Mungeli | NS | NS | NS | NS | NS | NS | NS | NS | NS | NS | NS | NS | NS |
| CHHATTISGARH | Raipur | NS | NS | NS | NS | H | H | H | NS | NS | NS | NS | NS | NS |
| CHHATTISGARH | Sukma | NS | NS | NS | NS | NS | NS | NS | NS | NS | NS | NS | NS | NS |
| CHHATTISGARH | Surajpur | NS | NS | NS | NS | NS | NS | NS | NS | NS | NS | NS | NS | NS |
| CHHATTISGARH | Surguja | NS | NS | NS | NS | NS | NS | NS | NS | NS | NS | NS | NS | NS |
| GOA | North Goa | NS | NS | NS | NS | NS | NS | NS | NS | NS | NS | NS | NS | NS |
| GOA | South Goa | NS | NS | NS | NS | NS | NS | NS | NS | NS | NS | NS | NS | NS |
| GUJARAT | Kachchh | H | H | NS | NS | NS | NS | H | NS | NS | NS | NS | NS | NS |
| GUJARAT | Banas Kantha | NS | H | H | NS | H | H | H | NS | NS | NS | NS | NS | NS |
| GUJARAT | Patan | H | NS | NS | NS | NS | NS | NS | NS | NS | NS | NS | NS | NS |
| GUJARAT | Mahesana | NS | H | H | NS | NS | NS | NS | NS | NS | NS | NS | NS | NS |
| GUJARAT | Gandhinagar | NS | H | NS | NS | NS | H | NS | NS | NS | NS | H | NS | NS |
| GUJARAT | Porbandar | NS | NS | NS | NS | NS | NS | NS | H | NS | NS |  | NS | NS |
| GUJARAT | Amreli | NS | NS | NS | NS | NS | NS | NS | NS | NS | NS | NS | NS | NS |
| GUJARAT | Anand | H | H | NS | NS | NS | NS | NS | NS | NS | NS | NS | NS | NS |
| GUJARAT | Dohad | NS | NS | NS | NS | NS | NS | NS | NS | NS | NS | NS | NS | NS |
| GUJARAT | Narmada | NS | NS | NS | NS | NS | NS | NS | NS | NS | NS | NS | NS | NS |
| GUJARAT | Bharuch | NS | NS | NS | NS | NS | NS | NS | NS | NS | NS | NS | NS | NS |
| GUJARAT | The Dangs | NS | NS | NS | NS | NS | NS | NS | NS | NS | NS | NS | NS | NS |
| GUJARAT | Navsari | NS | NS | NS | NS | NS | NS | NS | NS | NS | NS | NS | NS | NS |
| GUJARAT | Valsad | NS | H | NS | NS | H | H | NS | H | NS | NS | NS | NS | NS |
| GUJARAT | Surat | NS | NS | NS | H | NS | NS | H | NS | H | H | NS | NS | NS |
| GUJARAT | Tapi | NS | NS | NS | NS | NS | NS | NS | NS | NS | NS | NS | NS | NS |
| GUJARAT | Ahmadabad | H | NS | H | NS | NS | NS | NS | NS | H | H | H | H | NS |
| GUJARAT | Aravali | NS | H | NS | NS | NS | NS | NS | NS | NS | NS | NS | NS | NS |
| GUJARAT | Bhavnagar | NS | NS | NS | NS | NS | NS | NS | H | NS | NS | NS | NS | NS |
| GUJARAT | Botad | NS | NS | NS | NS | NS | NS | NS | NS | NS | NS | NS | NS | NS |
| GUJARAT | Chhota Udaipur | NS | NS | NS | NS | NS | NS | NS | NS | NS | NS | NS | NS | NS |
| GUJARAT | Devbhumi Dwarka | NS | NS | NS | NS | NS | NS | NS | NS | NS | NS | NS | NS | NS |
| GUJARAT | Gir Somnath | NS | NS | NS | NS | NS | NS | H | NS | NS | NS | NS | NS | NS |
| GUJARAT | Jamnagar | NS | NS | NS | NS | NS | NS | H | H | NS | NS | NS | NS | NS |
| GUJARAT | Junagadh | NS | H | NS | NS | NS | NS | NS | NS | NS | NS | NS | NS | NS |
| GUJARAT | Kheda | NS | NS | NS | NS | H | H | NS | NS | NS | NS | NS | NS | NS |
| GUJARAT | Mahisagar | NS | NS | NS | NS | NS | NS | NS | NS | NS | NS | NS | NS | NS |
| GUJARAT | Morbi | NS | NS | NS | NS | NS | NS | NS | NS | NS | NS | NS | NS | NS |
| GUJARAT | Panch Mahals | NS | H | NS | NS | NS | NS | H | H | NS | NS | H | NS | NS |
| GUJARAT | Rajkot | NS | H | NS | H | NS | NS | H | NS | NS | NS | NS | NS | NS |
| GUJARAT | Sabar Kantha | NS | NS | NS | NS | NS | NS | NS | NS | NS | NS | NS | NS | NS |
| GUJARAT | Surendranagar | NS | NS | NS | NS | NS | NS | NS | NS | NS | NS | NS | NS | NS |
| GUJARAT | Vadodara | NS | NS | NS | NS | L | NS | NS | NS | NS | NS | NS | H | NS |
| HARYANA | Panchkula | NS | NS | NS | NS | NS | NS | NS | NS | NS | NS | NS | NS | NS |
| HARYANA | Ambala | NS | NS | NS | NS | NS | NS | NS | NS | NS | NS | NS | NS | NS |
| HARYANA | Yamunanagar | NS | NS | NS | NS | NS | NS | NS | NS | NS | NS | NS | NS | NS |
| HARYANA | Kurukshetra | NS | NS | NS | NS | NS | NS | NS | NS | NS | NS | NS | NS | NS |
| HARYANA | Kaithal | NS | NS | NS | NS | NS | NS | NS | NS | NS | NS | NS | NS | NS |
| HARYANA | Karnal | NS | H | NS | NS | NS | NS | NS | NS | NS | NS | NS | NS | NS |
| HARYANA | Panipat | NS | NS | NS | NS | NS | NS | NS | NS | NS | NS | NS | NS | NS |
| HARYANA | Sonipat | NS | NS | NS | NS | NS | NS | H | NS | NS | NS | NS | NS | NS |
| HARYANA | Jind | NS | NS | NS | NS | NS | NS | H | NS | NS | NS | NS | NS | NS |
| HARYANA | Fatehabad | NS | NS | NS | NS | NS | NS | NS | NS | NS | NS | NS | NS | NS |
| HARYANA | Sirsa | NS | NS | NS | NS | NS | NS | NS | NS | NS | NS | NS | NS | NS |
| HARYANA | Hisar | NS | NS | NS | NS | NS | NS | H | NS | NS | NS | NS | NS | NS |
| HARYANA | Rohtak | NS | NS | NS | NS | NS | NS | NS | NS | NS | NS | NS | NS | NS |
| HARYANA | Jhajjar | NS | NS | NS | NS | NS | NS | NS | NS | NS | NS | NS | NS | NS |
| HARYANA | Mahendragarh | NS | NS | NS | NS | NS | NS | NS | NS | NS | NS | NS | NS | NS |
| HARYANA | Rewari | NS | NS | NS | NS | NS | NS | NS | NS | NS | NS | NS | NS | NS |
| HARYANA | Gurgaon | NS | NS | NS | NS | NS | NS | NS | NS | NS | NS | NS | NS | NS |
| HARYANA | Mewat | NS | NS | NS | NS | NS | NS | NS | NS | NS | NS | NS | NS | NS |
| HARYANA | Faridabad | NS | NS | NS | NS | NS | NS | NS | NS | NS | NS | NS | NS | NS |
| HARYANA | Palwal | NS | NS | NS | NS | NS | NS | H | NS | NS | NS | NS | NS | NS |
| HARYANA | Bhiwani | NS | NS | NS | NS | NS | NS | H | NS | NS | NS | NS | NS | NS |
| HARYANA | Charkhi Dadri | NS | NS | NS | NS | NS | NS | NS | NS | NS | NS | NS | NS | NS |
| HIMACHAL PRADESH | Chamba | NS | NS | NS | NS | NS | NS | NS | NS | NS | NS | NS | NS | NS |
| HIMACHAL PRADESH | Kangra | NS | NS | NS | NS | NS | NS | H | NS | NS | NS | NS | NS | NS |
| HIMACHAL PRADESH | Lahul & Spiti | NS | NS | NS | NS | NS | NS | NS | NS | NS | NS | NS | NS | NS |
| HIMACHAL PRADESH | Kullu | NS | NS | NS | NS | NS | NS | NS | NS | NS | NS | NS | NS | NS |
| HIMACHAL PRADESH | Mandi | NS | NS | NS | NS | NS | NS | NS | NS | NS | NS | NS | NS | NS |
| HIMACHAL PRADESH | Hamirpur | NS | NS | NS | NS | NS | NS | NS | NS | NS | NS | NS | NS | NS |
| HIMACHAL PRADESH | Una | NS | NS | NS | NS | NS | NS | NS | NS | NS | NS | NS | NS | NS |
| HIMACHAL PRADESH | Bilaspur | NS | NS | NS | NS | NS | NS | NS | NS | NS | NS | NS | NS | NS |
| HIMACHAL PRADESH | Solan | NS | NS | NS | NS | NS | NS | NS | NS | NS | NS | NS | NS | NS |
| HIMACHAL PRADESH | Sirmaur | NS | NS | NS | NS | NS | NS | NS | NS | NS | NS | NS | NS | NS |
| HIMACHAL PRADESH | Shimla | NS | NS | NS | NS | NS | NS | NS | NS | NS | NS | NS | NS | NS |
| HIMACHAL PRADESH | Kinnaur | NS | NS | NS | NS | NS | NS | NS | NS | NS | NS | NS | NS | NS |
| JAMMU & KASHMIR | Kupwara | NS | NS | NS | NS | NS | NS | NS | NS | NS | NS | NS | NS | NS |
| JAMMU & KASHMIR | Badgam | NS | NS | NS | NS | NS | NS | NS | NS | NS | NS | NS | NS | NS |
| JAMMU & KASHMIR | Punch | NS | NS | NS | NS | NS | NS | NS | NS | NS | NS | NS | NS | NS |
| JAMMU & KASHMIR | Rajouri | NS | NS | NS | NS | NS | NS | NS | NS | NS | NS | NS | NS | NS |
| JAMMU & KASHMIR | Kathua | NS | NS | NS | NS | NS | NS | NS | NS | NS | NS | NS | NS | NS |
| JAMMU & KASHMIR | Baramula | NS | NS | NS | NS | NS | NS | NS | NS | NS | NS | NS | NS | NS |
| JAMMU & KASHMIR | Bandipore | NS | NS | NS | NS | NS | NS | NS | NS | NS | NS | NS | NS | NS |
| JAMMU & KASHMIR | Srinagar | NS | NS | NS | NS | NS | NS | H | NS | NS | NS | NS | NS | NS |
| JAMMU & KASHMIR | Ganderbal | NS | NS | NS | NS | NS | NS | NS | NS | NS | NS | NS | NS | NS |
| JAMMU & KASHMIR | Pulwama | NS | NS | NS | NS | NS | NS | NS | NS | NS | NS | NS | NS | NS |
| JAMMU & KASHMIR | Shupiyan | NS | NS | NS | NS | NS | NS | NS | NS | NS | NS | NS | NS | NS |
| JAMMU & KASHMIR | Anantnag | NS | NS | NS | NS | H | NS | NS | NS | NS | NS | NS | NS | NS |
| JAMMU & KASHMIR | Kulgam | NS | NS | NS | NS | NS | NS | NS | NS | NS | NS | NS | NS | NS |
| JAMMU & KASHMIR | Doda | NS | NS | NS | NS | NS | NS | NS | NS | NS | NS | NS | NS | NS |
| JAMMU & KASHMIR | Ramban | NS | NS | NS | NS | NS | NS | NS | NS | NS | NS | NS | NS | NS |
| JAMMU & KASHMIR | Kishtwar | NS | NS | NS | NS | NS | NS | NS | NS | NS | NS | NS | NS | NS |
| JAMMU & KASHMIR | Udhampur | NS | NS | NS | NS | NS | NS | NS | NS | NS | NS | NS | NS | NS |
| JAMMU & KASHMIR | Reasi | NS | NS | NS | NS | NS | NS | NS | NS | NS | NS | NS | NS | NS |
| JAMMU & KASHMIR | Jammu | NS | NS | NS | NS | NS | NS | NS | NS | NS | NS | NS | NS | NS |
| JAMMU & KASHMIR | Samba | NS | NS | NS | NS | NS | NS | NS | NS | NS | NS | NS | NS | NS |
| JHARKHAND | Garhwa | NS | NS | NS | NS | NS | NS | H | NS | NS | NS | NS | NS | NS |
| JHARKHAND | Chatra | NS | NS | NS | NS | NS | NS | NS | NS | NS | NS | NS | NS | NS |
| JHARKHAND | Kodarma | NS | NS | NS | NS | NS | NS | NS | NS | NS | NS | NS | NS | NS |
| JHARKHAND | Giridih | NS | NS | NS | NS | NS | NS | NS | NS | NS | NS | NS | NS | NS |
| JHARKHAND | Deoghar | NS | NS | NS | NS | NS | NS | NS | NS | NS | NS | NS | NS | NS |
| JHARKHAND | Godda | NS | NS | NS | NS | NS | NS | NS | NS | NS | NS | NS | NS | NS |
| JHARKHAND | Sahibganj | NS | NS | NS | NS | NS | NS | NS | NS | NS | NS | NS | NS | NS |
| JHARKHAND | Pakur | NS | NS | NS | NS | NS | NS | NS | NS | NS | NS | NS | NS | NS |
| JHARKHAND | Dhanbad | H | NS | NS | NS | NS | NS | NS | NS | NS | NS | NS | NS | NS |
| JHARKHAND | Bokaro | NS | NS | NS | NS | NS | NS | H | NS | NS | NS | NS | NS | NS |
| JHARKHAND | Lohardaga | NS | NS | NS | NS | NS | NS | NS | NS | NS | NS | NS | NS | NS |
| JHARKHAND | Purbi Singhbhum | NS | NS | NS | NS | NS | NS | NS | NS | NS | NS | NS | NS | NS |
| JHARKHAND | Palamu | NS | NS | NS | NS | NS | NS | H | NS | NS | NS | NS | NS | NS |
| JHARKHAND | Latehar | NS | NS | NS | NS | NS | NS | NS | NS | NS | NS | NS | NS | NS |
| JHARKHAND | Hazaribagh | NS | NS | NS | NS | NS | NS | NS | NS | NS | NS | NS | NS | NS |
| JHARKHAND | Ramgarh | NS | NS | NS | NS | NS | NS | NS | H | NS | NS | NS | NS | NS |
| JHARKHAND | Dumka | NS | NS | H | NS | NS | NS | NS | NS | NS | NS | NS | NS | NS |
| JHARKHAND | Jamtara | NS | NS | NS | NS | NS | NS | NS | NS | NS | NS | NS | NS | NS |
| JHARKHAND | Ranchi | NS | NS | NS | NS | NS | NS | NS | NS | NS | NS | NS | NS | NS |
| JHARKHAND | Khunti | NS | NS | NS | NS | NS | NS | NS | NS | NS | NS | NS | NS | NS |
| JHARKHAND | Gumla | NS | NS | NS | NS | NS | NS | NS | NS | NS | NS | NS | NS | NS |
| JHARKHAND | Simdega | NS | NS | NS | NS | NS | NS | NS | NS | NS | NS | NS | NS | NS |
| JHARKHAND | Pashchimi Singhbhum | NS | NS | NS | NS | NS | NS | NS | NS | NS | NS | NS | NS | NS |
| JHARKHAND | Saraikela-Kharsawan | H | NS | NS | NS | NS | NS | NS | NS | NS | L | NS | NS | NS |
| KARNATAKA | Belgaum | NS | NS | NS | NS | NS | NS | H | H | NS | NS | NS | NS | NS |
| KARNATAKA | Bagalkot | NS | NS | NS | NS | NS | NS | H | L | NS | NS | NS | NS | NS |
| KARNATAKA | Bijapur | NS | H | H | NS | NS | NS | NS | NS | NS | NS | NS | NS | NS |
| KARNATAKA | Bidar | NS | NS | NS | NS | NS | NS | NS | NS | NS | NS | NS | NS | NS |
| KARNATAKA | Raichur | NS | NS | NS | NS | NS | NS | NS | NS | NS | NS | NS | NS | NS |
| KARNATAKA | Koppal | NS | H | NS | NS | NS | NS | NS | NS | NS | NS | NS | NS | NS |
| KARNATAKA | Gadag | NS | NS | NS | NS | NS | NS | NS | NS | NS | NS | NS | NS | NS |
| KARNATAKA | Dharwad | H | NS | NS | NS | NS | H | NS | NS | NS | L | NS | NS | NS |
| KARNATAKA | Uttara Kannada | NS | NS | NS | NS | NS | NS | NS | H | NS | H | NS | NS | NS |
| KARNATAKA | Haveri | NS | NS | NS | NS | NS | NS | H | NS | NS | NS | NS | NS | NS |
| KARNATAKA | Bellary | NS | H | NS | NS | NS | NS | NS | NS | H | NS | H | NS | NS |
| KARNATAKA | Chitradurga | NS | NS | NS | NS | NS | NS | NS | NS | NS | NS | NS | NS | NS |
| KARNATAKA | Davanagere | NS | H | NS | NS | NS | NS | NS | NS | NS | NS | NS | H | NS |
| KARNATAKA | Shimoga | NS | H | NS | H | NS | NS | NS | NS | NS | NS | NS | NS | NS |
| KARNATAKA | Udupi | H | H | NS | NS | NS | NS | NS | NS | NS | NS | NS | NS | NS |
| KARNATAKA | Chikmagalur | NS | NS | NS | NS | L | NS | H | NS | NS | NS | NS | H | NS |
| KARNATAKA | Tumkur | NS | NS | NS | NS | NS | NS | H | H | NS | NS | NS | NS | NS |
| KARNATAKA | Bangalore | NS | NS | NS | NS | NS | NS | H | H | NS | NS | L | H | L |
| KARNATAKA | Mandya | NS | NS | NS | L | L | L | H | NS | NS | NS | NS | NS | NS |
| KARNATAKA | Hassan | H | NS | NS | NS | NS | NS | NS | NS | NS | NS | L | NS | NS |
| KARNATAKA | Dakshina Kannada | NS | NS | NS | NS | NS | NS | H | L | NS | NS | NS | NS | NS |
| KARNATAKA | Kodagu | NS | NS | NS | NS | NS | NS | NS | NS | NS | NS | NS | NS | NS |
| KARNATAKA | Mysore | NS | NS | NS | NS | NS | NS | H | H | H | NS | NS | NS | NS |
| KARNATAKA | Chamarajanagar | NS | NS | NS | NS | NS | NS | NS | NS | NS | NS | NS | NS | NS |
| KARNATAKA | Gulbarga | NS | NS | NS | NS | NS | NS | NS | H | NS | NS | NS | NS | NS |
| KARNATAKA | Yadgir | NS | NS | NS | NS | NS | NS | NS | NS | NS | NS | NS | NS | NS |
| KARNATAKA | Kolar | NS | NS | NS | NS | NS | NS | NS | H | NS | NS | NS | NS | NS |
| KARNATAKA | Chikkaballapura | NS | NS | NS | NS | NS | NS | NS | NS | NS | NS | NS | NS | NS |
| KARNATAKA | Bangalore Rural | NS | NS | NS | NS | NS | NS | NS | NS | NS | NS | NS | NS | NS |
| KARNATAKA | Ramanagara | NS | NS | NS | NS | NS | NS | NS | NS | NS | NS | NS | NS | NS |
| KERALA | Kasaragod | NS | H | NS | NS | NS | NS | NS | NS | NS | NS | NS | NS | NS |
| KERALA | Kannur | NS | NS | NS | NS | NS | NS | NS | L | NS | NS | NS | H | NS |
| KERALA | Wayanad | NS | NS | NS | NS | NS | NS | NS | NS | NS | NS | NS | NS | NS |
| KERALA | Kozhikode | NS | H | NS | H | NS | NS | NS | H | NS | NS | NS | NS | NS |
| KERALA | Malappuram | H | H | NS | H | NS | NS | NS | NS | NS | NS | NS | NS | NS |
| KERALA | Palakkad | NS | H | NS | NS | NS | H | H | H | NS | NS | H | NS | NS |
| KERALA | Thrissur | H | NS | NS | NS | NS | NS | NS | NS | NS | NS | H | NS | NS |
| KERALA | Ernakulam | NS | NS | NS | NS | NS | NS | H | H | NS | NS | NS | H | NS |
| KERALA | Idukki | NS | NS | NS | NS | NS | NS | NS | NS | NS | NS | NS | NS | NS |
| KERALA | Kottayam | H | NS | NS | NS | NS | NS | NS | NS | NS | NS | NS | NS | NS |
| KERALA | Alappuzha | NS | H | NS | NS | NS | NS | NS | NS | NS | NS | NS | NS | NS |
| KERALA | Pathanamthitta | NS | NS | NS | NS | NS | NS | NS | NS | NS | NS | NS | NS | NS |
| KERALA | Kollam | H | H | NS | NS | H | H | NS | NS | NS | H | NS | NS | NS |
| KERALA | Thiruvananthapuram | NS | NS | NS | NS | NS | H | H | NS | NS | NS | NS | NS | NS |
| MADHYA PRADESH | Sheopur | NS | NS | NS | NS | NS | NS | H | NS | NS | NS | NS | NS | NS |
| MADHYA PRADESH | Morena | NS | NS | NS | NS | NS | NS | H | NS | NS | NS | NS | NS | NS |
| MADHYA PRADESH | Bhind | NS | NS | NS | NS | NS | NS | H | NS | NS | NS | NS | NS | NS |
| MADHYA PRADESH | Gwalior | NS | H | NS | NS | NS | H | NS | NS | NS | NS | NS | NS | NS |
| MADHYA PRADESH | Datia | NS | NS | NS | NS | NS | NS | NS | NS | NS | NS | NS | NS | NS |
| MADHYA PRADESH | Shivpuri | NS | NS | NS | NS | NS | H | H | NS | NS | NS | NS | NS | NS |
| MADHYA PRADESH | Tikamgarh | NS | NS | NS | NS | NS | NS | H | NS | NS | NS | NS | NS | NS |
| MADHYA PRADESH | Chhatarpur | NS | H | NS | NS | NS | NS | NS | NS | NS | NS | NS | NS | NS |
| MADHYA PRADESH | Panna | NS | NS | NS | NS | NS | NS | NS | NS | NS | NS | NS | NS | NS |
| MADHYA PRADESH | Sagar | NS | H | NS | H | NS | NS | NS | NS | NS | NS | NS | NS | NS |
| MADHYA PRADESH | Damoh | NS | NS | NS | NS | NS | NS | H | H | NS | NS | NS | NS | NS |
| MADHYA PRADESH | Satna | NS | NS | NS | NS | NS | NS | H | NS | NS | NS | NS | NS | NS |
| MADHYA PRADESH | Rewa | H | NS | NS | NS | NS | NS | H | NS | NS | NS | NS | NS | NS |
| MADHYA PRADESH | Umaria | NS | NS | NS | NS | NS | NS | NS | NS | NS | NS | NS | NS | NS |
| MADHYA PRADESH | Neemuch | NS | NS | NS | NS | NS | NS | NS | NS | NS | NS | NS | NS | NS |
| MADHYA PRADESH | Mandsaur | NS | H | NS | NS | NS | NS | NS | NS | NS | NS | NS | NS | NS |
| MADHYA PRADESH | Ratlam | NS | NS | NS | NS | NS | NS | NS | NS | NS | NS | NS | NS | NS |
| MADHYA PRADESH | Ujjain | NS | NS | NS | NS | NS | NS | NS | NS | NS | NS | NS | NS | NS |
| MADHYA PRADESH | Dewas | NS | H | NS | NS | NS | NS | NS | NS | NS | NS | NS | NS | NS |
| MADHYA PRADESH | Dhar | NS | NS | NS | NS | NS | NS | NS | NS | NS | NS | NS | NS | NS |
| MADHYA PRADESH | Indore | H | H | H | NS | NS | NS | NS | NS | NS | NS | NS | NS | NS |
| MADHYA PRADESH | Khargone (West Nimar) | NS | NS | NS | NS | NS | NS | NS | NS | NS | NS | NS | NS | NS |
| MADHYA PRADESH | Barwani | NS | H | NS | NS | NS | NS | NS | NS | NS | NS | NS | NS | NS |
| MADHYA PRADESH | Rajgarh | NS | NS | NS | NS | NS | NS | NS | NS | NS | NS | NS | NS | NS |
| MADHYA PRADESH | Vidisha | NS | NS | NS | NS | NS | NS | NS | NS | NS | NS | NS | NS | NS |
| MADHYA PRADESH | Bhopal | NS | H | NS | NS | NS | NS | H | NS | NS | NS | NS | NS | NS |
| MADHYA PRADESH | Sehore | NS | NS | NS | NS | NS | NS | NS | NS | NS | NS | NS | NS | NS |
| MADHYA PRADESH | Raisen | NS | NS | NS | NS | NS | NS | NS | NS | NS | NS | NS | NS | NS |
| MADHYA PRADESH | Betul | NS | NS | NS | NS | NS | NS | H | NS | NS | NS | NS | NS | NS |
| MADHYA PRADESH | Harda | NS | NS | NS | NS | NS | NS | NS | NS | NS | NS | NS | NS | NS |
| MADHYA PRADESH | Hoshangabad | NS | NS | NS | NS | H | NS | NS | NS | NS | NS | NS | NS | NS |
| MADHYA PRADESH | Katni | NS | NS | NS | NS | NS | NS | NS | NS | NS | NS | NS | NS | NS |
| MADHYA PRADESH | Jabalpur | NS | H | NS | NS | NS | NS | H | NS | NS | NS | NS | NS | NS |
| MADHYA PRADESH | Narsimhapur | NS | NS | NS | NS | NS | NS | H | NS | NS | NS | NS | NS | NS |
| MADHYA PRADESH | Dindori | NS | H | NS | NS | NS | NS | NS | NS | NS | NS | NS | NS | NS |
| MADHYA PRADESH | Mandla | NS | NS | NS | NS | NS | NS | NS | NS | NS | NS | NS | NS | NS |
| MADHYA PRADESH | Chhindwara | NS | NS | NS | NS | NS | NS | H | NS | NS | NS | NS | NS | NS |
| MADHYA PRADESH | Seoni | NS | NS | NS | NS | NS | NS | H | NS | NS | NS | NS | NS | NS |
| MADHYA PRADESH | Balaghat | NS | NS | NS | NS | NS | NS | NS | H | NS | NS | NS | NS | NS |
| MADHYA PRADESH | Guna | NS | NS | NS | NS | NS | NS | H | NS | NS | NS | NS | NS | NS |
| MADHYA PRADESH | Ashoknagar | NS | NS | NS | NS | NS | NS | NS | NS | NS | NS | NS | NS | NS |
| MADHYA PRADESH | Shahdol | NS | NS | NS | NS | NS | NS | NS | NS | NS | NS | NS | NS | NS |
| MADHYA PRADESH | Anuppur | NS | NS | NS | NS | NS | NS | NS | NS | NS | NS | NS | NS | NS |
| MADHYA PRADESH | Sidhi | NS | NS | NS | NS | NS | NS | H | NS | NS | NS | NS | NS | NS |
| MADHYA PRADESH | Singrauli | NS | NS | NS | NS | NS | NS | NS | NS | NS | NS | NS | NS | NS |
| MADHYA PRADESH | Jhabua | NS | NS | NS | NS | NS | NS | NS | NS | NS | NS | NS | NS | NS |
| MADHYA PRADESH | Alirajpur | NS | NS | NS | NS | NS | NS | NS | NS | NS | NS | NS | NS | NS |
| MADHYA PRADESH | Khandwa (East Nimar) | NA | NA | NA | NA | NA | NA | NA | NA | NA | NA | NA | NA | NA |
| MADHYA PRADESH | Burhanpur | NS | NS | NS | NS | NS | NS | NS | NS | NS | NS | NS | NS | NS |
| MADHYA PRADESH | Agar Malwa | NS | NS | NS | NS | NS | NS | NS | NS | NS | NS | NS | NS | NS |
| MADHYA PRADESH | Shajapur | NS | NS | NS | NS | NS | NS | NS | NS | NS | NS | NS | NS | NS |
| MAHARASHTRA | Nandurbar | NS | NS | NS | NS | NS | NS | NS | H | NS | NS | NS | NS | NS |
| MAHARASHTRA | Dhule | NS | NS | NS | NS | H | NS | NS | NS | NS | NS | NS | NS | NS |
| MAHARASHTRA | Jalgaon | H | NS | H | NS | H | NS | NS | NS | NS | NS | NS | NS | NS |
| MAHARASHTRA | Buldana | NS | NS | NS | NS | NS | NS | NS | NS | NS | NS | H | L | NS |
| MAHARASHTRA | Akola | NS | NS | NS | NS | NS | L | H | NS | NS | NS | NS | NS | NS |
| MAHARASHTRA | Washim | NS | NS | NS | NS | NS | NS | H | NS | NS | NS | NS | NS | NS |
| MAHARASHTRA | Amravati | NS | NS | NS | NS | NS | NS | H | L | NS | NS | NS | NS | NS |
| MAHARASHTRA | Wardha | NS | NS | NS | NS | NS | NS | NS | H | NS | L | NS | H | NS |
| MAHARASHTRA | Nagpur | NS | H | NS | NS | NS | NS | NS | NS | NS | NS | NS | NS | NS |
| MAHARASHTRA | Bhandara | NS | H | H | NS | NS | NS | NS | NS | NS | NS | NS | NS | NS |
| MAHARASHTRA | Gondiya | NS | H | NS | NS | NS | NS | NS | H | NS | NS | NS | NS | NS |
| MAHARASHTRA | Gadchiroli | NS | NS | NS | NS | NS | NS | H | NS | NS | NS | NS | NS | NS |
| MAHARASHTRA | Chandrapur | NS | NS | NS | NS | H | NS | H | H | NS | NS | H | NS | NS |
| MAHARASHTRA | Yavatmal | NS | NS | NS | NS | NS | NS | H | NS | NS | NS | NS | H | NS |
| MAHARASHTRA | Nanded | NS | H | NS | NS | NS | NS | H | NS | NS | NS | NS | H | NS |
| MAHARASHTRA | Hingoli | NS | H | NS | NS | NS | NS | NS | L | NS | NS | NS | NS | NS |
| MAHARASHTRA | Parbhani | NS | H | NS | NS | NS | NS | H | NS | NS | NS | NS | H | NS |
| MAHARASHTRA | Jalna | NS | H | NS | NS | NS | NS | NS | NS | NS | NS | NS | NS | NS |
| MAHARASHTRA | Aurangabad | H | NS | NS | NS | NS | H | NS | L | NS | NS | L | H | NS |
| MAHARASHTRA | Nashik | NS | NS | H | NS | NS | NS | H | NS | NS | NS | NS | H | NS |
| MAHARASHTRA | Mumbai Suburban | H | NS | NS | NS | H | NS | H | NS | NS | NS | H | H | H |
| MAHARASHTRA | Mumbai | NS | H | NS | NS | NS | H | H | NS | H | NS | H | NS | NS |
| MAHARASHTRA | Raigarh | NS | NS | NS | NS | NS | NS | NS | NS | NS | NS | H | H | NS |
| MAHARASHTRA | Pune | H | H | H | H | NS | NS | H | H | L | H | L | H | NS |
| MAHARASHTRA | Ahmadnagar | NS | H | NS | NS | NS | NS | NS | NS | NS | H | NS | NS | NS |
| MAHARASHTRA | Bid | H | NS | NS | NS | NS | NS | NS | NS | NS | NS | NS | NS | NS |
| MAHARASHTRA | Latur | NS | H | NS | NS | NS | NS | NS | NS | NS | NS | NS | NS | NS |
| MAHARASHTRA | Osmanabad | NS | H | NS | NS | H | NS | NS | NS | NS | NS | NS | NS | NS |
| MAHARASHTRA | Solapur | NS | H | NS | NS | NS | NS | H | NS | L | NS | H | L | NS |
| MAHARASHTRA | Satara | NS | H | NS | NS | NS | NS | H | NS | L | NS | NS | NS | NS |
| MAHARASHTRA | Ratnagiri | NS | NS | NS | NS | NS | NS | NS | NS | NS | NS | NS | NS | NS |
| MAHARASHTRA | Sindhudurg | NS | NS | NS | NS | NS | NS | NS | NS | NS | NS | NS | NS | NS |
| MAHARASHTRA | Kolhapur | NS | NS | NS | NS | H | H | NS | H | NS | NS | NS | H | NS |
| MAHARASHTRA | Sangli | NS | NS | NS | NS | NS | NS | H | NS | H | NS | NS | NS | NS |
| MAHARASHTRA | Palghar | H | H | NS | NS | NS | H | NS | NS | NS | NS | NS | NS | NS |
| MAHARASHTRA | Thane | NS | NS | NS | NS | H | H | H | H | NS | H | H | H | NS |
| MANIPUR | Senapati | NS | NS | NS | NS | NS | NS | NS | NS | NS | NS | NS | NS | NS |
| MANIPUR | Tamenglong | NS | NS | NS | NS | NS | NS | NS | NS | NS | NS | NS | NS | NS |
| MANIPUR | Churachandpur | NS | NS | NS | NS | NS | NS | NS | NS | NS | NS | NS | NS | NS |
| MANIPUR | Bishnupur | NS | NS | NS | NS | NS | NS | NS | NS | NS | NS | NS | NS | NS |
| MANIPUR | Thoubal | NS | NS | NS | NS | NS | NS | NS | NS | NS | NS | NS | NS | NS |
| MANIPUR | Imphal West | NS | NS | NS | NS | NS | NS | NS | NS | NS | NS | NS | NS | NS |
| MANIPUR | Imphal East | NS | NS | NS | NS | NS | NS | NS | NS | NS | NS | NS | NS | NS |
| MANIPUR | Ukhrul | NS | NS | NS | NS | NS | NS | NS | NS | NS | NS | NS | NS | NS |
| MANIPUR | Chandel | NS | NS | NS | NS | NS | NS | NS | NS | NS | NS | NS | NS | NS |
| NCT OF DELHI | Central | NS | NS | NS | NS | NS | NS | H | NS | NS | NS | NS | NS | NS |
| NCT OF DELHI | East | NS | NS | NS | NS | NS | NS | H | NS | NS | NS | H | NS | NS |
| NCT OF DELHI | New Delhi | NS | NS | NS | NS | NS | NS | H | NS | NS | NS | NS | NS | NS |
| NCT OF DELHI | North | NS | NS | NS | NS | NS | NS | H | NS | NS | NS | NS | NS | NS |
| NCT OF DELHI | North East | NS | NS | NS | NS | NS | NS | H | NS | NS | NS | NS | NS | NS |
| NCT OF DELHI | North West | NS | NS | NS | NS | NS | NS | H | NS | NS | NS | NS | NS | NS |
| NCT OF DELHI | Shahdara | NS | NS | NS | NS | NS | NS | H | NS | NS | NS | NS | NS | NS |
| NCT OF DELHI | South | NS | NS | NS | NS | NS | NS | H | NS | NS | NS | NS | NS | NS |
| NCT OF DELHI | South East | NS | NS | NS | NS | NS | NS | H | NS | NS | NS | NS | NS | NS |
| NCT OF DELHI | South West | NS | NS | NS | NS | NS | NS | H | NS | NS | NS | NS | NS | NS |
| NCT OF DELHI | West | NS | H | NS | H | NS | NS | H | NS | NS | NS | H | NS | NS |
| ODISHA | Bargarh | NS | NS | NS | NS | NS | NS | H | NS | NS | NS | NS | NS | NS |
| ODISHA | Jharsuguda | NS | NS | NS | NS | NS | NS | NS | NS | NS | NS | NS | NS | NS |
| ODISHA | Sambalpur | NS | NS | NS | NS | NS | NS | NS | NS | NS | NS | NS | NS | NS |
| ODISHA | Debagarh | NS | NS | NS | NS | NS | NS | NS | NS | NS | NS | NS | NS | NS |
| ODISHA | Sundargarh | NS | NS | NS | NS | NS | NS | H | NS | NS | NS | H | NS | NS |
| ODISHA | Kendujhar | NS | NS | NS | NS | NS | NS | NS | NS | NS | NS | NS | NS | NS |
| ODISHA | Mayurbhanj | NS | NS | NS | NS | NS | NS | NS | NS | NS | NS | NS | NS | NS |
| ODISHA | Baleshwar | NS | NS | NS | NS | NS | NS | NS | NS | NS | NS | NS | NS | NS |
| ODISHA | Bhadrak | NS | NS | NS | NS | NS | NS | NS | NS | NS | NS | NS | NS | NS |
| ODISHA | Kendrapara | NS | NS | NS | NS | NS | NS | NS | NS | NS | NS | NS | NS | NS |
| ODISHA | Jagatsinghapur | NS | NS | NS | NS | NS | NS | NS | NS | NS | NS | NS | NS | NS |
| ODISHA | Cuttack | NS | NS | NS | NS | NS | NS | H | NS | NS | NS | NS | NS | NS |
| ODISHA | Jajapur | NS | NS | NS | NS | NS | NS | NS | NS | NS | NS | NS | NS | NS |
| ODISHA | Dhenkanal | NS | NS | NS | NS | NS | NS | H | NS | NS | NS | NS | NS | NS |
| ODISHA | Anugul | NS | NS | NS | NS | NS | NS | NS | NS | NS | NS | NS | NS | NS |
| ODISHA | Nayagarh | NS | NS | NS | NS | NS | NS | NS | NS | NS | NS | NS | NS | NS |
| ODISHA | Khordha | NS | NS | NS | NS | NS | H | H | NS | NS | NS | NS | NS | NS |
| ODISHA | Puri | NS | NS | NS | NS | NS | NS | H | NS | NS | NS | NS | NS | NS |
| ODISHA | Ganjam | NS | NS | NS | NS | NS | NS | H | NS | NS | NS | NS | NS | NS |
| ODISHA | Gajapati | NS | NS | NS | NS | NS | NS | NS | NS | NS | NS | NS | NS | NS |
| ODISHA | Kandhamal | NS | NS | NS | NS | NS | NS | H | NS | NS | NS | NS | NS | NS |
| ODISHA | Baudh | NS | NS | NS | NS | NS | NS | NS | NS | NS | NS | NS | NS | NS |
| ODISHA | Subarnapur | NS | NS | NS | NS | NS | NS | NS | NS | NS | NS | NS | NS | NS |
| ODISHA | Balangir | NS | NS | NS | NS | NS | NS | H | NS | NS | NS | NS | NS | NS |
| ODISHA | Nuapada | NS | NS | NS | NS | NS | NS | NS | NS | NS | NS | NS | NS | NS |
| ODISHA | Kalahandi | NS | NS | NS | NS | NS | NS | H | NS | NS | NS | NS | NS | NS |
| ODISHA | Rayagada | NS | NS | NS | NS | NS | NS | NS | NS | NS | NS | NS | NS | NS |
| ODISHA | Nabarangapur | NS | NS | NS | NS | NS | NS | H | NS | NS | NS | NS | NS | NS |
| ODISHA | Koraput | NS | H | NS | NS | NS | NS | H | NS | NS | NS | NS | NS | NS |
| ODISHA | Malkangiri | NS | NS | NS | NS | NS | NS | H | NS | NS | NS | NS | NS | NS |
| PUNJAB | Kapurthala | NS | NS | NS | NS | NS | NS | NS | NS | NS | NS | NS | NS | NS |
| PUNJAB | Jalandhar | NS | NS | NS | NS | NS | NS | NS | NS | NS | NS | NS | NS | NS |
| PUNJAB | Hoshiarpur | NS | NS | NS | NS | NS | NS | NS | NS | NS | NS | NS | NS | NS |
| PUNJAB | Shahid Bhagat Singh Nagar | NS | NS | NS | NS | NS | NS | NS | NS | NS | NS | NS | NS | NS |
| PUNJAB | Fatehgarh Sahib | NS | NS | NS | NS | NS | NS | NS | NS | NS | NS | NS | NS | NS |
| PUNJAB | Ludhiana | NS | NS | NS | NS | NS | NS | NS | NS | NS | NS | NS | NS | NS |
| PUNJAB | Moga | NS | NS | NS | NS | NS | NS | NS | NS | NS | NS | NS | NS | NS |
| PUNJAB | Muktsar | NS | NS | NS | NS | NS | NS | NS | NS | NS | NS | NS | NS | NS |
| PUNJAB | Faridkot | NS | NS | NS | NS | NS | NS | NS | NS | NS | NS | NS | NS | NS |
| PUNJAB | Bathinda | NS | NS | NS | NS | NS | NS | NS | NS | NS | NS | NS | NS | NS |
| PUNJAB | Mansa | NS | NS | NS | NS | NS | NS | NS | NS | NS | NS | NS | NS | NS |
| PUNJAB | Patiala | NS | NS | NS | NS | NS | NS | NS | NS | NS | NS | NS | NS | NS |
| PUNJAB | Amritsar | NS | NS | NS | NS | NS | NS | NS | NS | NS | NS | NS | NS | NS |
| PUNJAB | Tarn Taran | NS | NS | NS | NS | NS | NS | NS | NS | NS | NS | NS | NS | NS |
| PUNJAB | Rupnagar | NS | NS | NS | NS | NS | NS | NS | NS | NS | NS | NS | NS | NS |
| PUNJAB | Sahibzada Ajit Singh Nagar | NS | NS | NS | NS | NS | NS | NS | NS | NS | NS | NS | NS | NS |
| PUNJAB | Sangrur | NS | NS | NS | NS | NS | NS | NS | NS | NS | NS | NS | NS | NS |
| PUNJAB | Barnala | NS | NS | NS | NS | NS | NS | NS | NS | NS | NS | NS | NS | NS |
| PUNJAB | Fazilka | NS | NS | NS | NS | NS | NS | NS | NS | NS | NS | NS | NS | NS |
| PUNJAB | Firozpur | NS | NS | NS | NS | NS | NS | NS | NS | NS | NS | NS | NS | NS |
| PUNJAB | Gurdaspur | NS | NS | NS | NS | NS | NS | NS | NS | NS | NS | NS | NS | NS |
| PUNJAB | Pathankot | NS | NS | NS | NS | NS | NS | NS | NS | NS | NS | NS | NS | NS |
| RAJASTHAN | Ganganagar | NS | NS | NS | NS | NS | NS | NS | NS | NS | NS | NS | NS | NS |
| RAJASTHAN | Hanumangarh | NS | NS | NS | NS | NS | NS | H | NS | NS | NS | NS | NS | NS |
| RAJASTHAN | Bikaner | NS | NS | NS | NS | NS | NS | H | H | NS | NS | H | NS | NS |
| RAJASTHAN | Churu | NS | NS | NS | NS | NS | NS | NS | NS | NS | NS | NS | NS | NS |
| RAJASTHAN | Jhunjhunun | NS | NS | NS | NS | NS | NS | H | NS | NS | NS | NS | NS | NS |
| RAJASTHAN | Alwar | NS | H | NS | NS | H | H | NS | NS | NS | NS | NS | NS | NS |
| RAJASTHAN | Bharatpur | NS | NS | NS | NS | NS | NS | H | NS | NS | NS | NS | NS | NS |
| RAJASTHAN | Dhaulpur | NS | NS | NS | NS | NS | NS | H | NS | NS | NS | NS | NS | NS |
| RAJASTHAN | Karauli | NS | NS | NS | NS | NS | NS | NS | NS | NS | NS | NS | NS | NS |
| RAJASTHAN | Sawai Madhopur | NS | NS | NS | NS | NS | NS | NS | NS | NS | NS | NS | NS | NS |
| RAJASTHAN | Dausa | NS | NS | NS | NS | NS | NS | NS | NS | NS | NS | NS | NS | NS |
| RAJASTHAN | Jaipur | NS | H | NS | NS | NS | NS | NS | NS | NS | NS | NS | NS | NS |
| RAJASTHAN | Sikar | NS | NS | NS | NS | NS | NS | NS | NS | NS | NS | NS | NS | NS |
| RAJASTHAN | Nagaur | NS | NS | NS | NS | NS | NS | H | NS | NS | NS | NS | NS | NS |
| RAJASTHAN | Jodhpur | NS | NS | NS | NS | H | NS | H | NS | NS | NS | NS | NS | NS |
| RAJASTHAN | Jaisalmer | NS | NS | NS | NS | NS | NS | NS | NS | NS | NS | NS | NS | NS |
| RAJASTHAN | Barmer | NS | NS | NS | NS | NS | NS | NS | NS | NS | NS | NS | NS | NS |
| RAJASTHAN | Jalor | H | NS | NS | NS | NS | NS | NS | NS | NS | NS | H | NS | NS |
| RAJASTHAN | Sirohi | NS | NS | NS | NS | NS | NS | NS | NS | NS | NS | NS | NS | NS |
| RAJASTHAN | Pali | NS | NS | NS | NS | NS | NS | H | NS | NS | NS | NS | NS | NS |
| RAJASTHAN | Ajmer | NS | NS | NS | NS | NS | NS | H | H | NS | NS | NS | NS | NS |
| RAJASTHAN | Tonk | NS | NS | NS | NS | NS | NS | NS | NS | NS | NS | NS | NS | NS |
| RAJASTHAN | Bundi | NS | NS | NS | NS | NS | NS | NS | NS | NS | NS | NS | NS | NS |
| RAJASTHAN | Bhilwara | NS | H | NS | NS | NS | NS | H | NS | NS | NS | NS | NS | NS |
| RAJASTHAN | Rajsamand | NS | NS | NS | NS | NS | NS | NS | NS | NS | NS | NS | NS | NS |
| RAJASTHAN | Dungarpur | H | NS | NS | H | NS | NS | NS | NS | NS | NS | NS | NS | NS |
| RAJASTHAN | Banswara | NS | NS | NS | H | NS | NS | H | NS | NS | NS | NS | NS | NS |
| RAJASTHAN | Chittaurgarh | NS | NS | NS | NS | NS | NS | NS | NS | NS | NS | NS | NS | NS |
| RAJASTHAN | Kota | NS | NS | NS | NS | NS | NS | NS | NS | NS | NS | NS | NS | NS |
| RAJASTHAN | Baran | NS | NS | NS | NS | NS | NS | H | NS | NS | NS | NS | NS | NS |
| RAJASTHAN | Jhalawar | NS | NS | NS | NS | NS | NS | NS | H | NS | NS | NS | NS | NS |
| RAJASTHAN | Udaipur | H | NS | NS | H | NS | NS | NS | NS | NS | NS | NS | NS | NS |
| RAJASTHAN | Pratapgarh | NS | NS | NS | NS | NS | NS | H | NS | NS | NS | NS | NS | NS |
| TAMIL NADU | Thiruvallur | NS | NS | NS | L | NS | H | H | H | NS | NS | NS | NS | NS |
| TAMIL NADU | Chennai | NS | NS | NS | NS | NS | NS | H | NS | NS | NS | NS | NS | NS |
| TAMIL NADU | Kancheepuram | NS | H | NS | NS | NS | NS | H | NS | NS | NS | NS | NS | NS |
| TAMIL NADU | Vellore | H | NS | NS | NS | NS | NS | NS | NS | NS | NS | NS | NS | NS |
| TAMIL NADU | Tiruvannamalai | NS | NS | NS | NS | NS | NS | H | NS | NS | NS | NS | NS | NS |
| TAMIL NADU | Viluppuram | NS | NS | H | NS | NS | NS | H | NS | NS | NS | NS | NS | NS |
| TAMIL NADU | Salem | NS | NS | NS | NS | NS | NS | NS | NS | NS | NS | NS | NS | NS |
| TAMIL NADU | Namakkal | NS | NS | NS | NS | NS | NS | H | NS | NS | NS | NS | NS | NS |
| TAMIL NADU | Erode | NS | NS | NS | NS | NS | L | H | L | NS | NS | NS | NS | NS |
| TAMIL NADU | The Nilgiris | NS | NS | NS | NS | NS | NS | NS | NS | NS | NS | NS | NS | NS |
| TAMIL NADU | Dindigul | NS | NS | NS | NS | NS | NS | NS | NS | NS | NS | NS | NS | NS |
| TAMIL NADU | Karur | NS | NS | NS | NS | NS | NS | NS | NS | NS | NS | NS | NS | NS |
| TAMIL NADU | Tiruchirappalli | NS | NS | NS | NS | NS | NS | NS | NS | NS | NS | NS | NS | NS |
| TAMIL NADU | Perambalur | NS | NS | NS | NS | NS | NS | NS | NS | NS | NS | NS | NS | NS |
| TAMIL NADU | Ariyalur | NS | NS | NS | NS | NS | NS | NS | NS | NS | NS | NS | NS | NS |
| TAMIL NADU | Cuddalore | NS | NS | NS | NS | NS | NS | NS | NS | NS | NS | NS | NS | NS |
| TAMIL NADU | Nagapattinam | NS | NS | NS | NS | NS | NS | H | NS | NS | NS | NS | NS | NS |
| TAMIL NADU | Thiruvarur | NS | NS | NS | NS | NS | NS | NS | NS | NS | NS | NS | NS | NS |
| TAMIL NADU | Thanjavur | NS | NS | NS | NS | NS | NS | NS | NS | NS | NS | NS | NS | NS |
| TAMIL NADU | Pudukkottai | NS | NS | NS | NS | NS | NS | H | NS | NS | NS | NS | NS | NS |
| TAMIL NADU | Sivaganga | NS | NS | NS | NS | NS | NS | H | NS | NS | NS | NS | NS | NS |
| TAMIL NADU | Madurai | NS | NS | NS | NS | H | H | H | NS | NS | NS | NS | NS | NS |
| TAMIL NADU | Theni | NS | NS | NS | NS | NS | NS | NS | NS | NS | NS | NS | NS | NS |
| TAMIL NADU | Virudhunagar | NS | NS | NS | NS | NS | NS | H | NS | NS | NS | NS | NS | NS |
| TAMIL NADU | Ramanathapuram | NS | NS | NS | NS | NS | NS | NS | NS | NS | NS | NS | NS | NS |
| TAMIL NADU | Thoothukkudi | NS | NS | NS | NS | H | NS | NS | NS | NS | NS | NS | NS | NS |
| TAMIL NADU | Tirunelveli | NS | NS | NS | NS | NS | NS | NS | NS | NS | NS | NS | NS | NS |
| TAMIL NADU | Kanniyakumari | NS | NS | NS | NS | NS | NS | H | L | NS | NS | NS | NS | NS |
| TAMIL NADU | Dharmapuri | NS | NS | NS | NS | NS | NS | H | L | NS | NS | NS | NS | NS |
| TAMIL NADU | Krishnagiri | NS | NS | NS | H | H | H | NS | NS | NS | NS | NS | NS | NS |
| TAMIL NADU | Coimbatore | NS | NS | NS | NS | NS | NS | H | NS | NS | NS | H | NS | NS |
| TAMIL NADU | Tiruppur | NS | H | NS | NS | NS | NS | NS | NS | NS | NS | NS | NS | NS |
| TELANGANA | Adilabad | NS | NS | NS | NS | NS | NS | NS | NS | NS | NS | NS | NS | NS |
| TELANGANA | Bhadradri Kothagudem | NS | NS | NS | NS | NS | NS | NS | NS | NS | NS | NS | NS | NS |
| TELANGANA | Hyderabad | NS | NS | H | NS | NS | NS | NS | NS | NS | NS | NS | NS | NS |
| TELANGANA | Jagitial | NS | NS | NS | NS | NS | NS | NS | NS | NS | NS | NS | H | NS |
| TELANGANA | Jangoan | NS | NS | NS | NS | NS | NS | NS | NS | NS | NS | NS | NS | NS |
| TELANGANA | Jayashankar Bhupalapally | NS | NS | NS | NS | NS | NS | NS | NS | NS | NS | NS | NS | NS |
| TELANGANA | Jogulamba Gadwal | NS | NS | NS | NS | NS | NS | NS | NS | NS | NS | NS | NS | NS |
| TELANGANA | Kamareddy | NS | NS | NS | NS | NS | NS | NS | NS | NS | NS | NS | NS | NS |
| TELANGANA | Karimnagar | NS | NS | NS | NS | H | H | NS | NS | NS | NS | NS | NS | NS |
| TELANGANA | Khammam | NS | H | NS | NS | NS | NS | H | NS | NS | NS | NS | NS | NS |
| TELANGANA | Komaram Bheem Asifabad | NS | NS | NS | NS | NS | NS | NS | NS | NS | NS | NS | NS | NS |
| TELANGANA | Mahabubabad | NS | NS | NS | NS | NS | NS | NS | NS | NS | NS | NS | NS | NS |
| TELANGANA | Mahabubnagar | NS | NS | NS | NS | NS | NS | NS | L | NS | NS | NS | NS | NS |
| TELANGANA | Mancherial | NS | NS | NS | NS | NS | H | NS | NS | NS | NS | NS | NS | NS |
| TELANGANA | Medak | NS | NS | NS | NS | NS | NS | NS | NS | NS | NS | NS | NS | NS |
| TELANGANA | Medchal-Malkajgiri | NS | NS | NS | NS | NS | NS | NS | NS | NS | NS | NS | NS | NS |
| TELANGANA | Nagarkurnool | NS | NS | NS | NS | NS | NS | NS | L | NS | NS | NS | NS | NS |
| TELANGANA | Nalgonda | NS | NS | NS | NS | NS | NS | NS | L | NS | NS | NS | NS | NS |
| TELANGANA | Nirmal | NS | NS | NS | NS | NS | NS | NS | NS | NS | NS | NS | NS | NS |
| TELANGANA | Nizamabad | NS | NS | NS | NS | NS | NS | NS | NS | NS | NS | NS | NS | NS |
| TELANGANA | Peddapalli | NS | NS | NS | NS | NS | NS | NS | NS | NS | NS | NS | NS | NS |
| TELANGANA | Rajanna Sircilla | NS | NS | NS | NS | NS | NS | NS | NS | NS | NS | NS | NS | NS |
| TELANGANA | Ranga Reddy | NS | NS | NS | NS | NS | NS | NS | NS | NS | NS | NS | NS | NS |
| TELANGANA | Sangareddy | NS | NS | NS | NS | NS | NS | NS | NS | NS | NS | NS | NS | NS |
| TELANGANA | Siddipet | NS | NS | NS | NS | NS | NS | NS | NS | NS | NS | NS | NS | NS |
| TELANGANA | Suryapet | NS | NS | NS | NS | H | NS | NS | NS | NS | NS | NS | NS | NS |
| TELANGANA | Vikarabad | NS | H | NS | NS | NS | NS | NS | NS | NS | NS | NS | NS | NS |
| TELANGANA | Wanaparthy | NS | NS | NS | NS | NS | NS | NS | NS | NS | NS | NS | NS | NS |
| TELANGANA | Warangal Rural | NS | NS | NS | NS | NS | NS | NS | L | NS | NS | NS | NS | NS |
| TELANGANA | Warangal Urban | NS | NS | NS | NS | NS | NS | NS | NS | NS | NS | NS | NS | NS |
| TELANGANA | Yadadri Bhuvanagiri | NS | NS | NS | NS | NS | NS | NS | NS | NS | NS | NS | NS | NS |
| TRIPURA | Dhalai | NS | NS | NS | NS | NS | NS | NS | NS | NS | NS | NS | NS | NS |
| TRIPURA | Gomati | NS | NS | NS | NS | NS | NS | NS | NS | NS | NS | NS | NS | NS |
| TRIPURA | Khowai | NS | NS | NS | NS | NS | NS | NS | NS | NS | NS | NS | NS | NS |
| TRIPURA | North Tripura | NS | NS | NS | NS | NS | NS | NS | NS | NS | NS | NS | NS | NS |
| TRIPURA | Sepahijala | NS | NS | NS | NS | NS | NS | NS | NS | NS | NS | NS | NS | NS |
| TRIPURA | South Tripura | NS | NS | NS | NS | NS | NS | NS | NS | NS | NS | NS | NS | NS |
| TRIPURA | Unakoti | NS | NS | NS | NS | NS | NS | NS | NS | NS | NS | NS | NS | NS |
| TRIPURA | West Tripura | NS | NS | NS | NS | NS | H | NS | NS | NS | NS | NS | NS | NS |
| UTTAR PRADESH | Saharanpur | NS | NS | NS | NS | NS | NS | H | NS | NS | NS | NS | NS | NS |
| UTTAR PRADESH | Bijnor | NS | NS | NS | NS | NS | NS | H | NS | NS | NS | NS | NS | NS |
| UTTAR PRADESH | Rampur | NS | NS | NS | NS | NS | NS | NS | NS | NS | NS | NS | NS | NS |
| UTTAR PRADESH | Jyotiba Phule Nagar | NS | NS | NS | NS | NS | NS | NS | NS | NS | NS | NS | NS | NS |
| UTTAR PRADESH | Meerut | NS | NS | NS | NS | NS | NS | H | NS | NS | NS | NS | NS | NS |
| UTTAR PRADESH | Baghpat | NS | NS | NS | NS | NS | NS | NS | NS | NS | NS | NS | NS | NS |
| UTTAR PRADESH | Gautam Buddha Nagar | NS | NS | NS | NS | NS | NS | NS | NS | NS | NS | NS | NS | NS |
| UTTAR PRADESH | Bulandshahr | NS | NS | NS | NS | NS | NS | H | NS | NS | NS | NS | NS | NS |
| UTTAR PRADESH | Aligarh | NS | NS | NS | NS | NS | NS | NS | H | NS | NS | NS | NS | NS |
| UTTAR PRADESH | Mahamaya Nagar | NS | NS | NS | NS | NS | NS | NS | NS | NS | NS | NS | NS | NS |
| UTTAR PRADESH | Mathura | NS | NS | NS | NS | NS | NS | NS | NS | NS | NS | NS | NS | NS |
| UTTAR PRADESH | Agra | NS | NS | NS | NS | NS | NS | H | H | NS | NS | NS | NS | NS |
| UTTAR PRADESH | Firozabad | NS | NS | NS | NS | NS | NS | H | NS | NS | NS | NS | NS | NS |
| UTTAR PRADESH | Mainpuri | NS | NS | NS | NS | NS | NS | NS | NS | NS | NS | NS | NS | NS |
| UTTAR PRADESH | Bareilly | L | NS | NS | H | H | NS | H | NS | NS | H | NS | NS | NS |
| UTTAR PRADESH | Pilibhit | NS | NS | NS | NS | NS | NS | NS | NS | NS | NS | NS | NS | NS |
| UTTAR PRADESH | Shahjahanpur | NS | NS | NS | NS | NS | NS | H | L | NS | NS | NS | NS | NS |
| UTTAR PRADESH | Kheri | NS | H | NS | NS | NS | NS | NS | NS | NS | NS | NS | NS | NS |
| UTTAR PRADESH | Sitapur | NS | NS | NS | NS | NS | NS | NS | NS | NS | NS | NS | H | NS |
| UTTAR PRADESH | Hardoi | NS | NS | NS | NS | NS | NS | H | NS | NS | NS | NS | NS | NS |
| UTTAR PRADESH | Unnao | NS | NS | NS | NS | NS | NS | NS | NS | NS | NS |  | H | L |
| UTTAR PRADESH | Lucknow | NS | NS | NS | NS | NS | NS | NS | L | L | NS | NS | NS | NS |
| UTTAR PRADESH | Farrukhabad | NS | NS | NS | NS | NS | NS | H | NS | NS | NS | NS | NS | NS |
| UTTAR PRADESH | Kannauj | NS | NS | NS | NS | NS | NS | NS | NS | NS | NS | NS | NS | NS |
| UTTAR PRADESH | Etawah | NS | NS | NS | NS | NS | NS | NS | NS | NS | NS | NS | NS | NS |
| UTTAR PRADESH | Auraiya | NS | NS | NS | NS | NS | NS | NS | NS | NS | NS | NS | NS | NS |
| UTTAR PRADESH | Kanpur Dehat | NS | NS | NS | NS | NS | NS | NS | NS | NS | NS | NS | NS | NS |
| UTTAR PRADESH | Kanpur Nagar | NS | NS | NS | NS | NS | NS | H | NS | NS | NS | NS | NS | NS |
| UTTAR PRADESH | Jalaun | NS | NS | NS | NS | NS | NS | NS | NS | NS | NS | NS | NS | NS |
| UTTAR PRADESH | Jhansi | NS | NS | L | NS | NS | NS | NS | NS | NS | NS | NS | NS | NS |
| UTTAR PRADESH | Lalitpur | NS | NS | NS | NS | NS | NS | H | NS | NS | NS | NS | NS | NS |
| UTTAR PRADESH | Hamirpur | NS | NS | NS | NS | NS | NS | NS | NS | NS | NS | NS | NS | NS |
| UTTAR PRADESH | Mahoba | NS | NS | NS | NS | NS | NS | NS | NS | NS | NS | NS | NS | NS |
| UTTAR PRADESH | Banda | NS | NS | NS | NS | NS | NS | NS | NS | NS | NS | NS | NS | NS |
| UTTAR PRADESH | Chitrakoot | NS | NS | NS | NS | NS | NS | NS | NS | NS | NS | NS | NS | NS |
| UTTAR PRADESH | Fatehpur | NS | NS | NS | NS | NS | NS | H | NS | NS | NS | NS | NS | NS |
| UTTAR PRADESH | Pratapgarh | NS | NS | NS | NS | NS | NS | H | NS | NS | NS | NS | NS | NS |
| UTTAR PRADESH | Kaushambi | NS | NS | NS | NS | NS | NS | H | NS | NS | NS | NS | NS | NS |
| UTTAR PRADESH | Allahabad | NS | H | NS | NS | NS | NS | H | NS | NS | NS | NS | NS | NS |
| UTTAR PRADESH | Bara Banki | NS | NS | NS | NS | NS | NS | H | NS | NS | NS | NS | NS | NS |
| UTTAR PRADESH | Faizabad | NS | NS | NS | NS | NS | NS | H | NS | NS | NS | NS | NS | NS |
| UTTAR PRADESH | Ambedkar Nagar | NS | NS | NS | NS | NS | NS | NS | NS | NS | NS | NS | NS | NS |
| UTTAR PRADESH | Bahraich | NS | NS | NS | NS | NS | NS | NS | NS | NS | NS | NS | NS | NS |
| UTTAR PRADESH | Shrawasti | NS | NS | NS | NS | NS | NS | NS | NS | NS | NS | NS | NS | NS |
| UTTAR PRADESH | Balrampur | NS | NS | NS | NS | NS | NS | NS | NS | NS | NS | NS | NS | NS |
| UTTAR PRADESH | Gonda | H | NS | H | NS | NS | NS | NS | NS | NS | NS | NS | NS | NS |
| UTTAR PRADESH | Siddharthnagar | NS | NS | NS | NS | NS | NS | NS | NS | NS | NS | NS | NS | NS |
| UTTAR PRADESH | Basti | NS | NS | NS | NS | NS | NS | NS | NS | NS | NS | NS | NS | NS |
| UTTAR PRADESH | Sant Kabir Nagar | NS | NS | NS | NS | NS | NS | NS | H | NS | NS | NS | NS | NS |
| UTTAR PRADESH | Mahrajganj | NS | NS | NS | NS | NS | NS | H | NS | NS | NS | NS | NS | NS |
| UTTAR PRADESH | Gorakhpur | NS | NS | NS | NS | NS | NS | H | NS | NS | NS | NS | NS | NS |
| UTTAR PRADESH | Kushinagar | NS | NS | NS | H | NS | NS | H | NS | NS | NS | NS | NS | NS |
| UTTAR PRADESH | Deoria | NS | NS | NS | NS | NS | NS | H | NS | NS | NS | NS | NS | NS |
| UTTAR PRADESH | Azamgarh | NS | NS | NS | NS | NS | NS | H | NS | NS | NS | NS | NS | NS |
| UTTAR PRADESH | Mau | NS | NS | NS | H | NS | NS | H | NS | NS | NS | NS | NS | NS |
| UTTAR PRADESH | Ballia | NS | NS | NS | NS | NS | NS | NS | H | NS | NS | NS | NS | NS |
| UTTAR PRADESH | Jaunpur | NS | NS | NS | NS | NS | NS | H | NS | H | NS | NS | NS | NS |
| UTTAR PRADESH | Ghazipur | NS | NS | NS | NS | NS | NS | H | NS | NS | NS | NS | NS | NS |
| UTTAR PRADESH | Chandauli | NS | NS | NS | NS | NS | NS | H | H | NS | NS | NS | NS | NS |
| UTTAR PRADESH | Varanasi | NS | NS | NS | NS | NS | NS | H | L | NS | NS | NS | NS | NS |
| UTTAR PRADESH | Sant Ravidas Nagar (Bhadohi) | NS | NS | NS | NS | NS | NS | H | NS | NS | NS | NS | NS | NS |
| UTTAR PRADESH | Mirzapur | NS | NS | NS | NS | NS | NS | H | NS | NS | NS | NS | NS | NS |
| UTTAR PRADESH | Sonbhadra | NS | NS | NS | NS | NS | NS | NS | NS | NS | NS | NS | NS | NS |
| UTTAR PRADESH | Etah | NS | NS | NS | NS | NS | NS | NS | L | NS | NS | NS | NS | NS |
| UTTAR PRADESH | Kanshiram Nagar | NS | NS | NS | NS | NS | NS | NS | H | NS | NS | NS | NS | NS |
| UTTAR PRADESH | Amethi | NS | NS | NS | NS | NS | NS | H | NS | NS | NS | NS | NS | NS |
| UTTAR PRADESH | Budaun | NS | NS | NS | NS | NS | NS | H | NS | NS | NS | NS | H | NS |
| UTTAR PRADESH | Ghaziabad | NS | NS | NS | NS | NS | NS | H | NS | NS | NS | NS | NS | NS |
| UTTAR PRADESH | Hapur | NS | NS | NS | NS | NS | NS | NS | NS | NS | NS | NS | NS | NS |
| UTTAR PRADESH | Moradabad | NS | NS | NS | NS | NS | H | NS | L | NS | NS | NS | NS | NS |
| UTTAR PRADESH | Muzaffarnagar | NS | NS | NS | NS | NS | NS | H | NS | NS | NS | NS | NS | NS |
| UTTAR PRADESH | Rae Bareli | NS | NS | NS | NS | NS | NS | H | NS | NS | NS | NS | NS | NS |
| UTTAR PRADESH | Sambhal | NS | NS | NS | NS | NS | NS | H | NS | NS | NS | NS | NS | NS |
| UTTAR PRADESH | Shamli | NS | NS | NS | NS | NS | NS | NS | NS | NS | NS | NS | NS | NS |
| UTTAR PRADESH | Sultanpur | NS | NS | NS | NS | NS | NS | H | NS | NS | NS | NS | NS | NS |
| UTTARAKHAND | Uttarkashi | NS | NS | NS | NS | NS | NS | NS | NS | NS | NS | NS | NS | NS |
| UTTARAKHAND | Chamoli | NS | NS | NS | NS | NS | NS | NS | NS | NS | NS | NS | NS | NS |
| UTTARAKHAND | Rudraprayag | NS | NS | NS | NS | NS | NS | NS | NS | NS | NS | NS | NS | NS |
| UTTARAKHAND | Tehri Garhwal | NS | NS | NS | NS | NS | NS | NS | NS | NS | NS | NS | NS | NS |
| UTTARAKHAND | Dehradun | NS | NS | NS | NS | NS | NS | NS | NS | NS | NS | NS | NS | NS |
| UTTARAKHAND | Garhwal | NS | NS | NS | NS | NS | NS | NS | NS | NS | NS | NS | NS | NS |
| UTTARAKHAND | Pithoragarh | NS | NS | NS | NS | NS | NS | NS | NS | NS | NS | NS | NS | NS |
| UTTARAKHAND | Bageshwar | NS | NS | NS | NS | NS | NS | NS | NS | NS | NS | NS | NS | NS |
| UTTARAKHAND | Almora | NS | NS | NS | NS | NS | NS | NS | NS | NS | NS | NS | NS | NS |
| UTTARAKHAND | Champawat | NS | NS | NS | NS | NS | NS | NS | NS | NS | NS | NS | NS | NS |
| UTTARAKHAND | Nainital | NS | NS | NS | NS | NS | NS | NS | NS | NS | NS | NS | NS | NS |
| UTTARAKHAND | Udham Singh Nagar | NS | H | H | NS | NS | NS | H | NS | NS | NS | NS | NS | NS |
| UTTARAKHAND | Hardwar | NS | NS | NS | NS | NS | NS | NS | NS | NS | NS | NS | NS | NS |
| WEST BENGAL | Darjiling | NS | NS | NS | NS | NS | NS | NS | NS | NS | NS | NS | NS | NS |
| WEST BENGAL | Jalpaiguri | NS | NS | H | NS | NS | NS | H | L | NS | NS | NS | H | NS |
| WEST BENGAL | Koch Bihar | NS | NS | NS | NS | NS | NS | H | H | NS | NS | NS | NS | NS |
| WEST BENGAL | Uttar Dinajpur | NS | H | L | NS | NS | NS | NS | NS | NS | NS | NS | NS | NS |
| WEST BENGAL | Dakshin Dinajpur | H | NS | H | NS | NS | NS | H | NS | NS | NS | NS | NS | NS |
| WEST BENGAL | Maldah | NS | H | NS | NS | NS | H | H | H | NS | NS | NS | NS | NS |
| WEST BENGAL | Murshidabad | NS | NS | NS | L | NS | NS | H | NS | NS | NS | NS | H | NS |
| WEST BENGAL | Birbhum | NS | H | NS | NS | NS | H | NS | NS | NS | H | NS | NS | NS |
| WEST BENGAL | Nadia | H | NS | NS | NS | NS | H | NS | NS | NS | NS | NS | H | NS |
| WEST BENGAL | North Twenty Four Parganas | H | NS | NS | NS | NS | H | H | L | L | NS | H | NS | NS |
| WEST BENGAL | Hugli | NS | NS | H | NS | NS | H | NS | H | NS | NS | NS | NS | NS |
| WEST BENGAL | Bankura | H | NS | NS | NS | NS | NS | NS | NS | NS | NS | NS | NS | NS |
| WEST BENGAL | Puruliya | H | NS | H | NS | NS | NS | NS | NS | NS | NS | NS | NS | NS |
| WEST BENGAL | Haora | NS | H | NS | H | NS | NS | H | L | NS | NS | L | H | H |
| WEST BENGAL | Kolkata | NS | NS | NS | NS | L | NS | H | NS | NS | NS | NS | NS | NS |
| WEST BENGAL | South Twenty Four Parganas | NS | H | NS | H | H | NS | H | L | NS | L | H | L | NS |
| WEST BENGAL | Paschim Medinipur | H | H | NS | NS | H | NS | NS | H | NS | H | NS | NS | NS |
| WEST BENGAL | Purba Medinipur | H | NS | NS | L | H | NS | H | NS | NS | NS | NS | NS | NS |
| WEST BENGAL | Paschim Barddhaman | NS | NS | NS | NS | NS | NS | H | NS | NS | NS | NS | H | NS |
| WEST BENGAL | Purba Barddhaman | H | NS | NS | NS | NS | H | H | NS | H | NS | NS | NS | NS |
| **H%** |  | 8.3 | 12.8 | 3.6 | 3.3 | 4.9 | 5.3 | 28.5 | 9.7 | 2.8 | 1.9 | 4.1 | 5.0 | 0.3 |
| **L%** |  | 0.3 | 0.0 | 0.3 | 0.8 | 0.9 | 0.5 | 0.2 | 3.6 | 1.3 | 1.1 | 0.9 | 0.5 | 0.5 |

H: Higher odds [Adjusted odds ratio(AOR) >1 and P<0.05], L: Lower odds (AOR<1 and P<0.05), NS: Not Significant (p>0.05), NA: Data Not Available.

Districts from 11 Non significant states excluded from table (ie., Andaman & Nicobar Islands, Arunachal Pradesh, Chandigarh, Dadra & Nagar Haveli And Daman & Diu, Ladakh, Lakshadweep, Meghalaya, Mizoram, Nagaland, Puducherry, Sikkim).

## **Table S11: District wise determinants of Prehypertension among adults in India(AOR) (National Family Health Survey-5, India, 2019-2021)**

|  |  | Age Group (>=30) | Sex (Female) | Education (Literate) | Occupation (Employed) | Household wealth quintile (middle) | Household wealth quintile (richer/richest) | Marital status (Currently married) | Place Of Residence (Urban) | Tobacco consumption (Yes) | Alcohol consumption (Yes) | BMI (Overweight/Obese) | Central obesity (Present) | Blood glucose level (Raised) |
| --- | --- | --- | --- | --- | --- | --- | --- | --- | --- | --- | --- | --- | --- | --- |
| ANDHRA PRADESH | SRIKAKULAM | **H** | **L** | **NS** | **NS** | **NS** | **NS** | **NS** | **NS** | **L** | **NS** | **NS** | **NS** | **NS** |
| ANDHRA PRADESH | VIZIANAGARAM | **NS** | **NS** | **H** | **H** | **NS** | **NS** | **NS** | **NS** | **NS** | **NS** | **H** | **NS** | **NS** |
| ANDHRA PRADESH | VISAKHAPATNAM | **NS** | **L** | **L** | **L** | **NS** | **H** | **NS** | **NS** | **NS** | **L** | **NS** | **NS** | **NA** |
| ANDHRA PRADESH | EAST GODAVARI | **NS** | **L** | **NS** | **L** | **NS** | **NS** | **NS** | **NS** | **NS** | **NS** | **L** | **H** | **NS** |
| ANDHRA PRADESH | WEST GODAVARI | **L** | **L** | **L** | **NS** | **NS** | **NS** | **L** | **NS** | **NS** | **NS** | **NS** | **NS** | **NS** |
| ANDHRA PRADESH | KRISHNA | **NS** | **NS** | **NS** | **H** | **NS** | **NS** | **NS** | **NS** | **NS** | **NS** | **NS** | **NS** | **NS** |
| ANDHRA PRADESH | GUNTUR | **H** | **NS** | **NS** | **NS** | **NS** | **NS** | **L** | **L** | **L** | **NS** | **NS** | **L** | **NS** |
| ANDHRA PRADESH | PRAKASAM | **H** | **L** | **NS** | **NS** | **NS** | **NS** | **L** | **L** | **NS** | **L** | **H** | **NS** | **H** |
| ANDHRA PRADESH | SRI POTTI SRIRAMULU NELLORE | **H** | **L** | **NS** | **NS** | **NS** | **NS** | **L** | **NS** | **H** | **L** | **NS** | **H** | **NS** |
| ANDHRA PRADESH | Y.S.R. | **NS** | **L** | **NS** | **NS** | **H** | **H** | **NS** | **NS** | **NS** | **NS** | **NS** | **H** | **NS** |
| ANDHRA PRADESH | KURNOOL | **NS** | **L** | **L** | **H** | **NS** | **NS** | **L** | **NS** | **NS** | **NS** | **H** | **NS** | **H** |
| ANDHRA PRADESH | ANANTAPUR | **NS** | **NS** | **NS** | **H** | **NS** | **NS** | **L** | **NS** | **NS** | **NS** | **NS** | **NS** | **NS** |
| ANDHRA PRADESH | CHITTOOR | **NS** | **L** | **NS** | **L** | **H** | **NS** | **NS** | **H** | **H** | **L** | **H** | **NS** | **NS** |
| ASSAM | KOKRAJHAR | **NS** | **NS** | **NS** | **NS** | **NS** | **NS** | **NS** | **NS** | **NS** | **NS** | **NS** | **NS** | **NS** |
| ASSAM | GOALPARA | **NS** | **NS** | **NS** | **NS** | **NS** | **NS** | **NS** | **NS** | **NS** | **NS** | **H** | **NS** | **NS** |
| ASSAM | BARPETA | **NS** | **NS** | **NS** | **NS** | **NS** | **NS** | **NS** | **NS** | **NS** | **NS** | **NS** | **NS** | **NS** |
| ASSAM | MORIGAON | **NS** | **L** | **NS** | **NS** | **NS** | **NS** | **NS** | **NS** | **NS** | **NS** | **NS** | **NS** | **NS** |
| ASSAM | LAKHIMPUR | **NS** | **NS** | **NS** | **H** | **NS** | **NS** | **NS** | **NS** | **NS** | **NS** | **NS** | **NS** | **NS** |
| ASSAM | DHEMAJI | **NS** | **NS** | **NS** | **NS** | **NS** | **NS** | **NS** | **NS** | **NS** | **NS** | **NS** | **NS** | **NS** |
| ASSAM | TINSUKIA | **NS** | **NS** | **NS** | **NS** | **NS** | **NS** | **NS** | **NS** | **NS** | **NS** | **NS** | **NS** | **NS** |
| ASSAM | DIBRUGARH | **NS** | **L** | **NS** | **NS** | **NS** | **NS** | **NS** | **NS** | **NS** | **NS** | **NS** | **NS** | **NS** |
| ASSAM | GOLAGHAT | **NS** | **NS** | **NS** | **NS** | **NS** | **NS** | **NS** | **NS** | **NS** | **NS** | **NS** | **NS** | **NS** |
| ASSAM | DIMA HASAO | **NS** | **NS** | **NS** | **NS** | **NS** | **NS** | **NS** | **NS** | **NS** | **NS** | **NS** | **NS** | **NS** |
| ASSAM | CACHAR | **NS** | **L** | **NS** | **NS** | **NS** | **NS** | **NS** | **NS** | **NS** | **NS** | **NS** | **NS** | **NS** |
| ASSAM | KARIMGANJ | **NS** | **NS** | **NS** | **NS** | **NS** | **NS** | **NS** | **NS** | **NS** | **NS** | **NS** | **NS** | **NS** |
| ASSAM | HAILAKANDI | **NS** | **NS** | **NS** | **NS** | **NS** | **NS** | **NS** | **NS** | **L** | **NS** | **NS** | **NS** | **NS** |
| ASSAM | BONGAIGAON | **NS** | **NS** | **NS** | **NS** | **NS** | **NS** | **NS** | **NS** | **NS** | **NS** | **NS** | **NS** | **NS** |
| ASSAM | CHIRANG | **NS** | **NS** | **NS** | **NS** | **NS** | **NS** | **NS** | **NS** | **NS** | **NS** | **NS** | **NS** | **NS** |
| ASSAM | KAMRUP | **NS** | **NS** | **NS** | **NS** | **NS** | **NS** | **NS** | **NS** | **NS** | **NS** | **H** | **NS** | **NS** |
| ASSAM | KAMRUP METROPOLITAN | **NS** | **NS** | **NS** | **NS** | **NS** | **NS** | **NS** | **NS** | **NS** | **NS** | **NS** | **NS** | **NS** |
| ASSAM | NALBARI | **NS** | **NS** | **NS** | **NS** | **NS** | **NS** | **NS** | **NS** | **NS** | **NS** | **NS** | **NS** | **NS** |
| ASSAM | BAKSA | **NS** | **NS** | **NS** | **NS** | **NS** | **NS** | **NS** | **NA** | **NS** | **NS** | **NS** | **NS** | **NS** |
| ASSAM | DARRANG | **NS** | **NS** | **NS** | **NS** | **NS** | **NS** | **NS** | **NS** | **NS** | **NS** | **NS** | **NS** | **NS** |
| ASSAM | UDALGURI | **NS** | **NS** | **NS** | **NS** | **NS** | **NS** | **NS** | **NS** | **NS** | **NS** | **NS** | **NS** | **NS** |
| ASSAM | BISWANATH | **NS** | **NS** | **NS** | **NS** | **NS** | **NS** | **NS** | **NS** | **NS** | **NS** | **NS** | **NS** | **NS** |
| ASSAM | CHARAIDEO | **NS** | **NS** | **NS** | **NS** | **NS** | **NS** | **NS** | **NS** | **NS** | **NS** | **NS** | **NS** | **NS** |
| ASSAM | DHUBRI | **NS** | **H** | **NS** | **NS** | **NS** | **NS** | **NS** | **NS** | **NS** | **NS** | **NS** | **NS** | **NS** |
| ASSAM | HOJAI | **NS** | **NS** | **NS** | **NS** | **NS** | **NS** | **NS** | **NS** | **NS** | **NS** | **NS** | **NS** | **NS** |
| ASSAM | JORHAT | **NS** | **NS** | **NS** | **L** | **NS** | **NS** | **H** | **NS** | **NS** | **NS** | **NS** | **NS** | **NS** |
| ASSAM | KARBI ANGLONG | **NS** | **NS** | **NS** | **NS** | **NS** | **NS** | **NS** | **NS** | **NS** | **NS** | **NS** | **NS** | **NS** |
| ASSAM | MAJULI | **NS** | **NS** | **NS** | **NS** | **NS** | **NS** | **NS** | **NA** | **NS** | **NS** | **NS** | **NS** | **NS** |
| ASSAM | NAGAON | **H** | **L** | **NS** | **NS** | **NS** | **L** | **NS** | **NS** | **L** | **NS** | **NS** | **H** | **NS** |
| ASSAM | SIVASAGAR | **NS** | **NS** | **NS** | **NS** | **NS** | **NS** | **NS** | **NS** | **NS** | **NS** | **NS** | **NS** | **NS** |
| ASSAM | SONITPUR | **NS** | **L** | **NS** | **NS** | **NS** | **NS** | **NS** | **NS** | **NS** | **NS** | **NS** | **NS** | **NS** |
| ASSAM | SOUTH SALMARA MANCACHAR | **NS** | **NS** | **NS** | **NS** | **NS** | **NS** | **NS** | **NS** | **NS** | **NS** | **NS** | **NS** | **NS** |
| ASSAM | WEST KARBI ANGLONG | **NS** | **NS** | **NS** | **NS** | **NS** | **NS** | **NS** | **NS** | **NS** | **NS** | **NS** | **NS** | **NS** |
| BIHAR | PASHCHIM CHAMPARAN | **NS** | **L** | **L** | **NS** | **NS** | **NS** | **NS** | **L** | **NS** | **NS** | **NS** | **L** | **NS** |
| BIHAR | PURBA CHAMPARAN | **H** | **L** | **L** | **NS** | **L** | **NS** | **L** | **H** | **NS** | **L** | **H** | **H** | **NA** |
| BIHAR | SHEOHAR | **NS** | **NS** | **NS** | **NS** | **NS** | **NS** | **NS** | **NS** | **NS** | **NS** | **NS** | **NS** | **NS** |
| BIHAR | SITAMARHI | **NS** | **NS** | **NS** | **H** | **NS** | **NS** | **NS** | **NS** | **NS** | **H** | **NS** | **NS** | **NS** |
| BIHAR | MADHUBANI | **NS** | **L** | **L** | **L** | **NS** | **NS** | **NS** | **H** | **NS** | **NS** | **H** | **NS** | **NS** |
| BIHAR | SUPAUL | **NS** | **L** | **NS** | **NS** | **NS** | **NS** | **H** | **NS** | **NS** | **NS** | **NS** | **NS** | **NS** |
| BIHAR | ARARIA | **NS** | **L** | **NS** | **NS** | **NS** | **NS** | **NS** | **NS** | **NS** | **NS** | **H** | **NS** | **NS** |
| BIHAR | KISHANGANJ | **H** | **L** | **NS** | **NS** | **NS** | **NA** | **NS** | **NS** | **NS** | **NS** | **H** | **NS** | **NS** |
| BIHAR | PURNIA | **H** | **L** | **NS** | **NS** | **NS** | **NS** | **NS** | **NS** | **NS** | **H** | **NS** | **H** | **NA** |
| BIHAR | KATIHAR | **NS** | **L** | **NS** | **NS** | **NS** | **NS** | **NS** | **NS** | **NS** | **NS** | **NS** | **NS** | **NS** |
| BIHAR | MADHEPURA | **NS** | **NS** | **NS** | **NS** | **H** | **NS** | **NS** | **NS** | **NS** | **NS** | **NS** | **NS** | **NS** |
| BIHAR | SAHARSA | **NS** | **NS** | **NS** | **NS** | **L** | **NS** | **NS** | **H** | **H** | **NS** | **H** | **L** | **NS** |
| BIHAR | DARBHANGA | **NS** | **NS** | **NS** | **NS** | **NS** | **L** | **NS** | **NS** | **L** | **NS** | **H** | **NS** | **NS** |
| BIHAR | MUZAFFARPUR | **H** | **NS** | **NS** | **L** | **NS** | **H** | **NS** | **L** | **H** | **H** | **H** | **H** | **NS** |
| BIHAR | GOPALGANJ | **NS** | **L** | **NS** | **NS** | **NS** | **NS** | **NS** | **L** | **L** | **NS** | **NS** | **NS** | **NS** |
| BIHAR | SIWAN | **NS** | **L** | **NS** | **NS** | **NS** | **NS** | **NS** | **NS** | **L** | **NS** | **NS** | **NS** | **NS** |
| BIHAR | SARAN | **NS** | **NS** | **NS** | **NS** | **NS** | **NS** | **NS** | **L** | **NS** | **NS** | **NS** | **NS** | **NS** |
| BIHAR | VAISHALI | **NS** | **L** | **NS** | **NS** | **NS** | **NS** | **NS** | **NS** | **NS** | **NS** | **NS** | **H** | **NS** |
| BIHAR | SAMASTIPUR | **H** | **L** | **NS** | **L** | **NS** | **L** | **NS** | **NS** | **L** | **NS** | **H** | **H** | **NS** |
| BIHAR | BEGUSARAI | **H** | **L** | **H** | **L** | **NS** | **NS** | **NS** | **NS** | **NS** | **H** | **L** | **NS** | **NS** |
| BIHAR | KHAGARIA | **NS** | **L** | **L** | **H** | **NS** | **NS** | **NS** | **NS** | **NS** | **NS** | **NS** | **NS** | **NS** |
| BIHAR | BHAGALPUR | **H** | **L** | **NS** | **NS** | **NS** | **NS** | **NS** | **NS** | **L** | **H** | **H** | **NS** | **NS** |
| BIHAR | BANKA | **NS** | **L** | **NS** | **NS** | **NS** | **NS** | **NS** | **NS** | **NS** | **NS** | **H** | **NS** | **NS** |
| BIHAR | MUNGER | **NS** | **L** | **NS** | **NS** | **NS** | **NS** | **NS** | **NS** | **NS** | **NS** | **H** | **NS** | **NS** |
| BIHAR | LAKHISARAI | **NS** | **NS** | **NS** | **NS** | **NS** | **H** | **NS** | **NS** | **NS** | **NS** | **NS** | **NS** | **NS** |
| BIHAR | SHEIKHPURA | **NS** | **NS** | **NS** | **NS** | **NS** | **NS** | **NS** | **NS** | **NS** | **NS** | **NS** | **NS** | **NS** |
| BIHAR | NALANDA | **NS** | **L** | **NS** | **NS** | **NS** | **NS** | **NS** | **L** | **L** | **H** | **NS** | **H** | **NS** |
| BIHAR | PATNA | **L** | **L** | **L** | **NS** | **NS** | **H** | **NS** | **L** | **NS** | **L** | **NS** | **H** | **NS** |
| BIHAR | BHOJPUR | **H** | **L** | **NS** | **NS** | **H** | **NS** | **NS** | **NS** | **L** | **NS** | **H** | **NS** | **NS** |
| BIHAR | BUXAR | **NS** | **NS** | **NS** | **NS** | **H** | **NS** | **NS** | **H** | **NS** | **NS** | **H** | **NS** | **NS** |
| BIHAR | KAIMUR (BHABUA) | **H** | **NS** | **NS** | **NS** | **NS** | **NS** | **NS** | **NS** | **NS** | **NS** | **NS** | **NS** | **NS** |
| BIHAR | ROHTAS | **NS** | **L** | **H** | **NS** | **NS** | **NS** | **NS** | **NS** | **L** | **NS** | **H** | **NS** | **NS** |
| BIHAR | AURANGABAD | **NS** | **NS** | **NS** | **NS** | **NS** | **NS** | **NS** | **NS** | **NS** | **NS** | **NS** | **NS** | **NS** |
| BIHAR | GAYA | **H** | **NS** | **NS** | **NS** | **H** | **H** | **NS** | **NS** | **NS** | **H** | **H** | **NS** | **NS** |
| BIHAR | NAWADA | **NS** | **L** | **NS** | **NS** | **NS** | **NS** | **H** | **NS** | **L** | **NS** | **H** | **NS** | **NS** |
| BIHAR | JAMUI | **H** | **NS** | **NS** | **NS** | **NS** | **NS** | **NS** | **NS** | **NS** | **NS** | **NS** | **L** | **NS** |
| BIHAR | JEHANABAD | **NS** | **NS** | **NS** | **NS** | **NS** | **NS** | **NS** | **NS** | **NS** | **NS** | **NS** | **NS** | **NS** |
| BIHAR | ARWAL | **NS** | **NS** | **NS** | **NS** | **NS** | **NS** | **NS** | **NS** | **NS** | **NS** | **NS** | **NS** | **NS** |
| CHANDIGARH | CHANDIGARH | **NS** | **L** | **NS** | **NS** | **NS** | **NS** | **NS** | **NA** | **NS** | **NS** | **NS** | **NS** | **NS** |
| CHHATTISGARH | KORIYA | **NS** | **NS** | **NS** | **NS** | **NS** | **NS** | **NS** | **NS** | **NS** | **NS** | **NS** | **NS** | **NS** |
| CHHATTISGARH | JASHPUR | **NS** | **NS** | **NS** | **NS** | **NS** | **NS** | **NS** | **NS** | **NS** | **NS** | **NS** | **NS** | **NS** |
| CHHATTISGARH | RAIGARH | **L** | **NS** | **NS** | **H** | **NS** | **NS** | **NS** | **NS** | **NS** | **NS** | **H** | **NS** | **NS** |
| CHHATTISGARH | KORBA | **NS** | **NS** | **NS** | **H** | **NS** | **NS** | **NS** | **NS** | **NS** | **NS** | **NS** | **NS** | **NS** |
| CHHATTISGARH | JANJGIR - CHAMPA | **NS** | **NS** | **NS** | **NS** | **NS** | **NS** | **NS** | **NS** | **NS** | **NS** | **H** | **NS** | **NS** |
| CHHATTISGARH | KABEERDHAM | **NS** | **NS** | **NS** | **NS** | **NS** | **NS** | **NS** | **NS** | **NS** | **NS** | **NS** | **NS** | **NS** |
| CHHATTISGARH | RAJNANDGAON | **NS** | **NS** | **NS** | **NS** | **NS** | **NS** | **NS** | **NS** | **NS** | **NS** | **NS** | **NS** | **NS** |
| CHHATTISGARH | MAHASAMUND | **NS** | **NS** | **NS** | **NS** | **NS** | **NS** | **NS** | **NS** | **NS** | **NS** | **NS** | **NS** | **NS** |
| CHHATTISGARH | DHAMTARI | **NS** | **NS** | **NS** | **NS** | **NS** | **NS** | **NS** | **NS** | **NS** | **NS** | **NS** | **NS** | **NS** |
| CHHATTISGARH | UTTAR BASTAR KANKER | **NS** | **NS** | **NS** | **NS** | **NS** | **NS** | **NS** | **NS** | **NS** | **NS** | **NS** | **NS** | **NS** |
| CHHATTISGARH | NARAYANPUR | **NS** | **NS** | **NS** | **NS** | **NS** | **NS** | **NS** | **NS** | **NS** | **NS** | **NS** | **NS** | **NS** |
| CHHATTISGARH | BIJAPUR | **NS** | **NS** | **NS** | **NS** | **NS** | **NS** | **NS** | **NS** | **NS** | **NS** | **NS** | **NS** | **NS** |
| CHHATTISGARH | BALOD | **NS** | **NS** | **NS** | **NS** | **NS** | **NS** | **NS** | **NS** | **NS** | **NS** | **NS** | **NS** | **NS** |
| CHHATTISGARH | BALODA BAZAR | **NS** | **NS** | **NS** | **NS** | **NS** | **NS** | **NS** | **NS** | **NS** | **NS** | **NS** | **NS** | **NS** |
| CHHATTISGARH | BALRAMPUR | **NS** | **NS** | **NS** | **NS** | **NS** | **NS** | **NS** | **NS** | **NS** | **NS** | **NS** | **NS** | **NS** |
| CHHATTISGARH | BASTAR | **NS** | **NS** | **NS** | **NS** | **NS** | **NS** | **NS** | **NS** | **NS** | **NS** | **NS** | **NS** | **NS** |
| CHHATTISGARH | BEMETARA | **NS** | **NS** | **NS** | **NS** | **NS** | **NS** | **NS** | **NS** | **NS** | **NS** | **NS** | **NS** | **NA** |
| CHHATTISGARH | BILASPUR | **NS** | **NS** | **NS** | **NS** | **NS** | **NS** | **NS** | **NS** | **NS** | **NS** | **L** | **NS** | **NS** |
| CHHATTISGARH | DANTEWADA | **NS** | **NS** | **NS** | **NS** | **NS** | **NS** | **NS** | **NS** | **NS** | **NS** | **NS** | **NS** | **NS** |
| CHHATTISGARH | DURG | **NS** | **NS** | **NS** | **NS** | **NS** | **NS** | **NS** | **NS** | **NS** | **NS** | **H** | **L** | **NS** |
| CHHATTISGARH | GARIYABAND | **NS** | **NS** | **NS** | **NS** | **NS** | **NS** | **NS** | **NS** | **NS** | **NS** | **NS** | **NS** | **NS** |
| CHHATTISGARH | KODAGAON | **NS** | **NS** | **NS** | **NS** | **NS** | **NS** | **NS** | **NS** | **NS** | **NS** | **NS** | **NS** | **NS** |
| CHHATTISGARH | MUNGELI | **NS** | **NS** | **NS** | **NS** | **NS** | **NS** | **NS** | **NS** | **NS** | **NS** | **NS** | **NS** | **NS** |
| CHHATTISGARH | RAIPUR | **NS** | **NS** | **L** | **NS** | **NS** | **NS** | **NS** | **NS** | **NS** | **NS** | **NS** | **NS** | **NS** |
| CHHATTISGARH | SUKMA | **NS** | **NS** | **NS** | **NS** | **NS** | **NS** | **NS** | **NS** | **NS** | **NS** | **NS** | **NS** | **NS** |
| CHHATTISGARH | SURAJPUR | **NS** | **NS** | **NS** | **NS** | **NS** | **NS** | **NS** | **NS** | **NS** | **NS** | **NS** | **NS** | **NS** |
| CHHATTISGARH | SURGUJA | **NS** | **NS** | **NS** | **NS** | **NS** | **NS** | **NS** | **NS** | **NS** | **NS** | **NS** | **NS** | **NS** |
| GOA | NORTH GOA | **NS** | **NS** | **NS** | **NS** | **NS** | **NS** | **NS** | **NS** | **NS** | **NS** | **NS** | **NS** | **NS** |
| GOA | SOUTH GOA | **NS** | **NS** | **NS** | **NS** | **NS** | **NS** | **NS** | **NS** | **H** | **NS** | **NS** | **NS** | **NS** |
| GUJARAT | KACHCHH | **L** | **L** | **NS** | **NS** | **NS** | **L** | **NS** | **NS** | **NS** | **NS** | **H** | **NS** | **NS** |
| GUJARAT | BANAS KANTHA | **H** | **NS** | **NS** | **H** | **L** | **NS** | **L** | **NS** | **NS** | **NS** | **NS** | **H** | **NA** |
| GUJARAT | PATAN | **NS** | **NS** | **NS** | **NS** | **NS** | **NS** | **NS** | **NS** | **NS** | **NS** | **H** | **NS** | **NS** |
| GUJARAT | MAHESANA | **NS** | **L** | **NS** | **NS** | **NS** | **NS** | **NS** | **L** | **NS** | **NS** | **NS** | **NS** | **NS** |
| GUJARAT | GANDHINAGAR | **NS** | **L** | **L** | **NS** | **NS** | **NS** | **NS** | **NS** | **NS** | **H** | **NS** | **NS** | **NS** |
| GUJARAT | PORBANDAR | **H** | **NS** | **NS** | **NS** | **NS** | **NS** | **NS** | **NS** | **NS** | **NS** | **NS** | **NS** | **NS** |
| GUJARAT | AMRELI | **NS** | **NS** | **NS** | **H** | **NS** | **NS** | **NS** | **NS** | **NS** | **NA** | **NS** | **H** | **NS** |
| GUJARAT | ANAND | **NS** | **NS** | **NS** | **NS** | **NS** | **NS** | **NS** | **NS** | **NS** | **NS** | **NS** | **NS** | **NS** |
| GUJARAT | DOHAD | **NS** | **NS** | **NS** | **NS** | **NS** | **NS** | **NS** | **NS** | **NS** | **NS** | **NS** | **NS** | **NS** |
| GUJARAT | NARMADA | **H** | **NS** | **NS** | **NS** | **NS** | **NS** | **NS** | **NS** | **NS** | **NS** | **NS** | **NS** | **NS** |
| GUJARAT | BHARUCH | **NS** | **NS** | **L** | **NS** | **NS** | **NS** | **NS** | **NS** | **NS** | **NS** | **NS** | **NS** | **NS** |
| GUJARAT | THE DANGS | **NS** | **NS** | **NS** | **NS** | **NS** | **NS** | **NS** | **NS** | **NS** | **NS** | **NS** | **NS** | **NS** |
| GUJARAT | NAVSARI | **H** | **L** | **NS** | **NS** | **NS** | **NS** | **NS** | **NS** | **NS** | **NS** | **NS** | **NS** | **NS** |
| GUJARAT | VALSAD | **NS** | **NS** | **NS** | **NS** | **NS** | **NS** | **NS** | **NS** | **NS** | **NS** | **NS** | **NS** | **NS** |
| GUJARAT | SURAT | **H** | **L** | **NS** | **NS** | **NS** | **NS** | **NS** | **NS** | **NS** | **L** | **H** | **NS** | **L** |
| GUJARAT | TAPI | **NS** | **NS** | **NS** | **NS** | **NS** | **NS** | **NS** | **NS** | **NS** | **NS** | **NS** | **NS** | **NS** |
| GUJARAT | AHMADABAD | **H** | **L** | **H** | **NS** | **L** | **L** | **L** | **L** | **L** | **H** | **H** | **H** | **NS** |
| GUJARAT | ARAVALI | **NS** | **NS** | **NS** | **NS** | **H** | **NS** | **NS** | **NS** | **NS** | **NS** | **NS** | **NS** | **NS** |
| GUJARAT | BHAVNAGAR | **H** | **L** | **NS** | **L** | **NS** | **NS** | **NS** | **NS** | **NS** | **NS** | **H** | **NS** | **NS** |
| GUJARAT | BOTAD | **NS** | **L** | **NS** | **NS** | **NS** | **NS** | **NS** | **NS** | **NS** | **NS** | **NS** | **NS** | **NS** |
| GUJARAT | CHHOTA UDAIPUR | **NS** | **NS** | **NS** | **NS** | **NS** | **NS** | **NS** | **NS** | **NS** | **NS** | **NS** | **NS** | **NS** |
| GUJARAT | DEVBHUMI DWARKA | **NS** | **NS** | **NS** | **NS** | **NS** | **NS** | **NS** | **H** | **NS** | **NS** | **NS** | **NS** | **NS** |
| GUJARAT | GIR SOMNATH | **NS** | **NS** | **NS** | **NS** | **NS** | **NS** | **NS** | **NS** | **NS** | **NS** | **NS** | **NS** | **NS** |
| GUJARAT | JAMNAGAR | **H** | **NS** | **NS** | **NS** | **NS** | **NS** | **NS** | **NS** | **NS** | **NS** | **NS** | **L** | **NS** |
| GUJARAT | JUNAGADH | **NS** | **NS** | **NS** | **NS** | **NS** | **NS** | **NS** | **H** | **NS** | **NS** | **H** | **NS** | **L** |
| GUJARAT | KHEDA | **NS** | **NS** | **NS** | **NS** | **NS** | **NS** | **NS** | **NS** | **NS** | **NS** | **NS** | **NS** | **NS** |
| GUJARAT | MAHISAGAR | **NS** | **NS** | **NS** | **NS** | **NS** | **NS** | **NS** | **NS** | **NS** | **NS** | **NS** | **NS** | **NS** |
| GUJARAT | MORBI | **NS** | **NS** | **NS** | **NS** | **NS** | **NS** | **NS** | **NS** | **NS** | **NS** | **NS** | **NS** | **NS** |
| GUJARAT | PANCH MAHALS | **H** | **NS** | **NS** | **NS** | **NS** | **NS** | **NS** | **NS** | **NS** | **NS** | **NS** | **NS** | **NS** |
| GUJARAT | RAJKOT | **H** | **NS** | **NS** | **H** | **NS** | **NS** | **NS** | **L** | **NS** | **NS** | **H** | **NS** | **NS** |
| GUJARAT | SABAR KANTHA | **NS** | **NS** | **NS** | **NS** | **NS** | **NS** | **NS** | **NS** | **NS** | **NS** | **NS** | **NS** | **NA** |
| GUJARAT | SURENDRANAGAR | **H** | **NS** | **NS** | **NS** | **NS** | **NS** | **NS** | **NS** | **NS** | **NA** | **NS** | **NS** | **NS** |
| GUJARAT | VADODARA | **H** | **L** | **NS** | **NS** | **L** | **NS** | **H** | **NS** | **L** | **NS** | **NS** | **L** | **NS** |
| HARYANA | PANCHKULA | **NS** | **NS** | **NS** | **NS** | **NS** | **NA** | **NS** | **NS** | **NS** | **NS** | **NS** | **NS** | **NS** |
| HARYANA | AMBALA | **NS** | **NS** | **NS** | **NS** | **NS** | **NS** | **NS** | **NS** | **NS** | **NS** | **NS** | **NS** | **NS** |
| HARYANA | YAMUNANAGAR | **NS** | **NS** | **NS** | **NS** | **NS** | **NS** | **NS** | **NS** | **NS** | **NS** | **NS** | **NS** | **NS** |
| HARYANA | KURUKSHETRA | **NS** | **NS** | **NS** | **NS** | **NS** | **NS** | **NS** | **NS** | **NS** | **NS** | **NS** | **NS** | **NS** |
| HARYANA | KAITHAL | **NS** | **NS** | **NS** | **NS** | **NS** | **NS** | **NS** | **NS** | **NS** | **NS** | **NS** | **NS** | **NS** |
| HARYANA | KARNAL | **H** | **L** | **NS** | **NS** | **NS** | **NS** | **NS** | **NS** | **NS** | **NS** | **NS** | **NS** | **NS** |
| HARYANA | PANIPAT | **NS** | **L** | **NS** | **NS** | **NS** | **NS** | **NS** | **NS** | **NS** | **NS** | **NS** | **NS** | **NS** |
| HARYANA | SONIPAT | **NS** | **L** | **L** | **NS** | **NS** | **NS** | **NS** | **NS** | **NS** | **NS** | **NS** | **NS** | **NS** |
| HARYANA | JIND | **NS** | **NS** | **NS** | **NS** | **NS** | **NS** | **NS** | **NS** | **NS** | **NS** | **NS** | **NS** | **NS** |
| HARYANA | FATEHABAD | **NS** | **NS** | **NS** | **NS** | **NS** | **NS** | **NS** | **NS** | **NS** | **NS** | **NS** | **NS** | **NA** |
| HARYANA | SIRSA | **NS** | **NS** | **NS** | **L** | **NS** | **NS** | **NS** | **NA** | **NS** | **NS** | **NS** | **NS** | **NS** |
| HARYANA | HISAR | **NS** | **NS** | **NS** | **NS** | **NS** | **NS** | **NS** | **NS** | **NS** | **NS** | **NS** | **NS** | **NS** |
| HARYANA | ROHTAK | **NS** | **NS** | **NS** | **NS** | **NS** | **NS** | **NS** | **NS** | **NS** | **NS** | **NS** | **NS** | **NS** |
| HARYANA | JHAJJAR | **NS** | **NS** | **NS** | **NS** | **NS** | **NS** | **NS** | **NS** | **NS** | **NS** | **NS** | **NS** | **NS** |
| HARYANA | MAHENDRAGARH | **NS** | **NS** | **NS** | **NS** | **NS** | **NS** | **NS** | **NS** | **NS** | **NS** | **NS** | **NS** | **NS** |
| HARYANA | REWARI | **NS** | **NS** | **NS** | **NS** | **NS** | **NS** | **NS** | **NS** | **NS** | **NS** | **NS** | **NS** | **NS** |
| HARYANA | GURGAON | **NS** | **NS** | **NS** | **NS** | **NS** | **NS** | **NS** | **NS** | **NS** | **NS** | **NS** | **NS** | **NS** |
| HARYANA | MEWAT | **NS** | **NS** | **NS** | **NS** | **NS** | **NS** | **NS** | **NS** | **NS** | **NS** | **NS** | **NS** | **NS** |
| HARYANA | FARIDABAD | **NS** | **NS** | **NS** | **NS** | **NS** | **NS** | **NS** | **NS** | **NS** | **NS** | **NS** | **NS** | **NS** |
| HARYANA | PALWAL | **NS** | **NS** | **NS** | **NS** | **NS** | **NS** | **NS** | **NS** | **NS** | **NS** | **NS** | **NS** | **NS** |
| HARYANA | BHIWANI | **NS** | **NS** | **NS** | **NS** | **NS** | **NS** | **NS** | **NS** | **NS** | **NS** | **NS** | **NS** | **NS** |
| HARYANA | CHARKHI DADRI | **NS** | **NS** | **NS** | **NS** | **NS** | **NS** | **NS** | **NS** | **NS** | **NS** | **NS** | **NS** | **NS** |
| HIMACHAL PRADESH | CHAMBA | **NS** | **NS** | **NS** | **NS** | **NS** | **NS** | **NS** | **NS** | **NS** | **NS** | **NS** | **NS** | **NA** |
| HIMACHAL PRADESH | KANGRA | **NS** | **NS** | **NS** | **NS** | **NS** | **NS** | **NS** | **NS** | **NS** | **NS** | **NS** | **L** | **NS** |
| HIMACHAL PRADESH | LAHUL & SPITI | **NS** | **NS** | **NS** | **NS** | **NS** | **NS** | **NS** | **NA** | **NS** | **NS** | **NS** | **NS** | **NA** |
| HIMACHAL PRADESH | KULLU | **NS** | **NS** | **NS** | **NS** | **NS** | **NS** | **NS** | **NS** | **NS** | **NS** | **NS** | **NS** | **NS** |
| HIMACHAL PRADESH | MANDI | **NS** | **NS** | **NS** | **NS** | **H** | **NS** | **NS** | **NS** | **NS** | **NS** | **NS** | **NS** | **NS** |
| HIMACHAL PRADESH | HAMIRPUR | **NS** | **NS** | **NS** | **NS** | **NS** | **NS** | **NS** | **NS** | **NS** | **NS** | **NS** | **NS** | **NS** |
| HIMACHAL PRADESH | UNA | **NS** | **NS** | **NS** | **NS** | **NS** | **NS** | **NS** | **NS** | **NS** | **NS** | **NS** | **NS** | **NS** |
| HIMACHAL PRADESH | BILASPUR | **NS** | **NS** | **NS** | **NS** | **NS** | **NS** | **NS** | **NS** | **NS** | **NS** | **NS** | **NS** | **NS** |
| HIMACHAL PRADESH | SOLAN | **NS** | **NS** | **NS** | **NS** | **NS** | **NS** | **NS** | **NS** | **NS** | **NS** | **NS** | **NS** | **NS** |
| HIMACHAL PRADESH | SIRMAUR | **NS** | **L** | **NS** | **NS** | **NS** | **NS** | **NS** | **NS** | **NS** | **NS** | **NS** | **NS** | **NS** |
| HIMACHAL PRADESH | SHIMLA | **H** | **NS** | **NS** | **NS** | **NS** | **NS** | **NS** | **NS** | **NS** | **H** | **NS** | **NS** | **NS** |
| HIMACHAL PRADESH | KINNAUR | **NS** | **NS** | **NS** | **NS** | **NS** | **NS** | **NS** | **NA** | **NS** | **NS** | **NS** | **NS** | **NS** |
| JAMMU & KASHMIR | KUPWARA | **NS** | **NS** | **NS** | **NS** | **NS** | **NS** | **NS** | **NS** | **NS** | **NA** | **NS** | **NS** | **NS** |
| JAMMU & KASHMIR | BADGAM | **NS** | **NS** | **NS** | **NS** | **NS** | **NS** | **NS** | **NS** | **NS** | **NA** | **NS** | **NS** | **NS** |
| JAMMU & KASHMIR | PUNCH | **NS** | **NS** | **NS** | **NS** | **NS** | **NS** | **NS** | **NS** | **NS** | **NS** | **NS** | **NS** | **NS** |
| JAMMU & KASHMIR | RAJOURI | **NS** | **NS** | **NS** | **NS** | **NS** | **NS** | **NS** | **NS** | **NS** | **NS** | **NS** | **NS** | **NS** |
| JAMMU & KASHMIR | KATHUA | **NS** | **NS** | **NS** | **NS** | **NS** | **NS** | **NS** | **NS** | **NS** | **NS** | **NS** | **NS** | **NA** |
| JAMMU & KASHMIR | BARAMULA | **NS** | **L** | **NS** | **NS** | **NS** | **NS** | **NS** | **NS** | **NS** | **NS** | **NS** | **NS** | **NS** |
| JAMMU & KASHMIR | BANDIPORE | **NS** | **NS** | **NS** | **NS** | **NS** | **NS** | **NS** | **NS** | **NS** | **NA** | **NS** | **NS** | **NS** |
| JAMMU & KASHMIR | SRINAGAR | **NS** | **NS** | **NS** | **NS** | **NS** | **NS** | **NS** | **NA** | **NS** | **NA** | **NS** | **NS** | **NS** |
| JAMMU & KASHMIR | GANDERBAL | **NS** | **NS** | **NS** | **NS** | **NS** | **NS** | **NS** | **NS** | **NS** | **NA** | **NS** | **NS** | **NS** |
| JAMMU & KASHMIR | PULWAMA | **NS** | **NS** | **NS** | **NS** | **NS** | **NS** | **NS** | **NS** | **NS** | **NA** | **NS** | **NS** | **NS** |
| JAMMU & KASHMIR | SHUPIYAN | **NS** | **NS** | **NS** | **NS** | **NS** | **NS** | **NS** | **NS** | **NS** | **NA** | **NS** | **NS** | **NA** |
| JAMMU & KASHMIR | ANANTNAG | **NS** | **NS** | **NS** | **NS** | **H** | **NS** | **NS** | **NS** | **NS** | **NA** | **NS** | **NS** | **NA** |
| JAMMU & KASHMIR | KULGAM | **NS** | **NS** | **NS** | **NS** | **NS** | **NS** | **NS** | **NS** | **NS** | **NA** | **NS** | **NS** | **NS** |
| JAMMU & KASHMIR | DODA | **NS** | **NS** | **NS** | **NS** | **NS** | **NS** | **NS** | **NS** | **NS** | **NS** | **NS** | **NS** | **NA** |
| JAMMU & KASHMIR | RAMBAN | **NS** | **NS** | **NS** | **NS** | **NS** | **NS** | **NS** | **NS** | **NS** | **NS** | **NS** | **NS** | **NA** |
| JAMMU & KASHMIR | KISHTWAR | **NS** | **NS** | **NS** | **NS** | **NS** | **NS** | **NS** | **NS** | **NS** | **NS** | **NS** | **NS** | **NS** |
| JAMMU & KASHMIR | UDHAMPUR | **NS** | **NS** | **NS** | **NS** | **NS** | **NS** | **NS** | **NS** | **NS** | **NS** | **NS** | **NS** | **NS** |
| JAMMU & KASHMIR | REASI | **NS** | **NS** | **NS** | **NS** | **NS** | **NS** | **NS** | **NS** | **NS** | **NS** | **NS** | **NS** | **NS** |
| JAMMU & KASHMIR | JAMMU | **NS** | **L** | **NS** | **NS** | **NS** | **NS** | **NS** | **NS** | **NS** | **NS** | **NS** | **H** | **NA** |
| JAMMU & KASHMIR | SAMBA | **NS** | **NS** | **NS** | **NS** | **NS** | **NS** | **NS** | **NS** | **NS** | **NS** | **NS** | **NS** | **NA** |
| JHARKHAND | GARHWA | **NS** | **NS** | **NS** | **NS** | **NS** | **NS** | **NS** | **NS** | **NS** | **NS** | **NS** | **NS** | **NS** |
| JHARKHAND | CHATRA | **NS** | **NS** | **NS** | **NS** | **NS** | **NS** | **NS** | **NS** | **NS** | **NS** | **NS** | **NS** | **NS** |
| JHARKHAND | KODARMA | **NS** | **NS** | **NS** | **NS** | **NS** | **NS** | **NS** | **NS** | **NS** | **NS** | **NS** | **NS** | **NS** |
| JHARKHAND | GIRIDIH | **NS** | **NS** | **NS** | **H** | **NS** | **NS** | **NS** | **NS** | **NS** | **NS** | **NS** | **NS** | **NS** |
| JHARKHAND | DEOGHAR | **NS** | **NS** | **NS** | **NS** | **NS** | **NS** | **NS** | **NS** | **NS** | **NS** | **NS** | **NS** | **NS** |
| JHARKHAND | GODDA | **NS** | **NS** | **NS** | **NS** | **NS** | **NS** | **NS** | **NS** | **NS** | **NS** | **NS** | **NS** | **NS** |
| JHARKHAND | SAHIBGANJ | **NS** | **NS** | **L** | **NS** | **NS** | **NS** | **NS** | **NS** | **NS** | **NS** | **NS** | **NS** | **NS** |
| JHARKHAND | PAKUR | **NS** | **NS** | **NS** | **NS** | **NS** | **NS** | **NS** | **NS** | **NS** | **NS** | **NS** | **NS** | **NS** |
| JHARKHAND | DHANBAD | **NS** | **NS** | **NS** | **NS** | **NS** | **NS** | **NS** | **NS** | **NS** | **NS** | **NS** | **NS** | **NS** |
| JHARKHAND | BOKARO | **NS** | **NS** | **NS** | **NS** | **NS** | **NS** | **NS** | **NS** | **NS** | **NS** | **NS** | **NS** | **NS** |
| JHARKHAND | LOHARDAGA | **NS** | **NS** | **NS** | **NS** | **NS** | **NS** | **NS** | **NS** | **NS** | **NS** | **NS** | **NS** | **NS** |
| JHARKHAND | PURBI SINGHBHUM | **NS** | **NS** | **NS** | **NS** | **NS** | **NS** | **NS** | **NS** | **NS** | **NS** | **NS** | **NS** | **NS** |
| JHARKHAND | PALAMU | **NS** | **L** | **NS** | **NS** | **NS** | **NS** | **NS** | **NS** | **NS** | **NS** | **NS** | **NS** | **NS** |
| JHARKHAND | LATEHAR | **NS** | **NS** | **NS** | **NS** | **NS** | **NS** | **NS** | **NS** | **NS** | **NS** | **NS** | **NS** | **NS** |
| JHARKHAND | HAZARIBAGH | **NS** | **NS** | **NS** | **NS** | **NS** | **NS** | **NS** | **NS** | **NS** | **NS** | **NS** | **NS** | **NS** |
| JHARKHAND | RAMGARH | **NS** | **L** | **NS** | **NS** | **NS** | **NS** | **NS** | **NS** | **NS** | **NS** | **NS** | **NS** | **NS** |
| JHARKHAND | DUMKA | **NS** | **NS** | **NS** | **NS** | **NS** | **NS** | **NS** | **NS** | **NS** | **NS** | **NS** | **NS** | **NS** |
| JHARKHAND | JAMTARA | **NS** | **NS** | **NS** | **NS** | **NS** | **NS** | **NS** | **NS** | **NS** | **NS** | **NS** | **NS** | **NS** |
| JHARKHAND | RANCHI | **H** | **NS** | **NS** | **NS** | **NS** | **NS** | **NS** | **NS** | **H** | **NS** | **NS** | **NS** | **NS** |
| JHARKHAND | KHUNTI | **NS** | **NS** | **NS** | **NS** | **NS** | **NS** | **NS** | **NS** | **NS** | **NS** | **NS** | **NS** | **NA** |
| JHARKHAND | GUMLA | **NS** | **NS** | **NS** | **NS** | **NS** | **NS** | **NS** | **NS** | **NS** | **NS** | **NS** | **NS** | **NS** |
| JHARKHAND | SIMDEGA | **NS** | **NS** | **NS** | **NS** | **NS** | **NS** | **NS** | **NS** | **NS** | **NS** | **NS** | **NS** | **NS** |
| JHARKHAND | PASHCHIMI SINGHBHUM | **NS** | **NS** | **NS** | **NS** | **NS** | **NS** | **NS** | **NS** | **NS** | **NS** | **NS** | **NS** | **NS** |
| JHARKHAND | SARAIKELA-KHARSAWAN | **NS** | **NS** | **NS** | **NS** | **NS** | **NS** | **NS** | **NS** | **NS** | **NS** | **NS** | **NS** | **NS** |
| KARNATAKA | BELGAUM | **H** | **H** | **H** | **NS** | **NS** | **L** | **L** | **NS** | **H** | **NS** | **H** | **NS** | **NS** |
| KARNATAKA | BAGALKOT | **NS** | **NS** | **NS** | **NS** | **NS** | **H** | **NS** | **NS** | **H** | **NS** | **NS** | **NS** | **NS** |
| KARNATAKA | BIJAPUR | **NS** | **NS** | **NS** | **NS** | **NS** | **NS** | **NS** | **NS** | **H** | **NS** | **NS** | **NS** | **NS** |
| KARNATAKA | BIDAR | **NS** | **NS** | **NS** | **NS** | **NS** | **NS** | **NS** | **H** | **NS** | **NS** | **H** | **NS** | **NS** |
| KARNATAKA | RAICHUR | **H** | **L** | **NS** | **NS** | **NS** | **NS** | **L** | **NS** | **NS** | **NS** | **NS** | **NS** | **NS** |
| KARNATAKA | KOPPAL | **NS** | **L** | **NS** | **NS** | **NS** | **NS** | **NS** | **NS** | **L** | **H** | **NS** | **NS** | **NS** |
| KARNATAKA | GADAG | **H** | **L** | **NS** | **NS** | **NS** | **NS** | **NS** | **NS** | **NS** | **NS** | **NS** | **NS** | **NS** |
| KARNATAKA | DHARWAD | **NS** | **NS** | **NS** | **H** | **NS** | **NS** | **NS** | **NS** | **NS** | **NS** | **NS** | **H** | **NS** |
| KARNATAKA | UTTARA KANNADA | **NS** | **L** | **L** | **NS** | **H** | **NS** | **H** | **NS** | **NS** | **H** | **H** | **NS** | **NS** |
| KARNATAKA | HAVERI | **H** | **L** | **NS** | **NS** | **NS** | **H** | **L** | **NS** | **NS** | **NS** | **NS** | **L** | **NS** |
| KARNATAKA | BELLARY | **NS** | **L** | **NS** | **NS** | **NS** | **NS** | **NS** | **NS** | **NS** | **NS** | **NS** | **H** | **NS** |
| KARNATAKA | CHITRADURGA | **NS** | **L** | **NS** | **NS** | **NS** | **NS** | **NS** | **NS** | **NS** | **NS** | **H** | **NS** | **NS** |
| KARNATAKA | DAVANAGERE | **NS** | **L** | **NS** | **NS** | **NS** | **NS** | **NS** | **NS** | **L** | **H** | **H** | **NS** | **NS** |
| KARNATAKA | SHIMOGA | **NS** | **NS** | **NS** | **NS** | **NS** | **NS** | **NS** | **NS** | **NS** | **L** | **NS** | **NS** | **NS** |
| KARNATAKA | UDUPI | **NS** | **NS** | **NS** | **NS** | **NS** | **NS** | **NS** | **NS** | **NS** | **NS** | **NS** | **NS** | **NS** |
| KARNATAKA | CHIKMAGALUR | **NS** | **NS** | **NS** | **NS** | **H** | **H** | **L** | **NS** | **NS** | **H** | **NS** | **NS** | **NS** |
| KARNATAKA | TUMKUR | **H** | **L** | **NS** | **NS** | **NS** | **L** | **NS** | **L** | **L** | **H** | **H** | **NS** | **NS** |
| KARNATAKA | BANGALORE | **L** | **L** | **L** | **H** | **NS** | **NS** | **L** | **H** | **NS** | **L** | **NS** | **NS** | **L** |
| KARNATAKA | MANDYA | **NS** | **NS** | **NS** | **NS** | **NS** | **NS** | **NS** | **NS** | **NS** | **NS** | **NS** | **L** | **NS** |
| KARNATAKA | HASSAN | **NS** | **L** | **NS** | **NS** | **NS** | **NS** | **NS** | **NS** | **NS** | **NS** | **NS** | **NS** | **NS** |
| KARNATAKA | DAKSHINA KANNADA | **H** | **NS** | **NS** | **NS** | **NS** | **NS** | **NS** | **H** | **NS** | **NS** | **NS** | **NS** | **NS** |
| KARNATAKA | KODAGU | **NS** | **NS** | **NS** | **NS** | **NS** | **NS** | **NS** | **NS** | **NS** | **NS** | **NS** | **NS** | **NS** |
| KARNATAKA | MYSORE | **NS** | **NS** | **L** | **H** | **H** | **H** | **L** | **H** | **NS** | **L** | **H** | **NS** | **NS** |
| KARNATAKA | CHAMARAJANAGAR | **NS** | **L** | **L** | **NS** | **NS** | **NS** | **NS** | **NS** | **NS** | **NS** | **NS** | **NS** | **NS** |
| KARNATAKA | GULBARGA | **NS** | **L** | **NS** | **NS** | **NS** | **NS** | **NS** | **NS** | **NS** | **NS** | **L** | **NS** | **NS** |
| KARNATAKA | YADGIR | **NS** | **NS** | **NS** | **NS** | **NS** | **NS** | **NS** | **NS** | **NS** | **NS** | **NS** | **NS** | **NS** |
| KARNATAKA | KOLAR | **NS** | **NS** | **NS** | **NS** | **NS** | **NS** | **NS** | **L** | **NS** | **NS** | **NS** | **NS** | **L** |
| KARNATAKA | CHIKKABALLAPURA | **NS** | **NS** | **NS** | **NS** | **NS** | **NS** | **NS** | **NS** | **NS** | **NS** | **NS** | **NS** | **NS** |
| KARNATAKA | BANGALORE RURAL | **NS** | **NS** | **NS** | **NS** | **NS** | **NS** | **NS** | **NS** | **NS** | **NS** | **NS** | **NS** | **NS** |
| KARNATAKA | RAMANAGARA | **NS** | **L** | **NS** | **NS** | **NS** | **NS** | **NS** | **NS** | **L** | **NS** | **NS** | **NS** | **NS** |
| KERALA | KASARAGOD | **NS** | **NS** | **NS** | **NS** | **NS** | **NS** | **NS** | **NS** | **NS** | **NS** | **NS** | **NS** | **NS** |
| KERALA | KANNUR | **NS** | **L** | **NS** | **NS** | **H** | **H** | **NS** | **L** | **NS** | **NS** | **NS** | **L** | **L** |
| KERALA | WAYANAD | **H** | **L** | **NS** | **NS** | **NS** | **NS** | **NS** | **NS** | **NS** | **NS** | **NS** | **NS** | **NS** |
| KERALA | KOZHIKODE | **H** | **NS** | **NS** | **NS** | **NS** | **NS** | **L** | **NS** | **NS** | **L** | **H** | **NS** | **NS** |
| KERALA | MALAPPURAM | **H** | **L** | **NA** | **H** | **NS** | **NS** | **H** | **L** | **NS** | **NS** | **NS** | **NS** | **H** |
| KERALA | PALAKKAD | **H** | **NS** | **NS** | **NS** | **NS** | **NS** | **NS** | **L** | **NS** | **NS** | **NS** | **NS** | **NS** |
| KERALA | THRISSUR | **NS** | **L** | **NA** | **NS** | **NS** | **NS** | **L** | **L** | **L** | **NS** | **H** | **NS** | **NS** |
| KERALA | ERNAKULAM | **H** | **NS** | **NS** | **H** | **NS** | **NS** | **L** | **NS** | **H** | **NS** | **NS** | **NS** | **L** |
| KERALA | IDUKKI | **H** | **NS** | **NS** | **NS** | **NS** | **NS** | **NS** | **NS** | **NS** | **NS** | **NS** | **NS** | **NS** |
| KERALA | KOTTAYAM | **H** | **L** | **NS** | **NS** | **NS** | **NS** | **NS** | **L** | **L** | **NS** | **NS** | **NS** | **L** |
| KERALA | ALAPPUZHA | **H** | **L** | **NA** | **NS** | **L** | **L** | **L** | **NS** | **NS** | **NS** | **L** | **H** | **NS** |
| KERALA | PATHANAMTHITTA | **NS** | **NS** | **NA** | **NS** | **NS** | **L** | **NS** | **NS** | **NS** | **NS** | **L** | **H** | **NS** |
| KERALA | KOLLAM | **NS** | **L** | **NA** | **NS** | **NS** | **NS** | **H** | **NS** | **NS** | **NS** | **NS** | **NS** | **NS** |
| KERALA | THIRUVANANTHAPURAM | **NS** | **L** | **H** | **H** | **NS** | **NS** | **H** | **H** | **L** | **NS** | **NS** | **NS** | **NS** |
| MADHYA PRADESH | SHEOPUR | **NS** | **NS** | **NS** | **NS** | **NS** | **NS** | **NS** | **NS** | **NS** | **NS** | **NS** | **NS** | **NS** |
| MADHYA PRADESH | MORENA | **H** | **NS** | **H** | **NS** | **NS** | **NS** | **NS** | **NS** | **NS** | **NS** | **NS** | **NS** | **NS** |
| MADHYA PRADESH | BHIND | **NS** | **NS** | **NS** | **NS** | **NS** | **NS** | **NS** | **NS** | **NS** | **NS** | **NS** | **NS** | **NS** |
| MADHYA PRADESH | GWALIOR | **H** | **L** | **L** | **NS** | **H** | **H** | **L** | **L** | **NS** | **NS** | **NS** | **NS** | **NS** |
| MADHYA PRADESH | DATIA | **NS** | **NS** | **NS** | **NS** | **NS** | **NS** | **NS** | **NS** | **NS** | **NS** | **H** | **NS** | **NS** |
| MADHYA PRADESH | SHIVPURI | **NS** | **L** | **NS** | **NS** | **NS** | **NS** | **NS** | **NS** | **NS** | **NS** | **NS** | **NS** | **NA** |
| MADHYA PRADESH | TIKAMGARH | **NS** | **NS** | **H** | **NS** | **NS** | **NS** | **NS** | **NA** | **NS** | **NS** | **NS** | **NS** | **NS** |
| MADHYA PRADESH | CHHATARPUR | **NS** | **NS** | **NS** | **NS** | **NS** | **NS** | **NS** | **NS** | **NS** | **NS** | **NS** | **NS** | **NS** |
| MADHYA PRADESH | PANNA | **NS** | **NS** | **NS** | **NS** | **NS** | **NS** | **NS** | **NS** | **NS** | **NS** | **NS** | **NS** | **NS** |
| MADHYA PRADESH | SAGAR | **H** | **NS** | **NS** | **NS** | **NS** | **NS** | **NS** | **NS** | **NS** | **NS** | **NS** | **NS** | **NS** |
| MADHYA PRADESH | DAMOH | **NS** | **L** | **NS** | **L** | **NS** | **NS** | **NS** | **NS** | **NS** | **NS** | **NS** | **NS** | **NS** |
| MADHYA PRADESH | SATNA | **NS** | **L** | **NS** | **L** | **NS** | **NS** | **NS** | **NS** | **NS** | **NS** | **NS** | **NS** | **NS** |
| MADHYA PRADESH | REWA | **NS** | **NS** | **L** | **NS** | **NS** | **NS** | **NS** | **NS** | **NS** | **NS** | **NS** | **NS** | **NS** |
| MADHYA PRADESH | UMARIA | **NS** | **NS** | **NS** | **NS** | **NS** | **NS** | **NS** | **NS** | **NS** | **NS** | **NS** | **NS** | **NS** |
| MADHYA PRADESH | NEEMUCH | **NS** | **NS** | **NS** | **NS** | **NS** | **NS** | **NS** | **NS** | **NS** | **NS** | **NS** | **NS** | **NS** |
| MADHYA PRADESH | MANDSAUR | **NS** | **NS** | **NS** | **NS** | **NS** | **NS** | **NS** | **NS** | **NS** | **NS** | **NS** | **NS** | **NS** |
| MADHYA PRADESH | RATLAM | **NS** | **NS** | **NS** | **NS** | **NS** | **NS** | **NS** | **NS** | **NS** | **NS** | **NS** | **NS** | **NS** |
| MADHYA PRADESH | UJJAIN | **NS** | **L** | **NS** | **NS** | **NS** | **NS** | **NS** | **NS** | **NS** | **NS** | **NS** | **NS** | **NS** |
| MADHYA PRADESH | DEWAS | **NS** | **NS** | **NS** | **NS** | **NS** | **NS** | **NS** | **NS** | **NS** | **NS** | **NS** | **NS** | **NS** |
| MADHYA PRADESH | DHAR | **NS** | **NS** | **NS** | **H** | **NS** | **NS** | **NS** | **NA** | **NS** | **NS** | **NS** | **NS** | **NS** |
| MADHYA PRADESH | INDORE | **NS** | **L** | **NS** | **NS** | **NS** | **NS** | **NS** | **NS** | **NS** | **NS** | **NS** | **NS** | **NS** |
| MADHYA PRADESH | KHARGONE (WEST NIMAR) | **NS** | **NS** | **NS** | **NS** | **L** | **NS** | **NS** | **NS** | **H** | **NS** | **NS** | **NS** | **NS** |
| MADHYA PRADESH | BARWANI | **NS** | **NS** | **NS** | **NS** | **NS** | **NS** | **NS** | **NS** | **NS** | **NS** | **NS** | **NS** | **NA** |
| MADHYA PRADESH | RAJGARH | **NS** | **NS** | **NS** | **NS** | **NS** | **NS** | **NS** | **NS** | **NS** | **NS** | **NS** | **NS** | **NA** |
| MADHYA PRADESH | VIDISHA | **NS** | **NS** | **NS** | **NS** | **NS** | **NS** | **NS** | **NS** | **NS** | **NS** | **NS** | **NS** | **NS** |
| MADHYA PRADESH | BHOPAL | **H** | **NS** | **H** | **L** | **NS** | **NS** | **NS** | **L** | **NS** | **NS** | **NS** | **H** | **NS** |
| MADHYA PRADESH | SEHORE | **NS** | **L** | **NS** | **NS** | **NS** | **NS** | **NS** | **NS** | **L** | **NS** | **NS** | **NS** | **NA** |
| MADHYA PRADESH | RAISEN | **NS** | **NS** | **NS** | **NS** | **NS** | **NS** | **NS** | **NA** | **NS** | **NS** | **NS** | **NS** | **NA** |
| MADHYA PRADESH | BETUL | **NS** | **NS** | **NS** | **NS** | **NS** | **NS** | **NS** | **NS** | **NS** | **NS** | **NS** | **NS** | **NS** |
| MADHYA PRADESH | HARDA | **NS** | **NS** | **NS** | **NS** | **NS** | **NS** | **NS** | **NS** | **NS** | **NS** | **NS** | **NS** | **NS** |
| MADHYA PRADESH | HOSHANGABAD | **NS** | **NS** | **NS** | **NS** | **NS** | **NS** | **NS** | **NS** | **NS** | **NS** | **NS** | **NS** | **NS** |
| MADHYA PRADESH | KATNI | **NS** | **NS** | **NS** | **NS** | **NS** | **NS** | **NS** | **NS** | **NS** | **NS** | **NS** | **NS** | **NA** |
| MADHYA PRADESH | JABALPUR | **L** | **L** | **NS** | **L** | **NS** | **NS** | **NS** | **NA** | **H** | **L** | **H** | **NS** | **NA** |
| MADHYA PRADESH | NARSIMHAPUR | **NS** | **NS** | **NS** | **NS** | **NS** | **NS** | **NS** | **NS** | **NS** | **NS** | **NS** | **NS** | **NS** |
| MADHYA PRADESH | DINDORI | **NS** | **NS** | **NS** | **NS** | **NS** | **NS** | **NS** | **NS** | **NS** | **NS** | **NS** | **NS** | **NS** |
| MADHYA PRADESH | MANDLA | **NS** | **NS** | **NS** | **NS** | **NS** | **NS** | **NS** | **NS** | **NS** | **NS** | **NS** | **NS** | **NS** |
| MADHYA PRADESH | CHHINDWARA | **NS** | **NS** | **NS** | **NS** | **NS** | **NS** | **NS** | **NS** | **NS** | **NS** | **NS** | **NS** | **NS** |
| MADHYA PRADESH | SEONI | **H** | **NS** | **NS** | **NS** | **NS** | **NS** | **NS** | **NS** | **NS** | **NS** | **NS** | **NS** | **NS** |
| MADHYA PRADESH | BALAGHAT | **NS** | **NS** | **NS** | **H** | **H** | **NS** | **NS** | **NS** | **NS** | **NS** | **NS** | **NS** | **NA** |
| MADHYA PRADESH | GUNA | **NS** | **NS** | **L** | **NS** | **NS** | **NS** | **NS** | **NS** | **NS** | **NS** | **NS** | **NS** | **NS** |
| MADHYA PRADESH | ASHOKNAGAR | **NS** | **L** | **NS** | **L** | **NS** | **NS** | **NS** | **NS** | **NS** | **NS** | **NS** | **NS** | **NS** |
| MADHYA PRADESH | SHAHDOL | **NS** | **NS** | **NS** | **NS** | **NS** | **NS** | **NS** | **NS** | **NS** | **NS** | **NS** | **NS** | **NS** |
| MADHYA PRADESH | ANUPPUR | **NS** | **NS** | **NS** | **NS** | **NS** | **NS** | **NS** | **NS** | **NS** | **NS** | **NS** | **NS** | **NS** |
| MADHYA PRADESH | SIDHI | **NS** | **NS** | **NS** | **NS** | **NS** | **NS** | **NS** | **NS** | **NS** | **NS** | **NS** | **NS** | **NS** |
| MADHYA PRADESH | SINGRAULI | **NS** | **NS** | **L** | **NS** | **NS** | **NS** | **NS** | **L** | **NS** | **NS** | **NS** | **NS** | **NS** |
| MADHYA PRADESH | JHABUA | **NS** | **NS** | **NS** | **NS** | **NS** | **NS** | **NS** | **NS** | **NS** | **NS** | **NS** | **NS** | **NS** |
| MADHYA PRADESH | ALIRAJPUR | **NS** | **NS** | **NS** | **NS** | **NS** | **NS** | **NS** | **NS** | **NS** | **NS** | **NS** | **NS** | **NS** |
| MADHYA PRADESH | KHANDWA (EAST NIMAR) | **NS** | **NS** | **NS** | **NS** | **NS** | **NS** | **NS** | **NS** | **NS** | **NS** | **NS** | **NS** | **NS** |
| MADHYA PRADESH | BURHANPUR | **NS** | **NS** | **NS** | **NS** | **NS** | **NS** | **NS** | **NS** | **NS** | **NS** | **NS** | **NS** | **NS** |
| MADHYA PRADESH | AGAR MALWA | **NS** | **NS** | **NS** | **NS** | **NS** | **NS** | **NS** | **NA** | **NS** | **NS** | **NS** | **NS** | **NS** |
| MADHYA PRADESH | SHAJAPUR | **NS** | **NS** | **NS** | **NS** | **NS** | **NS** | **NS** | **NS** | **NS** | **NS** | **NS** | **NS** | **NA** |
| MAHARASHTRA | NANDURBAR | **NS** | **L** | **NS** | **NS** | **NS** | **NS** | **NS** | **NS** | **NS** | **NS** | **NS** | **NS** | **NA** |
| MAHARASHTRA | DHULE | **NS** | **L** | **L** | **NS** | **L** | **L** | **NS** | **NS** | **NS** | **NS** | **H** | **NS** | **NS** |
| MAHARASHTRA | JALGAON | **NS** | **L** | **NS** | **L** | **NS** | **L** | **NS** | **NS** | **NS** | **NS** | **H** | **NS** | **NS** |
| MAHARASHTRA | BULDANA | **NS** | **L** | **NS** | **NS** | **NS** | **NS** | **NS** | **NS** | **NS** | **NS** | **NS** | **H** | **NS** |
| MAHARASHTRA | AKOLA | **NS** | **L** | **NS** | **NS** | **NS** | **NS** | **NS** | **NS** | **NS** | **NS** | **NS** | **NS** | **NS** |
| MAHARASHTRA | WASHIM | **NS** | **L** | **NS** | **NS** | **NS** | **NS** | **NS** | **NS** | **NS** | **NS** | **NS** | **NS** | **NS** |
| MAHARASHTRA | AMRAVATI | **NS** | **L** | **NS** | **NS** | **H** | **NS** | **NS** | **NS** | **NS** | **NS** | **H** | **H** | **NS** |
| MAHARASHTRA | WARDHA | **NS** | **NS** | **L** | **NS** | **NS** | **NS** | **NS** | **NS** | **NS** | **NS** | **H** | **NS** | **NS** |
| MAHARASHTRA | NAGPUR | **NS** | **NS** | **NS** | **NS** | **NS** | **NS** | **NS** | **L** | **H** | **NS** | **NS** | **NS** | **NS** |
| MAHARASHTRA | BHANDARA | **NS** | **NS** | **NS** | **H** | **NS** | **NS** | **NS** | **NS** | **L** | **NS** | **NS** | **NS** | **NS** |
| MAHARASHTRA | GONDIYA | **NS** | **L** | **NS** | **NS** | **NS** | **NS** | **NS** | **NS** | **NS** | **NS** | **NS** | **NS** | **NS** |
| MAHARASHTRA | GADCHIROLI | **NS** | **NS** | **NS** | **NS** | **NS** | **NS** | **NS** | **NS** | **NS** | **NS** | **NS** | **NS** | **NS** |
| MAHARASHTRA | CHANDRAPUR | **NS** | **NS** | **NS** | **NS** | **NS** | **NS** | **NS** | **H** | **NS** | **NS** | **NS** | **NS** | **NS** |
| MAHARASHTRA | YAVATMAL | **H** | **L** | **L** | **NS** | **L** | **L** | **H** | **NS** | **NS** | **L** | **H** | **NS** | **NS** |
| MAHARASHTRA | NANDED | **H** | **L** | **NS** | **NS** | **NS** | **NS** | **L** | **NS** | **NS** | **H** | **NS** | **NS** | **NS** |
| MAHARASHTRA | HINGOLI | **NS** | **NS** | **NS** | **NS** | **NS** | **NS** | **NS** | **NS** | **NS** | **NS** | **NS** | **NS** | **NS** |
| MAHARASHTRA | PARBHANI | **NS** | **NS** | **NS** | **NS** | **NS** | **L** | **NS** | **H** | **NS** | **NS** | **NS** | **NS** | **NS** |
| MAHARASHTRA | JALNA | **NS** | **NS** | **NS** | **NS** | **NS** | **NS** | **NS** | **NS** | **H** | **NS** | **NS** | **NS** | **NS** |
| MAHARASHTRA | AURANGABAD | **NS** | **L** | **NS** | **NS** | **NS** | **NS** | **H** | **NS** | **NS** | **NS** | **NS** | **NS** | **NS** |
| MAHARASHTRA | NASHIK | **NS** | **L** | **NS** | **L** | **NS** | **L** | **NS** | **H** | **L** | **NS** | **NS** | **NS** | **NS** |
| MAHARASHTRA | MUMBAI SUBURBAN | **L** | **H** | **L** | **H** | **NA** | **NA** | **H** | **NA** | **NS** | **NS** | **H** | **L** | **L** |
| MAHARASHTRA | MUMBAI | **NS** | **NS** | **NS** | **NS** | **L** | **L** | **H** | **NA** | **NS** | **H** | **H** | **NS** | **L** |
| MAHARASHTRA | RAIGARH | **H** | **L** | **NS** | **NS** | **NS** | **NS** | **NS** | **NS** | **L** | **NS** | **NS** | **NS** | **NS** |
| MAHARASHTRA | PUNE | **L** | **L** | **L** | **L** | **H** | **H** | **H** | **L** | **H** | **L** | **H** | **L** | **L** |
| MAHARASHTRA | AHMADNAGAR | **NS** | **NS** | **NS** | **H** | **NS** | **NS** | **NS** | **NS** | **NS** | **NS** | **NS** | **H** | **NS** |
| MAHARASHTRA | BID | **H** | **NS** | **NS** | **NS** | **L** | **H** | **L** | **L** | **NS** | **NS** | **NS** | **NS** | **NS** |
| MAHARASHTRA | LATUR | **H** | **NS** | **NS** | **NS** | **NS** | **NS** | **NS** | **NS** | **L** | **NS** | **NS** | **NS** | **NS** |
| MAHARASHTRA | OSMANABAD | **NS** | **L** | **NS** | **NS** | **NS** | **NS** | **NS** | **NS** | **NS** | **NS** | **H** | **L** | **NS** |
| MAHARASHTRA | SOLAPUR | **NS** | **NS** | **NS** | **NS** | **NS** | **L** | **L** | **H** | **NS** | **NS** | **NS** | **NS** | **NS** |
| MAHARASHTRA | SATARA | **NS** | **NS** | **NS** | **NS** | **L** | **NS** | **NS** | **NS** | **NS** | **NS** | **NS** | **NS** | **NS** |
| MAHARASHTRA | RATNAGIRI | **NS** | **L** | **NS** | **NS** | **NS** | **NS** | **NS** | **NS** | **NS** | **NS** | **NS** | **NS** | **NS** |
| MAHARASHTRA | SINDHUDURG | **NS** | **NS** | **NS** | **NS** | **NS** | **NS** | **NS** | **NS** | **NS** | **NS** | **NS** | **NS** | **NS** |
| MAHARASHTRA | KOLHAPUR | **NS** | **NS** | **NS** | **NS** | **NS** | **NS** | **NS** | **NS** | **NS** | **L** | **NS** | **NS** | **H** |
| MAHARASHTRA | SANGLI | **NS** | **L** | **NS** | **NS** | **NS** | **NS** | **NS** | **NS** | **NS** | **NS** | **H** | **H** | **NS** |
| MAHARASHTRA | PALGHAR | **NS** | **NS** | **NS** | **NS** | **NS** | **NS** | **H** | **NS** | **NS** | **NS** | **NS** | **NS** | **NS** |
| MAHARASHTRA | THANE | **L** | **NS** | **H** | **H** | **H** | **NS** | **L** | **NS** | **H** | **NS** | **H** | **H** | **NA** |
| MANIPUR | SENAPATI | **NS** | **NS** | **NS** | **NS** | **NS** | **NS** | **NS** | **NS** | **NS** | **NS** | **NS** | **NS** | **NS** |
| MANIPUR | TAMENGLONG | **NS** | **NS** | **NS** | **NS** | **NS** | **NS** | **NS** | **NS** | **NS** | **NS** | **NS** | **NS** | **NS** |
| MANIPUR | CHURACHANDPUR | **NS** | **NS** | **NS** | **NS** | **NS** | **NS** | **NS** | **NS** | **NS** | **NS** | **NS** | **NS** | **NS** |
| MANIPUR | BISHNUPUR | **NS** | **NS** | **NS** | **NS** | **NS** | **NS** | **NS** | **NS** | **NS** | **NS** | **NS** | **NS** | **NS** |
| MANIPUR | THOUBAL | **NS** | **NS** | **NS** | **NS** | **NS** | **NS** | **NS** | **NS** | **NS** | **NS** | **NS** | **NS** | **NS** |
| MANIPUR | IMPHAL WEST | **NS** | **NS** | **NS** | **NS** | **NS** | **NS** | **NS** | **NS** | **NS** | **NS** | **NS** | **NS** | **NS** |
| MANIPUR | IMPHAL EAST | **NS** | **L** | **NS** | **NS** | **NS** | **NS** | **NS** | **NS** | **NS** | **NS** | **NS** | **NS** | **NS** |
| MANIPUR | UKHRUL | **NS** | **NS** | **NS** | **NS** | **NS** | **NS** | **NS** | **NS** | **NS** | **NS** | **NS** | **NS** | **NA** |
| MANIPUR | CHANDEL | **NS** | **NS** | **NS** | **NS** | **NS** | **NS** | **NS** | **NS** | **NS** | **NS** | **NS** | **NS** | **NS** |
| MEGHALAYA | SOUTH GARO HILLS | **NS** | **NS** | **NS** | **NS** | **NS** | **NS** | **NS** | **NS** | **NS** | **NS** | **NS** | **NS** | **NA** |
| MEGHALAYA | RIBHOI | **NS** | **NS** | **NS** | **NS** | **NS** | **NS** | **NS** | **NS** | **NS** | **NS** | **NS** | **NS** | **NS** |
| MEGHALAYA | EAST KHASI HILLS | **NS** | **NS** | **NS** | **NS** | **NS** | **NS** | **NS** | **H** | **NS** | **NS** | **NS** | **NS** | **NS** |
| MEGHALAYA | EAST GARO HILLS | **NS** | **NS** | **NS** | **NS** | **NS** | **NS** | **NS** | **NS** | **NS** | **NS** | **NS** | **NS** | **NS** |
| MEGHALAYA | EAST JANTIA HILLS | **NS** | **NS** | **NS** | **NS** | **NS** | **NS** | **NS** | **NA** | **NS** | **NS** | **NS** | **NS** | **NA** |
| MEGHALAYA | NORTH GARO HILLS | **NS** | **NS** | **NS** | **NS** | **NS** | **NS** | **NS** | **NS** | **NS** | **NS** | **NS** | **NS** | **NS** |
| MEGHALAYA | SOUTH WEST GARO HILLS | **NS** | **NS** | **NS** | **NS** | **NS** | **NS** | **NS** | **NA** | **NS** | **NS** | **NS** | **NS** | **NS** |
| MEGHALAYA | SOUTH WEST KHASI HILLS | **NS** | **NS** | **NS** | **NS** | **NS** | **NA** | **NS** | **NA** | **NS** | **NS** | **NS** | **NS** | **NS** |
| MEGHALAYA | WEST GARO HILLS | **NS** | **NS** | **NS** | **NS** | **NS** | **NS** | **NS** | **NS** | **NS** | **NS** | **NS** | **NS** | **NS** |
| MEGHALAYA | WEST JAINTIA HILLS | **NS** | **NS** | **NS** | **NS** | **NS** | **NS** | **NS** | **NS** | **NS** | **NS** | **NS** | **NS** | **NS** |
| MEGHALAYA | WEST KHASI HILLS | **NS** | **NS** | **NS** | **NS** | **NS** | **NS** | **NS** | **NS** | **NS** | **NS** | **NS** | **NS** | **NS** |
| NCT OF DELHI | CENTRAL | **NS** | **NS** | **NS** | **NS** | **NS** | **NS** | **NS** | **NA** | **NS** | **NS** | **NS** | **NS** | **NS** |
| NCT OF DELHI | EAST | **NS** | **NS** | **NS** | **NS** | **NS** | **NS** | **NS** | **NA** | **NS** | **NS** | **H** | **NS** | **NS** |
| NCT OF DELHI | NEW DELHI | **NS** | **NS** | **NS** | **NS** | **NS** | **NS** | **NS** | **NA** | **NS** | **NS** | **NS** | **NS** | **NS** |
| NCT OF DELHI | NORTH | **NS** | **NS** | **L** | **NS** | **NS** | **NS** | **NS** | **NS** | **NS** | **NS** | **NS** | **NS** | **NS** |
| NCT OF DELHI | NORTH EAST | **NS** | **NS** | **NS** | **NS** | **NS** | **NS** | **NS** | **NA** | **NS** | **NS** | **NS** | **NS** | **NS** |
| NCT OF DELHI | NORTH WEST | **NS** | **L** | **NS** | **NS** | **NS** | **NS** | **NS** | **NA** | **NS** | **NS** | **NS** | **NS** | **NS** |
| NCT OF DELHI | SHAHDARA | **NS** | **NS** | **NS** | **NS** | **NS** | **NS** | **NS** | **NA** | **NS** | **NS** | **NS** | **NS** | **NS** |
| NCT OF DELHI | SOUTH | **NS** | **NS** | **NS** | **NS** | **NS** | **NA** | **NS** | **NA** | **NS** | **NS** | **NS** | **NS** | **NS** |
| NCT OF DELHI | SOUTH EAST | **NS** | **NS** | **NS** | **NS** | **L** | **L** | **NS** | **NA** | **NS** | **NS** | **NS** | **NS** | **NS** |
| NCT OF DELHI | SOUTH WEST | **NS** | **L** | **NS** | **NS** | **NS** | **NS** | **NS** | **NS** | **NS** | **NS** | **NS** | **NS** | **NS** |
| NCT OF DELHI | WEST | **NS** | **NS** | **NS** | **NS** | **NS** | **NS** | **H** | **NA** | **NS** | **NS** | **NS** | **NS** | **NS** |
| ODISHA | BARGARH | **H** | **L** | **NS** | **NS** | **NS** | **L** | **NS** | **NS** | **NS** | **NS** | **NS** | **NS** | **NS** |
| ODISHA | JHARSUGUDA | **NS** | **NS** | **NS** | **NS** | **NS** | **NS** | **NS** | **NS** | **NS** | **NS** | **NS** | **NS** | **NS** |
| ODISHA | SAMBALPUR | **NS** | **NS** | **NS** | **NS** | **NS** | **NS** | **NS** | **NS** | **NS** | **NS** | **NS** | **NS** | **NS** |
| ODISHA | DEBAGARH | **NS** | **NS** | **NS** | **NS** | **NS** | **NS** | **NS** | **NS** | **NS** | **NS** | **NS** | **NS** | **NS** |
| ODISHA | SUNDARGARH | **H** | **NS** | **NS** | **NS** | **NS** | **NS** | **NS** | **L** | **NS** | **NS** | **NS** | **NS** | **NS** |
| ODISHA | KENDUJHAR | **NS** | **L** | **NS** | **NS** | **NS** | **NS** | **NS** | **NS** | **NS** | **L** | **NS** | **NS** | **NS** |
| ODISHA | MAYURBHANJ | **NS** | **NS** | **L** | **NS** | **NS** | **NS** | **H** | **H** | **NS** | **NS** | **NS** | **NS** | **NS** |
| ODISHA | BALESHWAR | **NS** | **NS** | **NS** | **NS** | **NS** | **NS** | **NS** | **NS** | **NS** | **NS** | **NS** | **H** | **NS** |
| ODISHA | BHADRAK | **NS** | **NS** | **NS** | **NS** | **NS** | **NS** | **NS** | **NS** | **NS** | **NS** | **NS** | **NS** | **NS** |
| ODISHA | KENDRAPARA | **NS** | **NS** | **NS** | **NS** | **NS** | **NS** | **NS** | **NS** | **NS** | **NS** | **NS** | **NS** | **NS** |
| ODISHA | JAGATSINGHAPUR | **NS** | **NS** | **NS** | **NS** | **NS** | **NS** | **NS** | **NS** | **NS** | **NS** | **NS** | **NS** | **NS** |
| ODISHA | CUTTACK | **NS** | **L** | **NS** | **NS** | **NS** | **NS** | **NS** | **L** | **NS** | **NS** | **NS** | **H** | **NS** |
| ODISHA | JAJAPUR | **H** | **NS** | **NS** | **NS** | **NS** | **NS** | **NS** | **NS** | **NS** | **NS** | **NS** | **NS** | **NS** |
| ODISHA | DHENKANAL | **NS** | **NS** | **NS** | **NS** | **NS** | **NS** | **NS** | **NS** | **NS** | **NS** | **NS** | **H** | **NS** |
| ODISHA | ANUGUL | **NS** | **NS** | **NS** | **NS** | **NS** | **NS** | **NS** | **NS** | **NS** | **NS** | **NS** | **NS** | **NS** |
| ODISHA | NAYAGARH | **NS** | **NS** | **NS** | **NS** | **NS** | **NS** | **NS** | **NS** | **NS** | **NS** | **NS** | **NS** | **NS** |
| ODISHA | KHORDHA | **NS** | **NS** | **NS** | **NS** | **NS** | **NS** | **NS** | **NS** | **H** | **NS** | **H** | **NS** | **NS** |
| ODISHA | PURI | **NS** | **NS** | **NS** | **NS** | **NS** | **NS** | **NS** | **NS** | **NS** | **NS** | **NS** | **H** | **NS** |
| ODISHA | GANJAM | **NS** | **NS** | **NS** | **NS** | **NS** | **NS** | **L** | **NS** | **NS** | **NS** | **NS** | **NS** | **NS** |
| ODISHA | GAJAPATI | **NS** | **NS** | **NS** | **NS** | **NS** | **NS** | **NS** | **NS** | **NS** | **NS** | **NS** | **NS** | **NS** |
| ODISHA | KANDHAMAL | **NS** | **NS** | **NS** | **NS** | **NS** | **NS** | **NS** | **NS** | **NS** | **NS** | **NS** | **NS** | **NS** |
| ODISHA | BAUDH | **NS** | **NS** | **NS** | **NS** | **NS** | **NS** | **NS** | **NS** | **NS** | **NS** | **NS** | **NS** | **NS** |
| ODISHA | SUBARNAPUR | **NS** | **NS** | **NS** | **NS** | **NS** | **NS** | **NS** | **NS** | **NS** | **NS** | **NS** | **NS** | **NS** |
| ODISHA | BALANGIR | **NS** | **NS** | **NS** | **NS** | **NS** | **NS** | **NS** | **NS** | **NS** | **NS** | **NS** | **NS** | **NS** |
| ODISHA | NUAPADA | **NS** | **NS** | **NS** | **NS** | **NS** | **NS** | **NS** | **NS** | **NS** | **NS** | **NS** | **NS** | **NS** |
| ODISHA | KALAHANDI | **NS** | **NS** | **NS** | **NS** | **NS** | **NS** | **NS** | **NS** | **NS** | **NS** | **H** | **NS** | **NS** |
| ODISHA | RAYAGADA | **H** | **NS** | **NS** | **NS** | **NS** | **NS** | **NS** | **NS** | **NS** | **NS** | **NS** | **NS** | **NS** |
| ODISHA | NABARANGAPUR | **NS** | **NS** | **NS** | **NS** | **NS** | **NS** | **NS** | **NS** | **NS** | **NS** | **NS** | **NS** | **NA** |
| ODISHA | KORAPUT | **NS** | **NS** | **NS** | **NS** | **NS** | **NS** | **NS** | **NS** | **NS** | **NS** | **NS** | **H** | **NS** |
| ODISHA | MALKANGIRI | **NS** | **NS** | **NS** | **NS** | **NS** | **NS** | **NS** | **NS** | **NS** | **NS** | **NS** | **NS** | **NS** |
| PUNJAB | KAPURTHALA | **NS** | **NS** | **NS** | **NS** | **NS** | **NS** | **NS** | **NS** | **NS** | **NS** | **NS** | **NS** | **NS** |
| PUNJAB | JALANDHAR | **NS** | **NS** | **NS** | **H** | **NS** | **NS** | **NS** | **NS** | **NS** | **NS** | **NS** | **NS** | **NS** |
| PUNJAB | HOSHIARPUR | **NS** | **NS** | **NS** | **NS** | **NS** | **NS** | **NS** | **NS** | **NS** | **NS** | **NS** | **NS** | **NS** |
| PUNJAB | SHAHID BHAGAT SINGH NAGAR | **NS** | **NS** | **NS** | **NS** | **NS** | **NS** | **NS** | **NS** | **NS** | **NS** | **NS** | **NS** | **NS** |
| PUNJAB | FATEHGARH SAHIB | **NS** | **NS** | **NS** | **NS** | **NS** | **NS** | **NS** | **NS** | **NS** | **NS** | **NS** | **NS** | **NS** |
| PUNJAB | LUDHIANA | **NS** | **L** | **NS** | **NS** | **NS** | **NS** | **NS** | **NS** | **NS** | **NS** | **NS** | **NS** | **NS** |
| PUNJAB | MOGA | **NS** | **NS** | **NS** | **NS** | **NS** | **NS** | **NS** | **NS** | **NS** | **NS** | **NS** | **NS** | **NS** |
| PUNJAB | MUKTSAR | **NS** | **NS** | **NS** | **NS** | **NS** | **NS** | **NS** | **NS** | **NS** | **NS** | **NS** | **NS** | **NS** |
| PUNJAB | FARIDKOT | **NS** | **NS** | **NS** | **NS** | **NS** | **NS** | **NS** | **NS** | **NS** | **NS** | **NS** | **NS** | **NS** |
| PUNJAB | BATHINDA | **NS** | **NS** | **NS** | **NS** | **NS** | **NS** | **NS** | **L** | **NS** | **NS** | **NS** | **NS** | **NS** |
| PUNJAB | MANSA | **NS** | **NS** | **NS** | **NS** | **NS** | **NS** | **NS** | **NS** | **NS** | **NS** | **NS** | **NS** | **NS** |
| PUNJAB | PATIALA | **NS** | **NS** | **L** | **H** | **NS** | **NS** | **NS** | **NS** | **NS** | **NS** | **H** | **NS** | **NS** |
| PUNJAB | AMRITSAR | **NS** | **NS** | **NS** | **NS** | **NS** | **L** | **NS** | **NS** | **NS** | **NS** | **NS** | **NS** | **NS** |
| PUNJAB | TARN TARAN | **NS** | **NS** | **NS** | **NS** | **NS** | **NS** | **NS** | **NS** | **NS** | **NS** | **NS** | **NS** | **NS** |
| PUNJAB | RUPNAGAR | **NS** | **NS** | **NS** | **NS** | **NS** | **NS** | **NS** | **NS** | **NS** | **NS** | **NS** | **NS** | **NS** |
| PUNJAB | SAHIBZADA AJIT SINGH NAGAR | **NS** | **NS** | **NS** | **NS** | **NS** | **NS** | **NS** | **NS** | **NS** | **NS** | **NS** | **NS** | **NS** |
| PUNJAB | SANGRUR | **NS** | **NS** | **NS** | **NS** | **NS** | **NS** | **NS** | **L** | **NS** | **NS** | **NS** | **NS** | **NS** |
| PUNJAB | BARNALA | **NS** | **NS** | **NS** | **NS** | **NS** | **NS** | **NS** | **NS** | **NS** | **NS** | **NS** | **NS** | **NS** |
| PUNJAB | FAZILKA | **NS** | **NS** | **NS** | **NS** | **NS** | **NS** | **NS** | **NS** | **NS** | **NS** | **NS** | **NS** | **NS** |
| PUNJAB | FIROZPUR | **NS** | **NS** | **NS** | **NS** | **NS** | **NS** | **NS** | **NS** | **NS** | **NS** | **NS** | **NS** | **NS** |
| PUNJAB | GURDASPUR | **NS** | **NS** | **NS** | **NS** | **NS** | **NS** | **NS** | **NS** | **NS** | **NS** | **NS** | **NS** | **NS** |
| PUNJAB | PATHANKOT | **NS** | **NS** | **NS** | **NS** | **NS** | **NS** | **NS** | **NS** | **NS** | **NS** | **NS** | **NS** | **NS** |
| RAJASTHAN | GANGANAGAR | **NS** | **L** | **NS** | **NS** | **NS** | **NS** | **NS** | **NS** | **NS** | **NS** | **L** | **H** | **NS** |
| RAJASTHAN | HANUMANGARH | **NS** | **NS** | **NS** | **NS** | **NS** | **NS** | **NS** | **NS** | **NS** | **NS** | **NS** | **NS** | **NS** |
| RAJASTHAN | BIKANER | **NS** | **L** | **NS** | **NS** | **NS** | **NS** | **NS** | **NS** | **NS** | **NS** | **NS** | **H** | **NA** |
| RAJASTHAN | CHURU | **NS** | **NS** | **NS** | **NS** | **NS** | **NS** | **NS** | **NS** | **NS** | **NS** | **NS** | **NS** | **NS** |
| RAJASTHAN | JHUNJHUNUN | **NS** | **L** | **NS** | **NS** | **NS** | **NS** | **NS** | **NS** | **NS** | **NS** | **NS** | **H** | **NS** |
| RAJASTHAN | ALWAR | **NS** | **L** | **NS** | **NS** | **NS** | **NS** | **NS** | **NS** | **NS** | **NS** | **NS** | **NS** | **NS** |
| RAJASTHAN | BHARATPUR | **H** | **L** | **NS** | **NS** | **NS** | **NS** | **NS** | **NS** | **NS** | **NS** | **NS** | **NS** | **NS** |
| RAJASTHAN | DHAULPUR | **NS** | **NS** | **NS** | **NS** | **NS** | **NS** | **NS** | **NS** | **NS** | **NS** | **NS** | **NS** | **NS** |
| RAJASTHAN | KARAULI | **NS** | **NS** | **NS** | **NS** | **NS** | **NS** | **NS** | **NS** | **NS** | **NS** | **NS** | **NS** | **NS** |
| RAJASTHAN | SAWAI MADHOPUR | **NS** | **NS** | **NS** | **NS** | **NS** | **NS** | **NS** | **NS** | **NS** | **NS** | **NS** | **NS** | **NA** |
| RAJASTHAN | DAUSA | **NS** | **L** | **NS** | **NS** | **NS** | **NS** | **NS** | **H** | **NS** | **NS** | **NS** | **NS** | **NS** |
| RAJASTHAN | JAIPUR | **NS** | **L** | **NS** | **NS** | **NS** | **NS** | **NS** | **NS** | **NS** | **NS** | **H** | **NS** | **NS** |
| RAJASTHAN | SIKAR | **NS** | **NS** | **L** | **H** | **NS** | **H** | **NS** | **NS** | **NS** | **NS** | **NS** | **NS** | **NS** |
| RAJASTHAN | NAGAUR | **NS** | **NS** | **NS** | **NS** | **NS** | **NS** | **H** | **NS** | **NS** | **NS** | **NS** | **NS** | **NS** |
| RAJASTHAN | JODHPUR | **NS** | **L** | **NS** | **NS** | **H** | **NS** | **H** | **NS** | **NS** | **NS** | **NS** | **NS** | **NS** |
| RAJASTHAN | JAISALMER | **NS** | **NS** | **NS** | **NS** | **NS** | **NS** | **NS** | **NS** | **NS** | **NS** | **NS** | **NS** | **NS** |
| RAJASTHAN | BARMER | **H** | **NS** | **NS** | **NS** | **NS** | **NS** | **NS** | **NS** | **NS** | **NS** | **NS** | **NS** | **NS** |
| RAJASTHAN | JALOR | **H** | **NS** | **NS** | **NS** | **L** | **NS** | **NS** | **NS** | **NS** | **NS** | **NS** | **NS** | **NA** |
| RAJASTHAN | SIROHI | **NS** | **NS** | **NS** | **NS** | **NS** | **NS** | **NS** | **NS** | **NS** | **NS** | **NS** | **NS** | **NS** |
| RAJASTHAN | PALI | **NS** | **NS** | **NS** | **NS** | **NS** | **NS** | **NS** | **NS** | **NS** | **NS** | **NS** | **NS** | **NA** |
| RAJASTHAN | AJMER | **NS** | **NS** | **NS** | **NS** | **NS** | **NS** | **NS** | **NS** | **H** | **NS** | **NS** | **NS** | **NS** |
| RAJASTHAN | TONK | **NS** | **NS** | **NS** | **NS** | **NS** | **NS** | **NS** | **NS** | **NS** | **NS** | **NS** | **NS** | **NS** |
| RAJASTHAN | BUNDI | **NS** | **L** | **NS** | **NS** | **NS** | **NS** | **NS** | **NS** | **NS** | **NS** | **NS** | **NS** | **NS** |
| RAJASTHAN | BHILWARA | **NS** | **NS** | **NS** | **NS** | **H** | **NS** | **NS** | **NS** | **NS** | **NS** | **NS** | **NS** | **NS** |
| RAJASTHAN | RAJSAMAND | **H** | **NS** | **NS** | **NS** | **NS** | **NS** | **NS** | **NS** | **NS** | **NS** | **NS** | **NS** | **NS** |
| RAJASTHAN | DUNGARPUR | **NS** | **NS** | **NS** | **NS** | **NS** | **NS** | **NS** | **NA** | **NS** | **NS** | **NS** | **NS** | **NA** |
| RAJASTHAN | BANSWARA | **H** | **L** | **NS** | **NS** | **NS** | **NS** | **NS** | **NS** | **NS** | **NS** | **NS** | **NS** | **NS** |
| RAJASTHAN | CHITTAURGARH | **NS** | **NS** | **NS** | **NS** | **NS** | **NS** | **NS** | **NS** | **NS** | **NS** | **NS** | **NS** | **NS** |
| RAJASTHAN | KOTA | **NS** | **NS** | **NS** | **NS** | **NS** | **H** | **NS** | **NS** | **NS** | **NS** | **NS** | **NS** | **NS** |
| RAJASTHAN | BARAN | **NS** | **NS** | **NS** | **NS** | **NS** | **NS** | **NS** | **NS** | **NS** | **NS** | **NS** | **NS** | **NA** |
| RAJASTHAN | JHALAWAR | **NS** | **NS** | **NS** | **NS** | **NS** | **NS** | **NS** | **NS** | **NS** | **NS** | **NS** | **H** | **NS** |
| RAJASTHAN | UDAIPUR | **H** | **L** | **NS** | **NS** | **NS** | **NS** | **NS** | **L** | **NS** | **NS** | **NS** | **H** | **NS** |
| RAJASTHAN | PRATAPGARH | **NS** | **NS** | **NS** | **NS** | **NS** | **NS** | **NS** | **NS** | **NS** | **NS** | **NS** | **NS** | **NA** |
| SIKKIM | NORTH DISTRICT | **NS** | **NS** | **NS** | **NS** | **NS** | **NS** | **NS** | **NS** | **NS** | **NS** | **NS** | **NS** | **NS** |
| SIKKIM | WEST DISTRICT | **NS** | **NS** | **NS** | **NS** | **NS** | **NS** | **NS** | **NS** | **NS** | **NS** | **NS** | **NS** | **NS** |
| SIKKIM | SOUTH DISTRICT | **NS** | **NS** | **NS** | **NS** | **NS** | **NS** | **NS** | **NS** | **NS** | **NS** | **NS** | **NS** | **NS** |
| SIKKIM | EAST DISTRICT | **NS** | **NS** | **NS** | **NS** | **NS** | **NS** | **NS** | **NS** | **NS** | **NS** | **NS** | **NS** | **NS** |
| TAMIL NADU | THIRUVALLUR | **NS** | **L** | **NS** | **NS** | **L** | **L** | **NS** | **NS** | **NS** | **L** | **NS** | **H** | **NS** |
| TAMIL NADU | CHENNAI | **NS** | **NS** | **NS** | **H** | **NS** | **NS** | **NS** | **NA** | **NS** | **NS** | **H** | **NS** | **NS** |
| TAMIL NADU | KANCHEEPURAM | **NS** | **L** | **H** | **NS** | **NS** | **NS** | **NS** | **NS** | **NS** | **L** | **H** | **NS** | **NS** |
| TAMIL NADU | VELLORE | **H** | **L** | **NS** | **NS** | **H** | **NS** | **NS** | **H** | **L** | **NS** | **NS** | **NS** | **L** |
| TAMIL NADU | TIRUVANNAMALAI | **H** | **NS** | **NS** | **NS** | **NS** | **NS** | **NS** | **NS** | **NS** | **NS** | **NS** | **NS** | **NS** |
| TAMIL NADU | VILUPPURAM | **NS** | **L** | **NS** | **NS** | **NS** | **NS** | **NS** | **H** | **NS** | **NS** | **NS** | **NS** | **NS** |
| TAMIL NADU | SALEM | **NS** | **L** | **H** | **NS** | **L** | **NS** | **NS** | **NS** | **NS** | **NS** | **H** | **NS** | **NS** |
| TAMIL NADU | NAMAKKAL | **NS** | **L** | **NS** | **NS** | **NS** | **NS** | **NS** | **NS** | **NS** | **NS** | **NS** | **NS** | **NS** |
| TAMIL NADU | ERODE | **H** | **L** | **NS** | **NS** | **L** | **NS** | **L** | **NS** | **NS** | **L** | **H** | **NS** | **L** |
| TAMIL NADU | THE NILGIRIS | **NS** | **NS** | **NS** | **NS** | **NS** | **NS** | **NS** | **NS** | **NS** | **NS** | **NS** | **NS** | **NS** |
| TAMIL NADU | DINDIGUL | **NS** | **NS** | **L** | **NS** | **NS** | **NS** | **NS** | **NS** | **NS** | **NS** | **NS** | **NS** | **NS** |
| TAMIL NADU | KARUR | **NS** | **L** | **NS** | **NS** | **NS** | **NS** | **NS** | **NS** | **NS** | **NS** | **H** | **NS** | **NS** |
| TAMIL NADU | TIRUCHIRAPPALLI | **NS** | **NS** | **L** | **NS** | **NS** | **NS** | **NS** | **NS** | **NS** | **NS** | **H** | **H** | **NS** |
| TAMIL NADU | PERAMBALUR | **NS** | **NS** | **NS** | **NS** | **NS** | **NS** | **NS** | **NS** | **NS** | **NS** | **NS** | **NS** | **NS** |
| TAMIL NADU | ARIYALUR | **NS** | **NS** | **NS** | **NS** | **NS** | **NS** | **NS** | **NS** | **NS** | **NS** | **NS** | **NS** | **NS** |
| TAMIL NADU | CUDDALORE | **H** | **L** | **H** | **L** | **NS** | **NS** | **NS** | **NS** | **NS** | **NS** | **NS** | **NS** | **NS** |
| TAMIL NADU | NAGAPATTINAM | **NS** | **NS** | **NS** | **NS** | **NS** | **NS** | **NS** | **NS** | **NS** | **NS** | **NS** | **NS** | **NS** |
| TAMIL NADU | THIRUVARUR | **NS** | **NS** | **NS** | **NS** | **NS** | **NS** | **NS** | **NS** | **NS** | **NS** | **NS** | **NS** | **NS** |
| TAMIL NADU | THANJAVUR | **H** | **L** | **NS** | **NS** | **NS** | **H** | **L** | **NS** | **NS** | **NS** | **NS** | **NS** | **NS** |
| TAMIL NADU | PUDUKKOTTAI | **H** | **NS** | **NS** | **NS** | **NS** | **NS** | **NS** | **NS** | **NS** | **NS** | **H** | **NS** | **L** |
| TAMIL NADU | SIVAGANGA | **NS** | **NS** | **NS** | **NS** | **NS** | **NS** | **NS** | **NS** | **NS** | **NS** | **NS** | **NS** | **NS** |
| TAMIL NADU | MADURAI | **H** | **NS** | **NS** | **NS** | **H** | **H** | **NS** | **NS** | **NS** | **NS** | **NS** | **NS** | **NS** |
| TAMIL NADU | THENI | **NS** | **NS** | **NS** | **NS** | **NS** | **NS** | **NS** | **NS** | **NS** | **NS** | **H** | **NS** | **NS** |
| TAMIL NADU | VIRUDHUNAGAR | **H** | **NS** | **NS** | **NS** | **NS** | **NS** | **NS** | **NS** | **NS** | **NS** | **NS** | **NS** | **NS** |
| TAMIL NADU | RAMANATHAPURAM | **H** | **NS** | **NS** | **NS** | **NS** | **NS** | **L** | **H** | **NS** | **NS** | **NS** | **NS** | **NS** |
| TAMIL NADU | THOOTHUKKUDI | **NS** | **L** | **NS** | **NS** | **NS** | **NS** | **NS** | **L** | **NS** | **NS** | **NS** | **H** | **NS** |
| TAMIL NADU | TIRUNELVELI | **NS** | **NS** | **NS** | **NS** | **NS** | **NS** | **NS** | **NS** | **NS** | **H** | **NS** | **NS** | **NS** |
| TAMIL NADU | KANNIYAKUMARI | **NS** | **L** | **NS** | **NS** | **NS** | **NS** | **NS** | **NS** | **NS** | **NS** | **NS** | **NS** | **NS** |
| TAMIL NADU | DHARMAPURI | **H** | **NS** | **NS** | **NS** | **NS** | **NS** | **NS** | **H** | **NS** | **NS** | **NS** | **NS** | **NS** |
| TAMIL NADU | KRISHNAGIRI | **H** | **NS** | **NS** | **NS** | **NS** | **NS** | **L** | **H** | **NS** | **NS** | **NS** | **NS** | **NS** |
| TAMIL NADU | COIMBATORE | **H** | **L** | **L** | **NS** | **NS** | **NS** | **NS** | **NS** | **NS** | **NS** | **NS** | **NS** | **NS** |
| TAMIL NADU | TIRUPPUR | **NS** | **L** | **NS** | **NS** | **NS** | **NS** | **NS** | **NS** | **NS** | **NS** | **NS** | **NS** | **NS** |
| TELANGANA | ADILABAD | **NS** | **NS** | **NS** | **NS** | **NS** | **NS** | **NS** | **NS** | **NS** | **NS** | **NS** | **NS** | **NS** |
| TELANGANA | BHADRADRI KOTHAGUDEM | **NS** | **L** | **NS** | **NS** | **NS** | **NS** | **NS** | **NS** | **NS** | **NS** | **NS** | **NS** | **NS** |
| TELANGANA | HYDERABAD | **NS** | **NS** | **NS** | **H** | **NS** | **NA** | **NS** | **NA** | **NA** | **NS** | **H** | **NS** | **NS** |
| TELANGANA | JAGITIAL | **NS** | **NS** | **NS** | **NS** | **NS** | **NS** | **NS** | **L** | **NS** | **NS** | **NS** | **NS** | **NS** |
| TELANGANA | JANGOAN | **NS** | **NS** | **NS** | **NS** | **NS** | **NS** | **NS** | **NS** | **NS** | **NS** | **NS** | **NS** | **NS** |
| TELANGANA | JAYASHANKAR BHUPALAPALLY | **NS** | **NS** | **NS** | **NS** | **NS** | **NS** | **NS** | **NS** | **NS** | **NS** | **NS** | **NS** | **NS** |
| TELANGANA | JOGULAMBA GADWAL | **NS** | **NS** | **NS** | **NS** | **NS** | **NS** | **NS** | **NS** | **NS** | **NS** | **NS** | **NS** | **NS** |
| TELANGANA | KAMAREDDY | **NS** | **NS** | **NS** | **NS** | **NS** | **NS** | **NS** | **NS** | **NS** | **NS** | **NS** | **NS** | **NS** |
| TELANGANA | KARIMNAGAR | **NS** | **NS** | **NS** | **NS** | **NS** | **NS** | **NS** | **NS** | **NS** | **NS** | **NS** | **NS** | **NS** |
| TELANGANA | KHAMMAM | **NS** | **NS** | **NS** | **NS** | **NS** | **NS** | **L** | **L** | **L** | **NS** | **NS** | **H** | **NS** |
| TELANGANA | KOMARAM BHEEM ASIFABAD | **NS** | **NS** | **NS** | **NS** | **NS** | **NS** | **NS** | **NS** | **NS** | **NS** | **NS** | **NS** | **NS** |
| TELANGANA | MAHABUBABAD | **NS** | **NS** | **NS** | **NS** | **NS** | **NS** | **NS** | **NS** | **NS** | **NS** | **NS** | **NS** | **NS** |
| TELANGANA | MAHABUBNAGAR | **H** | **NS** | **NS** | **NS** | **NS** | **NS** | **L** | **NS** | **NS** | **NS** | **NS** | **NS** | **NS** |
| TELANGANA | MANCHERIAL | **NS** | **NS** | **NS** | **NS** | **NS** | **NS** | **NS** | **L** | **NS** | **NS** | **NS** | **NS** | **NS** |
| TELANGANA | MEDAK | **NS** | **NS** | **NS** | **NS** | **NS** | **NS** | **NS** | **NS** | **NS** | **NS** | **NS** | **NS** | **NS** |
| TELANGANA | MEDCHAL-MALKAJGIRI | **NS** | **NS** | **NS** | **NS** | **NS** | **NS** | **NS** | **NS** | **H** | **NS** | **NS** | **NS** | **NS** |
| TELANGANA | NAGARKURNOOL | **NS** | **L** | **NS** | **NS** | **NS** | **NS** | **NS** | **L** | **NS** | **NS** | **H** | **NS** | **NS** |
| TELANGANA | NALGONDA | **NS** | **NS** | **NS** | **NS** | **NS** | **NS** | **L** | **NS** | **NS** | **NS** | **H** | **NS** | **NS** |
| TELANGANA | NIRMAL | **NS** | **L** | **NS** | **NS** | **NS** | **NS** | **NS** | **NS** | **NS** | **NS** | **NS** | **NS** | **NS** |
| TELANGANA | NIZAMABAD | **H** | **L** | **NS** | **NS** | **NS** | **NS** | **NS** | **NS** | **NS** | **NS** | **NS** | **NS** | **NS** |
| TELANGANA | PEDDAPALLI | **NS** | **NS** | **NS** | **NS** | **NS** | **NS** | **NS** | **NS** | **NS** | **NS** | **NS** | **NS** | **NS** |
| TELANGANA | RAJANNA SIRCILLA | **NS** | **NS** | **NS** | **NS** | **NS** | **NS** | **NS** | **NS** | **NS** | **NS** | **NS** | **NS** | **NS** |
| TELANGANA | RANGA REDDY | **NS** | **NS** | **NS** | **NS** | **NS** | **NS** | **L** | **NS** | **L** | **NS** | **NS** | **NS** | **NS** |
| TELANGANA | SANGAREDDY | **H** | **NS** | **NS** | **NS** | **NS** | **NS** | **L** | **NS** | **NS** | **NS** | **NS** | **NS** | **NS** |
| TELANGANA | SIDDIPET | **NS** | **NS** | **NS** | **L** | **NS** | **NS** | **NS** | **NS** | **NS** | **NS** | **NS** | **NS** | **NS** |
| TELANGANA | SURYAPET | **NS** | **NS** | **NS** | **NS** | **L** | **L** | **NS** | **NS** | **NS** | **NS** | **NS** | **NS** | **NS** |
| TELANGANA | VIKARABAD | **NS** | **L** | **NS** | **NS** | **NS** | **NS** | **NS** | **NS** | **L** | **NS** | **NS** | **NS** | **NS** |
| TELANGANA | WANAPARTHY | **NS** | **NS** | **NS** | **NS** | **NS** | **NS** | **NS** | **NS** | **NS** | **NS** | **NS** | **NS** | **NS** |
| TELANGANA | WARANGAL RURAL | **NS** | **L** | **NS** | **NS** | **NS** | **NS** | **NS** | **L** | **L** | **NS** | **NS** | **NS** | **NS** |
| TELANGANA | WARANGAL URBAN | **NS** | **L** | **NS** | **NS** | **NS** | **NS** | **H** | **NS** | **NS** | **NS** | **NS** | **NS** | **NS** |
| TELANGANA | YADADRI BHUVANAGIRI | **NS** | **NS** | **NS** | **NS** | **NS** | **NS** | **NS** | **NS** | **NS** | **NS** | **NS** | **NS** | **NS** |
| TRIPURA | DHALAI | **NS** | **NS** | **L** | **NS** | **NS** | **NS** | **NS** | **NS** | **NS** | **NS** | **NS** | **NS** | **NS** |
| TRIPURA | GOMATI | **NS** | **NS** | **NS** | **NS** | **NS** | **NS** | **NS** | **NS** | **NS** | **NS** | **NS** | **NS** | **NS** |
| TRIPURA | KHOWAI | **NS** | **L** | **NS** | **NS** | **NS** | **NS** | **NS** | **NS** | **NS** | **NS** | **NS** | **NS** | **NS** |
| TRIPURA | NORTH TRIPURA | **NS** | **NS** | **NS** | **NS** | **NS** | **NS** | **NS** | **NS** | **NS** | **NS** | **NS** | **NS** | **NS** |
| TRIPURA | SEPAHIJALA | **NS** | **NS** | **NS** | **NS** | **NS** | **NS** | **NS** | **NS** | **NS** | **NS** | **NS** | **NS** | **NS** |
| TRIPURA | SOUTH TRIPURA | **NS** | **NS** | **NS** | **NS** | **NS** | **NS** | **NS** | **NS** | **NS** | **NS** | **NS** | **NS** | **NS** |
| TRIPURA | UNAKOTI | **NS** | **NS** | **NS** | **NS** | **NS** | **NS** | **NS** | **NS** | **NS** | **NS** | **NS** | **NS** | **NS** |
| TRIPURA | WEST TRIPURA | **NS** | **NS** | **NS** | **NS** | **NS** | **NS** | **NS** | **NS** | **NS** | **NS** | **NS** | **L** | **NS** |
| UTTAR PRADESH | SAHARANPUR | **NS** | **NS** | **NS** | **NS** | **NS** | **NS** | **NS** | **NS** | **NS** | **NS** | **NS** | **NS** | **NA** |
| UTTAR PRADESH | BIJNOR | **NS** | **L** | **NS** | **NS** | **NS** | **NS** | **NS** | **NS** | **NS** | **NS** | **H** | **NS** | **NS** |
| UTTAR PRADESH | RAMPUR | **NS** | **L** | **NS** | **NS** | **NS** | **NS** | **NS** | **NS** | **NS** | **NS** | **NS** | **H** | **NS** |
| UTTAR PRADESH | JYOTIBA PHULE NAGAR | **NS** | **L** | **NS** | **NS** | **NS** | **NS** | **NS** | **NS** | **NS** | **NS** | **NS** | **NS** | **NS** |
| UTTAR PRADESH | MEERUT | **H** | **L** | **NS** | **NS** | **NS** | **NS** | **NS** | **NS** | **NS** | **L** | **H** | **NS** | **NS** |
| UTTAR PRADESH | BAGHPAT | **NS** | **L** | **NS** | **NS** | **NS** | **NS** | **NS** | **NS** | **NS** | **NS** | **NS** | **NS** | **NS** |
| UTTAR PRADESH | GAUTAM BUDDHA NAGAR | **NS** | **NS** | **NS** | **NS** | **NS** | **NS** | **NS** | **NS** | **NS** | **NS** | **NS** | **NS** | **NS** |
| UTTAR PRADESH | BULANDSHAHR | **NS** | **L** | **L** | **NS** | **NS** | **NS** | **NS** | **NS** | **NS** | **NS** | **NS** | **NS** | **NS** |
| UTTAR PRADESH | ALIGARH | **H** | **L** | **H** | **NS** | **NS** | **NS** | **NS** | **NS** | **NS** | **NS** | **H** | **NS** | **NS** |
| UTTAR PRADESH | MAHAMAYA NAGAR | **NS** | **NS** | **NS** | **NS** | **NS** | **NS** | **NS** | **NS** | **NS** | **NS** | **NS** | **NS** | **NS** |
| UTTAR PRADESH | MATHURA | **NS** | **NS** | **NS** | **NS** | **NS** | **NS** | **NS** | **NS** | **NS** | **NS** | **H** | **NS** | **NS** |
| UTTAR PRADESH | AGRA | **H** | **NS** | **NS** | **H** | **NS** | **NS** | **NS** | **L** | **NS** | **NS** | **NS** | **NS** | **NS** |
| UTTAR PRADESH | FIROZABAD | **H** | **L** | **NS** | **L** | **NS** | **NS** | **NS** | **NS** | **NS** | **NS** | **NS** | **NS** | **NS** |
| UTTAR PRADESH | MAINPURI | **H** | **NS** | **NS** | **NS** | **NS** | **NS** | **NS** | **NS** | **NS** | **NS** | **NS** | **NS** | **NS** |
| UTTAR PRADESH | BAREILLY | **NS** | **NS** | **L** | **H** | **NS** | **H** | **NS** | **L** | **NS** | **NS** | **H** | **H** | **NS** |
| UTTAR PRADESH | PILIBHIT | **NS** | **L** | **NS** | **NS** | **NS** | **NS** | **NS** | **NS** | **NS** | **NS** | **NS** | **NS** | **NS** |
| UTTAR PRADESH | SHAHJAHANPUR | **NS** | **NS** | **NS** | **NS** | **NS** | **NS** | **NS** | **NS** | **NS** | **NS** | **NS** | **NS** | **NS** |
| UTTAR PRADESH | KHERI | **NS** | **NS** | **NS** | **NS** | **NS** | **NS** | **L** | **NS** | **NS** | **NS** | **NS** | **NS** | **NS** |
| UTTAR PRADESH | SITAPUR | **NS** | **L** | **L** | **NS** | **H** | **H** | **NS** | **NS** | **L** | **NS** | **NS** | **NS** | **NS** |
| UTTAR PRADESH | HARDOI | **NS** | **L** | **NS** | **NS** | **NS** | **NS** | **NS** | **NS** | **NS** | **NS** | **NS** | **H** | **NS** |
| UTTAR PRADESH | UNNAO | **NS** | **NS** | **NS** | **NS** | **NS** | **NS** | **NS** | **H** | **NS** | **NS** | **NS** | **NS** | **NS** |
| UTTAR PRADESH | LUCKNOW | **NS** | **L** | **NS** | **NS** | **NS** | **NS** | **NS** | **H** | **NS** | **NS** | **H** | **L** | **NS** |
| UTTAR PRADESH | FARRUKHABAD | **NS** | **NS** | **NS** | **H** | **NS** | **NS** | **NS** | **NS** | **NS** | **NS** | **NS** | **NS** | **NS** |
| UTTAR PRADESH | KANNAUJ | **NS** | **L** | **NS** | **NS** | **NS** | **NS** | **NS** | **NS** | **NS** | **NS** | **NS** | **NS** | **NA** |
| UTTAR PRADESH | ETAWAH | **NS** | **NS** | **NS** | **NS** | **NS** | **NS** | **NS** | **NS** | **NS** | **NS** | **H** | **NS** | **NA** |
| UTTAR PRADESH | AURAIYA | **NS** | **NS** | **NS** | **NS** | **NS** | **NS** | **NS** | **NS** | **NS** | **NS** | **NS** | **NS** | **NA** |
| UTTAR PRADESH | KANPUR DEHAT | **NS** | **NS** | **NS** | **NS** | **NS** | **NS** | **NS** | **NS** | **NS** | **NS** | **NS** | **NS** | **NS** |
| UTTAR PRADESH | KANPUR NAGAR | **L** | **L** | **NS** | **H** | **NS** | **NS** | **H** | **L** | **NS** | **NS** | **H** | **NS** | **NS** |
| UTTAR PRADESH | JALAUN | **NS** | **L** | **NS** | **NS** | **NS** | **NS** | **NS** | **NS** | **NS** | **NS** | **NS** | **NS** | **NS** |
| UTTAR PRADESH | JHANSI | **NS** | **NS** | **NS** | **NS** | **NS** | **NS** | **NS** | **NS** | **NS** | **NS** | **NS** | **NS** | **NS** |
| UTTAR PRADESH | LALITPUR | **NS** | **NS** | **NS** | **NS** | **NS** | **NS** | **NS** | **NS** | **NS** | **NS** | **NS** | **NS** | **NS** |
| UTTAR PRADESH | HAMIRPUR | **NS** | **NS** | **NS** | **NS** | **NS** | **NS** | **NS** | **NS** | **NS** | **NS** | **NS** | **NS** | **NS** |
| UTTAR PRADESH | MAHOBA | **NS** | **NS** | **NS** | **NS** | **NS** | **NS** | **NS** | **NS** | **NS** | **NS** | **NS** | **NS** | **NA** |
| UTTAR PRADESH | BANDA | **NS** | **L** | **L** | **NS** | **NS** | **NS** | **NS** | **NS** | **NS** | **NS** | **NS** | **NS** | **NS** |
| UTTAR PRADESH | CHITRAKOOT | **NS** | **NS** | **NS** | **NS** | **NS** | **NS** | **NS** | **NS** | **NS** | **NS** | **NS** | **NS** | **NS** |
| UTTAR PRADESH | FATEHPUR | **NS** | **L** | **NS** | **NS** | **NS** | **NS** | **NS** | **NS** | **NS** | **NS** | **H** | **NS** | **NS** |
| UTTAR PRADESH | PRATAPGARH | **NS** | **L** | **NS** | **NS** | **NS** | **NS** | **NS** | **NS** | **NS** | **NS** | **H** | **NS** | **NS** |
| UTTAR PRADESH | KAUSHAMBI | **NS** | **L** | **NS** | **NS** | **NS** | **NS** | **NS** | **NS** | **NS** | **NS** | **NS** | **NS** | **NS** |
| UTTAR PRADESH | ALLAHABAD | **H** | **NS** | **NS** | **NS** | **NS** | **NS** | **L** | **L** | **H** | **NS** | **NS** | **H** | **NS** |
| UTTAR PRADESH | BARA BANKI | **NS** | **L** | **NS** | **NS** | **NS** | **L** | **NS** | **NS** | **NS** | **NS** | **H** | **NS** | **NS** |
| UTTAR PRADESH | FAIZABAD | **H** | **L** | **NS** | **NS** | **NS** | **L** | **NS** | **NS** | **NS** | **NS** | **NS** | **NS** | **L** |
| UTTAR PRADESH | AMBEDKAR NAGAR | **NS** | **NS** | **NS** | **NS** | **NS** | **NS** | **NS** | **NS** | **NS** | **NS** | **NS** | **NS** | **NS** |
| UTTAR PRADESH | BAHRAICH | **NS** | **NS** | **NS** | **NS** | **NS** | **NS** | **NS** | **NS** | **NS** | **NS** | **NS** | **NS** | **NS** |
| UTTAR PRADESH | SHRAWASTI | **NS** | **NS** | **L** | **NS** | **NS** | **NS** | **NS** | **NS** | **NS** | **NS** | **NS** | **NS** | **NS** |
| UTTAR PRADESH | BALRAMPUR | **NS** | **L** | **NS** | **NS** | **NS** | **NS** | **NS** | **NS** | **L** | **NS** | **NS** | **H** | **NS** |
| UTTAR PRADESH | GONDA | **NS** | **L** | **NS** | **NS** | **NS** | **NS** | **NS** | **NS** | **NS** | **NS** | **NS** | **NS** | **NS** |
| UTTAR PRADESH | SIDDHARTHNAGAR | **NS** | **NS** | **NS** | **NS** | **NS** | **NS** | **NS** | **H** | **NS** | **NS** | **NS** | **NS** | **NS** |
| UTTAR PRADESH | BASTI | **NS** | **NS** | **L** | **NS** | **NS** | **L** | **NS** | **NS** | **NS** | **NS** | **NS** | **NS** | **NS** |
| UTTAR PRADESH | SANT KABIR NAGAR | **NS** | **NS** | **NS** | **NS** | **NS** | **NS** | **NS** | **NS** | **NS** | **NS** | **NS** | **NS** | **NS** |
| UTTAR PRADESH | MAHRAJGANJ | **NS** | **NS** | **NS** | **NS** | **NS** | **NS** | **NS** | **NS** | **NS** | **NS** | **NS** | **NS** | **NS** |
| UTTAR PRADESH | GORAKHPUR | **NS** | **NS** | **L** | **NS** | **NS** | **NS** | **L** | **L** | **NS** | **NS** | **H** | **NS** | **NS** |
| UTTAR PRADESH | KUSHINAGAR | **NS** | **NS** | **NS** | **NS** | **NS** | **NS** | **NS** | **NS** | **NS** | **NS** | **H** | **NS** | **NS** |
| UTTAR PRADESH | DEORIA | **NS** | **NS** | **NS** | **NS** | **NS** | **NS** | **NS** | **NS** | **NS** | **NS** | **NS** | **NS** | **NA** |
| UTTAR PRADESH | AZAMGARH | **NS** | **L** | **NS** | **NS** | **NS** | **NS** | **NS** | **NS** | **NS** | **NS** | **NS** | **NS** | **NS** |
| UTTAR PRADESH | MAU | **NS** | **NS** | **NS** | **NS** | **NS** | **NS** | **NS** | **NS** | **NS** | **NS** | **NS** | **H** | **NS** |
| UTTAR PRADESH | BALLIA | **NS** | **NS** | **NS** | **NS** | **NS** | **NS** | **NS** | **NS** | **NS** | **NS** | **NS** | **NS** | **NS** |
| UTTAR PRADESH | JAUNPUR | **H** | **L** | **H** | **NS** | **L** | **L** | **NS** | **NS** | **L** | **NS** | **NS** | **NS** | **NS** |
| UTTAR PRADESH | GHAZIPUR | **NS** | **L** | **NS** | **NS** | **NS** | **NS** | **NS** | **NS** | **NS** | **NS** | **NS** | **NS** | **NS** |
| UTTAR PRADESH | CHANDAULI | **NS** | **NS** | **NS** | **NS** | **NS** | **NS** | **NS** | **NS** | **NS** | **NS** | **NS** | **NS** | **NS** |
| UTTAR PRADESH | VARANASI | **NS** | **L** | **NS** | **L** | **NS** | **NS** | **NS** | **H** | **NS** | **NS** | **NS** | **H** | **NS** |
| UTTAR PRADESH | SANT RAVIDAS NAGAR (BHADOHI) | **NS** | **L** | **NS** | **NS** | **NS** | **NS** | **NS** | **NS** | **NS** | **NS** | **NS** | **NS** | **NS** |
| UTTAR PRADESH | MIRZAPUR | **NS** | **L** | **L** | **NS** | **NS** | **NS** | **NS** | **NS** | **NS** | **NS** | **NS** | **NS** | **NS** |
| UTTAR PRADESH | SONBHADRA | **NS** | **NS** | **NS** | **NS** | **NS** | **NS** | **NS** | **NS** | **NS** | **NS** | **NS** | **NS** | **NS** |
| UTTAR PRADESH | ETAH | **NS** | **NS** | **NS** | **NS** | **NS** | **NS** | **NS** | **NS** | **NS** | **NS** | **NS** | **NS** | **NS** |
| UTTAR PRADESH | KANSHIRAM NAGAR | **NS** | **NS** | **NS** | **NS** | **NS** | **NS** | **NS** | **NS** | **NS** | **NS** | **NS** | **NS** | **NA** |
| UTTAR PRADESH | AMETHI | **NS** | **NS** | **NS** | **NS** | **NS** | **NS** | **NS** | **NS** | **NS** | **NS** | **H** | **NS** | **NS** |
| UTTAR PRADESH | BUDAUN | **NS** | **L** | **NS** | **L** | **NS** | **H** | **NS** | **L** | **NS** | **NS** | **NS** | **NS** | **NS** |
| UTTAR PRADESH | GHAZIABAD | **H** | **NS** | **L** | **NS** | **NS** | **NS** | **L** | **NS** | **NS** | **NS** | **NS** | **NS** | **L** |
| UTTAR PRADESH | HAPUR | **NS** | **NS** | **NS** | **NS** | **NS** | **NS** | **NS** | **H** | **NS** | **NS** | **NS** | **NS** | **NS** |
| UTTAR PRADESH | MORADABAD | **NS** | **NS** | **NS** | **NS** | **NS** | **NS** | **NS** | **NS** | **NS** | **NS** | **NS** | **NS** | **NS** |
| UTTAR PRADESH | MUZAFFARNAGAR | **NS** | **NS** | **NS** | **H** | **NS** | **NS** | **NS** | **NS** | **NS** | **NS** | **NS** | **H** | **NS** |
| UTTAR PRADESH | RAE BARELI | **NS** | **NS** | **NS** | **NS** | **NS** | **NS** | **NS** | **NS** | **NS** | **NS** | **NS** | **NS** | **NS** |
| UTTAR PRADESH | SAMBHAL | **H** | **L** | **NS** | **NS** | **NS** | **NS** | **NS** | **NS** | **NS** | **NS** | **NS** | **NS** | **NS** |
| UTTAR PRADESH | SHAMLI | **NS** | **NS** | **NS** | **NS** | **NS** | **NS** | **NS** | **NS** | **NS** | **NS** | **NS** | **NS** | **NS** |
| UTTAR PRADESH | SULTANPUR | **NS** | **NS** | **NS** | **H** | **NS** | **NS** | **NS** | **NS** | **NS** | **NS** | **H** | **NS** | **NS** |
| UTTARAKHAND | UTTARKASHI | **NS** | **NS** | **NS** | **NS** | **NS** | **NS** | **NS** | **NS** | **NS** | **NS** | **NS** | **NS** | **NS** |
| UTTARAKHAND | CHAMOLI | **NS** | **NS** | **NS** | **NS** | **NS** | **NS** | **NS** | **NS** | **NS** | **NS** | **NS** | **NS** | **NS** |
| UTTARAKHAND | RUDRAPRAYAG | **NS** | **NS** | **NS** | **NS** | **NS** | **NS** | **NS** | **NS** | **NS** | **NS** | **NS** | **NS** | **NS** |
| UTTARAKHAND | TEHRI GARHWAL | **NS** | **NS** | **NS** | **NS** | **NS** | **NS** | **NS** | **NS** | **NS** | **NS** | **NS** | **NS** | **NS** |
| UTTARAKHAND | DEHRADUN | **NS** | **NS** | **NS** | **NS** | **NS** | **NS** | **NS** | **NS** | **NS** | **NS** | **NS** | **NS** | **NS** |
| UTTARAKHAND | GARHWAL | **NS** | **NS** | **NS** | **NS** | **NS** | **NS** | **NS** | **NS** | **NS** | **NS** | **NS** | **NS** | **NS** |
| UTTARAKHAND | PITHORAGARH | **NS** | **NS** | **NS** | **NS** | **NS** | **NS** | **NS** | **NS** | **NS** | **NS** | **NS** | **NS** | **NS** |
| UTTARAKHAND | BAGESHWAR | **NS** | **NS** | **NS** | **NS** | **NS** | **NS** | **NS** | **NS** | **NS** | **NS** | **NS** | **NS** | **NS** |
| UTTARAKHAND | ALMORA | **NS** | **NS** | **NS** | **NS** | **NS** | **NS** | **NS** | **NS** | **NS** | **NS** | **NS** | **NS** | **NS** |
| UTTARAKHAND | CHAMPAWAT | **NS** | **NS** | **NS** | **NS** | **NS** | **NS** | **NS** | **NS** | **NS** | **NS** | **NS** | **NS** | **NS** |
| UTTARAKHAND | NAINITAL | **NS** | **NS** | **NS** | **NS** | **NS** | **NS** | **NS** | **NS** | **NS** | **NS** | **NS** | **NS** | **NS** |
| UTTARAKHAND | UDHAM SINGH NAGAR | **NS** | **NS** | **NS** | **NS** | **NS** | **NS** | **NS** | **NS** | **NS** | **NS** | **NS** | **NS** | **NS** |
| UTTARAKHAND | HARDWAR | **NS** | **NS** | **NS** | **NS** | **L** | **NS** | **NS** | **NS** | **NS** | **NS** | **NS** | **NS** | **NS** |
| WEST BENGAL | DARJILING | **H** | **NS** | **NS** | **NS** | **H** | **H** | **NS** | **L** | **NS** | **NS** | **NS** | **NS** | **NS** |
| WEST BENGAL | JALPAIGURI | **H** | **L** | **NS** | **NS** | **L** | **L** | **NS** | **H** | **NS** | **NS** | **NS** | **H** | **NS** |
| WEST BENGAL | KOCH BIHAR | **H** | **NS** | **NS** | **NS** | **NS** | **NS** | **NS** | **NS** | **H** | **NS** | **NS** | **NS** | **NS** |
| WEST BENGAL | UTTAR DINAJPUR | **H** | **NS** | **H** | **NS** | **NS** | **NS** | **L** | **NS** | **NS** | **H** | **NS** | **NS** | **NS** |
| WEST BENGAL | DAKSHIN DINAJPUR | **NS** | **L** | **H** | **NS** | **NS** | **NS** | **NS** | **H** | **NS** | **NS** | **NS** | **NS** | **NS** |
| WEST BENGAL | MALDAH | **H** | **L** | **NS** | **NS** | **NS** | **NS** | **NS** | **L** | **H** | **L** | **H** | **NS** | **NS** |
| WEST BENGAL | MURSHIDABAD | **H** | **L** | **NS** | **H** | **L** | **H** | **H** | **H** | **NS** | **NS** | **NS** | **NS** | **NS** |
| WEST BENGAL | BIRBHUM | **L** | **NS** | **NS** | **NS** | **NS** | **NS** | **NS** | **NS** | **H** | **L** | **H** | **NS** | **NS** |
| WEST BENGAL | NADIA | **NS** | **L** | **NS** | **NS** | **NS** | **NS** | **NS** | **H** | **NS** | **NS** | **NS** | **NS** | **H** |
| WEST BENGAL | NORTH TWENTY FOUR PARGANAS | **H** | **L** | **H** | **NS** | **NS** | **H** | **L** | **H** | **L** | **L** | **L** | **L** | **H** |
| WEST BENGAL | HUGLI | **NS** | **L** | **L** | **NS** | **NS** | **NS** | **H** | **H** | **NS** | **L** | **H** | **L** | **NS** |
| WEST BENGAL | BANKURA | **NS** | **H** | **NS** | **H** | **NS** | **NS** | **NS** | **L** | **NS** | **H** | **NS** | **H** | **NS** |
| WEST BENGAL | PURULIYA | **NS** | **L** | **NS** | **NS** | **NS** | **NS** | **H** | **NS** | **NS** | **NS** | **NS** | **NS** | **NS** |
| WEST BENGAL | HAORA | **NS** | **NS** | **H** | **NS** | **NS** | **NS** | **NS** | **NS** | **NS** | **NS** | **NS** | **L** | **L** |
| WEST BENGAL | KOLKATA | **H** | **NS** | **L** | **NS** | **NS** | **NS** | **NS** | **NA** | **H** | **H** | **L** | **NS** | **NS** |
| WEST BENGAL | SOUTH TWENTY FOUR PARGANAS | **H** | **NS** | **H** | **H** | **L** | **H** | **NS** | **L** | **H** | **H** | **L** | **H** | **NS** |
| WEST BENGAL | PASCHIM MEDINIPUR | **H** | **NS** | **NS** | **H** | **L** | **H** | **NS** | **NS** | **NS** | **H** | **NS** | **NS** | **NS** |
| WEST BENGAL | PURBA MEDINIPUR | **NS** | **L** | **NS** | **H** | **L** | **NS** | **NS** | **NS** | **NS** | **NS** | **NS** | **NS** | **H** |
| WEST BENGAL | PASCHIM BARDDHAMAN | **H** | **L** | **NS** | **NS** | **L** | **L** | **NS** | **NS** | **NS** | **L** | **NS** | **NS** | **NA** |
| WEST BENGAL | PURBA BARDDHAMAN | **H** | **L** | **NS** | **L** | **NS** | **H** | **NS** | **H** | **NS** | **L** | **NS** | **NS** | **NS** |
| H% |  | **15.6** | **0.6** | **3.1** | **6.3** | **3.7** | **4.1** | **3.5** | **5.5** | **3.8** | **3.5** | **13.4** | **7.6** | **1.1** |
| L% |  | **1.7** | **28.1** | **6.9** | **3.7** | **4.0** | **4.1** | **6.1** | **6.9** | **5.5** | **4.0** | **1.5** | **2.9** | **2.4** |

*H: Higher odds [Adjusted odds ratio(AOR) >1 and P<0.05], L: Lower odds (AOR<1 and P<0.05), NS: Not Significant (p>0.05), NA: Data Not Available.*

*Districts from 8 Non significant states excluded from table (ie., Arunachal Pradesh, Nagaland, Mizoram, Dadra & Nagar Haveli And Daman & Diu, Lakshadweep, Puducherry, Andaman & Nicobar Islands, Ladakh)*

## **Table S12: District wise determinants of Raised blood pressure among adults in India (AOR) (National Family Health Survey-5, India, 2019-2021)**

| **State Name** | **District Name** | Age Group (>=30) | Sex (Female) | Education (Literate) | Occupation (Employed) | Household wealth quintile (middle) | Household wealth quintile (richer/richest) | Marital status (Currently married) | Place Of Residence (Urban) | Tobacco consumption (Yes) | Alcohol consumption (Yes) | BMI (Overweight/Obese) | Central obesity (Present) | Blood glucose level (Raised) |
| --- | --- | --- | --- | --- | --- | --- | --- | --- | --- | --- | --- | --- | --- | --- |
| ANDHRA PRADESH | SRIKAKULAM | H | NS | NS | L | L | NS | NS | NS | H | H | H | NS | NS |
| ANDHRA PRADESH | VIZIANAGARAM | H | NS | NS | NS | NS | H | NS | NS | NS | NS | NS | NS | NS |
| ANDHRA PRADESH | VISAKHAPATNAM | H | L | H | NS | NS | NS | L | L | L | H | NS | H | H |
| ANDHRA PRADESH | EAST GODAVARI | H | H | H | NS | NS | NS | NS | L | NS | H | H | NS | NS |
| ANDHRA PRADESH | WEST GODAVARI | H | NS | H | NS | NS | NS | NS | NS | NS | H | NS | H | NS |
| ANDHRA PRADESH | KRISHNA | H | L | NS | L | L | L | NS | NS | H | NS | H | H | NS |
| ANDHRA PRADESH | GUNTUR | H | L | H | NS | L | NS | NS | H | H | NS | H | H | NS |
| ANDHRA PRADESH | PRAKASAM | NS | L | NS | NS | NS | NS | H | NS | NS | H | H | NS | NA |
| ANDHRA PRADESH | SRI POTTI SRIRAMULU NELLORE | H | NS | NS | L | NS | NS | NS | NS | NS | H | H | NS | NS |
| ANDHRA PRADESH | Y.S.R. | H | L | NS | NS | L | NS | NS | L | NS | NS | NS | NS | NS |
| ANDHRA PRADESH | KURNOOL | H | L | NS | L | NS | NS | NS | NS | NS | NS | NS | H | NS |
| ANDHRA PRADESH | ANANTAPUR | H | L | NS | NS | H | NS | H | H | NS | L | NS | NS | NS |
| ANDHRA PRADESH | CHITTOOR | H | NS | NS | NS | L | L | L | L | NS | NS | H | H | NS |
| ASSAM | KOKRAJHAR | NS | NS | NS | NS | NS | NS | NS | H | NS | L | NS | NS | NS |
| ASSAM | GOALPARA | NS | NS | NS | NS | NS | NS | NS | NS | NS | NS | NS | NS | NS |
| ASSAM | BARPETA | NS | NS | NS | NS | NS | NS | NS | NS | NS | NS | NS | H | NS |
| ASSAM | MORIGAON | NS | NS | NS | NS | NS | NS | NS | NS | NS | NS | NS | NS | NS |
| ASSAM | LAKHIMPUR | H | NS | NS | NS | NS | NS | NS | NS | NS | NS | NS | H | NS |
| ASSAM | DHEMAJI | NS | NS | NS | NS | NS | NS | NS | NS | NS | NS | NS | NS | NS |
| ASSAM | TINSUKIA | NS | NS | NS | NS | NS | NS | NS | NS | NS | NS | NS | NS | NS |
| ASSAM | DIBRUGARH | H | NS | NS | NS | NS | NS | NS | L | NS | NS | NS | NS | NS |
| ASSAM | GOLAGHAT | NS | NS | L | NS | H | NS | NS | NS | NS | NS | NS | NS | NS |
| ASSAM | DIMA HASAO | NS | NS | NS | NS | NS | NS | NS | NS | NS | NS | NS | NS | NS |
| ASSAM | CACHAR | H | NS | NS | NS | NS | NS | NS | NS | NS | NS | H | NS | H |
| ASSAM | KARIMGANJ | NS | L | L | NS | NS | NS | NS | NS | NS | H | H | NS | NS |
| ASSAM | HAILAKANDI | NS | NS | NS | NS | NS | NS | NS | NS | NS | NS | H | NS | NS |
| ASSAM | BONGAIGAON | NS | NS | NS | NS | NS | NS | NS | NS | NS | NS | NS | NS | NS |
| ASSAM | CHIRANG | NS | NS | NS | NS | NS | NS | NS | NS | NS | NS | NS | NS | NS |
| ASSAM | KAMRUP | H | NS | NS | NS | NS | H | NS | NS | NS | NS | NS | NS | NS |
| ASSAM | KAMRUP METROPOLITAN | NS | NS | L | NS | NS | NS | NS | NS | H | NS | NS | NS | NS |
| ASSAM | NALBARI | NS | NS | NS | NS | NS | NS | NS | NS | NS | NS | NS | NS | NS |
| ASSAM | BAKSA | H | NS | NS | NS | NS | NS | NS | NA | NS | NS | NS | NS | NS |
| ASSAM | DARRANG | NS | NS | NS | NS | NS | NS | NS | NS | NS | NS | NS | NS | NS |
| ASSAM | UDALGURI | NS | NS | NS | NS | NS | NS | NS | NS | NS | NS | NS | NS | NS |
| ASSAM | BISWANATH | NS | NS | NS | NS | NS | NS | NS | NS | NS | NS | NS | NS | NS |
| ASSAM | CHARAIDEO | NS | NS | NS | NS | NS | NS | NS | NS | NS | NS | NS | NS | NS |
| ASSAM | DHUBRI | NS | NS | NS | NS | NS | NS | H | NS | NS | NS | NS | NS | NS |
| ASSAM | HOJAI | NS | NS | NS | NS | NS | NS | NS | NS | NS | NS | NS | NS | NS |
| ASSAM | JORHAT | NS | NS | NS | NS | L | NS | NS | NS | NS | NS | NS | H | NS |
| ASSAM | KARBI ANGLONG | NS | NS | NS | NS | NS | NS | NS | H | NS | NS | NS | NS | NS |
| ASSAM | MAJULI | NS | NS | NS | NS | NS | NS | NS | NA | NS | NS | NS | NS | NS |
| ASSAM | NAGAON | H | NS | NS | NS | H | NS | NS | NS | NS | NS | H | NS | NS |
| ASSAM | SIVASAGAR | NS | NS | NS | NS | NS | NS | NS | NS | NS | NS | H | NS | NS |
| ASSAM | SONITPUR | NS | NS | NS | NS | NS | NS | NS | NS | NS | NS | H | NS | NS |
| ASSAM | SOUTH SALMARA MANCACHAR | NS | NS | NS | NS | NS | NS | NS | NS | NS | NS | NS | NS | NS |
| ASSAM | WEST KARBI ANGLONG | NS | NS | NS | NS | NS | NS | NS | NS | NS | NS | NS | NS | NS |
| BIHAR | PASHCHIM CHAMPARAN | H | NS | H | NS | NS | NS | NS | H | H | NS | H | NS | NS |
| BIHAR | PURBA CHAMPARAN | NS | NS | NS | NS | H | H | H | NA | NS | H | NS | NS | H |
| BIHAR | SHEOHAR | NS | NS | NS | NS | NS | NS | NS | NS | NS | NS | NS | NS | NS |
| BIHAR | SITAMARHI | NS | NS | NS | NS | L | H | NS | NS | NS | NS | H | NS | NS |
| BIHAR | MADHUBANI | NS | L | L | NS | NS | NS | NS | NA | NS | NS | H | NS | NA |
| BIHAR | SUPAUL | NS | NS | NS | NS | NA | NS | NS | NA | NS | NS | NS | NS | NS |
| BIHAR | ARARIA | H | NS | NS | NS | NS | NS | NS | NS | NS | NS | NS | NS | H |
| BIHAR | KISHANGANJ | NS | NS | NS | NS | NS | NA | NS | NA | NS | NS | NS | NS | NS |
| BIHAR | PURNIA | H | NS | NS | NS | NS | NS | NS | NS | NS | L | H | NS | NA |
| BIHAR | KATIHAR | H | NS | NS | NS | H | H | NS | H | L | NS | NS | NS | NS |
| BIHAR | MADHEPURA | NS | NS | NS | NS | H | NS | NS | NA | NS | NS | H | NS | NS |
| BIHAR | SAHARSA | H | NS | NS | NS | H | NS | NS | NS | NS | H | NS | NS | NS |
| BIHAR | DARBHANGA | H | L | NS | NS | NS | NS | H | L | NS | L | NS | NS | H |
| BIHAR | MUZAFFARPUR | H | L | NS | NS | L | L | NS | H | L | NS | NS | H | H |
| BIHAR | GOPALGANJ | H | NS | NS | NS | H | H | NS | H | NS | NS | NS | H | NS |
| BIHAR | SIWAN | NS | NS | NS | NS | NS | NS | H | L | NS | NS | NS | NS | NS |
| BIHAR | SARAN | NS | NS | NS | NS | H | NS | H | H | NS | NS | NS | NS | H |
| BIHAR | VAISHALI | NS | NS | NS | NS | NS | NS | H | H | NS | NS | NS | NS | NS |
| BIHAR | SAMASTIPUR | H | H | NS | NS | NS | H | NS | NS | H | NS | NS | L | NS |
| BIHAR | BEGUSARAI | H | NS | L | H | NS | NS | NS | NS | NS | NS | H | NS | NS |
| BIHAR | KHAGARIA | NS | NS | NS | NS | NS | H | NS | NS | H | NS | NS | NS | NS |
| BIHAR | BHAGALPUR | NS | H | NS | NS | NS | NS | NS | NS | NS | NS | NS | NS | H |
| BIHAR | BANKA | NS | NS | NS | NS | NS | NS | H | H | NS | NS | H | NS | NS |
| BIHAR | MUNGER | NS | H | NS | NS | NS | NS | NS | NS | NS | NS | NS | NS | NS |
| BIHAR | LAKHISARAI | NS | NS | NS | NS | NS | NS | NS | NS | NS | NS | NS | NS | NS |
| BIHAR | SHEIKHPURA | NS | NS | NS | NS | NS | NS | NS | NS | NS | NS | NS | NS | NS |
| BIHAR | NALANDA | H | NS | NS | NS | H | NS | NS | NS | NS | NS | NS | NS | NS |
| BIHAR | PATNA | NS | NS | NS | NS | NS | L | NS | H | H | NS | H | H | NS |
| BIHAR | BHOJPUR | H | NS | NS | NS | NS | L | NS | NS | NS | NS | NS | H | NS |
| BIHAR | BUXAR | H | NS | NS | NS | NS | NS | NS | NA | NS | NS | NS | NS | NS |
| BIHAR | KAIMUR (BHABUA) | NS | NS | NS | NS | NS | H | NS | NS | NS | NS | H | NS | NS |
| BIHAR | ROHTAS | H | NS | NS | NS | NS | NS | L | L | NS | H | NS | NS | NS |
| BIHAR | AURANGABAD | NS | NS | NS | NS | NS | NS | H | NS | NS | NS | NS | NS | NS |
| BIHAR | GAYA | NS | NS | NS | NS | L | NS | H | NS | NS | NS | H | H | NS |
| BIHAR | NAWADA | NS | NS | NS | NS | NS | L | NS | NS | NS | NS | NS | NS | NS |
| BIHAR | JAMUI | NS | NS | NS | NS | NS | NS | NS | H | NS | NA | NS | H | NS |
| BIHAR | JEHANABAD | H | NS | NS | NS | NS | NS | NS | NS | NS | NS | NS | NS | NS |
| BIHAR | ARWAL | NS | NS | NS | NS | NS | NS | NS | NA | NS | NS | NS | NS | NS |
| CHHATTISGARH | KORIYA | NS | NS | NS | NS | NS | NS | NS | NS | NS | NS | NS | NS | NS |
| CHHATTISGARH | JASHPUR | NS | NS | NS | NS | NS | NS | NS | NS | NS | NS | NS | NS | NS |
| CHHATTISGARH | RAIGARH | H | NS | NS | NS | NS | NS | NS | NS | NS | NS | NS | NS | H |
| CHHATTISGARH | KORBA | NS | NS | NS | NS | NS | NS | NS | NS | NS | NS | NS | NS | NS |
| CHHATTISGARH | JANJGIR - CHAMPA | NS | NS | NS | NS | NS | NS | NS | NS | NS | NS | NS | NS | NS |
| CHHATTISGARH | KABEERDHAM | NS | NS | NS | NS | NS | NS | NS | NS | NS | NS | H | NS | NS |
| CHHATTISGARH | RAJNANDGAON | H | NS | NS | NS | NS | NS | NS | NS | NS | NS | NS | NS | NS |
| CHHATTISGARH | MAHASAMUND | NS | NS | NS | NS | NS | NS | NS | NS | NS | NS | NS | NS | NS |
| CHHATTISGARH | DHAMTARI | NS | NS | NS | NS | NS | NS | NS | NS | NS | NS | NS | NS | NS |
| CHHATTISGARH | UTTAR BASTAR KANKER | NS | NS | NS | NS | NS | NS | NS | NS | NS | NS | NS | NS | NS |
| CHHATTISGARH | NARAYANPUR | NS | NS | NS | NS | NS | NS | NS | NS | NS | NS | NS | NS | NS |
| CHHATTISGARH | BIJAPUR | NS | NS | NS | NS | NS | NS | NS | NS | NS | NS | NS | NS | NS |
| CHHATTISGARH | BALOD | NS | NS | NS | NS | NS | NS | NS | NS | NS | NS | NS | NS | NS |
| CHHATTISGARH | BALODA BAZAR | H | NS | NS | L | NS | NS | NS | NS | NS | NS | NS | H | NS |
| CHHATTISGARH | BALRAMPUR | NS | NS | NS | NS | NS | NS | NS | NS | NS | NS | NS | NS | NS |
| CHHATTISGARH | BASTAR | NS | NS | NS | NS | NS | NS | NS | NS | NS | NS | NS | NS | NS |
| CHHATTISGARH | BEMETARA | H | NS | NS | NS | NS | NS | NS | NS | NS | NS | NS | NS | NA |
| CHHATTISGARH | BILASPUR | H | NS | NS | NS | NS | NS | NS | NS | NS | NS | NS | NS | NS |
| CHHATTISGARH | DANTEWADA | NS | NS | NS | NS | NS | NS | NS | NS | NS | NS | NS | NS | NS |
| CHHATTISGARH | DURG | H | NS | NS | NS | NS | NS | NS | NS | NS | NS | NS | H | NS |
| CHHATTISGARH | GARIYABAND | NS | NS | NS | NS | NS | NS | NS | NS | NS | NS | NS | NS | NS |
| CHHATTISGARH | KODAGAON | NS | NS | NS | NS | NS | NS | NS | NS | NS | NS | NS | NS | NS |
| CHHATTISGARH | MUNGELI | NS | NS | NS | NS | NS | NS | NS | NS | NS | NS | NS | NS | NS |
| CHHATTISGARH | RAIPUR | H | NS | NS | NS | NS | NS | NS | NS | NS | NS | NS | NS | NS |
| CHHATTISGARH | SUKMA | NS | NS | NS | NS | NS | NS | NS | NS | NS | NS | NS | NS | NS |
| CHHATTISGARH | SURAJPUR | NS | NS | NS | NS | NS | NS | NS | NS | NS | NS | NS | NS | NS |
| CHHATTISGARH | SURGUJA | H | NS | NS | NS | NS | NS | NS | NS | NS | NS | NS | NS | NS |
| GOA | NORTH GOA | NS | NS | NS | NS | NS | NS | NS | NS | NS | NS | NS | NS | H |
| GOA | SOUTH GOA | H | NS | NS | NS | NS | NS | NS | NS | NS | NS | NS | NS | NS |
| GUJARAT | KACHCHH | H | H | NS | H | NS | H | NS | L | NS | NS | H | H | NS |
| GUJARAT | BANAS KANTHA | NS | NS | NS | NS | NS | NS | NS | NS | NS | NS | NS | H | NA |
| GUJARAT | PATAN | H | NS | NS | NS | NS | NS | NS | H | NS | NS | NS | NS | NS |
| GUJARAT | MAHESANA | H | NS | NS | NS | NS | NS | NS | NS | NS | H | NS | NS | NS |
| GUJARAT | GANDHINAGAR | H | NS | NS | H | NS | H | NS | NS | NS | NS | H | NS | NS |
| GUJARAT | PORBANDAR | NS | NS | NS | NS | NS | NS | NS | NS | NS | NS | NS | NS | NS |
| GUJARAT | AMRELI | NS | NS | NS | NS | H | NS | NS | NS | NS | NA | NS | NS | H |
| GUJARAT | ANAND | NS | NS | NS | NS | NS | NS | NS | NS | NS | NS | NS | NS | NS |
| GUJARAT | DOHAD | H | NS | NS | NS | NS | NS | NS | NS | NS | NS | NS | H | NS |
| GUJARAT | NARMADA | NS | NS | NS | NS | NS | NS | NS | L | NS | NS | NS | NS | NS |
| GUJARAT | BHARUCH | H | NS | NS | NS | NS | NS | NS | NS | NS | NS | NS | NS | NS |
| GUJARAT | THE DANGS | NS | NS | NS | NS | NS | NS | NS | NS | NS | NS | NS | NS | NS |
| GUJARAT | NAVSARI | NS | L | NS | NS | NS | NS | NS | NS | NS | NS | NS | NS | NS |
| GUJARAT | VALSAD | H | L | NS | NS | NS | NS | NS | H | NS | NS | NS | H | H |
| GUJARAT | SURAT | H | L | L | NS | L | NS | H | NS | NS | NS | H | H | NS |
| GUJARAT | TAPI | NS | NS | NS | NS | NS | NS | NS | NS | NS | NS | H | NS | NS |
| GUJARAT | AHMADABAD | NS | L | L | NS | NA | NA | NS | NS | NS | H | H | H | H |
| GUJARAT | ARAVALI | H | NS | NS | NS | NS | NS | NS | NS | NS | NS | NS | NS | NS |
| GUJARAT | BHAVNAGAR | NS | NS | NS | NS | NS | NS | NS | L | NS | H | NS | NS | NS |
| GUJARAT | BOTAD | NS | NS | NS | NS | NS | NS | NS | NS | NS | NS | NS | NS | NS |
| GUJARAT | CHHOTA UDAIPUR | NS | NS | NS | NS | NS | NS | NS | NS | NS | NS | H | NS | NS |
| GUJARAT | DEVBHUMI DWARKA | H | NS | NS | NS | NS | NS | NS | NS | NS | NS | NS | NS | NS |
| GUJARAT | GIR SOMNATH | NS | NS | NS | NS | NS | NS | NS | L | NS | NS | NS | NS | NS |
| GUJARAT | JAMNAGAR | NS | NS | NS | NS | NS | NS | NS | NS | NS | NS | NS | H | NS |
| GUJARAT | JUNAGADH | H | NS | NS | NS | NS | H | L | NS | NS | H | NS | NS | H |
| GUJARAT | KHEDA | H | NS | NS | NS | NS | NS | NS | NS | H | H | H | NS | NS |
| GUJARAT | MAHISAGAR | H | NS | NS | NS | NS | NS | NS | NS | NS | NS | NS | NS | NS |
| GUJARAT | MORBI | H | NS | NS | NS | NS | NS | NS | NS | NS | NS | NS | NS | NS |
| GUJARAT | PANCH MAHALS | NS | NS | NS | NS | NS | NS | NS | NS | NS | NS | NS | NS | NS |
| GUJARAT | RAJKOT | L | NS | L | H | NA | NA | NA | L | NS | NS | NS | NS | NS |
| GUJARAT | SABAR KANTHA | H | L | NS | NS | NS | NS | NS | NS | NS | NS | NS | NS | NA |
| GUJARAT | SURENDRANAGAR | NS | NS | NS | H | NS | NS | NS | NS | NS | NA | NS | NS | NS |
| GUJARAT | VADODARA | H | NS | NS | NS | NS | NS | NS | NS | NS | NS | NS | H | H |
| HARYANA | PANCHKULA | H | NS | NS | NS | NS | NA | NS | NS | NS | NS | NS | NS | NS |
| HARYANA | AMBALA | NS | NS | NS | NS | NS | NS | NS | NS | NS | NS | NS | NS | NS |
| HARYANA | YAMUNANAGAR | NS | NS | NS | NS | NS | NS | NS | NS | NS | NS | NS | NS | NS |
| HARYANA | KURUKSHETRA | NS | NS | NS | NS | NS | NS | NS | NS | NS | NS | NS | NS | NS |
| HARYANA | KAITHAL | NA | NS | NS | NS | NS | NS | NS | NS | NS | NS | NS | NS | NS |
| HARYANA | KARNAL | NS | NS | NS | NS | NS | NS | H | NS | NS | NS | NS | NS | H |
| HARYANA | PANIPAT | NS | NS | NS | NS | NS | NS | NS | NS | NS | NS | NS | NS | NS |
| HARYANA | SONIPAT | H | NS | NS | NS | NS | NS | NS | NS | NS | NS | NS | NS | H |
| HARYANA | JIND | NS | NS | NS | NS | NS | NS | NS | NS | NS | NS | NS | NS | NS |
| HARYANA | FATEHABAD | NS | NS | NS | NS | NS | NS | NS | NS | NS | NS | NS | NS | NA |
| HARYANA | SIRSA | NS | NS | NS | NS | NS | NS | NS | NA | NS | NS | NS | NS | NS |
| HARYANA | HISAR | NS | L | NS | NS | NS | NS | NS | NS | NS | NS | NS | NS | NS |
| HARYANA | ROHTAK | NS | NS | NS | NS | NS | NS | NS | NS | NS | NS | NS | NS | NS |
| HARYANA | JHAJJAR | NS | NS | NS | NS | NS | NS | NS | NS | NS | NS | NS | NS | NS |
| HARYANA | MAHENDRAGARH | NS | NS | NS | NS | NS | NS | NS | NS | NS | NS | NS | NS | NS |
| HARYANA | REWARI | NS | NS | NS | NS | NS | NS | NS | NS | NS | NS | NS | NS | NS |
| HARYANA | GURGAON | NS | NA | NS | NA | NS | NS | NA | NA | NS | NS | NS | NS | NS |
| HARYANA | MEWAT | NS | NS | NS | NS | NS | NS | NS | NS | NS | NS | NS | NS | NS |
| HARYANA | FARIDABAD | NS | NS | NS | NS | NS | NS | NS | L | NS | NS | NS | NS | NS |
| HARYANA | PALWAL | NS | NS | NS | NS | NS | NS | NS | NS | NS | NS | NS | NS | NS |
| HARYANA | BHIWANI | NS | L | NS | NS | NS | NS | NS | NS | NS | NS | NS | NS | NS |
| HARYANA | CHARKHI DADRI | NS | NS | NS | NS | NS | NS | NS | NS | NS | NS | NS | NS | NS |
| HIMACHAL PRADESH | CHAMBA | NS | NS | NS | NS | NS | NS | NS | NS | NS | NS | NS | NS | NA |
| HIMACHAL PRADESH | KANGRA | H | NS | NS | H | NS | NS | NS | NS | NS | NS | NS | H | NS |
| HIMACHAL PRADESH | LAHUL & SPITI | NS | NS | NS | NS | NS | NS | NS | NA | NS | NS | NS | NS | NA |
| HIMACHAL PRADESH | KULLU | NS | NS | NS | NS | NS | NS | NS | NS | NS | NS | NS | NS | NS |
| HIMACHAL PRADESH | MANDI | NS | L | NS | NS | L | NS | NS | NA | NS | NS | NS | NS | NS |
| HIMACHAL PRADESH | HAMIRPUR | NS | NS | NS | NS | NS | NS | NS | NS | NS | NS | NS | NS | NS |
| HIMACHAL PRADESH | UNA | NS | NS | NS | NS | NS | NS | NS | NS | NS | NS | NS | NS | NS |
| HIMACHAL PRADESH | BILASPUR | NS | NS | NS | NS | NS | NS | NS | NS | NS | NS | NS | NS | NS |
| HIMACHAL PRADESH | SOLAN | NS | NS | NS | NS | NS | NS | NS | NS | NS | NS | NS | NS | NS |
| HIMACHAL PRADESH | SIRMAUR | NS | NS | NS | NS | NS | NS | NS | NS | NS | NS | NS | NS | NS |
| HIMACHAL PRADESH | SHIMLA | NS | NS | NS | NS | NS | NS | NS | NS | NS | NS | NS | NS | NS |
| HIMACHAL PRADESH | KINNAUR | NS | NS | NS | NS | NS | NS | NS | NA | NS | NS | NS | NS | NS |
| JAMMU & KASHMIR | KUPWARA | NS | NS | NS | NS | NS | NS | NS | NS | NS | NA | NS | NS | NS |
| JAMMU & KASHMIR | BADGAM | NS | NS | NS | NS | NS | NS | NS | NS | NS | NA | NS | NS | NS |
| JAMMU & KASHMIR | PUNCH | NS | NS | NS | NS | NS | NS | NS | NS | NS | NS | NS | NS | NS |
| JAMMU & KASHMIR | RAJOURI | NS | NS | NS | NS | NS | NS | NS | NS | NS | NS | NS | NS | NS |
| JAMMU & KASHMIR | KATHUA | NS | NS | NS | NS | NS | NS | NS | NS | NS | NS | NS | NS | NA |
| JAMMU & KASHMIR | BARAMULA | NS | NS | NS | NS | NS | NS | NS | NS | NS | NS | NS | NS | NS |
| JAMMU & KASHMIR | BANDIPORE | NS | NS | NS | NS | NS | NS | NS | NS | NS | NA | NS | NS | NS |
| JAMMU & KASHMIR | SRINAGAR | NS | NS | NS | NS | NS | NS | H | NA | NS | NA | NS | NS | NS |
| JAMMU & KASHMIR | GANDERBAL | NS | NS | NS | NS | NS | NS | NS | NS | NS | NA | NS | NS | NS |
| JAMMU & KASHMIR | PULWAMA | NS | NS | NS | NS | NS | NS | NS | NS | NS | NA | NS | NS | NS |
| JAMMU & KASHMIR | SHUPIYAN | NS | NS | NS | NS | NS | NS | NS | NS | NS | NA | NS | NS | NA |
| JAMMU & KASHMIR | ANANTNAG | NS | NS | NS | NS | NS | NS | NS | NS | H | NA | NS | NS | NA |
| JAMMU & KASHMIR | KULGAM | NS | NS | NS | NS | NS | NS | NS | NS | NS | NA | NS | NS | NS |
| JAMMU & KASHMIR | DODA | NS | NS | NS | NS | NS | NS | NS | NS | NS | NS | NS | NS | NA |
| JAMMU & KASHMIR | RAMBAN | NS | NS | NS | NS | NS | NS | NS | NS | NS | NS | NS | NS | NA |
| JAMMU & KASHMIR | KISHTWAR | NS | NS | NS | NS | NS | NS | NS | NS | NS | NS | NS | NS | NS |
| JAMMU & KASHMIR | UDHAMPUR | NS | NS | NS | NS | NS | NS | NS | NS | NS | NS | NS | NS | NS |
| JAMMU & KASHMIR | REASI | NS | NS | NS | NS | NS | NS | NS | NS | NS | NS | NS | NS | NS |
| JAMMU & KASHMIR | JAMMU | NS | NS | NS | NS | NS | NS | H | NS | NS | NS | H | NS | NA |
| JAMMU & KASHMIR | SAMBA | NS | NS | NS | NS | NS | NS | NS | NS | NS | NS | NS | NS | NA |
| JHARKHAND | GARHWA | NS | NS | NS | NS | NS | NS | NS | NS | NS | NS | NS | NS | NS |
| JHARKHAND | CHATRA | NS | NS | NS | NS | NS | NS | NS | NS | NS | NS | NS | NS | NS |
| JHARKHAND | KODARMA | NS | NS | NS | NS | NS | NS | NS | NS | NS | NS | NS | NS | NS |
| JHARKHAND | GIRIDIH | H | NS | NS | NS | NS | H | NS | L | NS | NS | NS | NS | NS |
| JHARKHAND | DEOGHAR | NS | NS | NS | NS | NS | NS | NS | NS | NS | NS | NS | NS | NS |
| JHARKHAND | GODDA | NS | NS | NS | NS | NS | NS | NS | NS | NS | NS | NS | NS | NS |
| JHARKHAND | SAHIBGANJ | H | NS | NS | NS | NS | NS | NS | NS | NS | NS | NS | NS | NS |
| JHARKHAND | PAKUR | NS | NS | NS | NS | NS | NS | NS | NS | NS | NS | NS | NS | NS |
| JHARKHAND | DHANBAD | NS | NS | L | NS | NS | H | NS | NS | NS | NS | NS | NS | NS |
| JHARKHAND | BOKARO | NS | NS | NS | NS | NS | NS | NS | NS | NS | NS | NS | NS | NS |
| JHARKHAND | LOHARDAGA | NS | NS | NS | NS | NS | NS | NS | NS | NS | NS | NS | NS | NS |
| JHARKHAND | PURBI SINGHBHUM | NS | L | NS | L | NS | NS | NS | NS | NS | NS | H | NS | NS |
| JHARKHAND | PALAMU | NS | NS | NS | NS | NS | NS | NS | NS | NS | NS | NS | NS | NS |
| JHARKHAND | LATEHAR | NS | NS | NS | NS | NS | NS | NS | NS | NS | NS | H | NS | NS |
| JHARKHAND | HAZARIBAGH | NS | L | NS | NS | NS | NS | NS | NS | NS | NS | NS | NS | NS |
| JHARKHAND | RAMGARH | NS | NS | NS | NS | NS | NS | NS | NS | NS | NS | NS | NS | NS |
| JHARKHAND | DUMKA | NS | NS | NS | NS | NS | NS | NS | NS | NS | NS | NS | NS | NS |
| JHARKHAND | JAMTARA | NS | NS | NS | NS | NS | NS | NS | NS | NS | NS | NS | NS | NS |
| JHARKHAND | RANCHI | NS | NS | NS | NS | NS | NS | NS | NS | L | H | H | NS | NS |
| JHARKHAND | KHUNTI | NS | NS | NS | NS | NS | NS | NS | NS | NS | NS | NS | NS | NA |
| JHARKHAND | GUMLA | NS | NS | NS | NS | NS | NS | NS | NS | NS | NS | NS | H | NS |
| JHARKHAND | SIMDEGA | NS | NS | NS | NS | NS | NS | NS | NS | NS | NS | NS | NS | NS |
| JHARKHAND | PASHCHIMI SINGHBHUM | NS | NS | NS | NS | NS | NS | NS | L | NS | NS | NS | NS | NS |
| JHARKHAND | SARAIKELA-KHARSAWAN | NS | NS | NS | NS | NS | NS | NS | NS | NS | NS | NS | NS | NS |
| KARNATAKA | BELGAUM | H | L | L | L | H | H | H | NS | NS | NS | NS | NS | NS |
| KARNATAKA | BAGALKOT | NS | L | NS | NS | NS | NS | NS | NS | NS | NS | H | H | NS |
| KARNATAKA | BIJAPUR | NS | NS | NS | NS | NS | NS | L | NS | L | H | NS | H | NS |
| KARNATAKA | BIDAR | NS | NS | L | L | NS | NS | NS | NS | NS | H | NS | NS | NS |
| KARNATAKA | RAICHUR | NS | NS | NS | NS | NS | NS | NS | NS | NS | NS | H | NS | NS |
| KARNATAKA | KOPPAL | H | NS | NS | H | H | NS | NS | NS | NS | NA | NS | NS | H |
| KARNATAKA | GADAG | NS | NS | NS | NS | NS | NS | NS | NS | NS | NS | NS | NS | NS |
| KARNATAKA | DHARWAD | H | L | NS | NS | NS | NS | L | NS | NS | H | NS | NS | NS |
| KARNATAKA | UTTARA KANNADA | H | NS | NS | NS | NS | NS | H | L | NS | NS | NS | H | H |
| KARNATAKA | HAVERI | NS | NS | NS | NS | NS | NS | NS | NS | NS | NS | NS | NS | H |
| KARNATAKA | BELLARY | NS | NS | L | NS | NS | NS | NS | NS | NS | NS | H | NS | NS |
| KARNATAKA | CHITRADURGA | H | NS | NS | NS | NS | NS | L | NS | NS | NS | NS | H | NS |
| KARNATAKA | DAVANAGERE | H | NS | NS | NS | H | NS | NS | NS | NS | NS | NS | NS | NS |
| KARNATAKA | SHIMOGA | H | NS | NS | NS | NS | NS | NS | NS | NS | NS | NS | NS | NS |
| KARNATAKA | UDUPI | NS | NS | NS | NS | NS | NS | NS | NS | NS | NS | NS | NS | NS |
| KARNATAKA | CHIKMAGALUR | NS | L | NS | NS | NS | NS | NS | NS | L | NS | NS | NS | H |
| KARNATAKA | TUMKUR | H | L | NS | NS | NS | H | L | NS | H | NS | L | H | NS |
| KARNATAKA | BANGALORE | H | L | H | L | L | L | H | H | L | NS | H | H | NS |
| KARNATAKA | MANDYA | H | L | NS | NS | NS | NS | NS | H | NS | NS | NS | H | NS |
| KARNATAKA | HASSAN | H | L | L | NS | NS | NS | NS | L | NS | NS | NS | H | NS |
| KARNATAKA | DAKSHINA KANNADA | H | NS | NS | NS | NS | NS | NS | NS | H | H | NS | NS | NS |
| KARNATAKA | KODAGU | NS | NS | NS | NS | NS | NS | NS | NS | NS | NS | NS | NS | NS |
| KARNATAKA | MYSORE | H | L | NS | L | NS | NS | NS | L | NS | H | H | NS | H |
| KARNATAKA | CHAMARAJANAGAR | H | NS | NS | NS | NS | NS | NS | NS | NS | NS | NS | NS | NS |
| KARNATAKA | GULBARGA | NS | NS | NS | NS | NS | NS | NS | H | NS | NS | H | H | NS |
| KARNATAKA | YADGIR | H | NS | NS | NS | NS | NS | NS | NS | NS | NS | NS | NS | NS |
| KARNATAKA | KOLAR | H | L | NS | NS | NS | NS | NS | H | NS | NS | NS | H | H |
| KARNATAKA | CHIKKABALLAPURA | H | L | NS | NS | NS | NS | NS | NS | NS | NS | NS | NS | NS |
| KARNATAKA | BANGALORE RURAL | H | NS | NS | NS | NS | NS | NS | H | NS | NS | NS | NS | NS |
| KARNATAKA | RAMANAGARA | NS | NS | NS | NS | NS | NS | NS | NS | NS | H | NS | NS | H |
| KERALA | KASARAGOD | NS | NS | NS | NS | NS | NS | NS | NS | NS | NS | NS | NS | NS |
| KERALA | KANNUR | H | L | NS | NS | NS | NS | L | NS | NS | H | NS | H | H |
| KERALA | WAYANAD | NS | NS | NS | NS | NS | NS | NS | NS | NS | NS | NS | NS | NS |
| KERALA | KOZHIKODE | H | NS | NS | NS | L | L | NS | NS | H | H | NS | H | NS |
| KERALA | MALAPPURAM | H | L | NA | L | NS | NS | H | NS | H | NS | H | NS | NS |
| KERALA | PALAKKAD | H | NS | NS | NS | NS | NS | NS | H | NS | H | H | NS | NS |
| KERALA | THRISSUR | NS | NS | NA | H | NS | NS | H | NS | NS | H | NS | H | NS |
| KERALA | ERNAKULAM | H | L | NS | NS | NS | NS | H | L | H | NS | H | H | NS |
| KERALA | IDUKKI | H | L | NS | L | NS | NS | NS | NS | NS | NS | NS | H | NS |
| KERALA | KOTTAYAM | NS | L | NS | NS | NS | NS | NS | NS | NS | NS | NS | NS | H |
| KERALA | ALAPPUZHA | H | NS | NA | NS | NS | NS | NS | NS | L | H | H | L | NS |
| KERALA | PATHANAMTHITTA | NS | NS | NA | NS | NS | NS | NS | NS | NS | NS | NS | NS | NS |
| KERALA | KOLLAM | H | L | NA | NS | NS | NS | L | NS | NS | NS | NS | H | NS |
| KERALA | THIRUVANANTHAPURAM | H | L | NS | L | NS | NS | NS | L | NS | H | NS | H | H |
| MADHYA PRADESH | SHEOPUR | NS | NS | NS | NS | NS | NS | NS | NS | NS | NS | H | NS | NS |
| MADHYA PRADESH | MORENA | NS | NS | NS | NS | NS | NS | NS | NS | NS | NS | NS | NS | NS |
| MADHYA PRADESH | BHIND | NS | NS | NS | NS | NS | NS | H | NS | NS | NS | NS | NS | NS |
| MADHYA PRADESH | GWALIOR | NS | NS | NS | NS | NS | H | NS | NS | NS | NS | NS | NS | NS |
| MADHYA PRADESH | DATIA | NS | NS | NS | NS | NS | NS | NS | NS | NS | NS | NS | NS | NS |
| MADHYA PRADESH | SHIVPURI | NS | NS | NS | NS | NA | NS | NS | NS | NS | NS | NS | H | NA |
| MADHYA PRADESH | TIKAMGARH | NS | NS | NS | NS | H | NA | NS | NA | NS | NS | NS | NS | NA |
| MADHYA PRADESH | CHHATARPUR | H | NS | NS | NS | NS | NS | NS | NS | NS | NS | NS | H | NS |
| MADHYA PRADESH | PANNA | NS | NS | NS | NS | NS | NS | NS | NS | NS | NS | NS | NS | NS |
| MADHYA PRADESH | SAGAR | NS | NS | NS | NS | NS | NS | NS | NS | NS | NS | H | NS | NS |
| MADHYA PRADESH | DAMOH | NS | NS | NS | NS | NS | NS | NS | NS | NS | NS | H | NS | NS |
| MADHYA PRADESH | SATNA | NS | NS | NS | NS | NS | NS | L | NS | NS | NS | H | NS | NS |
| MADHYA PRADESH | REWA | NS | NS | NS | NS | H | NS | NS | NS | NS | NS | NS | NS | NS |
| MADHYA PRADESH | UMARIA | NS | NS | NS | NS | NS | NS | NS | NS | NS | NS | NS | NS | NS |
| MADHYA PRADESH | NEEMUCH | H | NS | NS | NS | NS | NS | NS | NS | NS | NS | NS | NS | NS |
| MADHYA PRADESH | MANDSAUR | NS | L | NS | NS | NS | NS | NS | NS | NS | NS | H | NS | NS |
| MADHYA PRADESH | RATLAM | NS | NS | NS | NS | NS | NS | NS | NS | NS | NS | NS | NS | NS |
| MADHYA PRADESH | UJJAIN | H | NS | NS | NS | NS | NS | NS | NS | NS | NS | H | H | NS |
| MADHYA PRADESH | DEWAS | H | NS | NS | NS | NS | NS | NS | NS | NS | NS | NS | NS | NS |
| MADHYA PRADESH | DHAR | H | NS | H | NS | NS | NS | NS | NA | NS | NS | NS | NS | NS |
| MADHYA PRADESH | INDORE | H | NS | NS | NS | NS | NS | NS | NS | NS | NS | H | NS | NS |
| MADHYA PRADESH | KHARGONE (WEST NIMAR) | NS | NS | NS | NS | NS | NS | NA | NS | NS | NS | NS | NS | NS |
| MADHYA PRADESH | BARWANI | NS | NS | NS | NS | NS | NS | NS | NS | NS | NS | NS | NS | NA |
| MADHYA PRADESH | RAJGARH | NS | NS | NS | L | NS | NS | NS | NS | NS | NS | NS | NS | NA |
| MADHYA PRADESH | VIDISHA | NS | NS | NS | NS | NS | NS | NS | NS | NS | NS | NS | NS | NS |
| MADHYA PRADESH | BHOPAL | L | NA | NA | NA | NA | NS | NA | NA | NA | NA | H | H | NA |
| MADHYA PRADESH | SEHORE | NS | NS | NS | NS | NS | NS | NS | NS | NS | NS | NS | H | NA |
| MADHYA PRADESH | RAISEN | NS | NS | NS | NS | NS | NS | NS | NA | NS | NS | NS | H | NA |
| MADHYA PRADESH | BETUL | NS | NS | NS | L | NS | NS | NS | NS | NS | NS | NS | NS | NS |
| MADHYA PRADESH | HARDA | NS | NS | NS | NS | NS | NS | NS | NS | NS | NS | NS | NS | NS |
| MADHYA PRADESH | HOSHANGABAD | NS | NS | NS | NS | NS | NS | NS | NS | NS | NS | NS | NS | NS |
| MADHYA PRADESH | KATNI | NS | NS | NS | NS | NS | NS | NS | NS | NS | NS | NS | NS | NA |
| MADHYA PRADESH | JABALPUR | NS | NS | NA | NS | H | NS | NS | NA | NS | H | NS | H | NA |
| MADHYA PRADESH | NARSIMHAPUR | H | NS | NS | NS | NS | NS | NS | NS | NS | NS | NS | NS | NS |
| MADHYA PRADESH | DINDORI | NS | NS | NS | NS | NS | NS | NS | NS | NS | NS | NS | NS | NS |
| MADHYA PRADESH | MANDLA | H | NS | NS | NS | NS | NS | NS | NS | NS | NS | NS | NS | NS |
| MADHYA PRADESH | CHHINDWARA | NS | NS | L | NS | NS | NS | NS | NS | NS | NS | NS | NS | NS |
| MADHYA PRADESH | SEONI | NS | NS | NS | NS | NS | NS | NS | NS | NS | NS | NS | NS | NS |
| MADHYA PRADESH | BALAGHAT | NS | NS | NS | NS | NS | NS | NS | L | NS | NS | NS | NS | NA |
| MADHYA PRADESH | GUNA | H | NS | NS | NS | NS | NS | NS | NS | NS | NS | NS | NS | NS |
| MADHYA PRADESH | ASHOKNAGAR | NS | NS | NS | NS | NS | NS | NS | NS | NS | NS | NS | NS | NS |
| MADHYA PRADESH | SHAHDOL | NS | L | NS | NS | NS | NS | NS | NS | NS | NS | NS | NS | NS |
| MADHYA PRADESH | ANUPPUR | NS | NS | NS | NS | NS | NS | NS | NS | NS | NS | NS | NS | NS |
| MADHYA PRADESH | SIDHI | NS | NS | NS | NS | NS | NS | NS | NS | NS | NS | NS | NS | NS |
| MADHYA PRADESH | SINGRAULI | H | NS | NS | NS | NS | NS | NA | NS | NS | NS | NS | NS | NS |
| MADHYA PRADESH | JHABUA | NS | NS | NS | NS | NS | NS | NS | NS | NS | NS | NS | NS | NS |
| MADHYA PRADESH | ALIRAJPUR | NS | NS | NS | NS | NS | NS | NS | NS | NS | NS | NS | NS | NS |
| MADHYA PRADESH | KHANDWA (EAST NIMAR) | NA | NA | NA | NA | NA | NA | NA | NA | NA | NA | NA | NA | NA |
| MADHYA PRADESH | BURHANPUR | NS | NS | NS | NS | NS | NS | NS | NS | NS | NS | NS | NS | NS |
| MADHYA PRADESH | AGAR MALWA | NS | NS | NS | NS | NS | NS | NS | NA | NS | NS | NS | NS | NS |
| MADHYA PRADESH | SHAJAPUR | NS | NS | NS | NS | NS | NS | NS | NS | NS | NS | NS | NS | NA |
| MAHARASHTRA | NANDURBAR | H | NS | NS | NS | NS | NS | NS | NS | NS | NS | NS | H | NA |
| MAHARASHTRA | DHULE | NS | NS | NS | NS | NS | NS | NS | NS | NS | H | NS | NS | NS |
| MAHARASHTRA | JALGAON | NS | L | NS | NS | L | NS | NS | H | NS | L | NS | H | H |
| MAHARASHTRA | BULDANA | H | NS | NS | H | NS | NS | NS | H | NS | NS | H | NS | NS |
| MAHARASHTRA | AKOLA | NS | NS | NS | NS | NS | NS | L | H | NS | H | H | NS | NA |
| MAHARASHTRA | WASHIM | NS | NS | NS | NS | NS | NS | NS | NS | NS | NS | NS | NS | NS |
| MAHARASHTRA | AMRAVATI | H | NS | NS | NS | L | NS | NS | NS | H | NS | NS | H | NS |
| MAHARASHTRA | WARDHA | NS | NS | NS | NS | NS | NS | NS | L | NS | NS | NS | H | NS |
| MAHARASHTRA | NAGPUR | H | NS | NS | H | NS | NS | NS | NS | NS | NS | NS | NS | H |
| MAHARASHTRA | BHANDARA | NS | NS | NS | NS | NS | NS | NS | NS | NS | NS | H | NS | NS |
| MAHARASHTRA | GONDIYA | NS | NS | NS | NS | NS | NS | NA | NS | NS | NS | NS | NS | NS |
| MAHARASHTRA | GADCHIROLI | NS | NS | NS | NS | L | NS | NS | NS | NS | NS | H | NS | NS |
| MAHARASHTRA | CHANDRAPUR | H | NS | NS | NS | L | L | NS | NS | NS | NS | NS | H | H |
| MAHARASHTRA | YAVATMAL | H | NS | NS | H | NS | NS | NS | H | NS | H | NS | NS | H |
| MAHARASHTRA | NANDED | NS | NS | L | L | NS | H | NA | NS | H | L | NS | H | H |
| MAHARASHTRA | HINGOLI | NS | NS | NS | NS | NS | NS | NS | NS | NS | H | H | NS | NS |
| MAHARASHTRA | PARBHANI | NS | NS | NS | NS | NS | NS | NS | NS | NS | NS | NS | H | NS |
| MAHARASHTRA | JALNA | H | NS | NS | H | NS | NS | NS | H | L | H | H | NS | NS |
| MAHARASHTRA | AURANGABAD | NS | NS | NS | NS | H | H | L | L | H | H | H | NS | NA |
| MAHARASHTRA | NASHIK | NS | NS | L | H | NS | H | NS | NS | H | H | H | NS | NS |
| MAHARASHTRA | MUMBAI SUBURBAN | NA | L | H | NS | NA | NA | NA | NA | H | L | NS | H | H |
| MAHARASHTRA | MUMBAI | H | L | L | NS | NA | NA | NS | NA | NS | L | NS | NS | H |
| MAHARASHTRA | RAIGARH | H | NS | NS | NS | NS | NS | NS | H | NS | NS | NS | H | NS |
| MAHARASHTRA | PUNE | H | L | L | NS | H | NS | NS | H | H | H | L | H | H |
| MAHARASHTRA | AHMADNAGAR | H | L | NS | NS | NS | NS | NS | H | NS | H | H | NS | H |
| MAHARASHTRA | BID | NS | NS | NS | NS | NS | NS | H | NS | NS | H | NS | NS | NS |
| MAHARASHTRA | LATUR | NS | L | L | L | NS | NS | NS | NS | NS | NS | H | H | H |
| MAHARASHTRA | OSMANABAD | NS | NS | NS | NS | NS | NS | NS | L | H | NS | NS | H | NS |
| MAHARASHTRA | SOLAPUR | H | L | NS | NS | NS | NS | H | NS | NS | NS | NS | NS | NS |
| MAHARASHTRA | SATARA | H | NS | NS | NS | NS | NS | NS | H | NS | NS | NS | H | NS |
| MAHARASHTRA | RATNAGIRI | H | NS | NS | H | NS | NS | NS | H | L | NS | H | NS | NS |
| MAHARASHTRA | SINDHUDURG | H | NS | NS | NS | NS | NS | NS | NS | NS | NS | NS | NS | NS |
| MAHARASHTRA | KOLHAPUR | H | L | NS | NS | H | NS | NS | NS | NS | H | NS | H | L |
| MAHARASHTRA | SANGLI | H | NS | NA | NS | NS | NS | NS | NS | L | NS | NS | NS | H |
| MAHARASHTRA | PALGHAR | H | L | L | NS | NS | L | NS | NS | NS | NS | H | NS | NS |
| MAHARASHTRA | THANE | H | L | L | NS | L | H | H | L | NS | H | NS | H | NA |
| MANIPUR | SENAPATI | NS | NS | NS | NS | NS | NS | NS | NS | NS | NS | NS | NS | NS |
| MANIPUR | TAMENGLONG | NS | NS | NS | NS | NS | NS | NS | NS | NS | NS | NS | NS | NS |
| MANIPUR | CHURACHANDPUR | NS | NS | NS | NS | NS | NS | NS | NS | NS | NS | NS | NS | NS |
| MANIPUR | BISHNUPUR | NS | NS | NS | NS | NS | NS | NS | NS | NS | NS | NS | NS | NS |
| MANIPUR | THOUBAL | NS | NS | NS | NS | NS | NS | NS | NS | NS | NS | NS | NS | NS |
| MANIPUR | IMPHAL WEST | H | NS | NS | NS | NS | NS | NS | NS | NS | NS | NS | NS | NS |
| MANIPUR | IMPHAL EAST | NS | NS | NS | NS | NS | H | NS | NS | NS | NS | NS | NS | NS |
| MANIPUR | UKHRUL | NS | NS | NS | NS | NS | NS | NS | NS | NS | NS | NS | NS | NA |
| MANIPUR | CHANDEL | NS | NS | NS | NS | NS | NS | NS | NS | NS | NS | NS | NS | NS |
| MEGHALAYA | SOUTH GARO HILLS | NS | NS | NS | NS | NS | NS | NS | NS | NS | NS | NS | NS | NA |
| MEGHALAYA | RIBHOI | NS | NS | NS | NS | NS | NS | NS | NS | NS | NS | NS | NS | NS |
| MEGHALAYA | EAST KHASI HILLS | NS | NS | NS | NS | NS | NS | NS | NS | NS | NS | NS | NS | NS |
| MEGHALAYA | EAST GARO HILLS | NS | NS | NS | NS | NS | NS | NS | NS | NS | NS | NS | NS | NS |
| MEGHALAYA | EAST JANTIA HILLS | NS | NS | NS | NS | NS | NS | NS | NA | NS | NS | NS | NS | NA |
| MEGHALAYA | NORTH GARO HILLS | NS | NS | NS | NS | NS | NS | NS | NS | NS | NS | NS | NS | NS |
| MEGHALAYA | SOUTH WEST GARO HILLS | NS | NS | NS | NS | NS | NS | NS | NA | NS | NS | NS | NS | NS |
| MEGHALAYA | SOUTH WEST KHASI HILLS | NS | NS | NS | NS | NS | NA | NS | NA | NS | NS | NS | NS | NS |
| MEGHALAYA | WEST GARO HILLS | NS | NS | NS | NS | NS | NS | NS | H | NS | NS | NS | NS | NS |
| MEGHALAYA | WEST JAINTIA HILLS | NS | NS | NS | NS | NS | NS | NS | NS | NS | NS | NS | NS | NS |
| MEGHALAYA | WEST KHASI HILLS | NS | NS | NS | NS | NS | NS | NS | NS | NS | NS | NS | NS | NS |
| NAGALAND | MON | NS | NS | NS | NS | NS | NS | NS | NS | NS | NS | NS | NS | NS |
| NAGALAND | MOKOKCHUNG | NS | NS | NS | NS | NS | NS | NS | NS | NS | NS | NS | NS | NS |
| NAGALAND | ZUNHEBOTO | NS | NS | NS | NS | NS | NS | NS | NS | NS | NS | NS | NS | NS |
| NAGALAND | WOKHA | NS | NS | NS | NS | NS | NS | NS | NS | NS | NS | NS | NS | NA |
| NAGALAND | DIMAPUR | NS | NS | NS | NS | NS | NS | NS | NS | NS | NS | NS | NS | NS |
| NAGALAND | PHEK | NS | NS | NS | NS | NS | NS | NS | NS | NS | NS | NS | NS | NA |
| NAGALAND | TUENSANG | NS | NS | NS | NS | NS | NS | NS | NS | NS | NS | NS | NS | NA |
| NAGALAND | LONGLENG | NS | NS | NS | NS | NS | NS | NS | NS | NS | NS | NS | NS | NS |
| NAGALAND | KIPHIRE | NS | NS | NS | NS | NS | NS | NS | NS | NS | NS | NS | NS | NA |
| NAGALAND | KOHIMA | NS | NS | NS | NS | NS | NS | NS | NS | NS | NS | NS | NS | NS |
| NAGALAND | PEREN | NS | NS | NS | NS | NS | NS | NS | NS | NS | NS | NS | NS | NS |
| NCT OF DELHI | CENTRAL | H | NS | NS | NS | NS | NS | NS | NA | NS | NS | NS | NS | NS |
| NCT OF DELHI | EAST | H | L | NS | NS | NS | NS | NS | NA | NS | NS | NS | H | NS |
| NCT OF DELHI | NEW DELHI | H | L | NS | NS | NS | NS | NS | NA | NS | NS | NS | NS | NS |
| NCT OF DELHI | NORTH | H | NS | H | NS | NS | NS | NS | NS | NS | NS | NS | H | NS |
| NCT OF DELHI | NORTH EAST | NS | NS | NS | NS | NS | NS | NS | NA | NS | NS | NS | NS | NS |
| NCT OF DELHI | NORTH WEST | NS | L | NS | NS | NS | NS | NS | NA | NS | NS | NS | H | NS |
| NCT OF DELHI | SHAHDARA | NS | NS | NS | NS | NS | NS | NS | NA | NS | NS | NS | NS | NS |
| NCT OF DELHI | SOUTH | NS | NS | NS | NS | NS | NA | NS | NA | NS | NS | NS | NS | NS |
| NCT OF DELHI | SOUTH EAST | NS | NS | NS | H | NS | NS | NS | NA | NS | NS | NS | NS | NS |
| NCT OF DELHI | SOUTH WEST | NS | NS | NS | NS | NS | NS | NS | NS | NS | NS | NS | NS | NS |
| NCT OF DELHI | WEST | H | L | NS | NS | NS | NS | L | NA | NS | NS | NS | NS | NS |
| ODISHA | BARGARH | NS | NS | NS | NS | NS | H | NS | NS | NS | NS | NS | NS | NS |
| ODISHA | JHARSUGUDA | NS | NS | NS | NS | NS | NS | NS | NS | NS | NS | NS | NS | NS |
| ODISHA | SAMBALPUR | NS | NS | NS | NS | NS | NS | NS | NS | NS | NS | NS | NS | NS |
| ODISHA | DEBAGARH | NS | NS | NS | NS | NS | NS | NS | NS | NS | NS | NS | NS | NS |
| ODISHA | SUNDARGARH | NS | NS | NS | NS | NS | NS | NS | H | NS | NS | NS | NS | NS |
| ODISHA | KENDUJHAR | H | NS | NS | NS | NS | NS | NS | NS | NS | NS | H | NS | NS |
| ODISHA | MAYURBHANJ | H | NS | NS | NS | NS | NS | L | NS | NS | H | NS | NS | NS |
| ODISHA | BALESHWAR | NS | NS | NS | NS | NS | NS | NS | NS | NS | NS | NS | NS | NS |
| ODISHA | BHADRAK | NS | NS | NS | NS | NS | NS | NS | NS | L | NS | NS | NS | NS |
| ODISHA | KENDRAPARA | NS | NS | NS | NS | NS | NS | NS | NS | NS | NS | NS | NS | NS |
| ODISHA | JAGATSINGHAPUR | NS | NS | NS | NS | NS | NS | NS | NS | NS | NS | NS | NS | NS |
| ODISHA | CUTTACK | H | NS | NS | NS | NS | NS | NS | NS | NS | NS | H | NS | NS |
| ODISHA | JAJAPUR | NS | NS | NS | NS | NS | NS | H | H | NS | NS | H | NS | NS |
| ODISHA | DHENKANAL | NS | NS | NS | NS | NS | NS | NS | NS | NS | NS | NS | NS | NS |
| ODISHA | ANUGUL | NS | NS | NS | NS | NS | NS | NS | NS | NS | NS | NS | NS | NS |
| ODISHA | NAYAGARH | H | NS | NS | NS | NS | NS | NS | NS | NS | NS | NS | NS | NS |
| ODISHA | KHORDHA | H | L | NS | NS | NS | NS | NS | NS | NS | NS | NS | NS | NS |
| ODISHA | PURI | NS | L | NS | NS | H | NS | NS | NS | NS | NS | NS | H | NS |
| ODISHA | GANJAM | H | NS | NS | NS | NS | NS | NS | NS | NS | NS | NS | NS | H |
| ODISHA | GAJAPATI | NS | NS | NS | NS | NS | NS | NS | NS | NS | NS | NS | NS | NS |
| ODISHA | KANDHAMAL | H | NS | NS | NS | NS | NS | NS | NS | NS | NS | NS | NS | NS |
| ODISHA | BAUDH | NS | NS | NS | NS | NS | NS | NS | NS | NS | NS | NS | NS | NS |
| ODISHA | SUBARNAPUR | NS | NS | NS | NS | NS | NS | NS | NS | NS | NS | NS | NS | NS |
| ODISHA | BALANGIR | H | NS | NS | NS | NS | NS | NS | NS | NS | NS | H | NS | NS |
| ODISHA | NUAPADA | NS | NS | NS | NS | NS | NS | NS | NS | NS | NS | NS | NS | NS |
| ODISHA | KALAHANDI | NS | NS | NS | NS | NS | NS | NS | NS | NS | NS | NS | NS | H |
| ODISHA | RAYAGADA | NS | NS | NS | NS | NS | NS | NS | NS | NS | NS | NS | NS | NS |
| ODISHA | NABARANGAPUR | H | NS | NS | NS | NS | NS | NS | NS | NS | NS | NS | NS | NA |
| ODISHA | KORAPUT | H | NS | NS | NS | NS | NS | NS | NS | NS | NS | NS | NS | NS |
| ODISHA | MALKANGIRI | NS | NS | NS | NS | NS | NS | NS | NS | NS | NS | NS | NS | NS |
| PUNJAB | KAPURTHALA | NS | NS | NS | NS | NS | NS | NS | NS | NS | NS | NS | NS | NS |
| PUNJAB | JALANDHAR | H | L | NS | NS | NS | NS | NS | H | NS | NS | NS | NS | NS |
| PUNJAB | HOSHIARPUR | H | NS | NS | NS | NS | NS | NS | NS | NS | NS | NS | NS | NS |
| PUNJAB | SHAHID BHAGAT SINGH NAGAR | NS | NS | NS | NS | NS | NS | NS | NS | NS | NS | NS | NS | NS |
| PUNJAB | FATEHGARH SAHIB | NS | NS | NS | NS | NS | NS | NS | NS | NS | NS | NS | NS | NS |
| PUNJAB | LUDHIANA | H | NS | NS | NS | NS | NS | NS | L | NS | NS | H | H | NS |
| PUNJAB | MOGA | NS | NS | NS | NS | NS | NS | NS | NS | NS | NS | NS | NS | NS |
| PUNJAB | MUKTSAR | H | NS | NS | NS | NS | NS | NS | NS | NS | NS | NS | NS | NS |
| PUNJAB | FARIDKOT | NS | NS | NS | NS | NS | NS | NS | NS | NS | NS | NS | NS | NS |
| PUNJAB | BATHINDA | H | NS | NS | NS | NS | NS | NS | NS | NS | NS | NS | NS | NS |
| PUNJAB | MANSA | NS | NS | NS | NS | NS | NS | NS | NS | NS | NS | NS | NS | NS |
| PUNJAB | PATIALA | NS | L | NS | L | NS | NS | NS | NS | NS | NS | NS | NS | NS |
| PUNJAB | AMRITSAR | H | NS | L | NS | NS | NS | NS | NS | NS | NS | NS | NS | NS |
| PUNJAB | TARN TARAN | H | NS | NS | NS | NS | NS | NS | L | NS | NS | NS | NS | NS |
| PUNJAB | RUPNAGAR | NS | NS | NS | NS | NS | NS | NS | NS | NS | NS | NS | NS | NS |
| PUNJAB | SAHIBZADA AJIT SINGH NAGAR | NS | NS | NS | NS | NS | NS | NS | L | NS | NS | NS | NS | NS |
| PUNJAB | SANGRUR | NS | NS | L | NS | NS | NS | NS | NS | NS | NS | NS | NS | NS |
| PUNJAB | BARNALA | NS | NS | NS | NS | NS | NS | NS | NS | NS | NS | NS | NS | NS |
| PUNJAB | FAZILKA | NS | NS | NS | NS | NS | NS | NS | NS | NS | NS | NS | NS | NS |
| PUNJAB | FIROZPUR | NS | NS | NS | NS | NS | NS | NS | NS | NS | NS | NS | NS | NS |
| PUNJAB | GURDASPUR | H | NS | NS | NS | NS | NS | NS | NS | NS | NS | H | NS | NS |
| PUNJAB | PATHANKOT | NS | NS | NS | NS | NS | NS | NS | NS | NS | NS | NS | NS | NS |
| RAJASTHAN | GANGANAGAR | H | H | NS | NS | NS | NS | NS | NS | NS | NS | H | NS | NS |
| RAJASTHAN | HANUMANGARH | H | NS | NS | NS | NS | NS | NS | NS | NS | NS | NS | NS | NS |
| RAJASTHAN | BIKANER | H | NS | NS | NS | NS | NS | NS | H | NS | NS | NS | NS | NA |
| RAJASTHAN | CHURU | NS | NS | NS | NS | NS | NS | NS | NS | NS | NS | NS | NS | NS |
| RAJASTHAN | JHUNJHUNUN | NS | NS | NS | NS | NS | NS | NS | NS | NS | NS | NS | L | NS |
| RAJASTHAN | ALWAR | H | NS | NS | NS | NS | NS | NS | L | NS | NS | NS | NS | NS |
| RAJASTHAN | BHARATPUR | NS | NS | NS | NS | NS | NS | NS | NS | NS | NS | H | NS | NS |
| RAJASTHAN | DHAULPUR | NS | NS | NS | NS | NS | NS | NS | NS | NS | NS | NS | NS | NS |
| RAJASTHAN | KARAULI | NS | NS | NS | NS | NS | NS | NS | NS | NS | NS | NS | NS | NS |
| RAJASTHAN | SAWAI MADHOPUR | NS | NS | NS | NS | NS | NS | NS | NS | NS | NS | NS | NS | NA |
| RAJASTHAN | DAUSA | H | NS | NS | NS | NS | NS | NS | NS | NS | NS | NS | NS | NS |
| RAJASTHAN | JAIPUR | NS | NS | NS | NS | NS | NS | H | H | NS | NS | NS | NS | NS |
| RAJASTHAN | SIKAR | H | NS | NS | NS | NS | NS | NS | NS | NS | NS | NS | NS | NS |
| RAJASTHAN | NAGAUR | NS | NS | NS | NS | NS | NS | NS | NS | NS | NS | H | NS | NS |
| RAJASTHAN | JODHPUR | H | NS | NS | H | H | H | NS | NS | NS | NS | H | H | NS |
| RAJASTHAN | JAISALMER | NS | NS | NS | NS | NS | NS | NS | NS | NS | NS | NS | NS | NS |
| RAJASTHAN | BARMER | NS | NS | NS | NS | NS | NS | NS | NS | NS | NS | NS | NS | NS |
| RAJASTHAN | JALOR | NS | NS | NS | NS | NS | NS | NS | NS | NS | NS | NS | NS | NA |
| RAJASTHAN | SIROHI | NS | L | NS | NS | NS | NS | NS | NS | NS | NS | NS | NS | NS |
| RAJASTHAN | PALI | NS | NS | NS | NS | NS | NS | NS | NS | NS | NS | NS | NS | NA |
| RAJASTHAN | AJMER | NS | L | NS | L | NS | NS | H | NS | NS | NS | NS | NS | NS |
| RAJASTHAN | TONK | H | NS | H | NS | NS | NS | NS | NS | NS | NS | NS | H | NS |
| RAJASTHAN | BUNDI | NS | NS | NS | NS | NS | NS | NS | NS | NS | NS | NS | NS | NS |
| RAJASTHAN | BHILWARA | NS | NS | NS | NS | NS | NS | NS | NS | NS | NS | NS | NS | NS |
| RAJASTHAN | RAJSAMAND | NS | NS | NS | NS | NS | NS | NS | NS | NS | NS | NS | NS | NS |
| RAJASTHAN | DUNGARPUR | NS | NS | NS | NS | NS | NS | NS | NA | NS | NS | NS | NS | NA |
| RAJASTHAN | BANSWARA | H | NS | NS | NS | NS | NS | NS | NA | NS | NS | H | NS | NS |
| RAJASTHAN | CHITTAURGARH | NS | NS | NS | NS | NS | NS | NS | NS | NS | NS | NS | NS | NS |
| RAJASTHAN | KOTA | NS | NS | NS | NS | NS | NS | NS | NS | NS | NS | H | NS | NS |
| RAJASTHAN | BARAN | NS | NS | NS | NS | NS | NS | NS | NS | NS | NS | NS | NS | NA |
| RAJASTHAN | JHALAWAR | NS | NS | NS | NS | NS | NS | NS | NS | NS | NS | NS | NS | NS |
| RAJASTHAN | UDAIPUR | NS | NS | NS | NS | NS | NS | NS | H | NS | NS | NS | NS | NS |
| RAJASTHAN | PRATAPGARH | NS | NS | NS | NS | NS | NS | NS | NS | NS | NS | NS | NS | NA |
| TAMIL NADU | THIRUVALLUR | H | NS | NS | NS | NS | NS | NS | NS | NS | H | H | L | NS |
| TAMIL NADU | CHENNAI | H | L | NS | L | NS | L | NS | NA | NS | NS | H | L | H |
| TAMIL NADU | KANCHEEPURAM | NS | NS | L | NS | NS | NS | NS | NS | NS | NS | H | NS | NS |
| TAMIL NADU | VELLORE | NS | L | L | NS | L | L | L | L | NS | NS | H | NS | NS |
| TAMIL NADU | TIRUVANNAMALAI | NS | L | NS | NS | NS | NS | NS | NS | NS | NS | NS | NS | NS |
| TAMIL NADU | VILUPPURAM | H | NS | NS | NS | NS | NS | L | L | NS | H | NS | NS | NS |
| TAMIL NADU | SALEM | H | L | L | NS | NS | NS | L | NS | NS | NS | NS | H | NS |
| TAMIL NADU | NAMAKKAL | NS | L | NS | NS | NS | NS | NS | NS | NS | NS | NS | NS | H |
| TAMIL NADU | ERODE | H | L | NS | L | NS | NS | H | NS | NS | NS | L | NS | H |
| TAMIL NADU | THE NILGIRIS | NS | NS | NS | NS | NS | NS | NS | NS | NS | NS | NS | NS | NS |
| TAMIL NADU | DINDIGUL | NS | L | NS | NS | NS | NS | NS | NS | NS | NS | NS | NS | H |
| TAMIL NADU | KARUR | NS | NS | NS | NS | NS | NS | NS | NS | NS | NS | NS | NS | NS |
| TAMIL NADU | TIRUCHIRAPPALLI | NS | L | NS | NS | NS | H | NS | NS | NS | NS | NS | NS | NS |
| TAMIL NADU | PERAMBALUR | NS | NS | NS | NS | NS | NS | NS | NS | NS | NS | NS | NS | NS |
| TAMIL NADU | ARIYALUR | NS | NS | NS | NS | NS | NS | NS | NS | NS | NS | NS | NS | NS |
| TAMIL NADU | CUDDALORE | NS | NS | NS | NS | NS | NS | NS | L | NS | NS | NS | H | NS |
| TAMIL NADU | NAGAPATTINAM | NS | NS | NS | NS | L | NS | NS | NS | H | NS | H | NS | NS |
| TAMIL NADU | THIRUVARUR | NS | L | L | NS | NS | NS | NS | NS | NS | NS | NS | NS | NS |
| TAMIL NADU | THANJAVUR | NS | NS | L | NS | NS | NS | NS | NS | NS | NS | NS | NS | NS |
| TAMIL NADU | PUDUKKOTTAI | H | NS | NS | NS | NS | NS | NS | NS | NS | NS | NS | NS | H |
| TAMIL NADU | SIVAGANGA | NS | NS | NS | NS | NS | NS | NS | NS | NS | NS | NS | NS | NS |
| TAMIL NADU | MADURAI | NS | NS | NS | NS | NS | NS | H | NS | NS | NS | NS | H | NS |
| TAMIL NADU | THENI | H | L | NS | NS | NS | NS | NS | NS | NS | NS | NS | NS | H |
| TAMIL NADU | VIRUDHUNAGAR | NS | NS | L | NS | NS | NS | NS | NS | NS | NS | NS | NS | NS |
| TAMIL NADU | RAMANATHAPURAM | H | NS | NS | NS | NS | NS | NS | L | NS | NS | NS | NS | NS |
| TAMIL NADU | THOOTHUKKUDI | H | NS | NS | NS | NS | NS | NS | NS | NS | NS | H | NS | NS |
| TAMIL NADU | TIRUNELVELI | H | NS | NS | NS | NS | H | NS | L | NS | L | NS | NS | H |
| TAMIL NADU | KANNIYAKUMARI | NS | NS | NS | NS | NS | NS | NS | NS | NS | H | NS | L | NS |
| TAMIL NADU | DHARMAPURI | NS | NS | NS | NS | NS | NS | NS | NS | NS | NS | NS | NS | NS |
| TAMIL NADU | KRISHNAGIRI | NS | L | NS | L | NS | NS | NS | NS | NS | NS | NS | NS | NS |
| TAMIL NADU | COIMBATORE | NS | L | NS | NS | L | L | NS | H | NS | NS | NS | H | H |
| TAMIL NADU | TIRUPPUR | H | NS | NS | NS | NS | NS | NS | NS | NS | H | NS | NS | NS |
| TELANGANA | ADILABAD | NS | NS | NS | NS | NS | NS | NS | NS | L | NS | NS | NS | NS |
| TELANGANA | BHADRADRI KOTHAGUDEM | H | NS | NS | NS | NS | NS | NS | NS | NS | NS | NS | NS | H |
| TELANGANA | HYDERABAD | H | L | NS | NS | L | NA | NS | NA | H | NS | NS | H | NS |
| TELANGANA | JAGITIAL | H | NS | NS | NS | NS | NS | NS | NS | NS | NS | NS | H | NS |
| TELANGANA | JANGOAN | NS | NS | NS | NS | NS | NS | NS | NS | NS | NS | NS | NS | NS |
| TELANGANA | JAYASHANKAR BHUPALAPALLY | NS | NS | L | NS | NS | NS | NS | NS | NS | NS | H | NS | NS |
| TELANGANA | JOGULAMBA GADWAL | NS | NS | NS | NS | NS | NS | NS | NS | NS | NS | NS | NS | NS |
| TELANGANA | KAMAREDDY | NS | NS | NS | NS | NS | NS | NS | NS | NS | NS | NS | NS | NS |
| TELANGANA | KARIMNAGAR | H | NS | NS | NS | NS | NS | NS | NS | NS | H | NS | H | NS |
| TELANGANA | KHAMMAM | H | L | NS | L | NS | NS | NS | NS | NS | NS | H | NS | NS |
| TELANGANA | KOMARAM BHEEM ASIFABAD | NS | NS | NS | NS | NS | NS | NS | NS | NS | NS | NS | NS | NS |
| TELANGANA | MAHABUBABAD | NS | NS | L | NS | NS | NS | NS | NS | NS | NS | NS | NS | NS |
| TELANGANA | MAHABUBNAGAR | NS | NS | NS | NS | NS | NS | NS | NS | NS | NS | NS | H | NS |
| TELANGANA | MANCHERIAL | NS | NS | NS | L | NS | NS | NS | H | NS | NS | NS | NS | H |
| TELANGANA | MEDAK | H | NS | NS | NS | NS | NS | NS | NS | NS | NS | NS | NS | H |
| TELANGANA | MEDCHAL-MALKAJGIRI | H | NS | NS | NS | NS | NS | NS | H | NS | NS | NS | NS | H |
| TELANGANA | NAGARKURNOOL | H | NS | NS | L | NS | NS | NS | NS | NS | NS | H | NS | NS |
| TELANGANA | NALGONDA | H | NS | NS | NS | NS | NS | NS | H | NS | H | NS | NS | NS |
| TELANGANA | NIRMAL | NS | NS | NS | NS | NS | NS | NS | NS | NS | NS | NS | NS | NS |
| TELANGANA | NIZAMABAD | H | NS | H | NS | NS | NS | NS | NS | NS | NS | NS | NS | NS |
| TELANGANA | PEDDAPALLI | H | NS | NS | NS | NS | NS | NS | NS | H | NS | NS | NS | NS |
| TELANGANA | RAJANNA SIRCILLA | NS | NS | NS | NS | L | L | NS | NS | NS | NS | NS | NS | NS |
| TELANGANA | RANGA REDDY | H | L | L | NS | NS | NS | NS | NS | NS | NS | NS | H | NS |
| TELANGANA | SANGAREDDY | NS | L | NS | NS | NS | NS | NS | NS | NS | NS | H | NS | NS |
| TELANGANA | SIDDIPET | NS | NS | NS | NS | NS | NS | NS | NS | H | NS | NS | NS | NS |
| TELANGANA | SURYAPET | H | NS | NS | NS | NS | NS | NS | NS | NS | NS | H | NS | NS |
| TELANGANA | VIKARABAD | NS | NS | NS | NS | NS | NS | NS | NS | NS | NS | H | NS | NS |
| TELANGANA | WANAPARTHY | NS | NS | NS | NS | NS | NS | NS | NS | NS | NS | NS | NS | NS |
| TELANGANA | WARANGAL RURAL | NS | NS | NS | NS | NS | NS | NS | NS | NS | NS | H | NS | NS |
| TELANGANA | WARANGAL URBAN | H | NS | NS | NS | NS | NS | NS | NS | H | NS | NS | NS | NS |
| TELANGANA | YADADRI BHUVANAGIRI | NS | L | NS | NS | NS | NS | NS | NS | NS | NS | NS | NS | NS |
| TRIPURA | DHALAI | NS | NS | NS | NS | NS | NS | NS | NS | NS | NS | NS | NS | NS |
| TRIPURA | GOMATI | NS | NS | NS | NS | NS | NS | NS | NS | NS | NS | NS | NS | NS |
| TRIPURA | KHOWAI | H | NS | NS | NS | NS | NS | NS | NS | L | NS | NS | NS | NS |
| TRIPURA | NORTH TRIPURA | NS | NS | NS | NS | NS | NS | NS | NS | NS | NS | NS | NS | NS |
| TRIPURA | SEPAHIJALA | NS | NS | NS | NS | NS | NS | NS | NS | NS | NS | NS | NS | NS |
| TRIPURA | SOUTH TRIPURA | NS | NS | NS | NS | NS | NS | NS | NS | NS | NS | NS | NS | NS |
| TRIPURA | UNAKOTI | NS | NS | NS | NS | NS | NS | NS | NS | NS | NS | NS | NS | NS |
| TRIPURA | WEST TRIPURA | NS | NS | NS | NS | NS | NS | NS | NS | NS | NS | NS | NS | NS |
| UTTAR PRADESH | SAHARANPUR | H | NS | NS | NS | NS | NS | NS | NS | NS | NS | NS | H | NA |
| UTTAR PRADESH | BIJNOR | H | NS | NS | H | H | NS | NS | NS | NS | NS | NS | H | H |
| UTTAR PRADESH | RAMPUR | H | NS | NS | NS | NS | NS | NS | NS | NS | NS | NS | NS | NS |
| UTTAR PRADESH | JYOTIBA PHULE NAGAR | NS | NS | NS | NS | NS | NS | NS | NS | NS | NS | H | NS | NS |
| UTTAR PRADESH | MEERUT | NS | NS | NS | NS | NS | NS | H | NS | NS | H | NS | H | H |
| UTTAR PRADESH | BAGHPAT | NS | NS | NS | NS | NS | NS | NS | NS | NS | NS | NS | NS | NS |
| UTTAR PRADESH | GAUTAM BUDDHA NAGAR | H | NS | NS | NS | NS | NS | NS | NS | NS | NS | NS | NS | NS |
| UTTAR PRADESH | BULANDSHAHR | H | NS | NS | NS | NS | NS | NS | NS | NS | H | NS | NS | NS |
| UTTAR PRADESH | ALIGARH | H | NS | L | NS | NS | NS | NS | NS | NS | NS | NS | H | NS |
| UTTAR PRADESH | MAHAMAYA NAGAR | H | NS | NS | NS | NS | NS | NS | NS | NS | NS | NS | NS | NS |
| UTTAR PRADESH | MATHURA | H | L | NS | NS | NS | H | NS | NS | NS | NS | NS | NS | NS |
| UTTAR PRADESH | AGRA | NS | L | NS | NS | NS | NS | NS | NS | NS | NS | NS | H | NS |
| UTTAR PRADESH | FIROZABAD | NS | NS | NS | NS | NS | NS | NS | NS | NS | NS | NS | NS | NS |
| UTTAR PRADESH | MAINPURI | NS | NS | NS | NS | NS | NS | NS | NS | NS | NS | NS | NS | NS |
| UTTAR PRADESH | BAREILLY | NS | NS | NS | NS | NS | NS | NS | NS | NS | NS | NS | H | H |
| UTTAR PRADESH | PILIBHIT | NS | NS | NS | NS | NS | NS | NS | NS | NS | NS | NS | NS | NS |
| UTTAR PRADESH | SHAHJAHANPUR | H | NS | NS | NS | NS | H | NS | NS | NS | NS | NS | NS | NS |
| UTTAR PRADESH | KHERI | NS | NS | NS | NS | H | NS | H | NS | NS | NS | H | NS | NS |
| UTTAR PRADESH | SITAPUR | H | NS | NS | NS | NS | NS | NS | NS | NS | NS | H | NS | NS |
| UTTAR PRADESH | HARDOI | H | L | L | NS | NS | H | NS | NS | NS | NS | NS | NS | NS |
| UTTAR PRADESH | UNNAO | NS | NS | NS | NS | NS | NS | NS | NS | NS | NS | NS | NS | NS |
| UTTAR PRADESH | LUCKNOW | NS | L | NS | L | NS | NS | H | NS | NS | NS | NS | H | H |
| UTTAR PRADESH | FARRUKHABAD | NS | NS | NS | NS | NS | NS | NS | NS | NS | NS | NS | NS | NS |
| UTTAR PRADESH | KANNAUJ | NS | NS | NS | NS | NS | NS | NS | NS | NS | NS | H | NS | NA |
| UTTAR PRADESH | ETAWAH | NS | NS | NS | NS | NS | NS | NS | NS | NS | NS | NS | NS | NA |
| UTTAR PRADESH | AURAIYA | H | NS | NS | NS | NS | NS | NS | NS | NS | NS | NS | NS | NA |
| UTTAR PRADESH | KANPUR DEHAT | NS | NS | NS | NS | NS | NS | NS | H | NS | NS | NS | H | NS |
| UTTAR PRADESH | KANPUR NAGAR | NS | NS | NS | NS | NS | NS | NS | NS | NS | NS | NS | NS | NS |
| UTTAR PRADESH | JALAUN | NS | NS | H | NS | NS | NS | NS | NS | NS | NS | NS | H | NS |
| UTTAR PRADESH | JHANSI | H | NS | NS | NS | NS | NS | NS | NS | NS | NS | NS | NS | NS |
| UTTAR PRADESH | LALITPUR | NS | NS | NS | NS | NS | NS | NS | NS | NS | NS | NS | NS | NS |
| UTTAR PRADESH | HAMIRPUR | NS | NS | NS | NS | NS | NS | NS | NS | NS | NS | NS | NS | NS |
| UTTAR PRADESH | MAHOBA | NS | NS | NS | NS | NS | NS | NS | NS | NS | NS | NS | NS | NA |
| UTTAR PRADESH | BANDA | H | NS | NS | NS | NS | NS | NS | NS | NS | NS | H | NS | NS |
| UTTAR PRADESH | CHITRAKOOT | NS | NS | NS | NS | NS | NS | NS | NS | NS | NS | NS | NS | NS |
| UTTAR PRADESH | FATEHPUR | NS | NS | NS | NS | NS | NS | NS | NS | H | NS | NS | NS | NS |
| UTTAR PRADESH | PRATAPGARH | H | NS | NS | NS | NS | NS | NS | NS | NS | NS | H | NS | NS |
| UTTAR PRADESH | KAUSHAMBI | NS | NS | NS | NS | NS | NS | NS | NS | NS | NS | NS | NS | NS |
| UTTAR PRADESH | ALLAHABAD | NS | L | NS | NS | L | L | NS | H | NS | NS | NS | H | NS |
| UTTAR PRADESH | BARA BANKI | NS | NS | NS | L | NS | NS | NS | NS | NS | NS | NS | H | NS |
| UTTAR PRADESH | FAIZABAD | H | NS | NS | NS | NS | NS | NS | H | NS | NS | NS | NS | H |
| UTTAR PRADESH | AMBEDKAR NAGAR | NS | L | NS | NS | NS | NS | NS | NS | NS | NS | NS | NS | NS |
| UTTAR PRADESH | BAHRAICH | NS | NS | NS | NS | NS | NS | NS | NS | NS | NS | H | NS | NS |
| UTTAR PRADESH | SHRAWASTI | NS | NS | NS | NS | NS | NS | NS | NS | NS | NS | NS | H | NS |
| UTTAR PRADESH | BALRAMPUR | NS | NS | NS | NS | NS | NS | NS | NS | NS | NS | H | NS | NS |
| UTTAR PRADESH | GONDA | H | NS | NS | NS | NS | NS | NS | NS | NS | NS | NS | H | NS |
| UTTAR PRADESH | SIDDHARTHNAGAR | H | NS | NS | NS | NS | NS | NS | NS | NS | NS | NS | NS | NS |
| UTTAR PRADESH | BASTI | NS | NS | NS | NS | NS | NS | NS | NS | NS | NS | NS | H | NS |
| UTTAR PRADESH | SANT KABIR NAGAR | NS | NS | NS | NS | NS | NS | NS | H | NS | NS | NS | NS | NS |
| UTTAR PRADESH | MAHRAJGANJ | NS | NS | NS | NS | NS | NS | NS | NS | NS | NS | NS | NS | NS |
| UTTAR PRADESH | GORAKHPUR | NS | NS | NS | NS | NS | NS | NS | H | NS | NS | L | NS | NS |
| UTTAR PRADESH | KUSHINAGAR | NS | NS | NS | NS | H | H | H | NS | NS | NS | H | NS | NS |
| UTTAR PRADESH | DEORIA | NS | NS | NS | NS | NS | NS | H | H | NS | NS | NS | NS | NA |
| UTTAR PRADESH | AZAMGARH | NS | NS | NS | NS | NS | NS | H | NS | NS | NS | H | NS | H |
| UTTAR PRADESH | MAU | NS | L | NS | NS | NS | NS | NS | NS | NS | NS | NS | NS | NS |
| UTTAR PRADESH | BALLIA | H | NS | NS | NS | NS | NS | NS | NS | NS | NS | NS | NS | NS |
| UTTAR PRADESH | JAUNPUR | H | NS | NS | NS | NS | H | NS | H | H | NS | NS | H | NS |
| UTTAR PRADESH | GHAZIPUR | NS | NS | NS | NS | NS | NS | H | NS | NS | NS | NS | NS | NS |
| UTTAR PRADESH | CHANDAULI | NS | NS | NS | NS | NS | NS | NS | NS | NS | NS | NS | H | NS |
| UTTAR PRADESH | VARANASI | NS | NS | L | NS | NS | NS | NS | NS | NS | NS | H | NS | NS |
| UTTAR PRADESH | SANT RAVIDAS NAGAR (BHADOHI) | NS | NS | NS | NS | NS | NS | NS | NS | NS | NS | NS | NS | NS |
| UTTAR PRADESH | MIRZAPUR | NS | NS | NS | NS | NS | NS | H | NS | H | NS | H | NS | NS |
| UTTAR PRADESH | SONBHADRA | NS | NS | NS | NS | NS | H | NS | NS | NS | NS | H | NS | NS |
| UTTAR PRADESH | ETAH | NS | NS | NS | NS | H | H | NS | L | NS | NS | NS | NS | NS |
| UTTAR PRADESH | KANSHIRAM NAGAR | NS | NS | NS | NS | NS | NS | NS | NS | NS | NS | NS | NS | NA |
| UTTAR PRADESH | AMETHI | NS | NS | NS | NS | NS | NS | NS | NS | NS | NS | NS | NS | NS |
| UTTAR PRADESH | BUDAUN | NS | NS | NS | NS | NS | NS | NS | NS | NS | NS | H | NS | NS |
| UTTAR PRADESH | GHAZIABAD | NS | L | NS | NS | NS | NS | NS | H | NS | NS | NS | NS | H |
| UTTAR PRADESH | HAPUR | NS | NS | NS | NS | NS | NS | NS | L | NS | NS | NS | H | NS |
| UTTAR PRADESH | MORADABAD | H | L | NS | NS | NS | NS | NS | NS | NS | NS | NS | NS | NS |
| UTTAR PRADESH | MUZAFFARNAGAR | NS | L | L | NS | NS | NS | H | NS | NS | NS | H | NS | NS |
| UTTAR PRADESH | RAE BARELI | H | NS | NS | NS | NS | NS | NS | NS | NS | NS | NS | NS | NS |
| UTTAR PRADESH | SAMBHAL | NS | NS | NS | NS | NS | NS | NS | NS | NS | NS | H | NS | NS |
| UTTAR PRADESH | SHAMLI | NS | NS | NS | NS | NS | NS | NS | NS | NS | NS | NS | NS | NS |
| UTTAR PRADESH | SULTANPUR | NS | NS | NS | NS | NS | NS | NS | NS | NS | NS | NS | H | NS |
| UTTARAKHAND | UTTARKASHI | NS | NS | NS | NS | NS | NS | NS | NS | NS | NS | NS | NS | NS |
| UTTARAKHAND | CHAMOLI | NS | NS | NS | NS | NS | NS | NS | NS | NS | NS | NS | NS | NS |
| UTTARAKHAND | RUDRAPRAYAG | NS | NS | NS | NS | NS | NS | NS | NS | NS | NS | NS | NS | NS |
| UTTARAKHAND | TEHRI GARHWAL | NS | NS | NS | NS | NS | NS | NS | NS | NS | NS | NS | NS | NS |
| UTTARAKHAND | DEHRADUN | H | NS | NS | NS | NS | NS | NS | H | NS | NS | NS | NS | NS |
| UTTARAKHAND | GARHWAL | NS | NS | NS | NS | NS | NS | NS | NS | NS | NS | NS | NS | NS |
| UTTARAKHAND | PITHORAGARH | NS | NS | NS | NS | NS | NS | NS | NS | NS | NS | NS | NS | NS |
| UTTARAKHAND | BAGESHWAR | NS | NS | NS | NS | NS | NS | NS | NS | NS | NS | NS | NS | NS |
| UTTARAKHAND | ALMORA | NS | NS | NS | NS | NS | NS | NS | NS | NS | NS | NS | NS | NS |
| UTTARAKHAND | CHAMPAWAT | NS | NS | NS | NS | NS | NS | NS | NS | NS | NS | NS | NS | NS |
| UTTARAKHAND | NAINITAL | NS | NS | NS | NS | NS | NS | NS | NS | NS | NS | NS | NS | NS |
| UTTARAKHAND | UDHAM SINGH NAGAR | H | NS | NS | NS | L | NS | NS | NS | NS | NS | NS | NS | NS |
| UTTARAKHAND | HARDWAR | H | NS | NS | NS | NS | NS | NS | NS | NS | NS | NS | NS | NS |
| WEST BENGAL | DARJILING | NS | L | NS | NS | NS | NS | NS | NS | NS | NS | NS | H | NS |
| WEST BENGAL | JALPAIGURI | H | L | NS | L | NS | NS | NS | L | NS | H | NS | H | NS |
| WEST BENGAL | KOCH BIHAR | H | L | L | NS | NS | NS | NS | NS | NS | NS | NS | H | H |
| WEST BENGAL | UTTAR DINAJPUR | NS | L | L | NS | NS | NS | NS | NS | NS | NA | H | NS | H |
| WEST BENGAL | DAKSHIN DINAJPUR | H | NS | NS | NS | NS | NS | NS | NS | NS | H | H | H | NS |
| WEST BENGAL | MALDAH | NS | NS | NS | NS | NS | NS | NS | NS | NS | NS | H | L | H |
| WEST BENGAL | MURSHIDABAD | H | NS | L | NS | NS | H | NS | H | NS | NS | NS | NS | H |
| WEST BENGAL | BIRBHUM | H | NS | NS | H | H | H | NS | NS | NS | H | NS | NS | H |
| WEST BENGAL | NADIA | H | L | NS | NS | NS | L | NS | NS | L | NS | H | H | NS |
| WEST BENGAL | NORTH TWENTY FOUR PARGANAS | H | H | L | L | NS | L | NS | H | H | NS | H | H | NS |
| WEST BENGAL | HUGLI | H | NS | NS | NS | H | H | NS | L | NS | NS | NS | NS | H |
| WEST BENGAL | BANKURA | H | NS | NS | H | NS | NS | L | NS | L | NS | H | NS | NS |
| WEST BENGAL | PURULIYA | H | NS | NS | NS | NS | H | NS | NS | H | NS | NS | H | NS |
| WEST BENGAL | HAORA | H | NS | NS | H | NS | H | NS | NS | NS | NS | L | H | H |
| WEST BENGAL | KOLKATA | H | NS | H | NS | NS | NS | NS | NA | NS | NS | H | NS | NS |
| WEST BENGAL | SOUTH TWENTY FOUR PARGANAS | NS | H | NS | NS | H | NS | NS | NS | NS | H | H | H | H |
| WEST BENGAL | PASCHIM MEDINIPUR | NS | NS | L | NS | H | NA | NS | L | L | NS | NS | H | NS |
| WEST BENGAL | PURBA MEDINIPUR | H | L | NS | L | L | NS | H | L | L | NS | H | H | H |
| WEST BENGAL | PASCHIM BARDDHAMAN | H | NS | NS | NS | NS | L | NS | L | NS | H | H | H | H |
| WEST BENGAL | PURBA BARDDHAMAN | H | H | NS | H | NS | H | H | L | NS | NS | NS | H | NS |
| H% |  | **33.0** | **1.4** | **2.0** | **3.2** | **4.5** | **5.9** | **6.2** | **8.0** | **4.8** | **8.3** | **17.5** | **17.1** | **10.4** |
| L% |  | **0.3** | **14.2** | **6.2** | **4.5** | **3.9** | **2.7** | **2.9** | **6.5** | **2.7** | **1.4** | **0.8** | **1.1** | **0.2** |

*H: Higher odds [Adjusted odds ratio(AOR) >1 and P<0.05], L: Lower odds (AOR<1 and P<0.05), NS: Not Significant (p>0.05), NA: Data Not Available.*

*Districts from 9 Non significant states excluded from table (ie., Chandigarh, Sikkim, Arunachal Pradesh, Mizoram, Dadra & Nagar Haveli And Daman & Diu, Lakshadweep, Puducherry, Andaman & Nicobar Islands, Ladakh)*

## **Table S13: Operational definitions (National Family Health Survey-5, India, 2019-2021)**

| **Sl.no** | **Terms** | **Definition/collected as per NFHS 5 survey** |
| --- | --- | --- |
| **Socio-demographic factors** | | |
| 1. | Education | Respondents who have completed standard nine (nine years of formal schooling) or higher were assumed to be literate. All other respondents were given a sentence to read, and they were considered to be literate if they could read all or part of the sentence. |
| 2. | Employment | Respondents who were employed in the seven days before the survey. Includes respondents who did not work in the past seven days but who are regularly employed and were absent from work for leave, illness, vacation, or any other such reasons |
| 3. | Household wealth index | Households are given scores based on the number and kinds of consumer goods they own, ranging from a television to a bicycle or car, and housing characteristics such as source of drinking water, toilet facilities, and flooring materials. These scores are derived using principal component analysis. National wealth quintiles are compiled by assigning the household score to each usual (de jure) household member, ranking each person in the household population by their score, and then dividing the distribution into five equal categories, each with 20 percent of the population. |
| **Behavioral risk factors** | | |
| 4. | Tobacco consumption | Responded yes to the question Do you currently smoke or use tobacco in any other form? On the day of survey |
| 5. | Alcohol consumption | Responded yes to question.  Do you drink alcohol? On the day of survey |
| **Anthropometric and metabolic factors** | | |
| 6. | Body Mass Index (BMI): | In the NFHS-5 survey, BMI is defined as the individual's weight in kilograms divided by the square of their height in meters (kg/m²). This value serves as a standardized indicator of weight relative to height and is categorized according to the Asian BMI cut-off  BMI Category & BMI Range  Normal (18.5 - 22.9)  Underweight (Lowest - 18.4)  Overweight (23.0 - 24.9)  Obese (25 and above) |
| 7. | Weight: | Measured using calibrated electronic weighing scales with a capacity of 200 kg.  Participants removed shoes and heavy clothing before stepping on the scale. |
| 8. | Height: | Measured using Seca 213 stadiometers for adults.  Individuals stood barefoot on the platform with head and shoulder blades touching the vertical scale. |
| 9. | Waist Circumference: | Measured using a non-stretchable Gulick tape at the midpoint between the lowest rib and the iliac crest (upper hip bone).  Measurement was taken during normal breathing after exhalation, without tightening the tape. |
| 10. | Central obesity | Central obesity: waist circumference (WC) ≥ 90cmfor men and ≥80 cmfor women |
